# Total evidence phylogeny and evolutionary timescale for Australian faunivorous marsupials (Dasyuromorphia)

**DOI:** 10.1186/s12862-017-1090-0

**Published:** 2017-12-04

**Authors:** Shimona Kealy, Robin Beck

**Affiliations:** 1 Archaeology and Natural History, School of Culture, History and Language, College of Asia and the Pacific, Australian National University, Acton, ACT Australia; 2 School of Environment and Life Sciences, University of Salford, Salford, M5 4WT UK

**Keywords:** Dasyuromorphia, Dasyuridae, *Myrmecobius*, Thylacinidae, Marsupial, Total evidence, Divergence times, Miocene, Middle Miocene Climatic Transition, Australia

## Abstract

**Background:**

The order Dasyuromorphia is a diverse radiation of faunivorous marsupials, comprising >80 modern species in Australia and New Guinea. It includes dasyurids, the numbat (the myrmecobiid *Myrmecobius fasciatus*) and the recently extinct thylacine (the thylacinid *Thylacinus cyncocephalus*). There is also a diverse fossil record of dasyuromorphians and “dasyuromorphian-like” taxa known from Australia. We present the first total evidence phylogenetic analyses of the order, based on combined morphological and molecular data (including a novel set of 115 postcranial characters), to resolve relationships and calculate divergence dates. We use this information to analyse the diversification dynamics of modern dasyuromorphians.

**Results:**

Our morphology-only analyses are poorly resolved, but our molecular and total evidence analyses confidently resolve most relationships within the order, and are strongly congruent with recent molecular studies. Thylacinidae is the first family to diverge within the order, and there is strong support for four tribes within Dasyuridae (Dasyurini, Phascogalini, Planigalini and Sminthopsini). Among fossil taxa, *Ankotarinja* and *Keeuna* do not appear to be members of Dasyuromorphia, whilst *Barinya* and *Mutpuracinus* are of uncertain relationships within the order. Divergence dates calculated using total evidence tip-and-node dating are younger than both molecular node-dating and total evidence tip-dating, but appear more congruent with the fossil record and are relatively insensitive to calibration strategy. The tip-and-node divergence dates indicate that Dasyurini, Phascogalini and Sminthopsini began to radiate almost simultaneously during the middle-to-late Miocene (11.5–13.1 MYA; composite 95% HPD: 9.5–15.9 MYA); the median estimates for these divergences are shortly after a drop in global temperatures (the middle Miocene Climatic Transition), and coincide with a faunal turnover event in the mammalian fossil record of Australia. Planigalini radiated much later, during the latest Miocene to earliest Pliocene (6.5 MYA; composite 95% HPD: 4.4–8.9 MYA); the median estimates for these divergences coincide with an increase in grass pollen in the Australian palynological record that suggests the development of more open habitats, which are preferred by modern planigale species.

**Conclusions:**

Our results provide a phylogenetic and temporal framework for interpreting the evolution of modern and fossil dasyuromorphians, but future progress will require a much improved fossil record.

**Electronic supplementary material:**

The online version of this article (10.1186/s12862-017-1090-0) contains supplementary material, which is available to authorized users.

## Background

Dasyuromorphia is the second most speciose order of Australian and New Guinean marsupials, after Diprotodontia (mainly herbivorous forms such as possums, kangaroos, wombats and the koala): more than 80 modern dasyuromorphian species have been named to date, and new species continue to be identified [1–9]. Dasyuromorphians are predominantly faunivorous, but they exhibit considerable ecomorphological diversity [3, 4]. The body masses of living dasyuromorphians span a range of more than three orders of magnitude, from the world’s smallest living marsupial, the long-tailed planigale (*Planigale ingrami*, body mass ~4 g), to the largest living carnivorous marsupial, the Tasmanian devil (*Sarcophilus harrisii*, body mass > 8 kg) [3, 4]. This range is even greater when the recently extinct thylacine (*Thylacinus cynocephalus*), which weighed up to 35 kg [4], is considered. Dasyuromorphia also includes the only known marsupial specialised for feeding on social insects, the numbat (*Myrmecobius fasciatus*), as well as a hopping form, the kultarr (*Antechinomys laniger*) [4]. Dasyuromorphian reproduction is also of interest: several dasyurid species are unusual among mammals in exhibiting semelparity, the males dying after a single breeding season [10, 11].

Modern dasyuromorphians are currently classified as comprising three families, of which two are monotypic: Myrmecobiidae (*Myrmecobius fasciatus*), Thylacinidae (*Thylacinus cynocephalus*) and Dasyuridae (the remaining species) [1, 4, 9]. There have been numerous published molecular studies of dasyuromorphian phylogeny, and these have confidently resolved many relationships within the order e.g. [8, 12–21]. For example, within Dasyuridae, the composition and branching order of the four currently recognised tribes (Dasyurini, Phascogalini, Planigalini and Sminthopsini) seem robustly resolved, as do several of the relationships within these clades. Inevitably, however, such molecular studies lack fossil taxa.

Numerous fossil dasyuromorphians and “dasyuromorphian-like” taxa have been described from various sites in Australia and New Guinea e.g. [22–35], including at least one entirely extinct family, the “hammer-toothed” malleodectids [36, 37]. At present, the oldest generally accepted crown-clade dasyuromorphian to be named appears to be *Badjcinus turnbulli* from Faunal Zone A deposits at Riversleigh (currently interpreted as late Oligocene in age [38, 39]), which is currently classified as a thylacinid [29, 40, 41]. However, the affinities of many other fossil taxa are unclear, largely because most are known only from dental remains; dasyuromorphians retain a relatively plesiomorphic dentition, and there is a general lack of obvious dental synapomorphies for Dasyuromorphia and for subclades within the order (notably the family Dasyuridae) [24, 42, 43].

A few phylogenetic analyses of dasyuromorphian relationships based on morphological data have been published [29, 41, 44–46], and Archer et al. [36] presented a “molecular scaffold” analysis using a morphological dataset modified from these earlier studies. However, all of these have suffered from limited taxon sampling. In addition, they have been based solely on characters of the skull and dentition, and unconstrained analyses show several areas of conflict with molecular phylogenies [13, 15].

Collectively, these molecular and morphological studies have improved our understanding of dasyuromorphian phylogeny, but a number of key issues remain unresolved. Particularly important are determining exactly which putative fossil dasyuromorphian and “dasyuromorphian-like” taxa belong to Dasyuromorphia (and, if so, whether they are stem- or crown-members), and also determining whether the referral of fossil taxa to modern genera is justified or not. A major stumbling block to resolving these issues has been a failure to combine available molecular and morphological data in a total evidence approach. Phylogenies based on morphological data alone are often poorly resolved, incongruent with molecular phylogenies, or both (as has been the case with published morphological phylogenies of dasyuromorphians [13, 15, 44, 46]); despite this, morphology can provide additional “hidden” support [47] for clades strongly supported by molecular data when the two datatypes are analysed in combination [48]. Morphological data may also help robustly resolve relationships in parts of the phylogeny where the phylogenetic signal in molecular data is weak, for example in the case of deep, closely spaced divergences [49]. Conversely, simulations have shown that the inclusion of molecular data for extant taxa can improve the accuracy of phylogenetic estimation of fossil taxa for which molecular data is unavailable [50].

To date, the morphological evidence used in phylogenetic analyses of dasyuromorphians has been restricted to characters from the skull and dentition. Other anatomical systems should provide additional information, with the most obvious candidate being the postcranial skeleton: postcranial characters have already been shown to be highly informative for resolving the phylogeny of various other marsupial clades [51–59] and so may be similarly useful for relationships within Dasyuromorphia.

The timing of diversification within Dasyuromorphia, particularly the radiation of modern dasyurids, is also controversial. A strict reading of the fossil record [24, 27, 28, 60] suggests that modern dasyurids probably did not begin to radiate widely until the middle-to-late Miocene, with some early molecular studies reaching similar conclusions e.g. [14]. At present, the oldest described fossil remains that can be convincingly referred to modern dasyurid genera are from the early Pliocene [24, 27, 28]. However, the most recent broadscale molecular analysis of dasyuromorphian phylogeny and divergence dates supported a much earlier diversification of modern dasyurids, with the tribes estimated as beginning to radiate in the early Miocene and all modern genera having originated by the middle Miocene [15].

There are several explanations for this apparent discrepancy. Despite ongoing fieldwork, the fossil record of dasyuromorphians and other Australian mammals remains highly incomplete [24, 27, 28]. Putative phascogalins and dasyurins have been reported from Faunal Zone B deposits at Riversleigh (currently interpreted as early Miocene in age, ~23.03–15.97 MYA) [28, 38, 39], which would markedly extend the records of these tribes, but these specimens are not yet described [28]. The general lack of obvious dental synapomorphies for Dasyuridae and dasyurid subclades also means that, even if found, fossils belonging to modern dasyurid lineages might not be identified as such, particularly if known from isolated teeth alone.

Conversely, molecular divergence dates should not be viewed uncritically, because they can be affected by a range of factors. These include the choice of clock model, the choice and number of fossils used to calibrate particular nodes, and the way in which those calibrations are specified, i.e. either as point estimates or different prior probability distributions [61–63]. Particularly problematic is the issue of maximum age constraints, which are difficult to specify objectively and yet are likely to have a major impact on resultant divergence time estimates [64–67]. Also of importance is the choice of tree branching prior: a uniform (= pure-birth or Yule) prior can result in much older divergence estimates than a birth-death prior, with the latter more appropriate for clades in which extinction has occurred [68].

A promising alternative approach to “node dating” with molecular clock models is “tip dating”, which allows phylogenetic relationships and divergence times of fossil and extant taxa to be inferred simultaneously in the context of a single analysis [69–72], rather than using fossil taxa as a priori calibrations. However, in several cases tip dating has been shown to result in unrealistically ancient divergence dates, particularly when a uniform tree branching prior was used [72–75], rather than a biologically more plausible fossilised birth-death (FBD) prior [either with or without the possibility of sampling ancestors; [70, 74, 76, 77]. Recently, it has been argued that node dating and tip dating should be combined into a hybrid “tip-and-node dating” approach, in which minimum age constraints of selected nodes are specified by a priori fossil calibrations, and maximum age constraints for nodes resulting from the interaction between node calibrations and fossil tips [72, 78].

Here we present morphological, molecular and first total evidence analyses of modern and fossil dasyuromorphians, using maximum parsimony (for the morphological data only), and undated and dated Bayesian analysis. The dated Bayesian analyses employed three different approaches: node dating using a molecular clock, tip dating using a total evidence clock, and tip-and-node dating using a total evidence clock [72, 78], in each case using the FBD prior and allowing for the possibility of sampled ancestors [70, 74, 76, 77]. We used the resultant phylogenies to infer relationships within Dasyuromorphia and probable divergence times. We investigate the pattern of diversification for the order implied by our tip-and-node dating analyses using Lineage Through Time (LTT) plots, and compare these against palaeoenvironmental change and patterns of faunal change in the fossil record. Finally, we use Bayesian Analysis of Macroevolutionary Mixtures (BAMM) to identify whether there is evidence for one or more statistically significant shifts in the rate of diversification within Dasyuromorphia, and to calculate speciation and extinction rates.

## Methods

### Systematics

To minimise ambiguity when discussing the phylogeny of dasyuromorphians, we propose formal phylogenetic definitions for Dasyuromorphia, and for clades within Dasyuromorphia that have been consistently recognised in previous studies and that have received strong support in recent phylogenetic analyses (Table 1). For higher level systematics we follow Aplin and Archer [79], Beck et al. [80], Jackson and Groves [9], and Beck [81].

**Table 1:** Formal phylogenetic definitions proposed here for Dasyuromorphia and selected dasyuromorphian subclades

Clade	Definition	Definition type
Dasyuromorphia	the most inclusive clade including *Dasyurus viverrinus*, but excluding *Perameles nasuta*, *Notoryctes typhlops*, *Phalanger orientalis* and *Dromiciops gliroides*	stem
Dasyuroidea	the least inclusive clade including *Dasyurus viverrinus*, *Myrmecobius fasciatus* and *Thylacinus cynocephalus*	crown
Dasyuridae	the most inclusive clade including *Dasyurus viverrinus*, but excluding *Myrmecobius fasciatus* and *Thylacinus cynocephalus*	stem
Myrmecobiidae	the most inclusive clade including *Myrmecobius fasciatus*, but excluding *Dasyurus viverrinus* and *Thylacinus cynocephalus*	stem
Thylacinidae	the most inclusive clade including *Thylacinus cynocephalus*, but excluding *Dasyurus viverrinus* and *Myrmecobius fasciatus*	stem
Dasyurinae	the most inclusive clade including *Dasyurus viverrinus*, but excluding *Sminthopsis crassicaudata*	stem
Sminthopsinae	the most inclusive clade including *Sminthopsis crassicaudata*, but excluding *Dasyurus viverrinus*	stem
Dasyurini	the most inclusive clade including *Dasyurus viverrinus*, but excluding *Phascogale tapoatafa*, *Planigale ingrami* and *Sminthopsis crassicaudata*	stem
Phascogalini	the most inclusive clade including *Phascogale tapoatafa,* but excluding *Dasyurus viverrinus*, *Planigale ingrami* and *Sminthopsis crassicaudata*	stem
Planigalini	the most inclusive clade including *Planigale ingrami*, but excluding *Dasyurus viverrinus*, *Phascogale tapoatafa* and *Sminthopsis crassicaudata*	stem
Sminthopsini	the most inclusive clade including *Sminthopsis crassicaudata*, but excluding *Dasyurus viverrinus*, *Phascogale tapoatafa* and *Planigale ingrami*	stem

### Taxon sampling for morphological data

For our morphological dataset, we included at least one representative of each currently-recognised modern genus [1, 15]. We specifically selected our modern dasyuromorphian terminals to take into account possible generic non-monophyly. Thus, we included representatives of all five genera recognised by Van Dyck [46] within *Murexia sensu* *lato* - namely *Micromurexia hageni, Murexia longicaudata*, *Murexechinus melanurus*, *Paramurexia rothschildi* and *Phascomurexia naso* – because Van Dyck’s [46] morphological analyses failed to group these genera in a clade, although we note that molecular data strongly support monophyly of *Murexia sensu* *lato* [13, 15, 20, 21]. Within *Sminthopsis*, we included a representative of both the ‘Macroura’ group (*S. crassicaudata*) and the ‘Murina’ group (*S. murina*), to test the possibility that *Sminthopsis* might be paraphyletic with respect to either *Antechinomys*, *Ningaui*, or both [15, 19, 21]. We included *Parantechinus apicalis*, *Pseudantechinus* (= ‘*Parantechinus*’) *bilarni* and *Ps. maccdonnellensis*, because the precise relationships between these three taxa have been controversial [16, 21, 22, 82]. Finally, we included three extant representatives of *Dasyurus* (*D. albopunctatus*, *D. hallucatus* and *D. maculatus*) to test the possibility that *Dasyurus* is paraphyletic with regard to *Sarcophilus*, as found in several morphological analyses [29, 41, 44]. This resulted in a total of 31 modern dasyuromorphian terminals (Additional file 1: Table S1).

We also included 13 fossil terminals that have previously been identified as dasyuromorphians or “dasyuromorphian-like” taxa (Additional file 1: Table S1). To ensure reasonable character overlap between taxa, we included only named fossil taxa that are currently known from at least one upper molar and one lower molar. Among the “dasyuromorphian-like” taxa, we included *Ankotarinja tirarensis* and *Keeuna woodburnei* from the late Oligocene [26, 83, 84] Ditjimanka Local Fauna from the Etadunna Formation of central South Australia, which were originally described as dasyurids [85], but more recently have been referred to Marsupialia *incertae sedis* [24, 86]. We also included *Djarthia murgonensis* from the early Eocene Tingamarra Fauna of southeastern Queensland, which appears to be a plesiomorphic australidelphian [58, 59, 86].

For our outgroup terminals, we used representatives of the following modern marsupial orders: Peramelemorphia (the extant *Echymipera kalubu* and *Perameles nasuta*, and the fossil *Yarala burchfieldi*), which is a close relative of Dasyuromorphia within the superorder Agreodontia [80]; Microbiotheria (the extant *Dromiciops gliroides*), which is the closest modern relative of Australian marsupials [87]; and Didelphimorphia (the extant *Caluromys philander*, *Didelphis virginiana*, and *Marmosa murina*), which are relatively plesiomorphic non-australidelphian marsupials [88]. We also included three well-preserved fossil stem-marsupials, all from the early or middle Palaeocene Tiupampa Fauna in Bolivia [89–92]: *Andinodelphys cochambambensis*, *Mayulestes ferox* and *Pucadelphys andinus*. Our final morphological matrix comprised 54 taxa (Additional file 1: Table S1).

### Morphological characters

Our craniodental characters were modified from those of Wroe et al. [44], Wroe and Musser [41] and Murray and Megirian [29]. We reviewed all 77 original characters defined by Wroe et al. [44], and excluded those that appeared ambiguous or that we could not score consistently. We also modified several of the remaining characters and character states to better correspond to our observations, resulting in a final set of 58 craniodental characters. The 16 multistate characters that represented apparent morphoclines were ordered. We scored these characters for the additional terminals not present in the Wroe et al. [44], Wroe and Musser [41] and Murray and Megirian [29] matrices. Where possible, we also reassessed the original scorings of the other terminals, and revised some character scores as a result. In general, we scored our characters based on direct observations of actual specimens or high quality casts; however, for some taxa (e.g. the Tiupampan stem-marsupials) we used published descriptions [89, 91–94]. Where possible, we examined multiple specimens for each species, with up to six specimens per species examined. In cases of intraspecific polymorphism, the modal condition was scored if one character state clearly predominated, following Voss and Jansa [88, 95]. If the different character states were approximately equally common, the character was scored as polymorphic.

In addition to our craniodental characters, we devised a novel set of postcranial characters intended to resolve dasyuromorphian phylogeny. The postcranial characters of Horovitz and Sánchez-Villagra [55] and Flores [57] were used for an initial assessment of variability within the marsupial postcranium. This was combined with information from other studies of postcranial morphology in marsupials and other metatherians [52, 53, 93, 96–106] and with additional personal observations to develop 115 characters that document the major morphological variability we observed among our taxon set. Thirty-one multistate characters that represented probable morphoclines were specified as ordered. Due to a lack of postcranial specimens, we were able to score these characters for only a subset of our morphological taxon sample (32 out of 54 terminals; Additional file 1: Table S1), and for most of these we were only able to examine one or two specimens. A list of our revised morphological characters and specimens examined to score these is given in Additional file 2: Text S2. The final morphological matrix (in Nexus format) is given in Additional file 3: Text S3. The morphological matrix is also available from Morphobank (Project 858: http://morphobank.org/permalink/?P858).

### Taxon sampling for molecular data

We obtained molecular data for all the 31 modern dasyuromorphian terminals and the six modern outgroup terminals that were scored for morphological characters, plus an additional 41 extant dasyurid species. This total of 72 dasyuromorphian species represents approximately 84% of named modern species (however, we note that additional species undoubtedly remain to be described, for example within *Planigale*) [8]. Our final molecular matrix included 78 taxa (Additional file 1: Table S1).

### Molecular sequence data

We downloaded existing DNA sequence data from the online GenBank database for seven mitochondrial genes, namely the protein-coding cytochrome b (*MT-CYB*), cytochrome oxidase I (*MT-CO1*), cytochdrome oxidase II (*MT-CO2*), NADH:ubiquinone oxidoreductase core subunit 1 (*MT-ND1*) and NADH:ubiquinone oxidoreductase core subunit 2 (*MT-ND2*) genes, and the ribosomal 12S RNA (*MT-RNR1*), and 16S RNA (*MT-RNR2*) genes, and eight nuclear protein-coding genes or gene fragments, namely exon 26 of apolipoprotein B (*APOB*), intron 7 of fibrinogen beta chain (*FGB*), exon 10 of BRCA1, DNA repair associated (*BRCA1*), haemoglobin subunit epsilon 1 (*HBE1*), exon 1 of retinol binding protein 3 (*RBP3*; often called interphotoreceptor retinoid binding protein, or *IRBP*), protamine 1 (*PRM1*), recombination activating 1 (*RAG1*) and exon 28 of von Willebrand factor (*VWF*). These genes were selected based on their use in previous molecular studies of dasyuromorphian phylogeny [8, 13, 15–17, 19], which means that there is good coverage for our modern taxon sample. In general, where more than one sequence of the same gene was available for a single species, the most recent and/or complete sequence was selected. A full list of Genbank accession numbers for the sequences used is given in Additional file 1: Table S1.

All protein-coding genes were aligned in MEGA 6.0 [107], using default settings for the alignment algorithm MUSCLE [108]. Intronic sequences were aligned using the standard MUSCLE algorithm, whilst exonic sequences were aligned using MUSCLE for codons. *HBE1* and *PRM1* were both subdivided into intronic and exonic sequences, and these were aligned separately. The mitochondrial ribosomal genes *MT-RNR1* and *MT-RNR2* were aligned manually in BioEdit 7.1.9 [109], with secondary structure (i.e. stems and loops) taken into account, based on published structures [110, 111] and the online RNA database OGRe [112]. After alignment, the aligned sequences of all genes and gene fragments were concatenated into a single matrix of ~16.4 kb. The final molecular matrix (in Nexus format) is given in Additional file 3: Text S3.

### Taxon sampling for total evidence data

We combined our morphological and molecular matrices to produce a total evidence matrix that included all taxa represented by morphological data and all taxa represented by molecular data. Where possible, we avoided creating supraspecific hybrids when combining the morphological and molecular data. The sole exception was *Micromurexia*, for which our morphological characters were scored using *M. hageni*, whereas the molecular sequence data represented *M. habbema*. Our final total evidence matrix included 95 taxa, with 17 fossil taxa represented by morphological data only, and 41 modern taxa by molecular data only (Additional file 1: Table S1). The final total evidence matrix (in Nexus format) is given in Additional file 3: Text S3.

### Maximum parsimony analyses

We carried out maximum parsimony analysis of the morphological matrix using TNT version 1.5 [113, 114]. Our maximum parsimony tree searches comprised an initial “new technology” search using ratchet, drift and tree fusing, until the same minimum length had been hit 100 times, followed by a “traditional” search using tree bisection-reconnection branch swapping among the trees saved from the initial search. All most parsimonious trees were saved, and then summarised using strict consensus. Bootstrap values were calculated in TNT as absolute frequencies, based on 250 replicates.

### Undated Bayesian analyses

We carried out undated, model-based Bayesian analyses using MrBayes 3.2 [115]. We analysed our morphological matrix using a single Mk model applied to the morphological characters [116], with the assumption that only variable characters were scored, and with a gamma distribution to model rate heterogeneity across characters, i.e. the Mkv + G model. For our molecular sequence data, we first used PartitionFinder v1.1.1 [117] to identify an appropriate partitioning scheme and models for each partition. The molecular sequences were initially partitioned by gene, codon position (for exonic sequences of protein-coding genes), and stem and loop regions (for the ribosomal genes *MT-RNR1* and *MT-RNR2*); intronic sequences were not partitioned further. For the PartitionFinder analysis, we restricted comparisons to models implemented by MrBayes, with the assumption of linked branch lengths, the “greedy” search algorithm, and with the Bayesian Information Criterion used for model selection, as preferred by Lanfear et al. [117]. The MrBayes analysis then applied the best-fitting partitioning scheme and models identified by PartitionFinder. We also carried out analyses of the nuclear genes only and the mitochondrial genes only, again using PartitionFinder to identify the best-fitting partitioning scheme and models. Finally, we carried out an undated Bayesian analysis of the total evidence matrix, using the same models as for the morphological and combined molecular analyses.

All undated Bayesian analyses comprised four runs of four chains (one cold, three heated) each, sampling trees every 5000 generations. The morphological and molecular analyses were run for 10 million generations, whilst the total evidence analysis was run for 20 million generations. For all three analyses, the MrBayes output was examined in Tracer v1.6 [118] to identify when stationarity and convergence between chains had been reached. The post-burn-in trees were summarised using 50% majority rule consensus, with Bayesian posterior probabilities (BPPs) as support values.

### Identification of unstable taxa

Analysis of the morphological matrix using maximum parsimony and undated Bayesian analysis resulted in relatively unresolved consensus trees, particularly for relationships within Dasyuridae. The Roguenarok algorithm [119] was therefore used to identify the most unstable taxa in each analysis; these taxa were then deleted and the analyses re-run.

### Molecular node-dating

To estimate divergence times within Dasyuromorphia, we carried out dated Bayesian analyses of the molecular dataset using node dating. We used a single Independent Gamma Rates (IGR) clock model, implementing a fossilised birth-death tree branching prior that assumed “diversity” sampling [70] and a sample probability of 0.8 for our modern taxa; this value is slightly less than the proportion of named dasyuromorphian species in our matrices (0.84), but allows for the existence of a few additional undescribed species see e.g. [8]. Because the molecular analyses include modern taxa only, the fossilisation prior was fixed as 0. For the molecular analyses, we employed six node calibrations, of which two were within Dasyuromorphia and four outside this clade. Details of these node calibrations are given in Additional file 4: Text S4.

Node calibrations can be implemented in a variety of ways, either as point estimates or as different types of probability distributions; the different implementations make different assumptions regarding the quality of the fossil record, which can have a major impact on the divergence dates estimated using those calibration(s) [61]. To investigate the impact of these, we implemented our node calibrations in two different ways. In the first scheme (NodeCalib1), all six node calibrations were specified as offset exponential distributions, with a “hard” minimum bound, and a “soft” maximum bound such that there was a 5% probability that the divergence date is older than this; this scheme assumes that the divergence date falls relatively close to the minimum bound [61]. In the second scheme (NodeCalib2), node calibrations 1 (=Didelphimorphia-Australidelphia split, i.e. the root), 2 (= crown-clade Didelphidae), 3 (= crown-clade Australidelphia) and 5 (= crown-clade Dasyuromorphia or Dasyuroidea) were specified as uniform distributions with “hard” minimum and maximum bounds, reflecting the particularly poor or uncertain fossil records of these groups, whilst node calibrations 4 (= crown-clade Peramelidae) and 6 (= crown-clade Dasyuridae) were maintained as offset exponential distributions (see Additional file 4: Text S4). The molecular node-dating analyses were run for 20 million generations, with MrBayes settings as for the undated molecular analysis.

### Total evidence tip-dating

In addition to the molecular node dating analyses, we estimated divergence times within Dasyuromorphia, using tip dating of the total evidence data [69–72]. As in the molecular node-dating analyses, we used a single IGR clock model, and assumed “diversity” sampling with a sample probability of 0.8 for our modern taxa. The fossilisation, extinction and speciation priors used the MrBayes default values. Tip dating requires that each terminal is specified an age, either as a point estimate or a range. Our Recent terminals were all assigned an age of 0 Ma, whereas fossil taxa were assigned age ranges based on the published literature and Gradstein et al. [120]. A full list of taxon ages and references for these is given in Additional file 5: Text S5. The age ranges of the fossil taxa were specified as uniform distributions.

To ensure comparability with the molecular node dating, we also included two node calibrations (see Additional file 4: Text S4). The first of these corresponds to node calibration 1 (= Didelphimorphia-Australidelphia split) in the molecular node dating analysis. A further calibration, node calibration 7, was placed on the root node (= the split between the Tiupampan stem-marsupials *Andinodelphys*, *Mayulestes* and *Pucadelphys*, and the remaining taxa), as is usual for tip-dating analyses [74]. Similarly to the molecular node dating analyses, we implemented these two node calibrations either as offset exponential distributions, with a ‘hard’ minimum bound and a “soft” maximum bound such that there was a 5% probability that the divergence date is older than this (TipCalib1), or as uniform distributions with “hard” minimum and maximum bounds (TipCalib2). The two total evidence tip dating analyses were both run for 50 million generations, with MrBayes settings otherwise the same as for the undated and node dating molecular analyses.

### Total evidence tip-and-node dating

O’Reilly and Donoghue [78] argued in favour of combining tip and node calibrations, concluding that this “makes the best use of palaeontological data in the construction of evolutionary timescales”. We therefore implemented total evidence tip-and-node dating analyses by combining the tip calibrations and all seven of the node calibrations discussed above (see Additional file 4: Text S4 and Additional file 5: Text S5). Similarly to the total evidence tip dating analyses, we used a single IGR clock model, we assumed “diversity” sampling with a sample probability of 0.8 for our modern taxa, and used the MrBayes default values for fossilisation, extinction and speciation priors, with age ranges of fossil taxa specified as uniform distributions. Similarly to the molecular node dating and total evidence tip dating analyses (see above), the node calibrations were either all implemented as offset exponential distributions (TipNodeCalib1), or with node calibrations 1 (= Didelphimorphia-Australidelphia split), 2 (= crown-clade Didelphidae), 3 (= crown-clade Australidelphia) and 5 (= crown-clade Dasyuromorphia) from the molecular node dating analyses and the root calibration from the total evidence tip dating analyses (node calibration 7; =  *Andinodelphys*-*Mayulestes*-*Pucadelphys*-Marsupialia split) specified as uniform distributions (TipNodeCalib2). MrBayes requires that calibrated nodes are constrained to be monophyletic a priori; the contents of the calibrated nodes were therefore determined based on the results of the undated and tip dating total evidence analyses, resulting in (for example) *Ankotarinja*, *Keeuna* and *Djarthia* being excluded from crown-clade Australidelphia. The two total evidence tip dating analyses were both run for 50 million generations, with MrBayes settings otherwise the same as for the undated and node dating molecular analyses and total evidence tip-dating analyses.

### Summarising the results of the dated analyses

For the six dated analyses, Tracer v1.6 was again used to identify when stationarity and convergence between chains had been reached. The post-burn-in trees were concatenated using the perl script Burntrees.pl (available from https://github.com/nylander/Burntrees), with branch lengths transformed from substitutions per site to time units. These post-burn-in trees were then summarised as maximum clade credibility (MCC) trees using TreeAnnotator v1.8.3, with node ages calculated as median heights. As in the undated analyses, Bayesian posterior probabilities (BPPs) were used to estimate support.

### Analyses of diversification

To analyse the pattern of diversification among Dasyuromorphia, we produced Lineage Through Time (LTT) plots of the post-burn-in trees from all six dated analyses using the R package *paleotree* [121]. The post-burn-in trees were first pruned to include only modern members of Dasyuromorphia (as defined here – see “Systematics” above and Table 1). LTT plots for the pruned trees were then produced using the multiDiv command, showing the median diversity curve and 95% quantiles, and with interval length set to 0.01 MYA. We also calculated individual median diversity curves for the modern representatives of the dasyurid tribes Dasyurini, Phascogalini, Planigalini and Sminthopsini.

To investigate whether the diversification of modern dasyurids might be linked to environmental change, namely the development of more open, drier habitats driven by falling temperatures, and/or ecological replacement of thylacinids [14, 15, 28, 42, 43], we compared the LTT plots with a recent estimate of global surface temperatures over the Cenozoic [122], and with current estimates of thylacinid generic diversity (based on formally named taxa only) from the late Oligocene onwards [24, 27–31].

We also used BAMM 2.5.0 to test for shifts in the rate of diversification within Dasyuromorphia [123, 124], using the MCC trees from all six dated analyses. We first used the R package *BAMMtools* [124] to identify appropriate priors for each MCC tree, and then ran BAMM for 10 million generations, sampling every 2000 generations, using these priors. We corrected for incomplete sampling of modern dasyurids by specifying the sampling fraction of each dasyurid genus, based on current estimates of species numbers [1–9]. Because there is only a single modern representative of Thylacinidae (*Thylacinus cynocephalus*) and a single modern representative of Myrmecobiidae (*Myrmecobius fasciatus*), we carried out two BAMM analyses for each MCC tree, firstly for modern dasyuromorphians as a whole, and secondly with the tree pruned to modern dasyurids only; this was to see if inclusion or exclusion of these two monotypic lineages had a major impact on the inference of rate shifts. We then used *BAMMtools* to produce 95% credible sets of rate shift configurations for each of the 12 analyses (i.e. six MCC trees, for either modern dasyuromorphians as a whole or modern dasyurids only), assuming a 10% burn-in.

We also used *BAMMtools* to calculate speciation and extinction rates for the MCC trees from each of the six dated analyses, for the following groups: modern dasyuromorphians as a whole; non-dasyurid dasyuromorphians (i.e. Myrmecobiidae and Thylacinidae), dasyurids, and the four dasyurid tribes.

## Results

### Undated analyses

#### Morphology

**Fig. 1:**
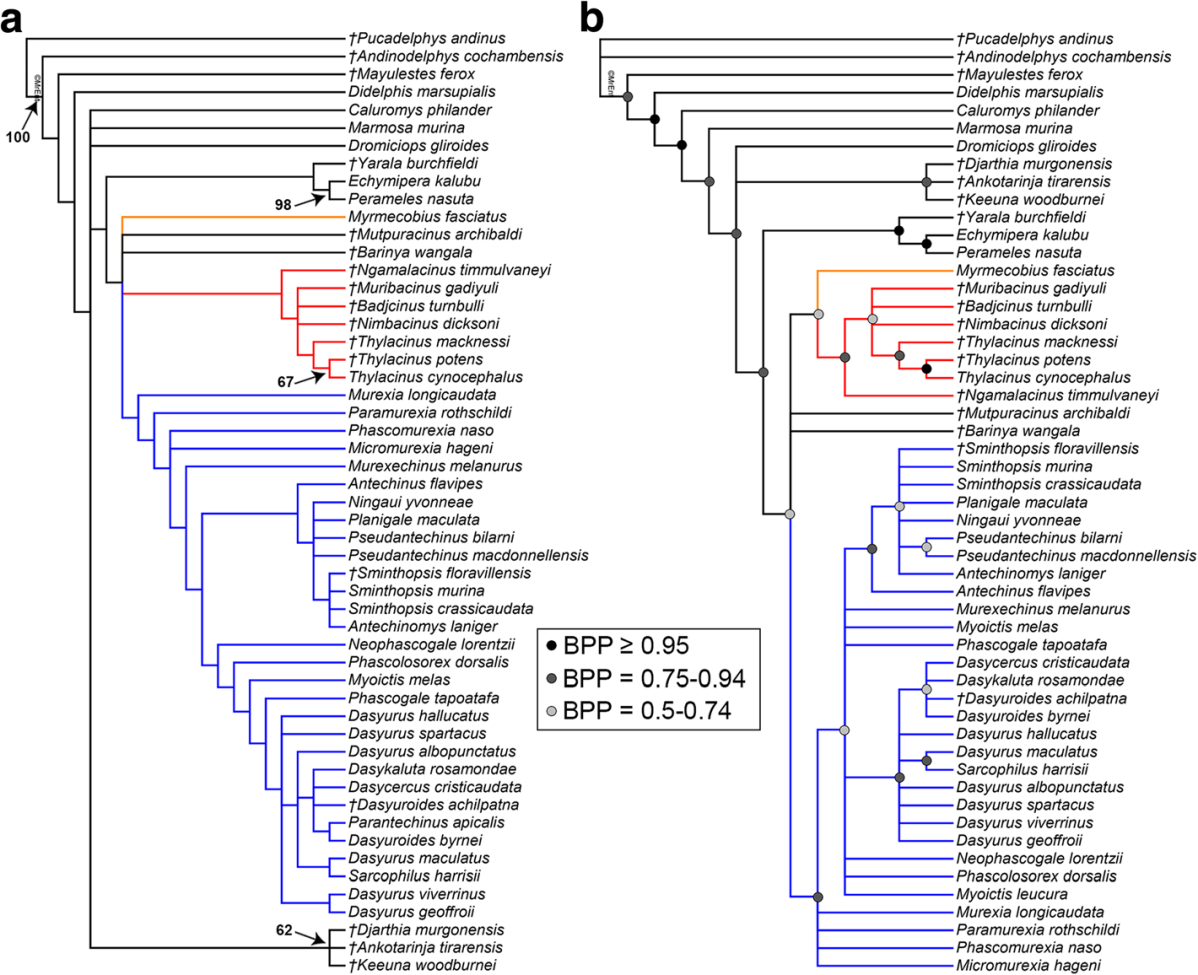
Undated phylogenies of Dasyuromorphia based on 173 morphological characters. **a** strict consensus of 3888 most parsimonious trees (length = 847 steps), with *Myoictis leucura* deleted from the starting matrix as a rogue taxon. **b** 50% majority rule consensus of post-burn-in trees from Bayesian analysis using the Mkv + G model with *Parantechinus apicalis* deleted from the matrix as a rogue taxon. In **a**, numbers at nodes represent bootstrap values ≥50%. Branch lengths are arbitrary in both **a** and **b**. Myrmecobiidae is highlighted in orange, Thylacinidae in red, and Dasyuridae in blue

Both maximum parsimony and undated Bayesian analysis of the full morphological dataset result in highly unresolved consensus trees. The Roguenarok algorithm [119] indicated that *Myoictis leucura* acted as a rogue taxon in the maximum parsimony analysis, and that *Parantechinus apicalis* did the same in the Bayesian analysis. Repeating the analyses with the relevant rogue taxon deleted resulted in the phylogenies shown in Fig. 1. Both analyses place the early or middle Miocene *Ankotarinja* and *Keeuna* in a clade with the early Eocene *Djarthia* with moderate support (bootstrap = 62%; BPP = 0.80), with this clade falling outside Dasyuromorphia. Monophyly of Dasyuromorphia is recovered, but without strong support (bootstrap <50%; BPP = 0.67). In the maximum parsimony analysis (Fig. 1a), *Barinya* (originally described as a dasyurid) and *Mutpuracinus* (originally described as a thylacinid) are placed in a polytomy with *Myrmecobius*, Dasyuridae and Thylacinidae. A similar arrangement is seen in the Bayesian analysis (Fig. 1b), except that *Myrmecobius* is weakly supported as sister to Thylacinidae (BPP = 0.57). Monophyly of Thylacinidae is relatively strongly supported in the Bayesian analysis (BPP = 0.91) but not in the maximum parsimony analysis (bootstrap <50%). Relationships within Thylacinidae are broadly similar between the two analyses, with *Ngamalacinus* sister to the remaining taxa, and *Thylacinus* spp. forming a clade, within which there is moderate-to-strong support for *T. potens + T. cynocephalus* (bootstrap = 67%; BPP = 0.96).

Monophyly of Dasyuridae is also recovered in both analyses, and receives moderate support in the Bayesian analysis (BPP = 0.80). Relationships within Dasyuridae are also similar in the two analyses: monophyly of *Murexia sensu* *lato* is not supported, and instead these taxa form a grade at the base of Dasyuridae, as also found by Van Dyck [46]. Both analyses recovered a clade comprising *Antechinus*, *Antechinomys*, *Ningaui*, *Planigale*, *Pseudantechinus* and *Sminthopsis*, which receives moderate support in the Bayesian analysis (BPP = 0.81). In the maximum parsimony analysis (Fig. 1a), the remaining taxa form a single clade, whereas the Bayesian analysis (Fig. 1b) is slightly less resolved. In the maximum parsimony analysis, *Sminthopsis* and *Antechinomys* form a clade, but *Pseudantechinus* spp. are part of a polytomy, whereas the reverse is true in the Bayesian analysis. *Dasyurus* is not monophyletic in either analysis, with *D. maculatus* instead sister to *Sarcophilus*; this relationship receives relatively strong support in the Bayesian analysis (BPP = 0.91) but not in the maximum parsimony analysis (bootstrap <50%).

Overall, the morphological results are broadly similar to those of previous morphological analyses of dasyuromorphians, which is perhaps unsurprising given that our craniodental characters and taxon set has been developed from these earlier studies [29, 36, 41, 44]. Support values are generally low in both analyses: only one clade within Dasyuromorphia (*Thylacinus potens* + *T. cynocephalus*) has >50% bootstrap support and none have >70% [125] in the maximum parsimony analysis (Fig. 1a), and only one clade (again, *Thylacinus potens* + *T. cynocephalus*) has BPP >0.95 in the Bayesian analysis (Fig. 1b).

#### Molecular

**Fig. 2:**
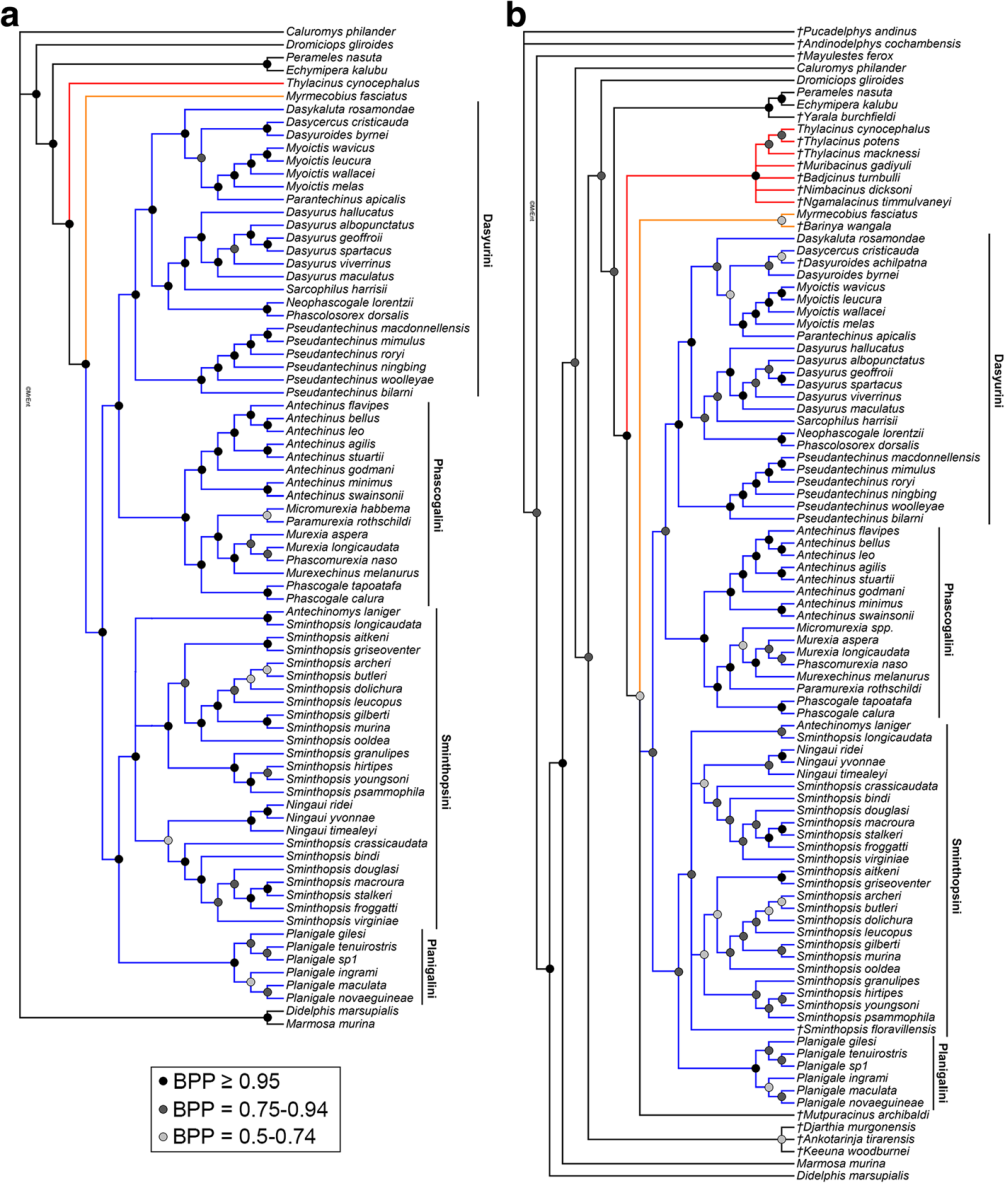
Undated Bayesian molecular and total evidence phylogenies of Dasyuromorphia. **a** 50% majority rule consensus of post-burn-in trees from Bayesian analysis of 16.4 kb of combined nuclear and mitochondrial sequence data. **b** 50% majority rule consensus of post-burn-in trees from Bayesian analysis of 16.4 kb of combined nuclear and mitochondrial sequence data and 173 craniodental and postcranial characters. Branch lengths are arbitrary in both **a** and **b**. Myrmecobiidae is highlighted in orange, Thylacinidae in red, and Dasyuridae in blue.

In contrast to the morphological analyses, the undated analysis of the combined nuclear and mitochondrial genes is characterised by high support values (BPP >0.95) for most clades (Fig. 2a). Within Dasyuromorphia, *Myrmecobius* is strongly supported as sister to Dasyuridae (BPP = 1.00), with *Thylacinus* the first taxon to diverge, in agreement with previous molecular studies [15, 20, 21, 126]. Relationships within Dasyuridae are also in agreement with most recent molecular phylogenies [8, 15, 17, 19, 20].

There is strong support for monophyly of the subfamilies Dasyurinae and Sminthopsinae, the dasyurine tribes Dasyurini and Phascogalini, and the sminthopsine tribes Sminthopsini and Planigalini (see Table 1). Within Dasyurini, *Dasyurus* is monophyletic and sister to *Sarcophilus*, with this clade sister to *Neophascogale* + *Phascolosorex*. In turn, this clade is sister to a clade comprising *Dasycercus*, *Dasykaluta, Dasyuroides*, *Myoictis* and *Parantechinus*. *Myoictis* is monophyletic, *Parantechinus* is sister to *Myoictis*, and *Dasycercus* and *Dasyuroides* form a clade. Within Phascogalini, *Antechinus*, *Phascogale* and *Murexia* sensu *lato* are all monophyletic, with *Phascogale* sister to *Murexia*. Within Sminthopsini, *Sminthopsis* is paraphyletic, with *Antechinomys* sister to *S. crassicaudata*, and *Ningaui* spp. sister to a clade corresponding to the “Macroura” group of Krajewski et al. (2012).

When the nuclear and mitochondrial genes were analysed separately, the nuclear-only phylogeny showed greater overall topological similarity to the combined analysis than did the mitochondrial-only phylogeny (see Additional file 6: Text S6). However, the nuclear-only analysis weakly supports a *Myrmecobius* + *Thylacinus* clade (BPP = 0.68). The mitochondrial-only analysis agrees with the combined analysis in supporting a *Myrmecobius* + Dasyuridae clade; however, this clade receives lower support in the mitochondrial-only analysis (BPP = 0.88) than in the combined analysis (BPP = 1.00; Fig. 2a), suggesting that the nuclear genes may be providing hidden support [47].

#### Total evidence

Similarly to the undated molecular analysis, the undated total evidence analysis placed Thylacinidae as the first family to diverge within Dasyuromorphia (Fig. 2b), but support for *Myrmecobius* + Dasyuridae is only moderate (BPP = 0.73). Monophyly of Thylacinidae is strongly supported (BPP = 0.97), although, as in the morphological analyses, *Mutpuracinus* is not recovered as a member of this clade. Instead, *Mutpuracinus* is in a polytomy with Dasyuridae and *Barinya* + *Myrmecobius*. The latter clade is intriguing and has not been found in previous published analyses, but receives only weak support (BPP = 0.60). Monophyly of Dasyuridae receives strong support (BPP = 0.92), with relationships among modern dasyurids essentially identical to those found in the undated molecular analysis; however, many support values are lower, presumably because of the destabilising effect of including fossil taxa that lack sequence data. The fossil *Dasyuroides achilpatna* is recovered as sister to *Dasycercus cristicauda*, not *Dasyuroides byrnei*, although this is relatively weakly supported (BPP = 0.64). The fossil *Sminthopsis floravillensis* is placed within Sminthopsini, but does not form a clade with any particular sminthopsin subgroup

### Dated analyses

**Fig. 3:**
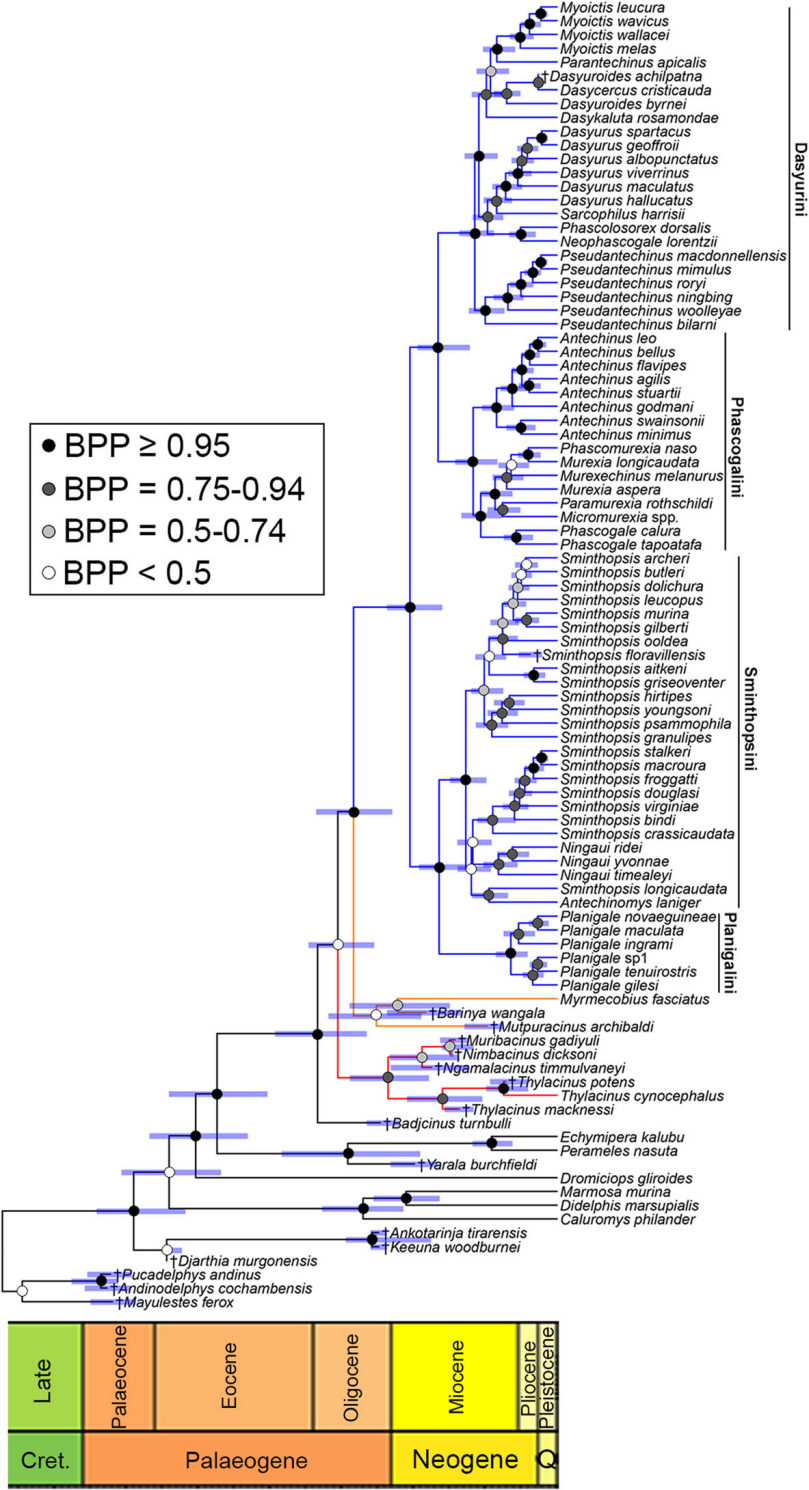
Dated total evidence phylogeny of Dasyuromorphia based on 16.4 kb of combined nuclear and mitochondrial sequence data and 173 morphological characters. Divergence dates were calculated using Bayesian tip-and-node dating, assuming a single IGR clock model and the “TipNodeCalib1” calibration scheme. The topology is a maximum clade credibility (MCC) tree of post-burn-in trees. Branch lengths are proportional to time, and bars at nodes represent 95% highest posterior densities (HPDs). Myrmecobiidae is highlighted in orange, Thylacinidae in red, and Dasyuridae in blue

Unsurprisingly, the two dated molecular analyses (NodeCalib1 and NodeCalib2) and four dated total evidence analyses (TipCalib1, TipCalib2, TipNodeCalib1 and TipNodeCalib2) recovered overall topologies that are very similar to their undated equivalents (Fig. 3 and Additional file 6: Text S6). However, *Badjcinus* is placed as a stem-dasyuromorphian rather than within Thylacinidae in both TipNodeCalib1 and TipNodeCalib2, whilst *Mutpuracinus* is sister to *Barinya* + *Myrmecobius* in TipNodeCalib1 and a stem-member of Dasyuridae in TipNodeCalib2, although these relationships are only very weakly supported (BPP <0.5: Additional file 6: Text S6).

Similarly unsurprisingly, divergence dates in the NodeCalib1 analysis (in which all fossil calibrations were specified as exponential distributions) were consistently younger than those from the NodeCalib2 analysis (in which four of the six fossil calibrations were specified as uniform distributions); this is particularly marked for the deepest divergences, (e.g. Marsupialia, crown-clade Australidelphia, Agreodontia), but divergence dates within Dasyuromorphia are also 15–25% older in NodeCalib2 than in NodeCalib1 (Table 2 and Additional file 6: Text S6). By contrast, age estimates for the two tip-dating analyses (TipCalib1 and TipCalib2) are almost identical (Table 2). It is striking that median estimates for divergence dates within Dasyuromorphia in TipCalib1 and TipCalib2 are very similar to those in NodeCalib1, but slightly younger; the 95% HPD intervals are also slightly narrower (Table 2 and Additional file 6: Text S6). This is despite the fact that no nodes within Dasyuromorphia were calibrated in the tip-dating analyses. Median estimates and 95% HPD intervals for most divergence dates are almost identical between the two tip-and-node dating analyses (TipNodeCalib1 and TipNodeCalib2), despite the difference in calibration strategy between the two.

**Table 2:** Summary of divergence dates for selected nodes from our six dating analyses and from two recent molecular studies

Analysis								
Clade	NodeCalib1	NodeCalib2	TipCalib1	TipCalib2	TipNodeCalib1	TipNodeCalib2	Mitchell et al. [20]	Westerman et al. [15]
Didelphidae-Australidelphia split	61.1 (54.6–75.2)	75.9 (61.2–83.6)	55.5 (47.9–64.3)	56.8 (50.8–64.1)	54.3 (47.9–61.8)	55.5 (49.5–62.6)	82.5	N/A
*Dromiciops*-Peramelemorphia-Dasyuromorphia split (= crown-Australidelphia)	55.5 (46.5–69.5)	68.6 (54.1–79.6)	53.6 (45.1–63.2)	N/A	50.6 (44.5–58.3)	51.5 (44.9–57.2)	69.2	N/A
Peramelemorphia-Dasyuromorphia split	52.1 (43.5–64.9)	64.4 (49.9–74.4)	51.8 (42.8–61.7)	N/A	47.6 (41.2–55.0)	48.4 (42.6–54.0)	64.3	N/A
*Thylacinus-Myrmecobius*-Dasyuridae split (= Dasyuroidea)	34.7 (28.4–44.1)	42.9 (33.4–51.2)	36.4 (30.3–44.0)	36.5 (30.2–43.1)	30.7 (26.9–36.1)	31.6 (27.7–35.5)	38.6	~40
Dasyurinae-Sminthopsinae split (= crown-Dasyuridae)	23.4 (18.8–29.4)	28.6 (22.2–34.5)	22.6 (18.7–26.7)	22.6 18.7–25.5)	20.6 (17.9–25.6)	21.1 (18.2–23.7)	24.9	29.3 (25.5–33.1)
crown-Dasyurinae	18.8 (15.2–23.6)	22.9 (17.6–27.7)	18.0 (14.8–21.7)	17.9 (15.7–20.9)	16.7 (14.1–21.5)	16.9 (14.7–19.4)	18.5	24.5 (21.2–28.0)
crown-Dasyurini	13.0 (10.4–16.5)	15.8 (12.1–19.3)	12.4 (10.2–15.0)	12.3 (10.8–14.6)	11.5 (9.6–14.5)	11.7 (9.9–13.3)	13.4	~18
*Dasyurus*	8.1 (6.1–10.5)	9.7 (7.0–12.3)	7.7 (5.9–9.7)	7.6 (6.1–9.3)	7.2 (5.4–9.6)	7.2 (5.6–8.7)	8.2	11.2 (9.0–13.4)
crown-Phascogalini	13.3 (10.6–17.0)	16.2 (12.2–19.8)	12.6 (10.0–15.4)	12.4 (10.7–14.7)	11.8 (9.5–15.4)	11.9 (10.2–13.6)	12.9	18.8 (15.7–21.9)
*Antechinus*	9.5 (7.2–12.4)	11.5 (7.9–14.7)	9.0 (6.9–11.3)	8.9 (7.2–10.8)	8.5 (6.6–11.1)	8.5 (6.6–9.9)	9.9	11.9 (9.5–14.5)
*Murexia* sensu *lato*	9.9 (7.7–13.0)	12.0 (9.0–15.0)	9.3 (7.3–11.8)	9.1 (7.8–10.7)	8.7 (6.7–11.2)	8.8 (7.1–10.5)	11.0	13.7 (11.0–16.6)
crown-Sminthopsinae	18.9 (14.9–23.8)	22.8 (17.6–27.8)	17.9 (14.6–21.6)	17.9 (14.9–20.8)	16.5 (14.1–20.8)	16.9 (14.3–19.2)	19.1	24.1 (20.5–27.7)
crown-Planigalini (= *Planigale*)	7.2 (5.2–9.7)	8.8 (5.9–11.6)	6.8 (5.1–9.0)	6.8 (5.1–8.6)	6.5 (4.4–8.9)	6.5 (4.8–8.2)	N/A	12.3 (9.4–15.5)
crown-Sminthopsini	14.7 (11.8–18.5)	17.8 (13.7–21.8)	13.8 (11.3–16.5)	13.8 (11.5–15.9)	12.8 (10.8–15.9)	13.1 (11.4–15.2)	12.2	19.7 (16.7–23.0)

NodeCalib1 and NodeCalib2 are molecular dating analyses, TipCalib1 and TipCalib2 are total evidence tip dating analyses, and TipNodeCalib1 and TipNodeCalib2 are total evidence tip-and-node dating analyses. Values in brackets represent 95% highest posterior densities (HPDs), where available

In all six analyses, the origin of crown-Dasyurinae (= Dasyurini-Phascogalini split) and the origin of crown-Sminthopsinae (=Sminthopsini-Planigalini split) are estimated as having occurred almost simultaneously, with estimates ranging from the late Oligocene or early Miocene in NodeCalib2 to the early or middle Miocene in the tip-dating and tip-and-node dating analyses (Table 2). The first splits within the tribes Dasyurini, Phascogalini and Sminthopsini are also estimated as occurring at roughly the same time, with the estimates again oldest in NodeCalib2 (early to middle Miocene) and youngest in the tip-and-node-dating analyses (middle to late Miocene). Our results also suggest that Planigalini began to diversify between 5 and 9 Ma later than the other three tribes, with the first split within *Planigale* estimated to be as old as the middle Miocene in NodeCalib2, but as young as the early Pliocene in the tip-dating and tip-and-node dating analyses (Table 2).

#### Diversification analyses

**Fig. 4:**
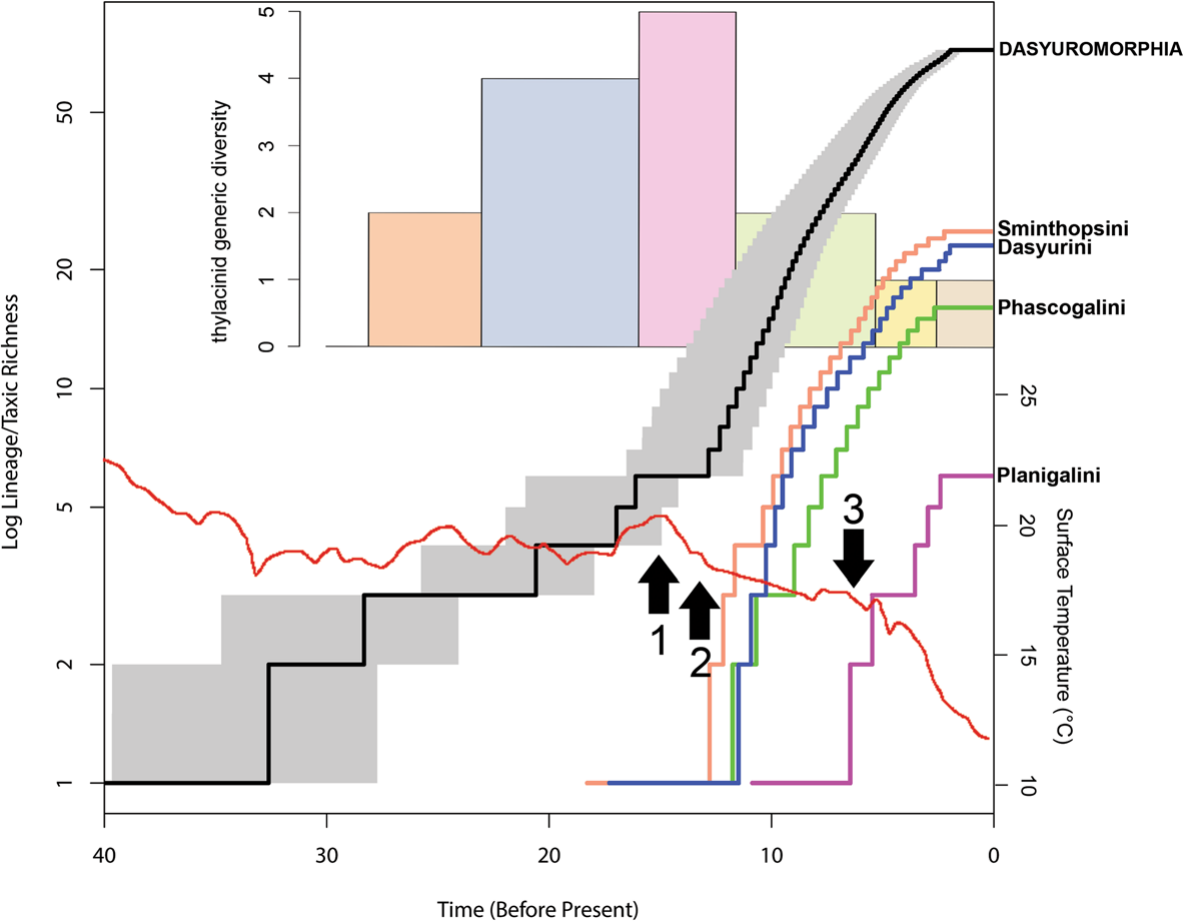
Lineage through time (LTT) plots of modern dasyuromorphians compared with global temperature and thylacinid diversity. The diversity curves were plotted based on the post-burn-in trees from the “TipNodeCalib1” total evidence tip-and-node dating analysis (see Fig. 3 and text). The black line represents the median diversity curve for modern dasyuromorphians as a whole, with the grey shading representing the 95% confidence interval (based on the post-burn-in trees). The red line represents estimated global surface temperature (taken from Hansen et al. 2013). Arrow 1 indicates the middle Miocene Climatic Optimum (MMCO), arrow 2 the middle Miocene Climatic Transition (MMCT), and arrow 3 the major increase in grass pollen seen in the palynological record of Australia (see Martin and McMinn, 1994: Fig. 2). The upper bar graph represents thylacinid generic diversity through time based on named genera (note that *Mutpuracinus* was not included, based on our results – see Figs 1, 2, 3), with five temporal bins used: late Oligocene (28.1–23.03 MYA), early Miocene (23.03–15.97 MYA), middle Miocene (15.97–11.62 MYA), late Miocene (11.62–5.333 MYA), Pliocene (5.333–2.58 MYA) and Quaternary (2.58–0 MYA)

Based on the arguments of O’Reilly and Donoghue [78], we consider that our two tip-and-node dating analyses (TipNodeCalib1 and TipNodeCalib2) are likely to have given the most accurate estimates of divergence time within Dasyuromorphia. Thus, we have focused on the results of these two analyses to investigate the pattern of diversification through time seen in modern dasyuromorphians. LTT plots of modern dasyuromorphians for TipNodeCalib1 are shown in Fig. 4, but results for TipNodeCalib2 are very similar. Median diversity curves were plotted from the post-burn-in trees, for Dasyuromorphia as a whole, and for the dasyurid subtribes Dasyurini, Phascogalini, Planigalini and Sminthopsini; the 95% confidence interval is also shown from Dasyuromorphia (Fig. 4).

There is evidence of an increase in the rate of diversification within Dasyuromorphia, centred around the late middle Miocene, which was driven by the radiation of the dasyurid tribes Dasyurini, Phascogalini and Sminthopsini (Fig. 4); the median estimate for the radiation of Sminthopsini (the first of the tribes to diversify) is 12.8 MYA in TipNodeCalib1 and 13.1 MYA in TipNodeCalib2. This is shortly after a rapid decline in global temperatures (the middle Miocene Climatic Transition) that followed the ~15–17 MYA middle Miocene Climatic Optimum (Fig. 4) [122, 127–129]. It also appears to coincide with a major drop in thylacinid generic diversity, from five named genera in the middle Miocene (*Maximucinus*, *Muribacinus*, *Nimbacinus*, *Wabulacinus* and *Thylacinus*; note that *Mutpuracinus* was not included in this total, because it was not recovered as a thylacinid in any of the phylogenetic analyses presented here – see above), to only two in the late Miocene (*Thylacinus* and *Tjarrpecinus*) (Fig. 4).

The radiation of Planigalini/*Planigale* is estimated as a much more recent event, with the median estimate being 6.5 MYA in both TipNodeCalib1 and TipNodeCalib2, i.e. latest Miocene. These dates coincide with palaeobotanical evidence for a major increase in the abundance of grasses in Australia ~6–7 MYA (Fig. 4) [130, 131].

The LTT plots for Dasyurini, Phascogalini and Sminthopsini all show a relatively constant accumulation of lineages before plateauing in the Pleistocene, with the plateau probably because we have failed to sample recently diverged cryptic species and/or multiple lineages within species. The LTT plots for Planigalini/*Planigale* are less smooth, probably due to failure to sample several major lineages [8], but possibly also because of the overall fewer number of species.

BAMM analysis was carried out on 12 trees (the MCC trees from each of the six dated analyses, with the trees either pruned to modern dasyuromorphians or to modern dasyurids only) to identify possible diversification rate shifts. BAMM analysis of modern dasyuromorphians as a whole consistently identified either zero shifts or only one shift within the 95% credible set of rate shift configurations: the highest posterior probability was for zero shifts, with a markedly lower probability of single shift occurring along the branch leading to Dasyuridae (Table 3). No shifts were identified within the 95% credible set of rate shift configurations when the analyses were repeated for modern dasyurids only (Table 3). Thus, although the LTT plots show a sharp increase in the diversification of dasyurids, related to the diversification of Dasyurini, Phascogalini and Sminthopsini, this is not interpreted as a significant change in diversification rate.

**Table 3:** Summary of BAMM analyses to test for evidence for of shifts in diversification rate within Dasyuromorphia

Analysis	Clade	Number of rate shift configurations within 95% credible set (and number of distinct shifts within each configuration)	Location of shift (and posterior probability)
NodeCalib1	Dasyuromorphia	2 (0, 1)	No shifts (PP = 0.78)
			Dasyuridae (PP = 0.19)
	Dasyuridae	1 (0)	No shifts (PP = 1.00)
NodeCalib2	Dasyuromorphia	2 (0, 1)	No shifts (PP = 0.78)
			Dasyuridae (PP = 0.18)
	Dasyuridae	1 (0)	No shifts (PP = 1.00)
TipCalib1	Dasyuromorphia	2 (0, 1)	No shifts (PP = 0.77)
			Dasyuridae (PP = 0.23)
	Dasyuridae	1 (0)	No shifts (PP = 1.00)
TipCalib2	Dasyuromorphia	2 (0, 1)	No shifts (PP = 0.85)
			Dasyuridae (PP = 0.15)
	Dasyuridae	1 (0)	No shifts (PP = 1.00)
TipNodeCalib1	Dasyuromorphia	2 (0, 1)	No shifts (PP = 0.86)
			Dasyuridae (PP = 0.12)
	Dasyuridae	1 (0)	No shifts (PP = 1.00)
TipNodeCalib2	Dasyuromorphia	2 (0, 1)	No shifts (PP = 0.86)
			Dasyuridae (PP = 0.12)
	Dasyuridae	1 (0)	No shifts (PP = 1.00)

MCC trees from each of our six dating analyses were tested, pruning the taxa to either modern dasyuromorphians only (“Dasyuromorphia”) or to modern dasyurids only (“Dasyuridae”), and correcting for the incomplete sampling of dasyurid species. BAMM analyses were run for 10 million generations, sampling every 2000 generations, with the first 10% discarded as burn-in

We also used BAMM to estimate mean rates of speciation and extinction for Dasyuromorphia as a whole, and for subgroups within the order (Additional file 7: Text S7). Relative rates are fairly consistent across the six dated analyses. Unsurprisingly, estimated speciation rates were higher in those analyses that had younger divergence dates (i.e. TipNodeCalib1 and TipNodeCalib2), but extinction rates were also higher. Collectively, these results suggest that non-dasyurid dasyuromorphians have been characterised by only slightly lower mean speciation rates but markedly (~5-6×) higher mean extinction rates than dasyurids; however, it is difficult to accurately estimate extinction rates from phylogenies of extant taxa only [132, 133]. Strikingly, mean speciation and extinction rates are almost identical across all four modern dasyurid tribes.

## Discussion

### Phylogenetic relationships

Our morphology-only analyses are relatively poorly resolved, even after deletion of unstable taxa, and support values are generally low. The morphological analyses also show several areas of conflict with the molecular and total evidence analyses presented here, as well as with other recent molecular analyses e.g. [15, 20, 21]. Monophyly of Thylacinidae (excluding *Mutpuracinus*) and of Dasyuridae (excluding *Barinya*) is recovered, but only with moderate support, and then only in the Bayesian analysis. The relationship of *Myrmecobius* to these two clades was not clearly resolved with morphological data, and relationships within Thylacinidae and Dasyuridae were also poorly resolved. Furthermore, relationships within Dasyuridae were relatively incongruent with molecular data in, for example, failing to recover monophyly of the currently recognised modern dasyurid subfamilies and tribes, as is also the case in other morphology-only analyses of Dasyuromorphia [29, 41, 46, 134].

Morphological data alone might not always be capable of fully resolving relationships within a clade, even in principle [135], which may explain, at least in part, the relatively low resolution and low support values for most clades. Areas of actual incongruence between our morphological phylogenies and our molecular and total evidence phylogenies, meanwhile, may be due to factors such as non-independence and/or saturation of morphological characters [136–140], rather than simply homoplasy, and this warrants further investigation. Like other mammalian clades, the fossil record of Dasyuromorphia is dominated by dental specimens, with several taxa known only from isolated teeth [22–25, 27, 141]. However, dental characters have been shown to perform worse than the rest of the skeleton at recovering mammalian phylogeny, as measured by their ability to recover clades that are strongly supported by molecular data [142]. Ultimately, the discovery of additional well-preserved fossil material may be required to increase congruence between morphological and molecular estimates of relationships within Dasyuridae [143].

The phylogenies that result from our combined molecular and total evidence analyses, by contrast, are highly congruent with most recent molecular studies [8, 13, 15, 19, 20, 126], and show high support values for most nodes. *Myrmecobius* was consistently recovered as the sister to Dasyuridae, with Thylacinidae branching off earlier as also found by [15, 20, 21, 126], usually with strong support. However, analysis of the nuclear genes alone weakly supported *Myrmecobius* + *Thylacinus* (Additional file 6: Text S6). Larger, “phylogenomic” datasets, or rare genomic changes that show minimal homoplasy (such as retroposon insertions) [87, 144–146], will probably be required to robustly resolve the relationship between the three modern dasyuromorphian families.

Interestingly, the molecular study of May-Collado et al. [21] shows several conflicts with our results and those of other recent molecular analyses of dasyurid relationships; for example, it failed to recover monophyly of *Pseudantechinus* or a *Dasyuroides* + *Dasycercus* clade. We suspect that this is due to May-Collado et al.’s [21] use of relatively old (pre-2000) *MT-CYB* sequences that differ markedly (in several cases, >5%) from more recent sequences from the same species (Additional file 8: Text S8); we did not use these early, possibly anomalous sequences in our analyses.

Among modern taxa, monophyly of all currently recognised genera was supported, with the exception of *Sminthopsis*, which was paraphyletic with regard to *Antechinomys* and *Ningaui* see also [15, 19–21]. The latter two genera should therefore either be reduced to subgeneric rank within *Sminthopsis*, or alternatively, additional monophyletic genera should be created within Sminthopsini. Given that our dated analyses suggest that earliest divergences among species currently classified as *Sminthopsis* are similar in age to those within Phascogalini and Dasyurini (both of which are classified into multiple genera), the second of these options is probably more appropriate. However, a new, thorough taxonomic revision of sminthopsins that builds on Archer’s [147] monograph on *Sminthopsis,* and which robustly resolves species-level relationships within the tribe as a whole, is needed; it seems likely that such a study will reveal additional cryptic species-level diversity. *Murexia sensu* *lato* (*Micromurexia, Murexia*, *Murexechinus*, *Paramurexia* and *Phascomurexia*) is monophyletic, *contra* our morphological analyses and those of Van Dyck [46], but in agreement with recent molecular studies [13, 15, 20, 21].

Turning now to our fossil taxa, the Miocene taxa *Ankotarinja* and *Keeuna* do not fall within Dasyuromorphia in any of the analyses in which their relationships were left unconstrained, but instead consistently form a clade with the early Eocene *Djarthia*. Our choice of taxa and characters was aimed at determining the membership of, and relationships within, Dasyuromorphia, and *Ankotarinja* and *Keeuna* are also both highly incomplete; thus, this result should be viewed with caution. Nevertheless, *Ankotarinja, Keeuna* and *Djarthia* share a distinctive putative synapomorphy [86] that is absent in all our other taxa: presence of a “central cusp” between the apex of the centrocrista and the stylar shelf of the upper molars,. Previous research suggests that *Djarthia* is a plesiomorphic australidelphian, based largely on tarsal evidence [59]. However, *Djarthia* does not consistently fall within Australidelphia in our analyses, probably because our dataset was not intended to resolve marsupial interordinal phylogeny. Nevertheless, while a close relationship between these three taxa is plausible, we prefer to classify *Ankotarinja* and *Keeuna* as Marsupialia *incertae sedis* following [24, 86], rather than Australidelphia *incertae sedis*. Additional, non-dental material (e.g. tarsal specimens) will probably be required to clarify their relationships.

*Barinya wangala* was originally described as the oldest known dasyurid by Wroe [60]. *Mutpuracinus archibaldi* was originally described as a thylacinid by Murray and Megirian [45], a conclusion that was maintained by these authors in a subsequent paper based on more complete material (a partial skull) [29]. However, only one of our analyses placed *Barinya* in Dasyuridae, and none placed *Mutpuracinus* in Thylacinidae. *Barinya* was consistently placed as sister to *Myrmecobius* in our total evidence analyses; this arrangement is intriguing, particularly given an otherwise total lack of a fossil record for myrmecobiids. However, it was only weakly supported, and should be viewed with caution pending the discovery of definitive fossil myrmecobiids that retain functional dentitions (the very reduced dentition of *Myrmecobius fasciatus* cannot be meaningfully compared to other dasyuromorphians for many of the dental characters used here). *Mutpuracinus* was recovered as either a stem-dasyurid or sister to *Barinya* + *Myrmecobius*, but this relationship was also weakly supported. Based on these results, we suggest that *Barinya* and *Mutpuracinus* should be considered Dasyuromorphia *incertae sedis*, pending further studies and the discovery of more complete material of both taxa.

Finally, the late Oligocene *Badjcinus* fell outside Thylacinidae in some analyses, forming the sister taxon to the rest of Dasyuromorphia; this relationship was also found by Wroe et al. [44]. However, most of our analyses place *Badjcinus* within Thylacinidae, in agreement with the original description by Muirhead and Wroe [40], the morphological analyses of Wroe and Musser [41] and Murray and Megirian [29], and the molecular scaffold analysis of Archer et al. [36]. Based on available evidence, we suggest that *Badjcinus* should be classified as ?Thylacinidae.

The relationships of the other fossil taxa were broadly as expected. Taxa currently identified as thylacinids (except *Mutpuracinus* and *Badjcinus*, discussed above) consistently formed a clade. The Plio-Pleistocene *Sminthopsis floravillensis* consistently fell within Sminthopsini as in [36], although it is unclear whether this fossil taxon is a member of any particular sminthopsin subclade. Finally, the Pliocene *Dasyuroides achilpatna* consistently fell as sister-taxon of *Dasycercus cristicauda*, rather than *Dasyuroides byrnei*, albeit with only weak support. Archer [22] only tentatively referred this fossil taxon to *Dasyuroides*, and in his original description Marshall [148] identified it as a “possible ancestral form of *Dasyuroides* or *Dasycercus* (or both)”. *Dasyuroides achilpatna* shares with *Dasycercus cristicauda* the synapomorphic presence of only a single talonid cuspid on m4, whereas two cuspids are present in *Dasyuroides byrnei*. Based on this, and on the results of our analyses, this taxon should perhaps be reassigned to *Dasycercus*.

Divergence times estimated using molecular node dating were broadly similar to the recent studies by Mitchell et al. [20] and Westerman et al. [15] (Table 2). Specifically, dates in the NodeCalib1 analysis (in which all six node calibrations were specified as exponential distributions) were closer to those of Mitchell et al. [20], who used uniform priors with hard minima and soft maxima (97.5%), whereas dates in the NodeCalib2 analysis (in which four of the six node calibrations were specified as uniform distributions) were closer to those of Westerman et al. [15], who used normal distributions (it should be noted that normal distributions are generally unsuitable for fossil calibrations) [61].

Despite this congruence, we note here that some of the fossil calibrations used by Mitchell et al. (2014) and Westerman et al. [15, 149] appear inappropriate in the light of current evidence. For example, both Mitchell et al. [20] and Westerman et al. [15] used a minimum of 4.36 MYA for the split between modern peramelemorphian subfamilies Peroryctinae and Echymiperinae, based on “cf. *Peroryctes*” *tedfordi* from the early Pliocene Hamilton Local Fauna; however, this fossil taxon has now been referred to a new genus, *Silvicultor*, and does not form a clade with *Peroryctes* in published phylogenetic analyses [150–155]. Likewise, both studies used “*Antechinus*” sp. from the Hamilton Local Fauna to date the split between *Antechinus* and *Phascogale* as >4.36 MYA. However, the Hamilton taxon was specifically considered by [22] to be most similar among modern species to “*Antechinus mayeri*” [156, 157], which is now classified as *Phascomurexia naso*, and a then-unnamed “*Antechinus*” species from Mount Wilhelm in New Guinea that Van Dyck [46] subsequently referred to *Micromurexia habbema*, i.e. two species of *Murexia* sensu *lato*. Thus, the Hamilton “*Antechinus*” is inappropriate for calibrating the *Antechinus*-*Phascogale* split; however, it may be appropriate for calibrating the split between *Murexia* and other phascogalins. Finally, Westerman et al. (2015) used a minimum of 65.18 MYA for the split between Australidelphia and Didelphimorphia on the assumption that peradectids (which are first known from the earliest Palaeocene) are didelphimorphians, based on Horovitz et al. [158]. However, subsequent studies have shown that peradectids are at best only questionably members of Marsupialia [58, 80, 159, 160], and so are not suitable for dating the Australidelphia-Didelphimorphia split. The oldest known taxon that can be confidently referred to Marsupialia is the early Eocene *Djarthia murgonensis* [58, 59, 80, 159, 160].

It is interesting to note that the divergence times we estimated using total evidence tip dating were broadly similar to the molecular node dating, even though no nodes within Dasyuromorphia were calibrated; the only temporal information was provided by two node calibrations deeper in the tree plus the ages of the tips. Divergence dates were almost identical between the NodeCalib1, TipCalib1 and TipCalib2 analyses, with the NodeCalib2 analysis slightly older (Table 2).

Finally, it is striking that, in contrast to the molecular node dating, our total evidence tip-and-node dating analyses appear to have been relatively insensitive to the way in which node calibrations were specified, i.e. whether as offset exponential or uniform distributions: the TipNode1 (in which all seven node calibrations were specified as offset exponential calibrations) and TipNode2 (in which five of the seven node calibrations were specified as uniform calibrations) analyses resulted in almost identical median estimates for all nodes (Table 2). The total evidence tip-and-node dating analyses gave the youngest dates out of our analyses, presumably because the additional temporal information provided by the tips resulted in tighter maxima being placed on the calibrated nodes [78].

It is worth emphasising that the comparatively young dates that result with tip-and-node dating are not simply due to the fact that none of our dasyuromorphian tips are older than the late Oligocene. Both tip dating and tip-and-node dating assume an underlying clock model in which the amount of change along a branch is assumed to be proportional to the length of time that branch represents [69]. Thus, the estimated divergence times for a particular node can be pushed far back in time relative to the tips descending from that node, if those tips are highly apomorphic and so represent comparatively long branches. The relatively young dates found here reflect the fact that our tips are not particularly apomorphic relative to the morphology inferred for their ancestral nodes. We agree with O’Reilly and Donoghue [78] that tip-and-node dating “makes the best use of palaeontological data in the construction of evolutionary timescales”, in particular by providing a more objective basis for defining maximum and minimum bounds on nodes [78]. We therefore consider that the divergence date estimates from our total evidence tip-and-node dating analyses are likely to be the most accurate such estimates for Dasyuromorphia currently available.

### Diversification dynamics of modern dasyuromorphians

The pattern of diversification dynamics within modern dasyuromorphians indicated by our tip-and-node dating analyses is much more congruent with the Australian fossil record and known palaeoenvironmental changes than other recent studies [15, 20]. Most obviously, we find evidence of an increase in net diversification rate in Dasyuridae starting 12.8–13.1 MYA (composite 95% HPD: 10.8–15.9 MYA), i.e. the late middle Miocene. This is due to the almost simultaneous diversification in three of the four dasyurid subfamilies, namely Dasyurini, Phascogalini and Sminthopsini (however, it should be noted that our BAMM analyses did not identify these as representing significant shifts in the rate of diversification; Table 3). The fossil record indicates a major turnover in Australian mammals at the end of the middle Miocene, with the apparent extinction of several families and the first appearance in the fossil record of several modern lineages; this turnover was likely connected with the replacement of closed, wet forest by more open, drier forest and woodland, in response to a fall in global temperatures of up to 7 °C (the middle Miocene Climatic Transition) [28, 122, 127–129, 161]. An increase in diversification in Dasyuridae at this time is congruent with such a turnover event.

By contrast, the molecular divergence dates of Westerman et al. [15] suggest that Dasyurini, Phascogalini and Sminthopsini probably began to diversify during the early Miocene (~18–19 MYA), at a time when closed, wet forest was widespread [28]. The Westerman et al. [15] dates are highly incongruent with the known fossil record, which instead indicates that during the early Miocene carnivorous-insectivorous niches in Australia were largely filled by thylacinids, peramelemorphians, and non-dasyuromorphian taxa such as *Ankotarinja* and *Keeuna* [40, 42, 60, 150, 162–165]. Dasyurids appear to have been very uncommon at this time, and no definitive members of the modern subfamilies (i.e. Dasyurinae and Sminthopsinae) have been described that are older than the Pliocene [24, 27, 60]. Black et al. [28] reported putative phascogalins and dasyurins from the early Miocene of Riversleigh, but these have yet to be described, and so their true affinities must be treated as uncertain at this stage.

As part of the end-middle Miocene turnover event, there was a major reduction in the diversity of thylacinids, from five genera in the middle Miocene (excluding *Mutpuracinus*, which we consider to be Dasyuromorphia *incertae sedis*) to only two in the late Miocene [24]. Several archaic faunivorous peramelemorphian lineages (e.g. species of *Galadi*, *Bulungu* and *Yarala*) [166] also appear to have gone extinct at this time. This is of significance because several authors have proposed that dasyurids diversified to fill the carnivorous-insectivorous niches previously occupied by thylacinids and peramelemorphians [40, 42, 60, 150, 162–165]. Ultimately, this hypothesis will need to be tested, for example by quantitative comparison of ecological metrics such as tooth shape [167] and bite force [168], to see whether or not Oligo-Miocene thylacinids and peramelemorphians did indeed fill similar dietary niches to modern dasyurids (Beck et al., in prep.), and by the discovery of postcranial material to see if they show similar locomotory adaptations. Also in need of testing is Wroe’s [42, 43] hypothesis that the ear auditory regions of dasyurids (which are relatively strongly pneumatised and largely enclosed by prominent tympanic processes) are better adapted to more open environments than those of thylacinids (which are less well pneumatised, with much smaller tympanic processes), which might explain the greater success of dasyurids following the development of drier, more open habitats from the middle Miocene onwards.

Improvements in the dating of Australian fossil sites will also be required to clarify the exact timing of the declines in diversity of thylacinids and archaic peramelemorphians. Radiometric dates are now available for a few Riversleigh sites [38], but many others lack dates, and the ages of many other Oligo-Miocene sites in Australia are poorly constrained or otherwise controversial. Without precise temporal information, it is difficult to determine whether the declines in diversity of thylacinids and archaic peramelemorphians coincided with the increase in diversification of dasyurids identified here, or whether they preceded or followed it. Distinguishing between these possibilities might help clarify whether: 1) thylacinid and archaic peramelemorphian diversity declined due to abiotic factors (e.g. the appearance of drier, more open habitats), with dasyurids diversifying later to fill the vacant niches (passive replacement); or 2) the diversifying dasyurids caused the decline in thylacinids and archaic peramelemorphians due to direct competition (active replacement); or 3) there was no link between the declines in thylacinid and archaic peramelemorphian diversity and the diversification of dasyurids.

An increase in diversification rate can be the result of an increase in speciation rate, a decrease in extinction rate, or both. Our BAMM results (Additional file 7: Text S7) suggest that modern dasyurids have been characterised by slightly higher speciation rates and much lower extinction rates than modern non-dasyurid dasyuromorphians (i.e. the lineages leading to *Myrmecobius* and *Thylacinus*). However, estimating extinction rates from phylogenies of modern species only is fraught with difficulty [133, 169–172], and so we view these results with caution.

To fully understand the diversification dynamics of dasyuromorphians, additional fossils from sites around Australia e.g. [26, 32, 173], will need to be incorporated within the broad phylogenetic context established here and in other studies e.g. [15, 20]. However, given the weakly supported relationships found in our morphology-only analyses, it may be difficult to robustly resolve their affinities, particularly those known only from dental specimens (some, e.g. *Maximucinus muirheadae*, are known from a single tooth). Thus, the use of methods for inferring diversification dynamics that do not require a phylogeny should also be investigated [174–176].

All of our dated analyses indicate that Planigalini (represented by the genus *Planigale*) began diversifying ~5–9 Ma later than the other three tribes; our tip-and-node dating analyses place this event (6.5 MYA; composite 95% HPD: 4.4–8.9 MYA), i.e. the latest Miocene to earliest Pliocene. Westerman et al.’s [15] point estimate for this event, 12.3 MYA, is nearly twice as old as ours, and, like their other dates, is strongly incongruent with the fossil record; the oldest known specimen of *Planigale* is from the Bluff Downs Local Fauna [22], which is between 3.6 and 5.2 Ma old [177]. Interestingly, our median estimate for the diversification of Planigalini roughly coincides with a major increase in the abundance of grass pollen (from 1 to 2% to ~35% of the total pollen count) in a deep sea core taken off the coast of northwestern Australia [130]: Figure 2, [131]. Modern planigale species are typically found in woodland with a grassy understory, shrublands and grasslands, particularly in association with cracking soils [178–181]. The increase in grass pollen observed by Martin and McMinn [130] in the latest Miocene may mark the development of these types of habitat in Australia, which in turn may have driven planigale diversification. The spread of grasses in Australia has been proposed to be causally linked to events in the evolution of several other Australian mammals, including the diversification of macropodin kangaroos and wallabies [182], and the loss of visual function in the lineage leading to modern marsupial moles (*Notoryctes* spp.) [183].

## Conclusion

Although most relationships recovered by our morphology-only analyses of dasyuromorphian phylogeny are only weakly supported, our total evidence analyses result in a relatively well-supported phylogeny that is highly congruent with previous studies. The temporal information provided by our fossil taxa also has a major impact on estimated divergence times, with the strong congruence between our two tip-and-node dating analyses (despite major differences in the representation of node calibrations between the two analyses) particularly striking. Tip-and-node dating has been argued to result in divergence times that are “better justified, more precise and accurate” than either node-dating or tip-dating alone [78]; we concur, and we suggest that the divergence times from our two tip-and-node dating analyses are likely to be the most accurate such estimates currently available for Dasyuromorphia. They indicate a pattern of diversification among modern dasyurids that is highly congruent with the known fossil record, and which can be linked to palaeoenvironmental factors that have previously been considered to have had a profound effect on mammal evolution in Australia [28]. Among marsupials, peramelemorphians and macropodoids also exhibit considerable modern and fossil diversity and both have large molecular [149, 184, 185] and morphological [152, 154, 182, 186, 187] datasets already available; as such, they are obvious candidates for this kind of analysis, which should reveal whether they show similar patterns of diversification to dasyuromorphians.

## Additional files

Additional file 1: Table S1. Table of taxa, indicating whether or not they have been scored for morphological characters, percentage completeness (if scored for morphological characters) and Genbank accession numbers for molecular sequences. (XLSX 18 kb)

Additional file 2: Text S2. List of morphological characters and specimens examined to score these. (DOCX 35 kb)

Additional file 3: Text S3. All data matrices used in the phylogenetic analyses presented here. (NEX 11855 kb)

Additional file 4: Text S4. List of fossil calibrations and justifications for these. (DOCX 43 kb)

Additional file 5: Text S5. Age ranges of fossil taxa and justifications for these. (DOCX 20 kb)

Additional file 6: Text S6. Tree topologies from all undated and dated analyses as figures and in Nexus format. (DOCX 2389 kb)

Additional file 7: Text S7. Summary of mean speciation and extinction rates estimated for modern dasyuromorphians using BAMM. (DOCX 13 kb)

Additional file 8: Text S8. Potentially problematic MT-CYB sequences used by May-Collado et al... [21]. (DOCX 13 kb)

## Abbreviations

BAMM: Bayesian Analysis of Macroevolutionary Mixtures
BPP: Bayesian posterior probability
FBD: Fossilised birth-death
g: Grammes
HPD: Highest posterior density
IGR: Independent gamma rates
Kg: kilogrammes
LTT: Lineage through time
Ma: Megannum (= million years)
MCC: Maximum clade credibility
MYA: Million years ago
