# Supplementary material for: Total evidence phylogeny and evolutionary timescale for Australian faunivorous marsupials (Dasyuromorphia)
Source: BMC Evol Biol. 2017 Dec 4;17:240. doi: 10.1186/s12862-017-1090-0 (PMC5715987; doi:10.1186/s12862-017-1090-0)
Supplement: Supplementary file 2 — List of morphological characters and specimens examined to score these. (DOCX 35 kb) [file 12862_2017_1090_MOESM2_ESM.docx]

**Text S2. Morphological Characters**

Craniodental:

**1.** **Upper incisor number:**

0. 5

1. 4

**2.** **Morphology of C1:**

0. Caniniform

1. premolariform

**3.** **C1 alveolus:**

0. between premaxilla and maxilla

1. entirely within maxilla

**4.** **DP3 morphology:**

0. molariform, with well-developed stylar cusps and protocone present

1. distinct paracone and metacone identifiable, but stylar shelf and protocone absent/weakly developed

2. vestigial; no distinct cusps identifiable

**5.** **Height of P3 (ordered):**

0. Higher crowned than P2

1. subequal in height

2. lower crowned than P2

3. absent

**6.** **Shape of P3 in occlusal view:**

0. Laterally compressed - much longer than wide

1. bulbous and ovate - not markedly longer than wide

**7.** **Presence or absence of anterior and posterior crests on P3 (ordered):**

0. both anterior and posterior crests present and well-developed

1. only posterior crest well-developed

2. anterior and posterior crests absent or weakly-developed

**8.** **Posterolingual cuspule present or absent on P3:**

0. Absent

1. present

**9.** **Relative timing of eruption of P3 (ordered):**

0. after M4

1. simultaneous with M4

2. before M4 but after M3

3. simultaneous with M3

4. before M3

**10.** **Relative size of paracone and metacone M2-3:**

0. Paracone and metacone equal or almost equal in size to metacone

1. metacone larger than paracone

**11.** **Shape and orientation of the centrocrista (unordered):**

0. centrocrista straight, apex terminating close to level of talon basin

1. centrocrista weakly v-shaped

2. centrocrista strongly v-shaped

3. centrocrista incomplete, breaching the ectoloph

4. centrocrista straight, with apex well above talon basin

**12.** **Preparacrista on M1:**

0. present

1. absent

**13.** **Orientation of preparacrista on M1:**

0. M1 preparacrista oriented anterobuccally or buccally relative to long axis of the tooth

1. M1 preparacrista oriented posterobuccally relative to long axis of tooth

**14.** **Relative lengths of M3 and M4 preparacristae:**

0. M4 preparacrista much shorter than that of M3

1. M4 preparacrista longer or similar in length to that of M3

**15.** **Stylar cusps:**

0. identifiable as discrete structures

1. indistinct

**16.** **Relative size of stylar cusp B and stylar cusp D on M2-3:**

0. stylar cusp D much larger than stylar cusp B

1. stylar cusp D smaller or subequal to stylar cusp B

**17.** **'Central cusp':**

0. absent

1. present

**18.** **Lower incisor number:**

0. 4

1. 3

**19.** **I3 bilobed or not bilobed:**

0. Not bilobed

1. bilobed

**20.** **I2 morphology:**

0. Not staggered

1. staggered

**21.** **Height of p3 relative to p2 (ordered):**

0. p3 higher crowned than p2

1. subequal in height

2. smaller than p2

3. absent

**22.** **Presence or absence of hypoconulid notch:**

0. Present

1. absent

**23.** **Entoconid size:**

0. Large

1. reduced or absent

**24.** **Size of metaconid on m1:**

0. Metaconid of m1 not reduced

1. Metaconid of m1 reduced

**25.** **Size of metaconid in m2-4 (ordered):**

0. Large

1. reduced

2. absent

**26.** **Size of paraconid in m1 (ordered):**

0. Large

1. reduced, but still identifiable as a distinct cusp

2. absent

**27.** **Posterior cingulid in m1-3:**

0. present

1. absent

**28.** **Anterior point of termination of the cristid obliqua in m3 with respect to carnassial notch formed by postprotocristid and metacristid:**

0. lingual to carnassial notch

1. Beneath or buccal to carnassial notch

**29.** **Relative size of m4 to that of m3:**

0. m4 smaller than m3

1. m4 larger than or equal to m3

**30.** **Number of distinct cusps on m4 talonid:**

0. Three cusps

1. two cusps

2. one cusp

**31.** **Presence or absence of orbital crest:**

0. Absent

1. present

**32.** **Presence or absence of maxillopalatine fenestrae:**

0. absent

1. present

**33.** **Presence or absence of palatine fenestrae:**

0. absent

1. present

**34.** **Presence or absence of anterior palatal fenestrae:**

0. absent

1. present

**35.** **Presence or absence of complete posterolateral palatine foramen:**

0. Present

1. absent

**36.** **Presence or absence of accessory posterolateral palatine foramen:**

0. Present

1. absent

**37.** **Contribution of alisphenoid and periotic to primary foramen ovale:**

0. Delimited by alisphenoid anteriorly and periotic part of the petrosal posteriorly

1. delimited by alisphenoid only

**38.** **Presence or absence of secondary foramen ovale:**

0. absent or incomplete

1. present

**39.** **Complete stylomastoid foramen:**

0. Absent

1. present

**40.** **Squamosal epitympanic sinus:**

0. Absent

1. present

**41.** **Bones contributing to hypotympanic sinus roof:**

0. squamosal does not contribute

1. squamosal contributes

**42.** **Size of alisphenoid tympanic process (ordered):**

0. Absent

1. does not contact rostral tympanic process of petrosal

2. contacts rostral tympanic process of petrosal

**43.** **Length of the internal jugular canal:**

0. Does not extend anteriorly to the basisphenoid

1. extends to basisphenoid

**44.** **Presence or absence of a well developed posteroventral lip formed by a mesially directed process in the pars petrosa, enclosing the internal jugular ventrally:**

0. Absent

1. present

**45.** **Presence or absence of transverse canal:**

0. Absent

1. present

**46.** **Frontal-squamosal or alisphenoid-parietal contact on the lateral wall of the braincase:**

0. alisphenoid-parietal contact.

1. Frontal-squamosal contact.

**47.** **Morphology of the rostral tympanic process of the petrosal (ordered):**

0. Absent

1. present but small, not enclosing a sinus

2. present and large, enclosing a distinct sinus

**48.** **Paroccipital process:**

0. Absent

1. Present

**49.** **Paroccipital process:**

0. not strongly pneumatised

1. strongly pneumatised

**50.** **Shape of nasals:**

0. Nasals posteriorly expanded

1. not posteriorly expanded

**51.** **Maxilla-nasal contact:**

0. Maxilla-nasal contact longer than premaxilla-nasal contact

1. premaxilla contact longer than maxilla-nasal contact

**52.** **Posterior extension of nasals:**

0. Nasals extend posteriorly beyond the anterior rim of the orbit

1. do not extend posteriorly beyond the orbit

**53.** **Frontal-maxillary contact:**

0. Present

1. absent

**54.** **Morphology of jugal:**

0. Jugal not Y-shaped

1. jugal Y-shaped

**55.** **Antorbital fossa:**

0. absent

1. present

**56.** **Presence or absence of prootic canal:**

0. Present

1. absent

**57.** **Interparietal:**

0. Present

1. absent

**58.** **Interparietal fusion:**

0. unfused to supraoccipital

1. fused to supraoccipital

Postcranial:

**59.** **Atlantal foramen or notch (ordered):**

*from Flores (2009) #1 and O'Leary et al. (2013) Morphobank #2739*

0. absent

1. alar notch

2. complete foramen

**60.** **Atlas transverse foramen (ordered):**

*from Flores (2009) #2*

0. absent

1. incomplete

2. present

**61.** **Atlas, posterior extention of the transverse processes:**

*from Flores (2009) #3*

0. does not extend beyond caudal facets for axis articulation

1. extends beyond caudal facets

**62.** **Atlas, cranial articular facets shape:**

*from Flores (2009) #4*

0. only concave

1. dorsal edge curved

**63.** **Atlas ventral arches:**

*from Horovitz & Sanchez-Villagra (2003) #7*

0. not fused

1. fused

**64.** **Atlas, suture line(s) at fusion of ventral arches:**

0. absent

1. one suture line

2. two suture lines

**65.** **Atlas, development of ventral tubercle** **(ordered):**

*from Flores (2003) #5*

0. absent

1. protuberance

2. tubercle well developed

**66.** **Atlas, transverse process craniocaudal length with respect to the dorsal hemal arches craniocaudal length** **(ordered):**

*from Flores (2003) #8*

0. longer

1. subequal

2. shorter

**67.** **Atlas, neural process/protuberance:**

0. absent

1. present

**68.** **Axis, posterior extention of spinous (neural) process:**

*from Flores (2003) #10*

0. does not extend beyond neural processes

1. extends beyond neural arches

**69.** **Axis, spinous process, dorsal profile in lateral view:**

*from O'Leary et al. (2013) Morphobank #2758*

0. convex

1. flat

2. concave

*at least in part*

**70.** **Axis, dens cranial extention relative to the cranial tip of the spinous process:**

*from Flores (2003) #11*

0. same level or barely anterior

1. notably anterior

**71.** **Axis caudal articular surface fovea shape:**

*from Flores (2003) #12*

0. round

1. oval

**72.** **Axis, inferior portion of neural arches shape in lateral view:**

*from Flores (2003) #13*

0. narrow

1. craniocaudally wide

**73.** **Axis, ventral tubercle shape:**

*from Flores (2003) #14*

0. uniform crest

1. two separate lobes

2. without lobes or crest

**74.** **Axis, transverse foramen:**

0. absent or incomplete

1. complete foramen

**75.** **Axis, bone(s) enclosing transverse foramen:**

*edited from Horovitz & Sanchez-Villagra (2003) #9*

0. enclosed by cervical rib and axis transverse process

1. enclosed by axis transverse process only

**76.** **Axis, suture between rib and axis:**

*from Horovitz & Sanchez-Villagra (2003) #13*

0. not visible

1. clearly visible

**77.** **Axis, postzygapophysis (caudal articular fovea) orientation:**

0. dorsoventrally, or almost dorsoventarlly orientated

1. antero-posteriorly orientated

**78.** **C3 transverse process:**

*from Flores (2003) #21*

0. one head

1. two heads

**79.** **C4 and C5 transverse processes:**

*from Flores (2003) #22*

0. one head

1. two heads

2. C5 has three heads

**80.** **C5 transverse process heads overlap transversally:**

*from Horovitz & Sanchez-Villagra (2003) #17*

0. absent

1. present

**81.** **Articulation between C4 and C5 bodies shape:**

*from Horovitz & Sanchez-Villagra (2003) #23*

0. flat

1. saddle

**82.** **C6 spinous process shape** **(ordered):**

*from Flores (2003) #17*

0. absent

1. protuberance

2. lamina

**83.** **C3 - C6 spinous process size with respect to the spinous process of the axis:**

*from Flores (2003) #19*

0. smaller

1. subequal

**84.** **Relative size between C 6 and C7 spinous processes:**

*from Flores (2003) #20*

0. C7 taller

1. C6 similar size or greater than C7

**85.** **C7 transverse foramen** **(ordered):**

*from Flores (2003) #18*

0. absent

1. present, notch

2. present, complete foramen

**86.** **C7 transverse process direction:**

*from Flores (2003) #23*

0. lateral

1. ventrolateral

**87.** **C5 and T1 body length:**

*from Flores (2003) #16*

0. subequal or C5 longer than T1

1. C5 anteroposterorly shorter than T1

**88.** **First thoracic vertebra with a tall spinous process relative to other vertebrae:**

*from Flores (2003) #24*

0. T1

1. T2

**89.** **First thoracic vertebra with prezygopophysis facing latterally:**

*from Flores (2003) #25*

0. T2

1. T3

**90.** **Position of the first thoracic vertebra with a low and craniocaudally expanded spinous process** **(ordered):**

*from Flores (2003) #27*

0. up to and including T10

1. T11

2. T12

3. T13

**91.** **Position of the diaphragnatic vertebrae (pre- and post-zygopophysis vertical)** **(ordered):**

*from Flores (2003) #28*

0. up to and including T9

1. T10

2. T11

3. T12

**92.** **Position of the first vertebrae where the accessory process is differentiated from the transverse process** **(ordered):**

*from Flores (2003) #30*

0. T6

1. T7

2. T8

3. T9

**93.** **Caudal extention of the postzygapophysis of T2 - T8:**

*from Flores (2003) #31*

0. absent

1. present

**94.** **Lumbar vertebrae, intervertebral space (dorsal view):**

*from Flores (2003) #36*

0. clearly evident

1. reduced or absent

**95.** **Extension of mammillary process in L3:**

*from Flores (2003) #34*

0. not beyond articulation with L2

1. extended beyond articulation with L2

**96.** **L3, midline ventral ridge:**

*from O'Leary et al. (2013) Morphobank #2861*

0. absent

1. present

**97.** **L4, transverse process length:**

*from O'Leary et al. (2013) Morphobank #2852*

0. longer or subequal to width of centrum

1. shorter than width of centrum

**98.** **L4, spinous process length vs height** **(ordered):**

*from O'Leary et al. (2013) Morphobank #2858*

0. height greater than anteroposterior length

1. height and length roughly equivalent

2. anteroposterior length greater than height

**99.** **Orientation L3 - L4 spinous processes** **(ordered):**

*from O'Leary et al. (2013) Morphobank #2859*

0. anteriorly orientated

1. dorsoventrally (vertically) orientated

2. posteriorly orientated

**100.** **Ventral extension of L4 - L6 transverse processes:**

*from Flores (2003) #37*

0. not extended beyond vertebral body

1. extend beyond vertebral body

**101.** **Number of vertebrae in contact with the ilium:**

*from Flores (2003) #38*

0. one

1. two

**102.** **S1 spinous process, presence** **(ordered):**

0. absent

1. small proturberance

2. well developed, laminar

**103.** **Spinous process size on S1 - S2** **(ordered):**

*from Flores (2003) #41*

0. S1 and S2 spinous processes similar size

1. S1 taller than S2

2. S2 spinous process absent

**104.** **Fusion of transverse processes on S1 - S2:**

*from O'Leary et al. (2013) Morphobank #2884*

0. present

1. absent

**105.** **Fusion of transverse processes on S2 - Ca1:**

0. absent

1. present

**106.** **Presence of proturberance or spinous processes on first 3 caudal vertebrae** **(ordered):**

*from Flores (2003) #44*

0. absent

1. only on Ca1

2. processes on Ca1 - Ca2

3. present on Ca1 - Ca3

**107.** **Position of the first caudal vertebra with articulation only through vertebral body** **(ordered):**

*from Flores (2003) #48*

0. up to and including Ca5-6

1. Ca6-7

2. Ca7-8

**108.** **Ca3 transverse process heads:**

0. two or square

1. one

**109.** **First rib, torsion of shaft** **(ordered):**

0. absent

1. slightly twisted

2. base edge twisted perpendicular to neck edge

**110.** **First rib, neck length subequal to length of shaft:**

0. absent

1. present

**111.** **First rib, neck curvature:**

0. absent, neck roughly straight

1. present, noticably curving upwards

**112.** **First rib, noticeable Angle on distal edge of shaft:**

0. absent

1. present

**113.** **Development of keel in the manubrium:**

*from Flores (2003) #52*

0. weakly developed

1. well developed

**114.** **Maximum length of manubrium in relation to maximum width** **(ordered):**

*from O'Leary et al. (2013) Morphobank #2918*

0. long, narrow manubrium

1. subequal

2. short, wide manubrium

**115.** **Costal tubercles of manubrium, location** **(ordered):**

0. notiably in anterior portion

1. approximatly midlength

2. located in posterior portion

**116.** **Manubrium, cephalic extremity level in relation to costal tubercles:**

0. lower or equal to tubercles

1. cephalic extremity raised

**117.** **Scapula, coracoid process:**

0. absent/small pedicle

1. present

**118.** **Scapula, coracoid process shape:**

*from Flores (2003) #53*

0. process extends both beyond and below glenoid

1. process extends below but not beyond glenoid

2. process extends beyond but not below glenoid

**119.** **Scapular spine width at neck level:**

*from Flores (2003) #55*

0. subequal to infraspinous fossa

1. wider than infraspinous fossa

**120.** **Scapular posterior border raised** **(ordered):**

0. absent

1. slightly raised

2. notably raised, almost reaching height of spine

**121.** **Scapula, infraspinous vs supraspinous fossa width at neck level** **(ordered):**

*from Flores (2003) #56*

0. infraspinous fossa narrower

1. subequal

2. supraspinous fossa narrower

**122.** **Scapula, caudal angle:**

*from Flores (2003) #57*

0. acute

1. rounded

**123.** **Scapular notch extension:**

*from Flores (2003) #58*

0. less than half of scapula

1. half or further extension of scapula

**124.** **Scapula, ventral extension of acromion:**

*from Horovitz & Sanchez-Villagra (2003) #37*

0. extends beyond glenoid

1. does not extend beyond level of glenoid

**125.** **Scapula, metacromion process** **(ordered):**

*partially from O'Leary et al. (2013) Morphobank #2958*

0. absent

1. indistinguishable from spine and acromion extension

2. present

**126.** **Scapula, extension of metacromion process:**

0. doen not extend beyond level of posterior border

1. extends beyond posterior border

**127.** **Crest on medial aspect of scapula, near the posterior border:**

*from Horovitz & Sanchez (2003) #38*

0. absent

1. present

**128.** **Scapula, infraglenoid fossa** **(ordered):**

0. absent

1. present

2. present and deep

**129.** **Humerus, capitulum shape:**

*from Flores (2003) #60*

0. spherical

1. cylindrical

**130.** **Humerus, olecranon fossa or foramen** **(ordered):**

*from Horovitz & Sanchez-Villagra (2003) #47*

0. absent

1. small fossa

2. large fossa

3. foramen

**131.** **Humerus, supracondyloid (entepicondylar) foramen:**

*modified from O'Leary et. al. (2013) Morphobank #3041*

0. present

1. absent

**132.** **Humerus, extension of the deltopectoral crest:**

*from Flores (2003) #62*

0. restricted to the proximal half

1. reaching distal half

**133.** **Humerus, deltopectoral crest notably developed:**

*from Flores (2003) #68*

0. absent

1. present

**134.** **Humerus, distal extension of capitulum and trochlea:**

*from Flores (2003) #63*

0. trochlea longer or equal extension

1. longer proximal extension of capitulum

**135.** **Humerus, capitulum lateral extension:**

*from Flores (2003) #64*

0. absent

1. present

**136.** **Humerus, medial epicondyle expansion (ordered):**

*from Flores (2003) #69*

0. absent

1. barely expanded, about the same width as trochlea

2. notably expanded, wider than trochlea

**137.** **Humerus, degree of flare for supinator crest:**

*from O'Leary et al. (2013) Morphobank #3033*

0. small, does not extend to a significant degree

1. well developed, extends proximally and laterally.

**138.** **Humerus, proximal process on the supinator ridge:**

*from Flores (2003) #67*

0. absent

1. present

**139.** **Ulna, shape of the proximal posterior border:**

*from Flores (2003) #71*

0. curved

1. straight

**140.** **Ulna, extension of the fossa for the exterior ligament** **(ordered):**

*from Flores (2003) #74*

0. restricted to olecraneon

1. extended to trochlear notch

2. extends beyond trochlear notch

**141.** **Ulna, mesial extension of the greater sigmoid cavity:**

*from Flores (2003) #75*

0. not extended

1. notably extended, beyond level of the anconeal process.

**142.** **Ulna, anconeal process development lateral side:**

0. present and well developed

1. adheared to side of olcraneon or absent

**143.** **Ulna, anconeal process development:**

*from Flores (2003) #76*

0. adheared to side of olecraneon

1. well developed and projecting

**144.** **Ulna, olecraneon process, length relative to semilunar notch (trochlear+sigmoid) length** **(ordered):**

*from O'Leary et al. (2013) Morphobank #3076*

0. shorther than semilunar notch

1. subequal

2. olecraneon longer than notch

**145.** **Distal ulna width relative to distal radius:**

*from O'Leary et al. (2013) Morphobank #3103*

0. ulna much narrower than distal radius

1. ulna broad, subequal to distal width of radius

**146.** **Radius, lateral compression:**

*from Flores (2003) #79*

0. little or no lateral compression

1. notably compressed laterally

**147.** **Radius, anterio-posterior compression:**

0. absent

1. present

**148.** **Radius, development of the bicipital tuberosity:**

*from Flores (2003) #81*

0. scarcely marked

1. well developed

**149.** **Pelvis, iliac wing forming a large blade:**

*from Flores (2003) #84*

0. absent

1. present

**150.** **Pelvis, angle formed by the two rami of the ischium in posterior view** **(ordered):**

*from Flores (2003) #85 and Argot (2002)*

0. sharp angle, less than 90 degrees

1. right angle, equal to 90 degrees

2. oblique angle, greater than 90 degrees

**151.** **Pelvis, caudal portion of ischium body curved laterally (other than ischiatic turberosity):**

*from Flores (2003) #92*

0. absent, almost straight

1. present, curved

**152.** **Femur, lesser trochanter size** **(ordered):**

*from O'Leary et al. (2013) Morphobank #3352*

0. small tubercle, not extensive medially

1. large, forms extended flange

2. very large, flange extended medially beyond the level of the head

**153.** **Relative height of greater trochanter to femoral head:**

*from Horovitz & Sanchez-Villagra #79*

0. greater trochanter lower or equal

1. trochanter higher

**154.** **Femur, trochanteric fossa, depth:**

*from O'Leary et al. (2013) Morphobank #3344*

0. deep

1. shallow

**155.** **Femur, trochanteric fossa, length:**

0. short

1. long, extending to or beyond base of lesser trochanter

**156.** **Tibia length relative to femur length** **(ordered):**

*from Flores (2003) #98*

0. tibia shorter

1. tibia subequal to femur

2. tibia noticably longer

**157.** **Tibia, cnemial crest development:**

*from O'Leary et al. (2013) Morphobank #3394*

0. sharp, raised

1. weak, rounded

**158.** **Tibia, groove or fossa on posterior aspect of proximal shaft:**

*from O'Leary et al. (2013) Morphobank #3397*

0. absent

1. present

**159.** **Tibia, groove or fossa on posterior aspect, shape:**

*from O'Leary et al. (2013) Morphobank #3398*

0. shallow groove or fossa

1. deep, elongate fossa

**160.** **Tibia, groove or fossa on lateral aspect of proximal shaft:**

0. absent

1. present and shallower than fossa on posterior aspect

2. present and deeper or subequal to fossa on posterior aspect

**161.** **Head of fibula, shape:**

*partially from Flores (2003) #102*

0. extention of fibular body

1. noticably fan shaped

**162.** **Comparitive length of fibula and tibia:**

*relates to the articulation of the fibula to tarsus and/or femur.*

0. fibula longer or equal to length of tibia

1. fibula shorter than tibia

**163.** **Astragalus, ridge between nedial and lateral astragalotibial facets:**

*?from Flores (2003) #108?*

0. absent

1. present

**164.** **Astragalonavicular facet connection with sustentacular facet:**

*from Horovitz & Sanchez-Villagra (2003) #110*

0. absent

1. present

**165.** **Astragalar foramen:**

0. absent

1. present

**166.** **Lateral astragalotibial, shape:**

0. concave

1. not concave

**167.** **Calcaneal anterior peroneal tubercle shape:**

*from Horovitz & Sanchez-Villagra (2003) #116*

0. small protuberance

1. large, approximatly half or more the width of the calcaneus

**168.** **Calcaneal anterior peroneal tubercle position:**

*from Horovitz & Sanchez-Villagra (2003) #117*

0. protruding anteriorly beyond calcaneocuboid facet

1. anterior, nonprotruding

2. at a distance from anterior end of calcaneum

**169.** **Calcaneocuboid facet of the calcaneus** **(ordered):**

0. not subdivided into separate facets

1. subdivided into distal (CaCud) and proximal (CaCup) facets

2. subdivided into auxiliary (CaCua), lateral (CaCul) and medial facets

**170.** **Calcaneal sustentacular facet and posterior calcaneoastragalar facets merging** **(ordered):**

*from Horovitz & Sanchez-Villagra (2003) #123*

0. separate

1. narrow connection

2. merged

**171.** **Calcaneal facet for fibula:**

*from Horovitz & Sanchez-Villagra (2003) #125*

0. absent

1. present

**172.** **Cuboid medial plantar process forms groove:**

*from Horovitz & Sanchez-Villagra (2003) #130*

0. absent

1. present

**173.** **Additional distal calcaneoastragalar facet, anterior to sustentacular facet:**

0. absent

1. present

**Specimens examined**

**Abbreviations:** ANWC – Australian National Wildlife Collection; AR – Archer collection at the University of New South Wales; AM – Australian Museum; AMNH – American Museum of Natural History; NTM - Museum and Art Gallery of the Northern Territory; SAM – South Australian Museum; QM – Queensland Museum; UCR – University of California, Riverside.

*Didelphis marsupialis*: AMNH M-266468; AR21585

*Caluromys philander*: AMNH M-267336

*Marmosa murina*: AMNH M-99983

*Dromiciops gliroides*: AM M-17826; AR21584

†*Ankotarinja tirarensis*: QM F7331 (cast)

†*Keeuna woodburnei*: QM F7334 (cast); SAM P18191 (cast); UCR 15324 (cast)

*Echymipera kalubu:* AM M24591, M24590

*Perameles nasuta*: AM M43649, M44868, M39047

*Myrmecobius fasciatus*: AM M42678, M42670

*Sminthopsis murina*: AM M24662, M13589; AR1571

*Sminthopsis crassicaudata*: AM M6859, M38549

*Planigale maculata*: AM M10744; AR702

*Ningaui yvonneae*: AM M33765, M23502, M12159

*Murexia longicaudata*: AM M18158, M18157

*Murexechinus melanurus*: AM M17657, M15589, M15594, M15578, M15588; AMNH M-221628

*Paramurexia rothschildi*: ANWC M29417, M29419

*Phascomurexia naso*: ANWC M29615; M29615

*Myoictis melas*: AM M15612, M8907, M15613; AMNH M-221649

*Phascogale tapoatafa*: AM M33612, M35234, M11630

*Antechinus flavipes*: AM M42734, M13586, M38538

*Parantechinus apicalis*: AM M21043, M21044; M21045, M21879

*Pseudantechinus bilarni*: NTM U4776

*Pseudantechinus macdonnellensis*: AM M37487; NTM U4516, U3259, U3244, U4820, U4514

*Antechinomys laniger*: AM M8428, M6954, M8460, M4641, M8465, M8463, M22870

*Dasycercus cristicauda*: AM M4356, M4866, M26821; AR1230-1241

*Dasykaluta rosamondae*: AM M12341

*Dasyuroides byrnei*: AM M8506, M8505, M24651, M24652; AR9360

*Phascolosorex dorsalis*: AM M28158, M8902, M12650, M19082

†*Dasyurus dunmalli*: AR5999 (cast of QM F742)

*Dasyurus hallucatus*: AM M24653, M40299

*Dasyurus maculatus*: AM S1560, S1146, M42614

*Dasyurus albopunctatus*: AM M23576, M23604

*Sarcophilus harrisii*: AM M42715, S1224

*Thylacinus cynocephalus*: AM S383, P763
