# Supplementary material for: Total evidence phylogeny and evolutionary timescale for Australian faunivorous marsupials (Dasyuromorphia)
Source: BMC Evol Biol. 2017 Dec 4;17:240. doi: 10.1186/s12862-017-1090-0 (PMC5715987; doi:10.1186/s12862-017-1090-0)
Supplement: Supplementary file 4 — List of fossil calibrations and justifications for these. (DOCX 43 kb) [file 12862_2017_1090_MOESM4_ESM.docx]

**Text S4. Node Calibrations**

Node-dating analyses:

The following six node calibrations were implemented in our molecular node-dating analyses (NodeCalib1 and NodeCalib2). In NodeCalib1, all six node calibrations were specified as offset exponential distributions, with a ‘hard’ minimum bound, and a ‘soft’ maximum bound such that there was a 5% probability that the divergence date is older than this. In NodeCalib2, node calibrations 1, 2, 3 and 5 were specified as uniform distributions with ‘hard’ minimum and maximum bounds, reflecting the particularly poor or uncertain fossil records of these groups, whilst node calibrations 4 and 6 were maintained as offset exponential distributions.

1. Didelphimorphia-Australidelphia split (= the root in the node-dating analyses)

Minimimum bound: 54.55 MYA

Maximum bound: 83.6 MYA

Justification: the minimum bound is based on the minimum age of *Djarthia murgonensis* from the early Eocene Tingamarra Local Fauna, which is the oldest known australidelphian [1, 2]; putative older records of crown-clade marsupials are questionable [3, 4]. The maximum bound is based on the maximum age of the Campanian [5, 6]: based on available evidence, Marsupialia probably originated in South America, and metatherians do not appear to have reached South America until the Maastrichtian at the earliest [see ref. 7: supporting information].

1. *Caluromys*-*Didelphis*-*Marmosa* split (= crown-clade Didelphidae)

Minimum bound: 12.1 MYA

Maximum bound: 65.7 MYA

Justification: the minimum bound is based on the minimum age of the marmosine *Micoureus laventicus* (= *Marmosa laventica*) from the Monkey Unit of the Honda Group in Colombia, which is the oldest definitive crown-clade didelphid [7, 8]. Identification of older didelphids is difficult due to the general absence of compelling dental synapomorphies of didelphid subclades [9]. However, assuming that Didelphidae originated in South America, we use the age of the oldest known definitive metatherians from South America, from the Punta Peligro fauna [10-12], as a conservative maximum bound; this is considerably older than recent published molecular estimates for the age of Didelphidae [7, 13, 14].

1. *Dromiciops*-Peramelemorphia-Dasyuromorphia split (= crown-clade Australidelphia)

Minimum bound: 44.54 MYA

Maximum bound: 83.6 MYA

Justification: the minimum bound is based on the minimum age of the microbiotherian *Woodburnodon casei* which is from the TELM 5/Cucullaea I Member of the La Meseta Formation, Seymour Island, and is the oldest definitive crown-clade australidelphian [15-17], The maximum bound is based on the maximum age of the Campanian, following the same considerations as for node calibration 1 (the root; see above).

1. *Echymipera*-*Perameles* split (= crown-clade Peramelidae)

Minimum bound: 3.62 MYA

Maximum bound: 28.1 MYA

Justification: the minimum bound is based on the minimum age of the perameline *Perameles allinghamensis*, from the early Pliocene Bluff Downs Local Fauna, which is the oldest definitive crown-clade peramelid [18-20]. The maximum bound is based on the maximum age of Riversleigh Faunal Zone A [21], from which numerous well-preserved fossil peramelemorphians are known, all of which fall well outside Peramelidae in published phylogenetic analyses [18, 20, 22].

1. Dasyuridae-*Myrmecobius*-*Thylacinus* split (= crown-clade Dasyuromorphia)

Minimum bound: 23.03 MYA

Maximum bound: 65.7 MYA

Jusitification: the minimum bound is based on the minimum age of *Badjcinus turnbulli*, which is from Riversleigh Fauna Zone A and has been identified as a thylacinid, rendering it the oldest definitive crown-clade dasyuromorphian [23-26]. Definitive crown-clade dasyuromorphians have not been described from the only terrestrial mammal-bearing fossil site in Australia, namely the early Eocene Tingamarra fauna. However, given that mammal fossils from this site are highly fragmentary (largely isolated teeth), and that identifying definitive crown-clade dasyuromorphians based on isolated teeth alone is extremely difficult, we do not use the age of the Tingamarra fauna as a maximum bound for this node. Instead, we use the maximum age of the South American Punta Peligro fauna as a more conservative maximum bound, following the same considerations as for node calibration 2 (Didelphidae; see above).

1. Dasyurini-Phascogalini split (= crown-clade Dasyurinae)

Minimum bound: 3.4 MYA

Maximum bound: 28.1 MYA

Justification: the minimum bound is based on the minimum age of *Dasyurus dunmalli* from the Pliocene Chinchilla Local Fauna, which is the oldest definitive crown-clade dasyurine [27-30]. The maximum bound is based on the maximum age of Riversleigh Faunal Zone A [21], from which numerous well-preserved fossil dasyuromorphians are known, all of which fall well outside Dasyurinae in published phylogenetic analyses [24-26, 31].

Tip-dating analyses:

For our tip dating analyses (TipCalib1 and TipCalib2), we implemented node calibration 1 above, plus an additional calibration is on the root (which represents a more inclusive grouping than in the molecular node dating analysis, due to the inclusion of the fossil stem-marsupials *Andinodelphys*, *Mayulestes* and *Pucadelphys*), and was specified as follows:

1. *Andinodelphys*-*Mayulestes*-*Pucadelphys*-Marsupialia split (= the root in the tip-dating analyses)

Minimum bound: 59.201 MYA

Maximum bound: 83.6 MYA

Justification: the minimum bound is very slightly (0.001 MYA) older than the minimum age of the oldest fossil taxa in the total evidence matrix, namely *Andinodelphys*, *Mayulestes* and *Pucadelphys*, which is required for the MrBayes analysis to run. The maximum bound is based on the maximum age of the Campanian (see node calibrations 1 and 3 used for the molecular node-dating analyses, above).

In TipCalib1, these two node calibrations (i.e. 1 and 7) were implemented as offset exponential distributions, with a ‘hard’ minimum bound and a ‘soft’ maximum bound such that there was a 5% probability that the divergence date is older than this. In TipCalib2, they were both implemented as uniform distributions with ‘hard’ minimum and maximum bounds.

Tip-and-node-dating analyses:

For our tip-and-node dating analyses (TipNodeCalib1 and TipNodeCalib2), we implemented all seven node calibrations above. In TipNodeCalib1, all seven two node calibrations were implemented as offset exponential distributions, with a ‘hard’ minimum bound and a ‘soft’ maximum bound such that there was a 5% probability that the divergence date is older than this. In TipNodeCalib2, node calibrations 1, 2, 3, 5 and 7 were specified as uniform distributions with ‘hard’ minimum and maximum bounds, reflecting the particularly poor or uncertain fossil records of these groups, whilst node calibrations 4 and 6 were maintained as offset exponential distributions.

References:

1. Beck RMD, Godthelp H, Weisbecker V, Archer M, Hand SJ: **Australia’s oldest marsupial fossils and their biogeographical implications**. *PLoS ONE* 2008, **3**(3):e1858.

2. Godthelp H, Archer M, Cifelli RL, Hand SJ, Gilkeson CF: **Earliest known Australian Tertiary mammal fauna**. *Nature* 1992, **356**:514-516.

3. Beck RMD: **The skull of *Epidolops ameghinoi* from the early Eocene Itaboraí fauna, southeastern Brazil, and the affinities of the extinct marsupialiform order Polydolopimorphia**. *J Mamm Evol* 2016.

4. Beck RMD: **Current understanding of the phylogeny of Metatheria: A review**. In: *New World marsupials and their extinct relatives: 100 million years of evolution.* Edited by Goin FJ, Forasiepi AM: Springer; in press.

5. Cohen KM, Finney SC, Gibbard PL, Fan J: **The ICS International Chronostratigraphic Chart**. *Episodes* 2013; updated, **36**:199-204.

6. Gradstein FM, Ogg JG, Schmitz MD, Ogg GM: **The geologic time scale**. Oxford: Elsevier; 2012.

7. Jansa SA, Barker FK, Voss RS: **The early diversification history of didelphid marsupials: a window into South America's "splendid isolation"**. *Evolution* 2014, **68**(3):684-695.

8. Goin FJ: **New clues for understanding Neogene marsupial radiations**. In: *Vertebrate paleontology in the Neotropics: the Miocene fauna of La Venta, Colombia.* Edited by Kay RF, Madden RH, Cifelli RL, Flynn JJ. Washington: Smithsonian Institution Press; 1997: 187-206.

9. Voss RS, Jansa SA: **Phylogenetic relationships and classification of didelphid marsupials, an extant radiation of New World metatherian mammals**. *Bull Am Mus Nat Hist* 2009, **322**:1-177.

10. Goin FJ, Forasiepi AM, Candela AM, Ortiz Jaureguizar E, Pascual R, Archer M, Godthelp H, Muirhead J, Augee M, Hand S *et al*: **Earliest Paleocene marsupials from Patagonia**. In: *I International Palaeontological Congress, Sydney: 2002; Sydney*. 68.

11. Forasiepi AM, Rougier GW: **Additional data on early Paleocene metatherians (Mammalia) from Punta Peligro (Salamanca Formation, Argentina): comments based on petrosal morphology**. *J Zool Syst Evol Res* 2009, **47**(4):391-398.

12. Clyde WC, Wilf P, Iglesias A, Slingerland RL, Barnum T, Bijl PK, Bralower TJ, Brinkhuis H, Comer EE, Huber BT *et al*: **New age constraints for the Salamanca Formation and lower Río Chico Group in the western San Jorge Basin, Patagonia, Argentina: implications for Cretaceous-Paleogene extinction recovery and land mammal age correlations**. *Geol Soc Am Bull* 2014, **126**(3-4):289-306.

13. Mitchell KJ, Pratt RC, Watson LN, Gibb GC, Llamas B, Kasper M, Edson J, Hopwood B, Male D, Armstrong KN *et al*: **Molecular phylogeny, biogeography, and habitat preference evolution of marsupials**. *Mol Biol Evol* 2014, **31**(9):2322-2330.

14. Meredith RW, Janecka JE, Gatesy J, Ryder OA, Fisher CA, Teeling EC, Goodbla A, Eizirik E, Simao TL, Stadler T *et al*: **Impacts of the Cretaceous Terrestrial Revolution and KPg extinction on mammal diversification**. *Science* 2011, **334**(6055):521-524.

15. Goin FJ, Zimicz N, Reguero MA, Santillana SN, Marenssi SA, Moly JJ: **New marsupial (Mammalia) from the Eocene of Antarctica, and the origins and affinities of the Microbiotheria**. *Rev Asoc Geol Argent* 2007, **62**(4):597-603.

16. Marenssi SA, Santillana SN: **Sr^87^/Sr^86^ derived ages from the lower Sobral Formation, Paleocene, Seymour Island, Antarctic Peninsula**. In: *9th International Symposium on Antarctic Earth Sciences (ISAES IX), Antarctic Contributions to Global Earth Sciences: 2003; Potsdam*. 219.

17. MacPhee RDE: **‘First’ appearances in the Cenozoic land-mammal record of the Greater Antilles: significance and comparison with South American and Antarctic records**. *J Biogeogr* 2005, **32**:551-564.

18. Warburton NM, Travouillon KJ: **The biology and palaeontology of the Peramelemorphia: a review of current knowledge and future research directions**. *Aust J Zool* 2016, **64**(3):151.

19. Mackness BS, Whitehead PW, McNamara GC: **New potassium-argon basalt date in relation to the Pliocene Bluff Downs Local Fauna, Northern Australia**. *Aust J Earth Sci* 2000, **47**(4):807-811.

20. Chamberlain PM, Travouillon KJ, Archer M, Hand SJ: ***Kutjamarcoot brevirostrum* gen. et sp. nov., a new short-snouted, early Miocene bandicoot (Marsupialia: Peramelemorphia) from the Kutjamarpu Local Fauna (Wipajiri Formation) in South Australia**. *Alcheringa* 2015, **40**(2):197-206.

21. Woodhead J, Hand SJ, Archer M, Graham I, Sniderman K, Arena DA, Black KH, Godthelp H, Creaser P, Price E: **Developing a radiometrically-dated chronologic sequence for Neogene biotic change in Australia, from the Riversleigh World Heritage Area of Queensland**. *Gondwana Res* 2014.

22. Kear BP, Aplin KP, Westerman M: **Bandicoot fossils and DNA elucidate lineage antiquity amongst xeric-adapted Australasian marsupials**. *Sci Rep* 2016, **6**:37537.

23. Muirhead J, Wroe S: **A new genus and species, *Badjcinus turnbulli* (Thylacinidae: Marsupialia), from the late Oligocene of Riversleigh, northern Australia, and an investigation of thylacinid phylogeny**. *J Vertebr Paleontol* 1998, **18**:612-626.

24. Wroe S, Musser A: **The skull of *Nimbacinus dicksoni* (Thylacinidae : Marsupialia)**. *Aust J Zool* 2001, **49**(5):487-514.

25. Murray PF, Megirian D: **Cranial morphology of the Miocene thylacinid *Mutpuracinus archibaldi* (Thylacinidae, Marsupialia) and relationships within the Dasyuromorphia)**. *Alcheringa* 2006, **Special Issue 1**:229-276.

26. Archer M, Hand SJ, Black KH, Beck RM, Arena DA, Wilson LA, Kealy S, Hung TT: **A new family of bizarre durophagous carnivorous marsupials from Miocene deposits in the Riversleigh World Heritage Area, northwestern Queensland**. *Sci Rep-Uk* 2016, **6**:26911.

27. Bartholomai A: ***Dasyurus dunmalli,* a new species of fossil marsupial (Dasyuridae) in the upper Cainozoic deposits of Queensland**. *Mem Queensl Mus* 1971, **16**(1):19-26.

28. Wroe S, Mackness BS: **Revision of the Pliocene dasyurid, *Dasyurus dunmalli* (Dasyuridae: Marsupialia)**. *Mem Queensl Mus* 1998, **42**(2):605-612.

29. Wroe S, Mackness BS: **Additional material of *Dasyurus dunmalli* from the Pliocene Chinchilla Local Fauna of Queensland and its phylogenetic implications**. *Mem Queensl Mus* 2000, **45**(2):641-645.

30. Louys JC, Price GJ: **The Chinchilla Local Fauna: an exceptionally rich and well-preserved Pliocene vertebrate assemblage from fluviatile deposits of south-eastern Queensland, Australia**. *Acta Palaeontol Pol* 2015, **60**:551-572.

31. Wroe S, Ebach M, Ahyong S, Muizon Cd, Muirhead J: **Cladistic analysis of dasyuromorphian (Marsupialia) phylogeny using cranial and dental characters**. *J Mammal* 2000, **81**(4):1008-1024.
