# Supplementary material for: Total evidence phylogeny and evolutionary timescale for Australian faunivorous marsupials (Dasyuromorphia)
Source: BMC Evol Biol. 2017 Dec 4;17:240. doi: 10.1186/s12862-017-1090-0 (PMC5715987; doi:10.1186/s12862-017-1090-0)
Supplement: Supplementary file 5 — Age ranges of fossil taxa and justifications for these. (DOCX 20 kb) [file 12862_2017_1090_MOESM5_ESM.docx]

**Text S3. Assumed age ranges (MYA) for fossil taxa included in phylogenetic analysis**

These age ranges were assumed for the fossil taxa in the “tip dating” (TipCalib1 and TipCalib2) and “tip-and-node dating” (TipNodeCalib1 and TipNodeCalib2) analyses, with the ages of modern taxa assumed to be 0 MYA. Beck and Lee (Beck and Lee 2014) provided detailed justification for the ages of several taxa, based on published sources.

| Taxon | Locality | Age range (MYA) | Justification | Reference(s) |
| --- | --- | --- | --- | --- |
| *Mayulestes ferox* | Tiupampa, Santa Lucia Formation, Bolivia | 66.0-59.2 | early or middle Palaeocene | (Beck and Lee 2014) |
| *Pucadelphys andinus* | Tiupampa, Santa Lucia Formation, Bolivia | 66.0-59.2 | early or middle Palaeocene | (Beck and Lee 2014) |
| *Andinodelphys cochabambensis* | Tiupampa, Santa Lucia Formation, Bolivia | 66.0-59.2 | early or middle Palaeocene | (Beck and Lee 2014) |
| *Djarthia murgonensis* | Tingamarra Local Fauna, southeastern Queensland, Australia | 54.65-54.55 | K/Ar radiometric dating | (Godthelp et al. 1992) |
| *Ankotarinja tirarensis* | Ditjimanka Local Fauna, Etadunna Formation, central South Australia | 26.1-23.6 | best-fit age-model for paleomagnetic data from the Etadunna  Formation | (Metzger and Retallack 2010) |
| *Keeuna woodburnei* | Ditjimanka Local Fauna, Etadunna Formation, central South Australia | 26.1-23.6 | best-fit age-model for paleomagnetic data from the Etadunna  Formation | (Metzger and Retallack 2010) |
| *Yarala burchfieldi* | Riversleigh Faunal Zone B, northwestern Queensland, Australia | 23.03-15.97 | early Miocene | (Arena et al. 2015) |
| *Mutpuracinus archibaldi* | Riversleigh Faunal Zone D, northwestern Queensland, Australia | 11.62-5.333 | late Miocene | (Arena et al. 2015) |
| *Barinya wangala* | Riversleigh Faunal Zones B and C, northwestern Queensland, Australia | 23.03-11.62 | early and middle Miocene | (Arena et al. 2015) |
| *Sminthopsis floravillensis* | Site 5C, Floraville Crossing, northwestern Queensland, Australia | 5.333-0.0117 | Plio-Pleistocene | (Archer 1982) |
| *Dasyuroides_achilpatna* | Fisherman’s Cliff Local Fauna, Moorna Formation, southwestern New South Wales, Australia | 2.87-2.42 | Magentostratigraphy | (Whitelaw 1991) |
| *Muribacinus gadiyuli* | Riversleigh Faunal Zone C, northwestern Queensland, Australia | 15.97-11.62 | middle Miocene | (Arena et al. 2015) |
| *Badjcinus turnbulli* | Riversleigh Faunal Zone A, northwestern Queensland, Australia | 28.1-23.03 | late Oligocene | (Arena et al. 2015) |
| *Nimbacinus dicksoni* | AL90 site, Riversleigh, northwestern Queensland, Australia | 15.11-14.53 | U–Pb radiometric dating | (Woodhead et al. 2014) |
| *Thylacinus macknessi* | Riversleigh Faunal Zone C, northwestern Queensland, Australia | 15.97-11.62 | middle Miocene | (Arena et al. 2015) |
| *Thylacinus potens* | Alcoota Local Fauna, Northern Territory, Australia | 11.62-5.333 | late Miocene | (Megirian et al. 2010) |
| *Ngamalacinus timmulvaneyi* | Riversleigh Faunal Zones B and C, northwestern Queensland, Australia | 23.03-11.62 | early and middle Miocene | (Arena et al. 2015) |

References

Archer M (1982) Review of the dasyurid (Marsupialia) fossil record, integration of data bearing on phylogenetic interpretation, and suprageneric classification. In: Archer M (ed) Carnivorous marsupials. Royal Zoological Society of New South Wales, Mosman, New South Wales, pp 397-443

Arena DA, Travouillon KJ, Beck RMD, Black KH, Gillespie AK, Myers TJ, Archer M, Hand SJ (2015) Mammalian lineages and the biostratigraphy and biochronology of Cenozoic faunas from the Riversleigh World Heritage Area, Australia. Lethaia 49 (1):43-60

Beck RMD, Lee MSY (2014) Ancient dates or accelerated rates? Morphological clocks and the antiquity of placental mammals. Proceedings of the Royal Society B: Biological Sciences 281:20141278. doi:10.1098/rspb.2014.1278

Godthelp H, Archer M, Cifelli RL, Hand SJ, Gilkeson CF (1992) Earliest known Australian Tertiary mammal fauna. Nature 356:514-516

Megirian D, Prideaux GJ, Murray PF, Smit N (2010) An Australian land mammal age biochronological scheme. Paleobiology 36 (4):658-671

Metzger CA, Retallack GJ (2010) Paleosol record of Neogene climate change in the Australian outback. Aust J Earth Sci 57:871-885

Whitelaw MJ (1991) Magnetic polarity stratigraphy of Pliocene and Pleistocene fossil vertebrate localities in southeastern Australia. Geol Soc Am Bull 103 (11):1493-1503

Woodhead J, Hand SJ, Archer M, Graham I, Sniderman K, Arena DA, Black KH, Godthelp H, Creaser P, Price E (2014) Developing a radiometrically-dated chronologic sequence for Neogene biotic change in Australia, from the Riversleigh World Heritage Area of Queensland. Gondwana Res. doi:10.1016/j.gr.2014.10.004
