# Supplementary material for: Total evidence phylogeny and evolutionary timescale for Australian faunivorous marsupials (Dasyuromorphia)
Source: BMC Evol Biol. 2017 Dec 4;17:240. doi: 10.1186/s12862-017-1090-0 (PMC5715987; doi:10.1186/s12862-017-1090-0)
Supplement: Supplementary file 6 — Tree topologies from all undated and dated analyses as figures and in Nexus format. (DOCX 2389 kb) [file 12862_2017_1090_MOESM6_ESM.docx]

**Text S6. All trees from dating analyses**

**Morphological data – maximum parsimony with *Myoictis leucura* deleted *a priori* (strict consensus)**

**
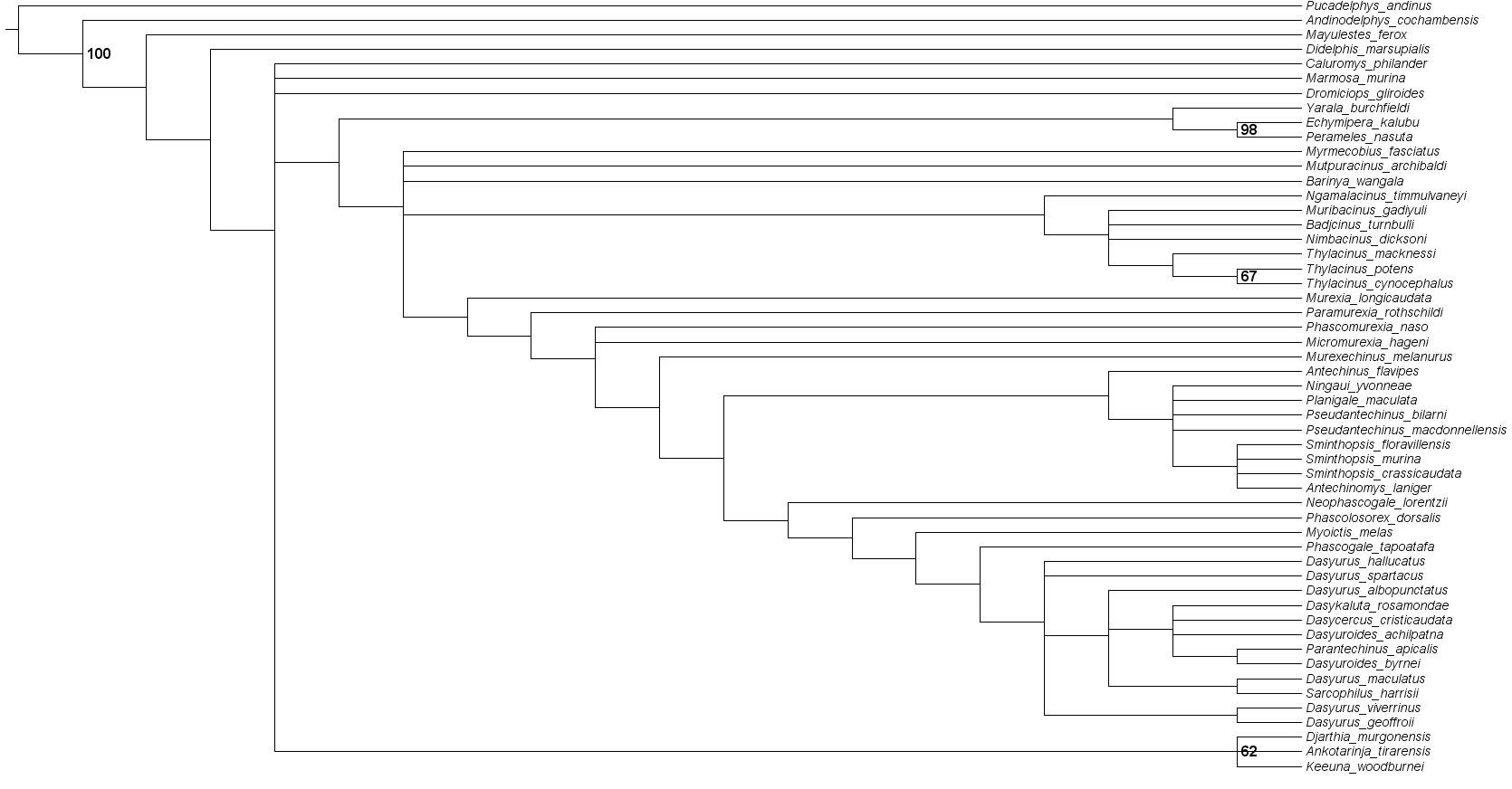
**

#NEXUS

BEGIN TAXA;

DIMENSIONS NTAX = 53;

TAXLABELS

Pucadelphys_andinus

Andinodelphys_cochambensis

Mayulestes_ferox

Didelphis_marsupialis

Caluromys_philander

Marmosa_murina

Dromiciops_gliroides

Yarala_burchfieldi

Echymipera_kalubu

Perameles_nasuta

Myrmecobius_fasciatus

Ngamalacinus_timmulvaneyi

Muribacinus_gadiyuli

Badjcinus_turnbulli

Nimbacinus_dicksoni

Thylacinus_macknessi

Thylacinus_potens

Thylacinus_cynocephalus

Mutpuracinus_archibaldi

Barinya_wangala

Murexia_longicaudata

Paramurexia_rothschildi

Murexechinus_melanurus

Antechinus_flavipes

Ningaui_yvonneae

Sminthopsis_floravillensis

Sminthopsis_murina

Sminthopsis_crassicaudata

Antechinomys_laniger

Planigale_maculata

Pseudantechinus_bilarni

Pseudantechinus_macdonnellensis

Neophascogale_lorentzii

Phascolosorex_dorsalis

Myoictis_melas

Phascogale_tapoatafa

Dasyurus_albopunctatus

Dasykaluta_rosamondae

Parantechinus_apicalis

Dasyuroides_byrnei

Dasycercus_cristicaudata

Dasyuroides_achilpatna

Dasyurus_maculatus

Sarcophilus_harrisii

Dasyurus_hallucatus

Dasyurus_spartacus

Dasyurus_viverrinus

Dasyurus_geoffroii

Phascomurexia_naso

Micromurexia_hageni

Djarthia_murgonensis

Ankotarinja_tirarensis

Keeuna_woodburnei

;

ENDBLOCK;

BEGIN TREES;

TRANSLATE

1 Pucadelphys_andinus,

2 Andinodelphys_cochambensis,

3 Mayulestes_ferox,

4 Didelphis_marsupialis,

5 Caluromys_philander,

6 Marmosa_murina,

7 Dromiciops_gliroides,

8 Yarala_burchfieldi,

9 Echymipera_kalubu,

10 Perameles_nasuta,

11 Myrmecobius_fasciatus,

12 Ngamalacinus_timmulvaneyi,

13 Muribacinus_gadiyuli,

14 Badjcinus_turnbulli,

15 Nimbacinus_dicksoni,

16 Thylacinus_macknessi,

17 Thylacinus_potens,

18 Thylacinus_cynocephalus,

19 Mutpuracinus_archibaldi,

20 Barinya_wangala,

21 Murexia_longicaudata,

22 Paramurexia_rothschildi,

23 Murexechinus_melanurus,

24 Antechinus_flavipes,

25 Ningaui_yvonneae,

26 Sminthopsis_floravillensis,

27 Sminthopsis_murina,

28 Sminthopsis_crassicaudata,

29 Antechinomys_laniger,

30 Planigale_maculata,

31 Pseudantechinus_bilarni,

32 Pseudantechinus_macdonnellensis,

33 Neophascogale_lorentzii,

34 Phascolosorex_dorsalis,

35 Myoictis_melas,

36 Phascogale_tapoatafa,

37 Dasyurus_albopunctatus,

38 Dasykaluta_rosamondae,

39 Parantechinus_apicalis,

40 Dasyuroides_byrnei,

41 Dasycercus_cristicaudata,

42 Dasyuroides_achilpatna,

43 Dasyurus_maculatus,

44 Sarcophilus_harrisii,

45 Dasyurus_hallucatus,

46 Dasyurus_spartacus,

47 Dasyurus_viverrinus,

48 Dasyurus_geoffroii,

49 Phascomurexia_naso,

50 Micromurexia_hageni,

51 Djarthia_murgonensis,

52 Ankotarinja_tirarensis,

53 Keeuna_woodburnei

;

TREE strict_consensus= (1,(2,(3,(4,(5,6,7,((8,(9,10)'98'),(11,19,20,(12,(13,14,15,(16,(17,18)'67'))),(21,(22,(49,50,(23,((24,(25,30,31,32,(26,27,28,29))),(33,(34,(35,(36,(45,46,(37,(38,41,42,(39,40)),(43,44)),(47,48))))))))))))),(51,52,53)'62'))))'100');

ENDBLOCK;

**Morphological data – undated Bayesian analysis with *Parantechinus apicalis* deleted *a priori* (50% majority rule consensus)**

**
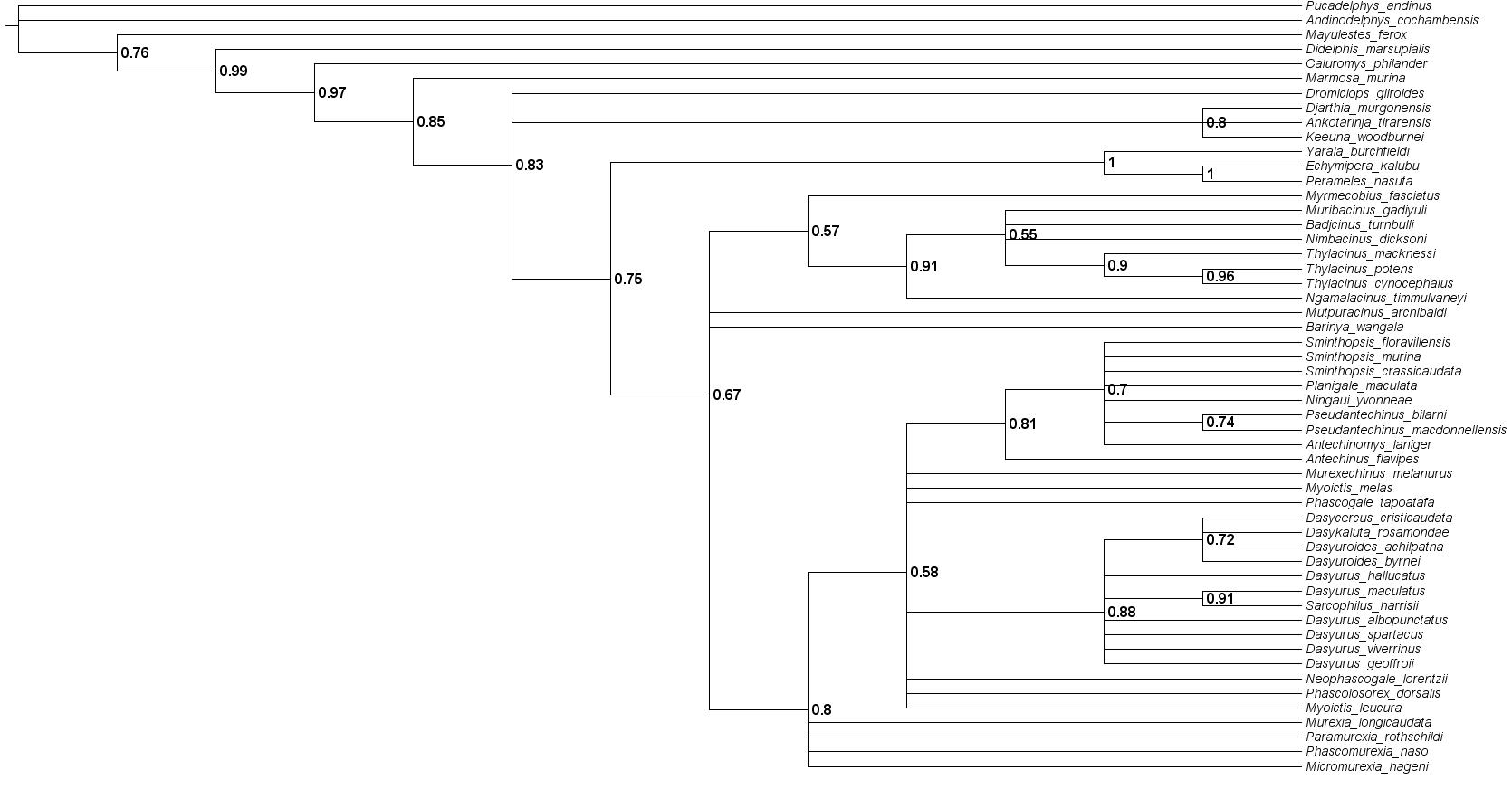
**

#NEXUS

begin taxa;

dimensions ntax=53;

taxlabels

Pucadelphys_andinus

Andinodelphys_cochambensis

Mayulestes_ferox

Didelphis_marsupialis

Caluromys_philander

Marmosa_murina

Dromiciops_gliroides

Djarthia_murgonensis

Ankotarinja_tirarensis

Keeuna_woodburnei

Yarala_burchfieldi

Echymipera_kalubu

Perameles_nasuta

Myrmecobius_fasciatus

Mutpuracinus_archibaldi

Barinya_wangala

Sminthopsis_floravillensis

Sminthopsis_murina

Sminthopsis_crassicaudata

Planigale_maculata

Ningaui_yvonneae

Murexia_longicaudata

Murexechinus_melanurus

Paramurexia_rothschildi

Phascomurexia_naso

Myoictis_melas

Phascogale_tapoatafa

Antechinus_flavipes

Pseudantechinus_bilarni

Pseudantechinus_macdonnellensis

Antechinomys_laniger

Dasycercus_cristicaudata

Dasykaluta_rosamondae

Dasyuroides_achilpatna

Dasyuroides_byrnei

Neophascogale_lorentzii

Phascolosorex_dorsalis

Dasyurus_hallucatus

Dasyurus_maculatus

Dasyurus_albopunctatus

Sarcophilus_harrisii

Muribacinus_gadiyuli

Badjcinus_turnbulli

Nimbacinus_dicksoni

Thylacinus_macknessi

Thylacinus_potens

Thylacinus_cynocephalus

Ngamalacinus_timmulvaneyi

Micromurexia_hageni

Myoictis_leucura

Dasyurus_spartacus

Dasyurus_viverrinus

Dasyurus_geoffroii

;

end;

begin trees;

translate

1 Pucadelphys_andinus,

2 Andinodelphys_cochambensis,

3 Mayulestes_ferox,

4 Didelphis_marsupialis,

5 Caluromys_philander,

6 Marmosa_murina,

7 Dromiciops_gliroides,

8 Djarthia_murgonensis,

9 Ankotarinja_tirarensis,

10 Keeuna_woodburnei,

11 Yarala_burchfieldi,

12 Echymipera_kalubu,

13 Perameles_nasuta,

14 Myrmecobius_fasciatus,

15 Mutpuracinus_archibaldi,

16 Barinya_wangala,

17 Sminthopsis_floravillensis,

18 Sminthopsis_murina,

19 Sminthopsis_crassicaudata,

20 Planigale_maculata,

21 Ningaui_yvonneae,

22 Murexia_longicaudata,

23 Murexechinus_melanurus,

24 Paramurexia_rothschildi,

25 Phascomurexia_naso,

26 Myoictis_melas,

27 Phascogale_tapoatafa,

28 Antechinus_flavipes,

29 Pseudantechinus_bilarni,

30 Pseudantechinus_macdonnellensis,

31 Antechinomys_laniger,

32 Dasycercus_cristicaudata,

33 Dasykaluta_rosamondae,

34 Dasyuroides_achilpatna,

35 Dasyuroides_byrnei,

36 Neophascogale_lorentzii,

37 Phascolosorex_dorsalis,

38 Dasyurus_hallucatus,

39 Dasyurus_maculatus,

40 Dasyurus_albopunctatus,

41 Sarcophilus_harrisii,

42 Muribacinus_gadiyuli,

43 Badjcinus_turnbulli,

44 Nimbacinus_dicksoni,

45 Thylacinus_macknessi,

46 Thylacinus_potens,

47 Thylacinus_cynocephalus,

48 Ngamalacinus_timmulvaneyi,

49 Micromurexia_hageni,

50 Myoictis_leucura,

51 Dasyurus_spartacus,

52 Dasyurus_viverrinus,

53 Dasyurus_geoffroii

;

tree con_50_majrule = [&U] (1[&prob=1.00000000e+000,prob_stddev=0.00000000e+000,prob_range={1.00000000e+000,1.00000000e+000},prob(percent)="100",prob+-sd="100+-0"]:2.544021e-002[&length_mean=3.69240385e-002,length_median=2.54402100e-002,length_95%HPD={6.29974000e-006,1.09517300e-001}],2[&prob=1.00000000e+000,prob_stddev=0.00000000e+000,prob_range={1.00000000e+000,1.00000000e+000},prob(percent)="100",prob+-sd="100+-0"]:2.193375e-002[&length_mean=3.21683522e-002,length_median=2.19337500e-002,length_95%HPD={1.62433700e-006,9.55373900e-002}],(3[&prob=1.00000000e+000,prob_stddev=0.00000000e+000,prob_range={1.00000000e+000,1.00000000e+000},prob(percent)="100",prob+-sd="100+-0"]:3.254667e-001[&length_mean=3.38948876e-001,length_median=3.25466700e-001,length_95%HPD={1.44815900e-001,5.71359100e-001}],(4[&prob=1.00000000e+000,prob_stddev=0.00000000e+000,prob_range={1.00000000e+000,1.00000000e+000},prob(percent)="100",prob+-sd="100+-0"]:1.671150e-001[&length_mean=1.79136030e-001,length_median=1.67115000e-001,length_95%HPD={4.01025000e-002,3.54477100e-001}],(5[&prob=1.00000000e+000,prob_stddev=0.00000000e+000,prob_range={1.00000000e+000,1.00000000e+000},prob(percent)="100",prob+-sd="100+-0"]:2.464127e-001[&length_mean=2.58384879e-001,length_median=2.46412700e-001,length_95%HPD={1.00210200e-001,4.39181600e-001}],(6[&prob=1.00000000e+000,prob_stddev=0.00000000e+000,prob_range={1.00000000e+000,1.00000000e+000},prob(percent)="100",prob+-sd="100+-0"]:9.029714e-002[&length_mean=1.00364435e-001,length_median=9.02971400e-002,length_95%HPD={5.39772200e-005,2.18943000e-001}],(7[&prob=1.00000000e+000,prob_stddev=0.00000000e+000,prob_range={1.00000000e+000,1.00000000e+000},prob(percent)="100",prob+-sd="100+-0"]:4.704142e-001[&length_mean=4.89447135e-001,length_median=4.70414200e-001,length_95%HPD={2.08886700e-001,8.07939300e-001}],(8[&prob=1.00000000e+000,prob_stddev=0.00000000e+000,prob_range={1.00000000e+000,1.00000000e+000},prob(percent)="100",prob+-sd="100+-0"]:2.891529e-002[&length_mean=4.36210298e-002,length_median=2.89152900e-002,length_95%HPD={6.07498600e-006,1.35744500e-001}],9[&prob=1.00000000e+000,prob_stddev=0.00000000e+000,prob_range={1.00000000e+000,1.00000000e+000},prob(percent)="100",prob+-sd="100+-0"]:3.072170e-002[&length_mean=4.53949081e-002,length_median=3.07217000e-002,length_95%HPD={1.20681800e-006,1.40720200e-001}],10[&prob=1.00000000e+000,prob_stddev=0.00000000e+000,prob_range={1.00000000e+000,1.00000000e+000},prob(percent)="100",prob+-sd="100+-0"]:3.540443e-002[&length_mean=5.28818470e-002,length_median=3.54044300e-002,length_95%HPD={7.15810800e-006,1.61446200e-001}])[&prob=8.01465690e-001,prob_stddev=1.05899347e-002,prob_range={7.90139907e-001,8.11459027e-001},prob(percent)="80",prob+-sd="80+-1"]:1.236894e-001[&length_mean=1.45172924e-001,length_median=1.23689400e-001,length_95%HPD={8.93121100e-004,3.39470800e-001}],((11[&prob=1.00000000e+000,prob_stddev=0.00000000e+000,prob_range={1.00000000e+000,1.00000000e+000},prob(percent)="100",prob+-sd="100+-0"]:7.814744e-002[&length_mean=1.02701245e-001,length_median=7.81474400e-002,length_95%HPD={1.69938100e-005,2.84479700e-001}],(12[&prob=1.00000000e+000,prob_stddev=0.00000000e+000,prob_range={1.00000000e+000,1.00000000e+000},prob(percent)="100",prob+-sd="100+-0"]:6.109630e-002[&length_mean=6.76475404e-002,length_median=6.10963000e-002,length_95%HPD={1.26617800e-006,1.52744400e-001}],13[&prob=1.00000000e+000,prob_stddev=0.00000000e+000,prob_range={1.00000000e+000,1.00000000e+000},prob(percent)="100",prob+-sd="100+-0"]:8.181880e-002[&length_mean=8.55316103e-002,length_median=8.18188000e-002,length_95%HPD={3.64386500e-004,1.69985500e-001}])[&prob=1.00000000e+000,prob_stddev=0.00000000e+000,prob_range={1.00000000e+000,1.00000000e+000},prob(percent)="100",prob+-sd="100+-0"]:5.180656e-001[&length_mean=5.42457713e-001,length_median=5.18065600e-001,length_95%HPD={2.16796900e-001,9.51784300e-001}])[&prob=9.99000666e-001,prob_stddev=8.60089573e-004,prob_range={9.98001332e-001,1.00000000e+000},prob(percent)="100",prob+-sd="100+-0"]:4.345017e-001[&length_mean=4.62783513e-001,length_median=4.34501700e-001,length_95%HPD={1.18954000e-001,8.37419900e-001}],((14[&prob=1.00000000e+000,prob_stddev=0.00000000e+000,prob_range={1.00000000e+000,1.00000000e+000},prob(percent)="100",prob+-sd="100+-0"]:3.372480e-001[&length_mean=3.52843215e-001,length_median=3.37248000e-001,length_95%HPD={1.33046400e-001,5.82119900e-001}],((42[&prob=1.00000000e+000,prob_stddev=0.00000000e+000,prob_range={1.00000000e+000,1.00000000e+000},prob(percent)="100",prob+-sd="100+-0"]:3.309166e-002[&length_mean=4.91361519e-002,length_median=3.30916600e-002,length_95%HPD={6.82679700e-006,1.52836400e-001}],43[&prob=1.00000000e+000,prob_stddev=0.00000000e+000,prob_range={1.00000000e+000,1.00000000e+000},prob(percent)="100",prob+-sd="100+-0"]:1.273638e-001[&length_mean=1.41675387e-001,length_median=1.27363800e-001,length_95%HPD={3.28125300e-003,2.98094500e-001}],44[&prob=1.00000000e+000,prob_stddev=0.00000000e+000,prob_range={1.00000000e+000,1.00000000e+000},prob(percent)="100",prob+-sd="100+-0"]:5.131034e-002[&length_mean=6.43912520e-002,length_median=5.13103400e-002,length_95%HPD={2.66834400e-005,1.68306900e-001}],(45[&prob=1.00000000e+000,prob_stddev=0.00000000e+000,prob_range={1.00000000e+000,1.00000000e+000},prob(percent)="100",prob+-sd="100+-0"]:9.074024e-002[&length_mean=1.17195625e-001,length_median=9.07402400e-002,length_95%HPD={1.61648300e-005,3.16489400e-001}],(46[&prob=1.00000000e+000,prob_stddev=0.00000000e+000,prob_range={1.00000000e+000,1.00000000e+000},prob(percent)="100",prob+-sd="100+-0"]:3.662028e-002[&length_mean=5.27757461e-002,length_median=3.66202800e-002,length_95%HPD={5.99826400e-006,1.58464400e-001}],47[&prob=1.00000000e+000,prob_stddev=0.00000000e+000,prob_range={1.00000000e+000,1.00000000e+000},prob(percent)="100",prob+-sd="100+-0"]:6.081830e-002[&length_mean=7.43667019e-002,length_median=6.08183000e-002,length_95%HPD={6.33725000e-005,1.86374000e-001}])[&prob=9.63191206e-001,prob_stddev=3.45644735e-003,prob_range={9.60026649e-001,9.67355097e-001},prob(percent)="96",prob+-sd="96+-0"]:1.629629e-001[&length_mean=1.76017773e-001,length_median=1.62962900e-001,length_95%HPD={1.65855000e-002,3.57209500e-001}])[&prob=9.04063957e-001,prob_stddev=6.41329295e-003,prob_range={8.97401732e-001,9.12724850e-001},prob(percent)="90",prob+-sd="90+-1"]:1.370213e-001[&length_mean=1.51251355e-001,length_median=1.37021300e-001,length_95%HPD={1.03235400e-002,3.21926700e-001}])[&prob=5.48301133e-001,prob_stddev=9.92649196e-003,prob_range={5.35642905e-001,5.58294470e-001},prob(percent)="55",prob+-sd="55+-1"]:8.691447e-002[&length_mean=1.04072728e-001,length_median=8.69144700e-002,length_95%HPD={2.21785300e-004,2.52979800e-001}],48[&prob=1.00000000e+000,prob_stddev=0.00000000e+000,prob_range={1.00000000e+000,1.00000000e+000},prob(percent)="100",prob+-sd="100+-0"]:4.807482e-002[&length_mean=6.64898512e-002,length_median=4.80748200e-002,length_95%HPD={1.26776300e-005,1.92094600e-001}])[&prob=9.11892072e-001,prob_stddev=5.74073549e-003,prob_range={9.05396402e-001,9.16722185e-001},prob(percent)="91",prob+-sd="91+-1"]:2.014413e-001[&length_mean=2.16306539e-001,length_median=2.01441300e-001,length_95%HPD={1.67368200e-003,4.28097300e-001}])[&prob=5.70619587e-001,prob_stddev=9.97852185e-003,prob_range={5.60959360e-001,5.84277149e-001},prob(percent)="57",prob+-sd="57+-1"]:1.388776e-001[&length_mean=1.51425789e-001,length_median=1.38877600e-001,length_95%HPD={1.53679200e-004,3.18933000e-001}],15[&prob=1.00000000e+000,prob_stddev=0.00000000e+000,prob_range={1.00000000e+000,1.00000000e+000},prob(percent)="100",prob+-sd="100+-0"]:1.133466e-001[&length_mean=1.27875651e-001,length_median=1.13346600e-001,length_95%HPD={6.35946800e-003,2.82487000e-001}],16[&prob=1.00000000e+000,prob_stddev=0.00000000e+000,prob_range={1.00000000e+000,1.00000000e+000},prob(percent)="100",prob+-sd="100+-0"]:9.899477e-002[&length_mean=1.13818707e-001,length_median=9.89947700e-002,length_95%HPD={3.16503600e-003,2.56657500e-001}],((((17[&prob=1.00000000e+000,prob_stddev=0.00000000e+000,prob_range={1.00000000e+000,1.00000000e+000},prob(percent)="100",prob+-sd="100+-0"]:3.737716e-002[&length_mean=5.68890230e-002,length_median=3.73771600e-002,length_95%HPD={3.26831800e-006,1.75999700e-001}],18[&prob=1.00000000e+000,prob_stddev=0.00000000e+000,prob_range={1.00000000e+000,1.00000000e+000},prob(percent)="100",prob+-sd="100+-0"]:6.524066e-002[&length_mean=6.91604735e-002,length_median=6.52406600e-002,length_95%HPD={5.34468600e-005,1.42969400e-001}],19[&prob=1.00000000e+000,prob_stddev=0.00000000e+000,prob_range={1.00000000e+000,1.00000000e+000},prob(percent)="100",prob+-sd="100+-0"]:8.358839e-002[&length_mean=8.92988034e-002,length_median=8.35883900e-002,length_95%HPD={1.02634100e-002,1.72012100e-001}],20[&prob=1.00000000e+000,prob_stddev=0.00000000e+000,prob_range={1.00000000e+000,1.00000000e+000},prob(percent)="100",prob+-sd="100+-0"]:1.494340e-001[&length_mean=1.53380733e-001,length_median=1.49434000e-001,length_95%HPD={5.21645500e-002,2.76294800e-001}],21[&prob=1.00000000e+000,prob_stddev=0.00000000e+000,prob_range={1.00000000e+000,1.00000000e+000},prob(percent)="100",prob+-sd="100+-0"]:2.984243e-002[&length_mean=4.18458084e-002,length_median=2.98424300e-002,length_95%HPD={5.78654000e-006,1.19880200e-001}],(29[&prob=1.00000000e+000,prob_stddev=0.00000000e+000,prob_range={1.00000000e+000,1.00000000e+000},prob(percent)="100",prob+-sd="100+-0"]:7.196546e-002[&length_mean=8.79265722e-002,length_median=7.19654600e-002,length_95%HPD={2.37629300e-005,2.23811600e-001}],30[&prob=1.00000000e+000,prob_stddev=0.00000000e+000,prob_range={1.00000000e+000,1.00000000e+000},prob(percent)="100",prob+-sd="100+-0"]:7.363355e-002[&length_mean=8.88489975e-002,length_median=7.36335500e-002,length_95%HPD={2.04734300e-005,2.20344900e-001}])[&prob=7.44337109e-001,prob_stddev=2.21854766e-002,prob_range={7.22185210e-001,7.74816789e-001},prob(percent)="74",prob+-sd="74+-2"]:1.208260e-001[&length_mean=1.35813913e-001,length_median=1.20826000e-001,length_95%HPD={7.28403000e-003,2.90171200e-001}],31[&prob=1.00000000e+000,prob_stddev=0.00000000e+000,prob_range={1.00000000e+000,1.00000000e+000},prob(percent)="100",prob+-sd="100+-0"]:1.840930e-001[&length_mean=1.80051182e-001,length_median=1.84093000e-001,length_95%HPD={7.30403300e-005,3.33057600e-001}])[&prob=6.98867422e-001,prob_stddev=1.70202143e-002,prob_range={6.73550966e-001,7.08860759e-001},prob(percent)="70",prob+-sd="70+-2"]:9.157019e-002[&length_mean=9.61499984e-002,length_median=9.15701900e-002,length_95%HPD={5.24712800e-005,1.86379600e-001}],28[&prob=1.00000000e+000,prob_stddev=0.00000000e+000,prob_range={1.00000000e+000,1.00000000e+000},prob(percent)="100",prob+-sd="100+-0"]:5.979857e-002[&length_mean=6.47957735e-002,length_median=5.97985700e-002,length_95%HPD={2.64394900e-003,1.34592200e-001}])[&prob=8.09293804e-001,prob_stddev=2.12522199e-002,prob_range={7.81479014e-001,8.28114590e-001},prob(percent)="81",prob+-sd="81+-2"]:8.678658e-002[&length_mean=9.16963224e-002,length_median=8.67865800e-002,length_95%HPD={1.55164900e-002,1.77883300e-001}],23[&prob=1.00000000e+000,prob_stddev=0.00000000e+000,prob_range={1.00000000e+000,1.00000000e+000},prob(percent)="100",prob+-sd="100+-0"]:6.982965e-002[&length_mean=7.39789338e-002,length_median=6.98296500e-002,length_95%HPD={4.94866700e-003,1.44339500e-001}],26[&prob=1.00000000e+000,prob_stddev=0.00000000e+000,prob_range={1.00000000e+000,1.00000000e+000},prob(percent)="100",prob+-sd="100+-0"]:7.661248e-002[&length_mean=7.99278251e-002,length_median=7.66124800e-002,length_95%HPD={2.74807400e-004,1.57404000e-001}],27[&prob=1.00000000e+000,prob_stddev=0.00000000e+000,prob_range={1.00000000e+000,1.00000000e+000},prob(percent)="100",prob+-sd="100+-0"]:1.881446e-001[&length_mean=1.92032940e-001,length_median=1.88144600e-001,length_95%HPD={7.97565000e-002,3.25811500e-001}],((32[&prob=1.00000000e+000,prob_stddev=0.00000000e+000,prob_range={1.00000000e+000,1.00000000e+000},prob(percent)="100",prob+-sd="100+-0"]:8.039859e-002[&length_mean=8.77584616e-002,length_median=8.03985900e-002,length_95%HPD={5.56417700e-005,1.94996300e-001}],33[&prob=1.00000000e+000,prob_stddev=0.00000000e+000,prob_range={1.00000000e+000,1.00000000e+000},prob(percent)="100",prob+-sd="100+-0"]:1.021402e-001[&length_mean=1.16200665e-001,length_median=1.02140200e-001,length_95%HPD={4.53815300e-003,2.55544300e-001}],34[&prob=1.00000000e+000,prob_stddev=0.00000000e+000,prob_range={1.00000000e+000,1.00000000e+000},prob(percent)="100",prob+-sd="100+-0"]:3.807190e-002[&length_mean=5.75199238e-002,length_median=3.80719000e-002,length_95%HPD={3.59510200e-006,1.78293800e-001}],35[&prob=1.00000000e+000,prob_stddev=0.00000000e+000,prob_range={1.00000000e+000,1.00000000e+000},prob(percent)="100",prob+-sd="100+-0"]:1.713841e-001[&length_mean=1.74667156e-001,length_median=1.71384100e-001,length_95%HPD={2.04459200e-002,3.18761900e-001}])[&prob=7.20019987e-001,prob_stddev=1.69886742e-002,prob_range={7.03530979e-001,7.43504330e-001},prob(percent)="72",prob+-sd="72+-2"]:6.814206e-002[&length_mean=7.46079769e-002,length_median=6.81420600e-002,length_95%HPD={2.58352800e-003,1.54337000e-001}],38[&prob=1.00000000e+000,prob_stddev=0.00000000e+000,prob_range={1.00000000e+000,1.00000000e+000},prob(percent)="100",prob+-sd="100+-0"]:1.025385e-001[&length_mean=1.07407672e-001,length_median=1.02538500e-001,length_95%HPD={3.40801800e-002,1.96329800e-001}],(39[&prob=1.00000000e+000,prob_stddev=0.00000000e+000,prob_range={1.00000000e+000,1.00000000e+000},prob(percent)="100",prob+-sd="100+-0"]:1.156783e-001[&length_mean=1.20208482e-001,length_median=1.15678300e-001,length_95%HPD={3.06595900e-002,2.12373300e-001}],41[&prob=1.00000000e+000,prob_stddev=0.00000000e+000,prob_range={1.00000000e+000,1.00000000e+000},prob(percent)="100",prob+-sd="100+-0"]:1.724960e-001[&length_mean=1.79090829e-001,length_median=1.72496000e-001,length_95%HPD={7.19577100e-002,2.98618900e-001}])[&prob=9.09726849e-001,prob_stddev=5.82071615e-003,prob_range={9.04063957e-001,9.15389740e-001},prob(percent)="91",prob+-sd="91+-1"]:8.832056e-002[&length_mean=9.43767600e-002,length_median=8.83205600e-002,length_95%HPD={1.14516700e-002,1.86370600e-001}],40[&prob=1.00000000e+000,prob_stddev=0.00000000e+000,prob_range={1.00000000e+000,1.00000000e+000},prob(percent)="100",prob+-sd="100+-0"]:8.866123e-002[&length_mean=9.36829260e-002,length_median=8.86612300e-002,length_95%HPD={1.48129800e-002,1.79165500e-001}],51[&prob=1.00000000e+000,prob_stddev=0.00000000e+000,prob_range={1.00000000e+000,1.00000000e+000},prob(percent)="100",prob+-sd="100+-0"]:2.881914e-002[&length_mean=3.73856066e-002,length_median=2.88191400e-002,length_95%HPD={1.97404500e-006,1.02650600e-001}],52[&prob=1.00000000e+000,prob_stddev=0.00000000e+000,prob_range={1.00000000e+000,1.00000000e+000},prob(percent)="100",prob+-sd="100+-0"]:2.414016e-002[&length_mean=3.01144366e-002,length_median=2.41401600e-002,length_95%HPD={1.48369300e-005,7.86470700e-002}],53[&prob=1.00000000e+000,prob_stddev=0.00000000e+000,prob_range={1.00000000e+000,1.00000000e+000},prob(percent)="100",prob+-sd="100+-0"]:2.593732e-002[&length_mean=3.86899451e-002,length_median=2.59373200e-002,length_95%HPD={1.15313700e-006,1.18085800e-001}])[&prob=8.76415723e-001,prob_stddev=1.75213567e-002,prob_range={8.52764823e-001,8.94070620e-001},prob(percent)="88",prob+-sd="88+-2"]:9.629919e-002[&length_mean=1.02023944e-001,length_median=9.62991900e-002,length_95%HPD={2.36641300e-002,1.91203700e-001}],36[&prob=1.00000000e+000,prob_stddev=0.00000000e+000,prob_range={1.00000000e+000,1.00000000e+000},prob(percent)="100",prob+-sd="100+-0"]:1.560105e-001[&length_mean=1.69344525e-001,length_median=1.56010500e-001,length_95%HPD={3.55463600e-002,3.44724600e-001}],37[&prob=1.00000000e+000,prob_stddev=0.00000000e+000,prob_range={1.00000000e+000,1.00000000e+000},prob(percent)="100",prob+-sd="100+-0"]:1.830272e-002[&length_mean=2.67167709e-002,length_median=1.83027200e-002,length_95%HPD={9.00574600e-006,8.22555300e-002}],50[&prob=1.00000000e+000,prob_stddev=0.00000000e+000,prob_range={1.00000000e+000,1.00000000e+000},prob(percent)="100",prob+-sd="100+-0"]:4.433234e-002[&length_mean=5.59226463e-002,length_median=4.43323400e-002,length_95%HPD={2.12160200e-005,1.44353700e-001}])[&prob=5.76615590e-001,prob_stddev=1.16350761e-002,prob_range={5.66289141e-001,5.90273151e-001},prob(percent)="58",prob+-sd="58+-1"]:6.787244e-002[&length_mean=7.23415713e-002,length_median=6.78724400e-002,length_95%HPD={4.20967800e-006,1.44664800e-001}],22[&prob=1.00000000e+000,prob_stddev=0.00000000e+000,prob_range={1.00000000e+000,1.00000000e+000},prob(percent)="100",prob+-sd="100+-0"]:1.338135e-001[&length_mean=1.38642749e-001,length_median=1.33813500e-001,length_95%HPD={3.23223000e-002,2.61874000e-001}],24[&prob=1.00000000e+000,prob_stddev=0.00000000e+000,prob_range={1.00000000e+000,1.00000000e+000},prob(percent)="100",prob+-sd="100+-0"]:4.604007e-002[&length_mean=5.16313517e-002,length_median=4.60400700e-002,length_95%HPD={1.07317200e-005,1.15333200e-001}],25[&prob=1.00000000e+000,prob_stddev=0.00000000e+000,prob_range={1.00000000e+000,1.00000000e+000},prob(percent)="100",prob+-sd="100+-0"]:1.071038e-001[&length_mean=1.08201125e-001,length_median=1.07103800e-001,length_95%HPD={2.64800100e-005,2.07268800e-001}],49[&prob=1.00000000e+000,prob_stddev=0.00000000e+000,prob_range={1.00000000e+000,1.00000000e+000},prob(percent)="100",prob+-sd="100+-0"]:2.872575e-002[&length_mean=4.07201723e-002,length_median=2.87257500e-002,length_95%HPD={6.37779700e-007,1.17879800e-001}])[&prob=7.95802798e-001,prob_stddev=1.33521808e-002,prob_range={7.78814124e-001,8.11459027e-001},prob(percent)="80",prob+-sd="80+-1"]:9.834999e-002[&length_mean=1.07096165e-001,length_median=9.83499900e-002,length_95%HPD={1.11853100e-002,2.15121100e-001}])[&prob=6.66555630e-001,prob_stddev=1.29813704e-002,prob_range={6.53564290e-001,6.80213191e-001},prob(percent)="67",prob+-sd="67+-1"]:1.887770e-001[&length_mean=2.06305098e-001,length_median=1.88777000e-001,length_95%HPD={1.65295400e-003,4.28422400e-001}])[&prob=7.47001999e-001,prob_stddev=7.14185153e-003,prob_range={7.38840773e-001,7.55496336e-001},prob(percent)="75",prob+-sd="75+-1"]:2.156758e-001[&length_mean=2.31698559e-001,length_median=2.15675800e-001,length_95%HPD={1.73695100e-002,4.82303500e-001}])[&prob=8.32944704e-001,prob_stddev=8.45122806e-003,prob_range={8.22118588e-001,8.42771486e-001},prob(percent)="83",prob+-sd="83+-1"]:1.543153e-001[&length_mean=1.67825990e-001,length_median=1.54315300e-001,length_95%HPD={7.92933600e-004,3.43627100e-001}])[&prob=8.53597602e-001,prob_stddev=6.37569497e-003,prob_range={8.44770153e-001,8.59427049e-001},prob(percent)="85",prob+-sd="85+-1"]:1.359093e-001[&length_mean=1.45806924e-001,length_median=1.35909300e-001,length_95%HPD={1.25250500e-002,2.88556500e-001}])[&prob=9.72684877e-001,prob_stddev=5.78246350e-003,prob_range={9.67355097e-001,9.78014657e-001},prob(percent)="97",prob+-sd="97+-1"]:2.321601e-001[&length_mean=2.42128497e-001,length_median=2.32160100e-001,length_95%HPD={4.86174200e-002,4.46539000e-001}])[&prob=9.94337109e-001,prob_stddev=1.76265910e-003,prob_range={9.92005330e-001,9.96002665e-001},prob(percent)="99",prob+-sd="99+-0"]:2.730560e-001[&length_mean=2.85426261e-001,length_median=2.73056000e-001,length_95%HPD={8.66595900e-002,5.09597100e-001}])[&prob=7.59160560e-001,prob_stddev=9.79898355e-003,prob_range={7.44836775e-001,7.66822119e-001},prob(percent)="76",prob+-sd="76+-1"]:9.242532e-002[&length_mean=1.04648637e-001,length_median=9.24253200e-002,length_95%HPD={1.77073800e-004,2.42857800e-001}]);

end;

**Combined molecular data – undated Bayesian analysis (majority rule consensus with all compatible clades)**


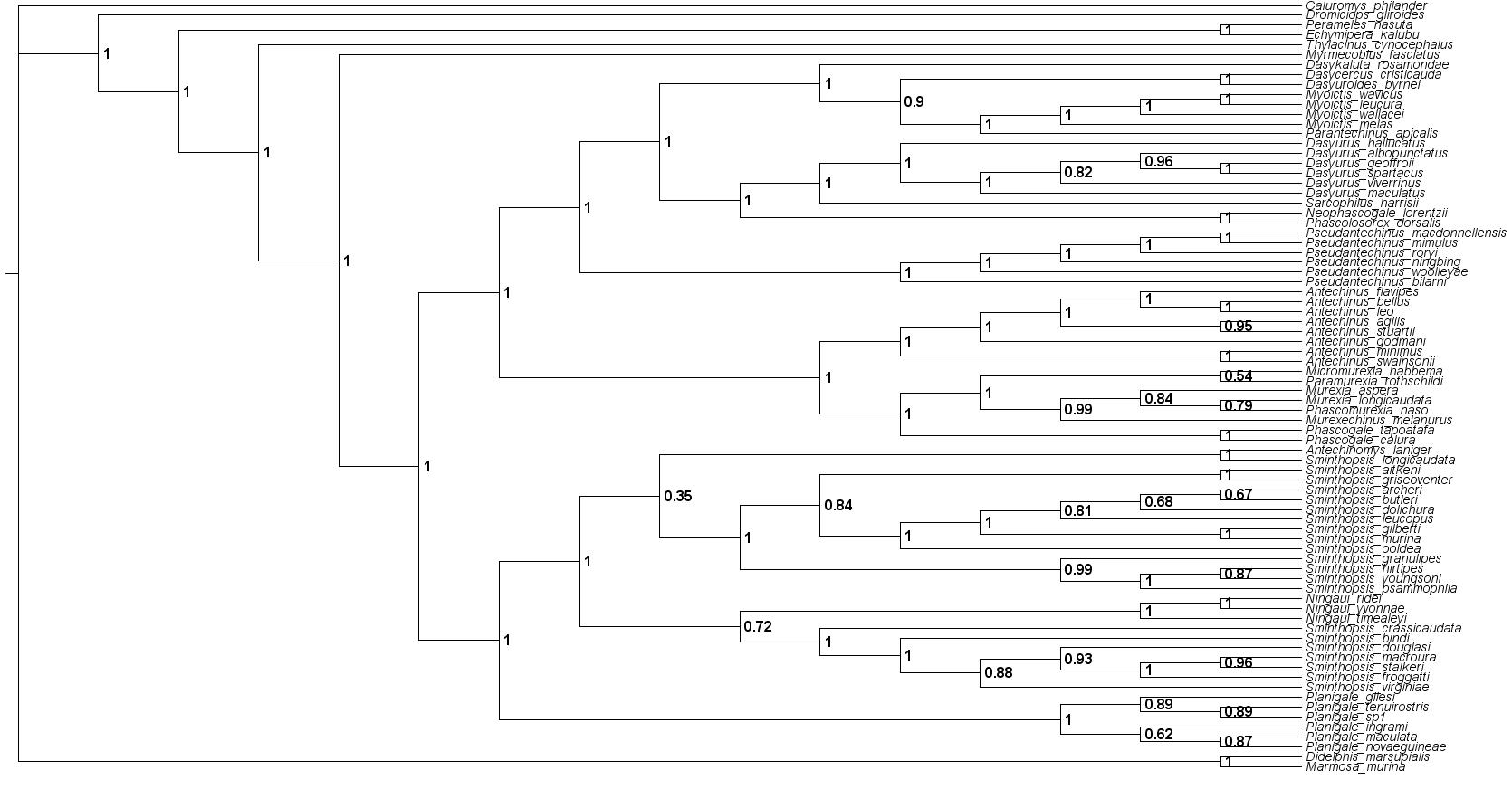


#NEXUS

begin taxa;

dimensions ntax=78;

taxlabels

Caluromys_philander

Didelphis_marsupialis

Marmosa_murina

Dromiciops_gliroides

Perameles_nasuta

Echymipera_kalubu

Thylacinus_cynocephalus

Myrmecobius_fasciatus

Dasykaluta_rosamondae

Dasycercus_cristicauda

Dasyuroides_byrnei

Dasyurus_hallucatus

Myoictis_wavicus

Neophascogale_lorentzii

Phascolosorex_dorsalis

Parantechinus_apicalis

Pseudantechinus_macdonnellensis

Sarcophilus_harrisii

Antechinus_flavipes

Micromurexia_habbema

Murexia_aspera

Murexia_longicaudata

Murexechinus_melanurus

Phascomurexia_naso

Paramurexia_rothschildi

Phascogale_tapoatafa

Antechinomys_laniger

Ningaui_ridei

Sminthopsis_crassicaudata

Planigale_gilesi

Dasyurus_albopunctatus

Dasyurus_geoffroii

Dasyurus_maculatus

Dasyurus_spartacus

Dasyurus_viverrinus

Myoictis_leucura

Myoictis_melas

Myoictis_wallacei

Pseudantechinus_bilarni

Pseudantechinus_mimulus

Pseudantechinus_ningbing

Pseudantechinus_roryi

Pseudantechinus_woolleyae

Antechinus_agilis

Antechinus_bellus

Antechinus_godmani

Antechinus_leo

Antechinus_minimus

Antechinus_stuartii

Antechinus_swainsonii

Phascogale_calura

Ningaui_timealeyi

Ningaui_yvonnae

Sminthopsis_aitkeni

Sminthopsis_archeri

Sminthopsis_bindi

Sminthopsis_butleri

Sminthopsis_dolichura

Sminthopsis_douglasi

Sminthopsis_gilberti

Sminthopsis_granulipes

Sminthopsis_griseoventer

Sminthopsis_hirtipes

Sminthopsis_leucopus

Sminthopsis_longicaudata

Sminthopsis_murina

Sminthopsis_macroura

Sminthopsis_psammophila

Sminthopsis_ooldea

Sminthopsis_virginiae

Sminthopsis_youngsoni

Sminthopsis_froggatti

Sminthopsis_stalkeri

Planigale_ingrami

Planigale_maculata

Planigale_novaeguineae

Planigale_tenuirostris

Planigale_sp1

;

end;

begin trees;

translate

1 Caluromys_philander,

2 Didelphis_marsupialis,

3 Marmosa_murina,

4 Dromiciops_gliroides,

5 Perameles_nasuta,

6 Echymipera_kalubu,

7 Thylacinus_cynocephalus,

8 Myrmecobius_fasciatus,

9 Dasykaluta_rosamondae,

10 Dasycercus_cristicauda,

11 Dasyuroides_byrnei,

12 Dasyurus_hallucatus,

13 Myoictis_wavicus,

14 Neophascogale_lorentzii,

15 Phascolosorex_dorsalis,

16 Parantechinus_apicalis,

17 Pseudantechinus_macdonnellensis,

18 Sarcophilus_harrisii,

19 Antechinus_flavipes,

20 Micromurexia_habbema,

21 Murexia_aspera,

22 Murexia_longicaudata,

23 Murexechinus_melanurus,

24 Phascomurexia_naso,

25 Paramurexia_rothschildi,

26 Phascogale_tapoatafa,

27 Antechinomys_laniger,

28 Ningaui_ridei,

29 Sminthopsis_crassicaudata,

30 Planigale_gilesi,

31 Dasyurus_albopunctatus,

32 Dasyurus_geoffroii,

33 Dasyurus_maculatus,

34 Dasyurus_spartacus,

35 Dasyurus_viverrinus,

36 Myoictis_leucura,

37 Myoictis_melas,

38 Myoictis_wallacei,

39 Pseudantechinus_bilarni,

40 Pseudantechinus_mimulus,

41 Pseudantechinus_ningbing,

42 Pseudantechinus_roryi,

43 Pseudantechinus_woolleyae,

44 Antechinus_agilis,

45 Antechinus_bellus,

46 Antechinus_godmani,

47 Antechinus_leo,

48 Antechinus_minimus,

49 Antechinus_stuartii,

50 Antechinus_swainsonii,

51 Phascogale_calura,

52 Ningaui_timealeyi,

53 Ningaui_yvonnae,

54 Sminthopsis_aitkeni,

55 Sminthopsis_archeri,

56 Sminthopsis_bindi,

57 Sminthopsis_butleri,

58 Sminthopsis_dolichura,

59 Sminthopsis_douglasi,

60 Sminthopsis_gilberti,

61 Sminthopsis_granulipes,

62 Sminthopsis_griseoventer,

63 Sminthopsis_hirtipes,

64 Sminthopsis_leucopus,

65 Sminthopsis_longicaudata,

66 Sminthopsis_murina,

67 Sminthopsis_macroura,

68 Sminthopsis_psammophila,

69 Sminthopsis_ooldea,

70 Sminthopsis_virginiae,

71 Sminthopsis_youngsoni,

72 Sminthopsis_froggatti,

73 Sminthopsis_stalkeri,

74 Planigale_ingrami,

75 Planigale_maculata,

76 Planigale_novaeguineae,

77 Planigale_tenuirostris,

78 Planigale_sp1

;

tree con_all_compat = [&U] (1[&prob=1.00000000e+00,prob_stddev=0.00000000e+00,prob_range={1.00000000e+00,1.00000000e+00},prob(percent)="100",prob+-sd="100+-0"]:7.143387e-01[&length_mean=6.92610258e-01,length_median=7.14338700e-01,length_95%HPD={3.24792100e-01,1.04271000e+00}],(4[&prob=1.00000000e+00,prob_stddev=0.00000000e+00,prob_range={1.00000000e+00,1.00000000e+00},prob(percent)="100",prob+-sd="100+-0"]:1.233934e+00[&length_mean=1.17939659e+00,length_median=1.23393400e+00,length_95%HPD={5.44251000e-01,1.75052900e+00}],((5[&prob=1.00000000e+00,prob_stddev=0.00000000e+00,prob_range={1.00000000e+00,1.00000000e+00},prob(percent)="100",prob+-sd="100+-0"]:3.228145e-01[&length_mean=3.16158699e-01,length_median=3.22814500e-01,length_95%HPD={1.50044900e-01,4.84226600e-01}],6[&prob=1.00000000e+00,prob_stddev=0.00000000e+00,prob_range={1.00000000e+00,1.00000000e+00},prob(percent)="100",prob+-sd="100+-0"]:3.676105e-01[&length_mean=3.61442157e-01,length_median=3.67610500e-01,length_95%HPD={1.73458800e-01,5.49510800e-01}])[&prob=1.00000000e+00,prob_stddev=0.00000000e+00,prob_range={1.00000000e+00,1.00000000e+00},prob(percent)="100",prob+-sd="100+-0"]:1.323940e+00[&length_mean=1.27439773e+00,length_median=1.32394000e+00,length_95%HPD={6.02164200e-01,1.92413700e+00}],(7[&prob=1.00000000e+00,prob_stddev=0.00000000e+00,prob_range={1.00000000e+00,1.00000000e+00},prob(percent)="100",prob+-sd="100+-0"]:1.122782e+00[&length_mean=1.08474357e+00,length_median=1.12278200e+00,length_95%HPD={4.92888400e-01,1.62666300e+00}],(8[&prob=1.00000000e+00,prob_stddev=0.00000000e+00,prob_range={1.00000000e+00,1.00000000e+00},prob(percent)="100",prob+-sd="100+-0"]:1.081665e+00[&length_mean=1.03506538e+00,length_median=1.08166500e+00,length_95%HPD={4.88742000e-01,1.53311300e+00}],(((((9[&prob=1.00000000e+00,prob_stddev=0.00000000e+00,prob_range={1.00000000e+00,1.00000000e+00},prob(percent)="100",prob+-sd="100+-0"]:3.428263e-01[&length_mean=3.29933893e-01,length_median=3.42826300e-01,length_95%HPD={1.63244700e-01,4.79974900e-01}],((10[&prob=1.00000000e+00,prob_stddev=0.00000000e+00,prob_range={1.00000000e+00,1.00000000e+00},prob(percent)="100",prob+-sd="100+-0"]:2.693297e-01[&length_mean=2.61709278e-01,length_median=2.69329700e-01,length_95%HPD={1.30645000e-01,3.86478700e-01}],11[&prob=1.00000000e+00,prob_stddev=0.00000000e+00,prob_range={1.00000000e+00,1.00000000e+00},prob(percent)="100",prob+-sd="100+-0"]:2.635887e-01[&length_mean=2.55687689e-01,length_median=2.63588700e-01,length_95%HPD={1.27724200e-01,3.74185800e-01}])[&prob=1.00000000e+00,prob_stddev=0.00000000e+00,prob_range={1.00000000e+00,1.00000000e+00},prob(percent)="100",prob+-sd="100+-0"]:6.916465e-02[&length_mean=6.99544886e-02,length_median=6.91646500e-02,length_95%HPD={3.15843300e-02,1.11066700e-01}],((((13[&prob=1.00000000e+00,prob_stddev=0.00000000e+00,prob_range={1.00000000e+00,1.00000000e+00},prob(percent)="100",prob+-sd="100+-0"]:7.431796e-02[&length_mean=7.62464111e-02,length_median=7.43179600e-02,length_95%HPD={3.10563600e-02,1.26622800e-01}],36[&prob=1.00000000e+00,prob_stddev=0.00000000e+00,prob_range={1.00000000e+00,1.00000000e+00},prob(percent)="100",prob+-sd="100+-0"]:5.908505e-02[&length_mean=6.18226629e-02,length_median=5.90850500e-02,length_95%HPD={2.20202100e-02,1.07239800e-01}])[&prob=1.00000000e+00,prob_stddev=0.00000000e+00,prob_range={1.00000000e+00,1.00000000e+00},prob(percent)="100",prob+-sd="100+-0"]:6.898821e-02[&length_mean=7.16114214e-02,length_median=6.89882100e-02,length_95%HPD={2.63095100e-02,1.19959300e-01}],38[&prob=1.00000000e+00,prob_stddev=0.00000000e+00,prob_range={1.00000000e+00,1.00000000e+00},prob(percent)="100",prob+-sd="100+-0"]:1.308924e-01[&length_mean=1.31643001e-01,length_median=1.30892400e-01,length_95%HPD={6.05635200e-02,2.07249200e-01}])[&prob=1.00000000e+00,prob_stddev=0.00000000e+00,prob_range={1.00000000e+00,1.00000000e+00},prob(percent)="100",prob+-sd="100+-0"]:4.449736e-02[&length_mean=4.69786025e-02,length_median=4.44973600e-02,length_95%HPD={1.58970500e-02,8.56944400e-02}],37[&prob=1.00000000e+00,prob_stddev=0.00000000e+00,prob_range={1.00000000e+00,1.00000000e+00},prob(percent)="100",prob+-sd="100+-0"]:1.556118e-01[&length_mean=1.54957701e-01,length_median=1.55611800e-01,length_95%HPD={7.11241100e-02,2.42490100e-01}])[&prob=1.00000000e+00,prob_stddev=0.00000000e+00,prob_range={1.00000000e+00,1.00000000e+00},prob(percent)="100",prob+-sd="100+-0"]:1.270083e-01[&length_mean=1.27521952e-01,length_median=1.27008300e-01,length_95%HPD={5.83513900e-02,1.97610100e-01}],16[&prob=1.00000000e+00,prob_stddev=0.00000000e+00,prob_range={1.00000000e+00,1.00000000e+00},prob(percent)="100",prob+-sd="100+-0"]:3.265758e-01[&length_mean=3.14761363e-01,length_median=3.26575800e-01,length_95%HPD={1.56687400e-01,4.62843600e-01}])[&prob=1.00000000e+00,prob_stddev=0.00000000e+00,prob_range={1.00000000e+00,1.00000000e+00},prob(percent)="100",prob+-sd="100+-0"]:3.396417e-02[&length_mean=3.59280117e-02,length_median=3.39641700e-02,length_95%HPD={1.35637200e-02,6.16038900e-02}])[&prob=9.04397069e-01,prob_stddev=2.52227775e-03,prob_range={9.02731512e-01,9.08061292e-01},prob(percent)="90",prob+-sd="90+-0"]:1.428135e-02[&length_mean=1.54152105e-02,length_median=1.42813500e-02,length_95%HPD={4.13190100e-03,2.98680100e-02}])[&prob=1.00000000e+00,prob_stddev=0.00000000e+00,prob_range={1.00000000e+00,1.00000000e+00},prob(percent)="100",prob+-sd="100+-0"]:2.954517e-02[&length_mean=3.07996595e-02,length_median=2.95451700e-02,length_95%HPD={1.23223900e-02,5.24304400e-02}],(((12[&prob=1.00000000e+00,prob_stddev=0.00000000e+00,prob_range={1.00000000e+00,1.00000000e+00},prob(percent)="100",prob+-sd="100+-0"]:3.415513e-01[&length_mean=3.31003675e-01,length_median=3.41551300e-01,length_95%HPD={1.64858100e-01,4.87350400e-01}],(((31[&prob=1.00000000e+00,prob_stddev=0.00000000e+00,prob_range={1.00000000e+00,1.00000000e+00},prob(percent)="100",prob+-sd="100+-0"]:1.521970e-01[&length_mean=1.51575608e-01,length_median=1.52197000e-01,length_95%HPD={6.90502800e-02,2.38151300e-01}],(32[&prob=1.00000000e+00,prob_stddev=0.00000000e+00,prob_range={1.00000000e+00,1.00000000e+00},prob(percent)="100",prob+-sd="100+-0"]:3.938366e-02[&length_mean=4.13384171e-02,length_median=3.93836600e-02,length_95%HPD={1.44870900e-02,7.15189900e-02}],34[&prob=1.00000000e+00,prob_stddev=0.00000000e+00,prob_range={1.00000000e+00,1.00000000e+00},prob(percent)="100",prob+-sd="100+-0"]:6.768581e-02[&length_mean=7.00814668e-02,length_median=6.76858100e-02,length_95%HPD={2.63616600e-02,1.19169400e-01}])[&prob=1.00000000e+00,prob_stddev=0.00000000e+00,prob_range={1.00000000e+00,1.00000000e+00},prob(percent)="100",prob+-sd="100+-0"]:9.080046e-02[&length_mean=9.22846377e-02,length_median=9.08004600e-02,length_95%HPD={4.09550200e-02,1.47362000e-01}])[&prob=9.63690873e-01,prob_stddev=6.44780419e-03,prob_range={9.55363091e-01,9.70686209e-01},prob(percent)="96",prob+-sd="96+-1"]:2.279406e-02[&length_mean=2.47942098e-02,length_median=2.27940600e-02,length_95%HPD={6.67005100e-03,4.67822100e-02}],35[&prob=1.00000000e+00,prob_stddev=0.00000000e+00,prob_range={1.00000000e+00,1.00000000e+00},prob(percent)="100",prob+-sd="100+-0"]:1.338638e-01[&length_mean=1.35340446e-01,length_median=1.33863800e-01,length_95%HPD={6.09970100e-02,2.16802100e-01}])[&prob=8.18287808e-01,prob_stddev=5.31240227e-03,prob_range={8.11459027e-01,8.24117255e-01},prob(percent)="82",prob+-sd="82+-1"]:1.069028e-02[&length_mean=1.22751560e-02,length_median=1.06902800e-02,length_95%HPD={9.36313700e-04,2.75789200e-02}],33[&prob=1.00000000e+00,prob_stddev=0.00000000e+00,prob_range={1.00000000e+00,1.00000000e+00},prob(percent)="100",prob+-sd="100+-0"]:1.955632e-01[&length_mean=1.93351617e-01,length_median=1.95563200e-01,length_95%HPD={9.06698500e-02,2.96795300e-01}])[&prob=1.00000000e+00,prob_stddev=0.00000000e+00,prob_range={1.00000000e+00,1.00000000e+00},prob(percent)="100",prob+-sd="100+-0"]:6.121866e-02[&length_mean=6.25036181e-02,length_median=6.12186600e-02,length_95%HPD={2.76035500e-02,1.03431200e-01}])[&prob=1.00000000e+00,prob_stddev=0.00000000e+00,prob_range={1.00000000e+00,1.00000000e+00},prob(percent)="100",prob+-sd="100+-0"]:5.774810e-02[&length_mean=5.92141317e-02,length_median=5.77481000e-02,length_95%HPD={2.55025500e-02,9.52950700e-02}],18[&prob=1.00000000e+00,prob_stddev=0.00000000e+00,prob_range={1.00000000e+00,1.00000000e+00},prob(percent)="100",prob+-sd="100+-0"]:3.449192e-01[&length_mean=3.32955398e-01,length_median=3.44919200e-01,length_95%HPD={1.64952300e-01,4.85263600e-01}])[&prob=1.00000000e+00,prob_stddev=0.00000000e+00,prob_range={1.00000000e+00,1.00000000e+00},prob(percent)="100",prob+-sd="100+-0"]:5.564909e-02[&length_mean=5.65644588e-02,length_median=5.56490900e-02,length_95%HPD={2.52329100e-02,9.18186900e-02}],(14[&prob=1.00000000e+00,prob_stddev=0.00000000e+00,prob_range={1.00000000e+00,1.00000000e+00},prob(percent)="100",prob+-sd="100+-0"]:1.992511e-01[&length_mean=1.93319463e-01,length_median=1.99251100e-01,length_95%HPD={9.51393600e-02,2.85820400e-01}],15[&prob=1.00000000e+00,prob_stddev=0.00000000e+00,prob_range={1.00000000e+00,1.00000000e+00},prob(percent)="100",prob+-sd="100+-0"]:2.175785e-01[&length_mean=2.10612396e-01,length_median=2.17578500e-01,length_95%HPD={1.04364500e-01,3.13897200e-01}])[&prob=1.00000000e+00,prob_stddev=0.00000000e+00,prob_range={1.00000000e+00,1.00000000e+00},prob(percent)="100",prob+-sd="100+-0"]:1.767406e-01[&length_mean=1.71896974e-01,length_median=1.76740600e-01,length_95%HPD={8.01726700e-02,2.53311500e-01}])[&prob=1.00000000e+00,prob_stddev=0.00000000e+00,prob_range={1.00000000e+00,1.00000000e+00},prob(percent)="100",prob+-sd="100+-0"]:5.965255e-02[&length_mean=5.97103846e-02,length_median=5.96525500e-02,length_95%HPD={2.76282500e-02,9.39682300e-02}])[&prob=1.00000000e+00,prob_stddev=0.00000000e+00,prob_range={1.00000000e+00,1.00000000e+00},prob(percent)="100",prob+-sd="100+-0"]:2.154498e-02[&length_mean=2.27930316e-02,length_median=2.15449800e-02,length_95%HPD={8.02401400e-03,3.96445900e-02}],(((((17[&prob=1.00000000e+00,prob_stddev=0.00000000e+00,prob_range={1.00000000e+00,1.00000000e+00},prob(percent)="100",prob+-sd="100+-0"]:5.648117e-02[&length_mean=5.88493745e-02,length_median=5.64811700e-02,length_95%HPD={2.24313600e-02,1.00466000e-01}],40[&prob=1.00000000e+00,prob_stddev=0.00000000e+00,prob_range={1.00000000e+00,1.00000000e+00},prob(percent)="100",prob+-sd="100+-0"]:1.031375e-01[&length_mean=1.04087022e-01,length_median=1.03137500e-01,length_95%HPD={4.49191600e-02,1.65659600e-01}])[&prob=1.00000000e+00,prob_stddev=0.00000000e+00,prob_range={1.00000000e+00,1.00000000e+00},prob(percent)="100",prob+-sd="100+-0"]:5.809594e-02[&length_mean=6.04832992e-02,length_median=5.80959400e-02,length_95%HPD={2.39532100e-02,1.02214500e-01}],42[&prob=1.00000000e+00,prob_stddev=0.00000000e+00,prob_range={1.00000000e+00,1.00000000e+00},prob(percent)="100",prob+-sd="100+-0"]:6.316033e-02[&length_mean=6.44431519e-02,length_median=6.31603300e-02,length_95%HPD={2.60243800e-02,1.08332400e-01}])[&prob=1.00000000e+00,prob_stddev=0.00000000e+00,prob_range={1.00000000e+00,1.00000000e+00},prob(percent)="100",prob+-sd="100+-0"]:5.049866e-02[&length_mean=5.31747030e-02,length_median=5.04986600e-02,length_95%HPD={1.85361200e-02,9.21850000e-02}],41[&prob=1.00000000e+00,prob_stddev=0.00000000e+00,prob_range={1.00000000e+00,1.00000000e+00},prob(percent)="100",prob+-sd="100+-0"]:1.694331e-01[&length_mean=1.68959949e-01,length_median=1.69433100e-01,length_95%HPD={7.32163100e-02,2.65744700e-01}])[&prob=1.00000000e+00,prob_stddev=0.00000000e+00,prob_range={1.00000000e+00,1.00000000e+00},prob(percent)="100",prob+-sd="100+-0"]:4.818469e-02[&length_mean=5.04409138e-02,length_median=4.81846900e-02,length_95%HPD={1.67470300e-02,8.70927400e-02}],43[&prob=1.00000000e+00,prob_stddev=0.00000000e+00,prob_range={1.00000000e+00,1.00000000e+00},prob(percent)="100",prob+-sd="100+-0"]:2.367156e-01[&length_mean=2.31607697e-01,length_median=2.36715600e-01,length_95%HPD={1.10693600e-01,3.56482200e-01}])[&prob=1.00000000e+00,prob_stddev=0.00000000e+00,prob_range={1.00000000e+00,1.00000000e+00},prob(percent)="100",prob+-sd="100+-0"]:9.574429e-02[&length_mean=9.68579470e-02,length_median=9.57442900e-02,length_95%HPD={4.27322400e-02,1.53402400e-01}],39[&prob=1.00000000e+00,prob_stddev=0.00000000e+00,prob_range={1.00000000e+00,1.00000000e+00},prob(percent)="100",prob+-sd="100+-0"]:2.446824e-01[&length_mean=2.37108742e-01,length_median=2.44682400e-01,length_95%HPD={1.19642300e-01,3.50459700e-01}])[&prob=1.00000000e+00,prob_stddev=0.00000000e+00,prob_range={1.00000000e+00,1.00000000e+00},prob(percent)="100",prob+-sd="100+-0"]:2.348775e-02[&length_mean=2.48654824e-02,length_median=2.34877500e-02,length_95%HPD={8.41408200e-03,4.38811000e-02}])[&prob=1.00000000e+00,prob_stddev=0.00000000e+00,prob_range={1.00000000e+00,1.00000000e+00},prob(percent)="100",prob+-sd="100+-0"]:2.507401e-01[&length_mean=2.41951540e-01,length_median=2.50740100e-01,length_95%HPD={1.19292000e-01,3.59251800e-01}],(((((19[&prob=1.00000000e+00,prob_stddev=0.00000000e+00,prob_range={1.00000000e+00,1.00000000e+00},prob(percent)="100",prob+-sd="100+-0"]:1.099788e-01[&length_mean=1.10591266e-01,length_median=1.09978800e-01,length_95%HPD={4.72447700e-02,1.72180100e-01}],(45[&prob=1.00000000e+00,prob_stddev=0.00000000e+00,prob_range={1.00000000e+00,1.00000000e+00},prob(percent)="100",prob+-sd="100+-0"]:1.269489e-01[&length_mean=1.28275116e-01,length_median=1.26948900e-01,length_95%HPD={5.51213300e-02,2.06116800e-01}],47[&prob=1.00000000e+00,prob_stddev=0.00000000e+00,prob_range={1.00000000e+00,1.00000000e+00},prob(percent)="100",prob+-sd="100+-0"]:9.232240e-02[&length_mean=9.48870006e-02,length_median=9.23224000e-02,length_95%HPD={3.92768000e-02,1.58029300e-01}])[&prob=1.00000000e+00,prob_stddev=0.00000000e+00,prob_range={1.00000000e+00,1.00000000e+00},prob(percent)="100",prob+-sd="100+-0"]:4.595337e-02[&length_mean=4.86927539e-02,length_median=4.59533700e-02,length_95%HPD={1.56695200e-02,8.66625400e-02}])[&prob=1.00000000e+00,prob_stddev=0.00000000e+00,prob_range={1.00000000e+00,1.00000000e+00},prob(percent)="100",prob+-sd="100+-0"]:3.773632e-02[&length_mean=4.04470408e-02,length_median=3.77363200e-02,length_95%HPD={1.12651500e-02,7.44896400e-02}],(44[&prob=1.00000000e+00,prob_stddev=0.00000000e+00,prob_range={1.00000000e+00,1.00000000e+00},prob(percent)="100",prob+-sd="100+-0"]:1.251530e-01[&length_mean=1.26283123e-01,length_median=1.25153000e-01,length_95%HPD={5.64328200e-02,2.03609300e-01}],49[&prob=1.00000000e+00,prob_stddev=0.00000000e+00,prob_range={1.00000000e+00,1.00000000e+00},prob(percent)="100",prob+-sd="100+-0"]:1.244419e-01[&length_mean=1.27194628e-01,length_median=1.24441900e-01,length_95%HPD={5.06626100e-02,2.03681300e-01}])[&prob=9.52365090e-01,prob_stddev=4.56739147e-03,prob_range={9.46702199e-01,9.56695536e-01},prob(percent)="95",prob+-sd="95+-0"]:2.054279e-02[&length_mean=2.28886803e-02,length_median=2.05427900e-02,length_95%HPD={3.59687000e-03,4.80005200e-02}])[&prob=1.00000000e+00,prob_stddev=0.00000000e+00,prob_range={1.00000000e+00,1.00000000e+00},prob(percent)="100",prob+-sd="100+-0"]:4.504971e-02[&length_mean=4.74387907e-02,length_median=4.50497100e-02,length_95%HPD={1.71626600e-02,8.43228000e-02}],46[&prob=1.00000000e+00,prob_stddev=0.00000000e+00,prob_range={1.00000000e+00,1.00000000e+00},prob(percent)="100",prob+-sd="100+-0"]:2.117682e-01[&length_mean=2.06187928e-01,length_median=2.11768200e-01,length_95%HPD={1.02179900e-01,3.04392300e-01}])[&prob=1.00000000e+00,prob_stddev=0.00000000e+00,prob_range={1.00000000e+00,1.00000000e+00},prob(percent)="100",prob+-sd="100+-0"]:7.525016e-02[&length_mean=7.57884520e-02,length_median=7.52501600e-02,length_95%HPD={3.37432800e-02,1.19321800e-01}],(48[&prob=1.00000000e+00,prob_stddev=0.00000000e+00,prob_range={1.00000000e+00,1.00000000e+00},prob(percent)="100",prob+-sd="100+-0"]:1.575903e-01[&length_mean=1.57296784e-01,length_median=1.57590300e-01,length_95%HPD={7.30669600e-02,2.52484700e-01}],50[&prob=1.00000000e+00,prob_stddev=0.00000000e+00,prob_range={1.00000000e+00,1.00000000e+00},prob(percent)="100",prob+-sd="100+-0"]:1.538999e-01[&length_mean=1.52712542e-01,length_median=1.53899900e-01,length_95%HPD={7.24236100e-02,2.36807800e-01}])[&prob=1.00000000e+00,prob_stddev=0.00000000e+00,prob_range={1.00000000e+00,1.00000000e+00},prob(percent)="100",prob+-sd="100+-0"]:8.810706e-02[&length_mean=8.96585599e-02,length_median=8.81070600e-02,length_95%HPD={3.89396800e-02,1.48328800e-01}])[&prob=1.00000000e+00,prob_stddev=0.00000000e+00,prob_range={1.00000000e+00,1.00000000e+00},prob(percent)="100",prob+-sd="100+-0"]:9.429729e-02[&length_mean=9.43711015e-02,length_median=9.42972900e-02,length_95%HPD={4.28257500e-02,1.46869400e-01}],(((20[&prob=1.00000000e+00,prob_stddev=0.00000000e+00,prob_range={1.00000000e+00,1.00000000e+00},prob(percent)="100",prob+-sd="100+-0"]:2.647150e-01[&length_mean=2.55501264e-01,length_median=2.64715000e-01,length_95%HPD={1.24155700e-01,3.79122600e-01}],25[&prob=1.00000000e+00,prob_stddev=0.00000000e+00,prob_range={1.00000000e+00,1.00000000e+00},prob(percent)="100",prob+-sd="100+-0"]:3.216527e-01[&length_mean=3.10534012e-01,length_median=3.21652700e-01,length_95%HPD={1.54292300e-01,4.52481100e-01}])[&prob=5.37475017e-01,prob_stddev=1.75118546e-02,prob_range={5.18321119e-01,5.53630913e-01},prob(percent)="54",prob+-sd="54+-2"]:2.256140e-02[&length_mean=2.43976817e-02,length_median=2.25614000e-02,length_95%HPD={6.75764000e-03,4.62241000e-02}],((21[&prob=1.00000000e+00,prob_stddev=0.00000000e+00,prob_range={1.00000000e+00,1.00000000e+00},prob(percent)="100",prob+-sd="100+-0"]:6.809173e-01[&length_mean=6.97528181e-01,length_median=6.80917300e-01,length_95%HPD={2.74958100e-01,1.13807800e+00}],(22[&prob=1.00000000e+00,prob_stddev=0.00000000e+00,prob_range={1.00000000e+00,1.00000000e+00},prob(percent)="100",prob+-sd="100+-0"]:1.350855e-01[&length_mean=1.33810398e-01,length_median=1.35085500e-01,length_95%HPD={6.47298400e-02,2.06373700e-01}],24[&prob=1.00000000e+00,prob_stddev=0.00000000e+00,prob_range={1.00000000e+00,1.00000000e+00},prob(percent)="100",prob+-sd="100+-0"]:1.587950e-01[&length_mean=1.55362870e-01,length_median=1.58795000e-01,length_95%HPD={7.72871900e-02,2.33834500e-01}])[&prob=7.89473684e-01,prob_stddev=1.71414795e-02,prob_range={7.65489674e-01,8.02798135e-01},prob(percent)="79",prob+-sd="79+-2"]:4.975569e-02[&length_mean=5.42581560e-02,length_median=4.97556900e-02,length_95%HPD={9.19046600e-05,1.16471000e-01}])[&prob=8.37941372e-01,prob_stddev=1.38938413e-02,prob_range={8.26115923e-01,8.55429714e-01},prob(percent)="84",prob+-sd="84+-1"]:5.217295e-02[&length_mean=5.71657109e-02,length_median=5.21729500e-02,length_95%HPD={1.06273500e-04,1.18792000e-01}],23[&prob=1.00000000e+00,prob_stddev=0.00000000e+00,prob_range={1.00000000e+00,1.00000000e+00},prob(percent)="100",prob+-sd="100+-0"]:2.163462e-01[&length_mean=2.11097438e-01,length_median=2.16346200e-01,length_95%HPD={1.02019700e-01,3.12949700e-01}])[&prob=9.92338441e-01,prob_stddev=3.10109706e-03,prob_range={9.88674217e-01,9.96002665e-01},prob(percent)="99",prob+-sd="99+-0"]:7.654150e-02[&length_mean=7.73442024e-02,length_median=7.65415000e-02,length_95%HPD={3.24847000e-02,1.26839400e-01}])[&prob=1.00000000e+00,prob_stddev=0.00000000e+00,prob_range={1.00000000e+00,1.00000000e+00},prob(percent)="100",prob+-sd="100+-0"]:9.919165e-02[&length_mean=9.97163458e-02,length_median=9.91916500e-02,length_95%HPD={4.58941200e-02,1.54094100e-01}],(26[&prob=1.00000000e+00,prob_stddev=0.00000000e+00,prob_range={1.00000000e+00,1.00000000e+00},prob(percent)="100",prob+-sd="100+-0"]:2.162270e-01[&length_mean=2.07650590e-01,length_median=2.16227000e-01,length_95%HPD={1.04915000e-01,3.04477700e-01}],51[&prob=1.00000000e+00,prob_stddev=0.00000000e+00,prob_range={1.00000000e+00,1.00000000e+00},prob(percent)="100",prob+-sd="100+-0"]:1.787529e-01[&length_mean=1.74675079e-01,length_median=1.78752900e-01,length_95%HPD={8.82294100e-02,2.60455600e-01}])[&prob=1.00000000e+00,prob_stddev=0.00000000e+00,prob_range={1.00000000e+00,1.00000000e+00},prob(percent)="100",prob+-sd="100+-0"]:1.454466e-01[&length_mean=1.42534688e-01,length_median=1.45446600e-01,length_95%HPD={6.72112000e-02,2.15017300e-01}])[&prob=1.00000000e+00,prob_stddev=0.00000000e+00,prob_range={1.00000000e+00,1.00000000e+00},prob(percent)="100",prob+-sd="100+-0"]:3.724792e-02[&length_mean=3.87896613e-02,length_median=3.72479200e-02,length_95%HPD={1.43116500e-02,6.61718700e-02}])[&prob=1.00000000e+00,prob_stddev=0.00000000e+00,prob_range={1.00000000e+00,1.00000000e+00},prob(percent)="100",prob+-sd="100+-0"]:1.981926e-01[&length_mean=1.94134224e-01,length_median=1.98192600e-01,length_95%HPD={9.05514200e-02,2.87833700e-01}])[&prob=1.00000000e+00,prob_stddev=0.00000000e+00,prob_range={1.00000000e+00,1.00000000e+00},prob(percent)="100",prob+-sd="100+-0"]:2.086620e-01[&length_mean=2.04360888e-01,length_median=2.08662000e-01,length_95%HPD={9.22358200e-02,3.06889900e-01}],((((27[&prob=1.00000000e+00,prob_stddev=0.00000000e+00,prob_range={1.00000000e+00,1.00000000e+00},prob(percent)="100",prob+-sd="100+-0"]:2.816189e-01[&length_mean=2.73686852e-01,length_median=2.81618900e-01,length_95%HPD={1.37587700e-01,4.07199600e-01}],65[&prob=1.00000000e+00,prob_stddev=0.00000000e+00,prob_range={1.00000000e+00,1.00000000e+00},prob(percent)="100",prob+-sd="100+-0"]:3.672037e-01[&length_mean=3.64471555e-01,length_median=3.67203700e-01,length_95%HPD={1.67531900e-01,5.51554900e-01}])[&prob=1.00000000e+00,prob_stddev=0.00000000e+00,prob_range={1.00000000e+00,1.00000000e+00},prob(percent)="100",prob+-sd="100+-0"]:6.209181e-02[&length_mean=6.46845177e-02,length_median=6.20918100e-02,length_95%HPD={2.40823200e-02,1.10318200e-01}],(((54[&prob=1.00000000e+00,prob_stddev=0.00000000e+00,prob_range={1.00000000e+00,1.00000000e+00},prob(percent)="100",prob+-sd="100+-0"]:9.866577e-02[&length_mean=1.00351177e-01,length_median=9.86657700e-02,length_95%HPD={4.02601300e-02,1.64319900e-01}],62[&prob=1.00000000e+00,prob_stddev=0.00000000e+00,prob_range={1.00000000e+00,1.00000000e+00},prob(percent)="100",prob+-sd="100+-0"]:9.456781e-02[&length_mean=9.74311934e-02,length_median=9.45678100e-02,length_95%HPD={3.84857500e-02,1.60845900e-01}])[&prob=1.00000000e+00,prob_stddev=0.00000000e+00,prob_range={1.00000000e+00,1.00000000e+00},prob(percent)="100",prob+-sd="100+-0"]:1.580389e-01[&length_mean=1.59779972e-01,length_median=1.58038900e-01,length_95%HPD={6.94883100e-02,2.54439700e-01}],(((((55[&prob=1.00000000e+00,prob_stddev=0.00000000e+00,prob_range={1.00000000e+00,1.00000000e+00},prob(percent)="100",prob+-sd="100+-0"]:1.992184e-01[&length_mean=1.99272638e-01,length_median=1.99218400e-01,length_95%HPD={8.62889100e-02,3.12616500e-01}],57[&prob=1.00000000e+00,prob_stddev=0.00000000e+00,prob_range={1.00000000e+00,1.00000000e+00},prob(percent)="100",prob+-sd="100+-0"]:2.507610e-01[&length_mean=2.47580159e-01,length_median=2.50761000e-01,length_95%HPD={1.17227900e-01,3.82927300e-01}])[&prob=6.65556296e-01,prob_stddev=2.13676441e-02,prob_range={6.40906063e-01,6.92205197e-01},prob(percent)="67",prob+-sd="67+-2"]:2.357650e-02[&length_mean=2.65327550e-02,length_median=2.35765000e-02,length_95%HPD={3.38207400e-05,5.62381900e-02}],58[&prob=1.00000000e+00,prob_stddev=0.00000000e+00,prob_range={1.00000000e+00,1.00000000e+00},prob(percent)="100",prob+-sd="100+-0"]:1.660390e-01[&length_mean=1.64435468e-01,length_median=1.66039000e-01,length_95%HPD={7.90265800e-02,2.52814800e-01}])[&prob=6.83377748e-01,prob_stddev=1.85458528e-02,prob_range={6.59560293e-01,7.02198534e-01},prob(percent)="68",prob+-sd="68+-2"]:1.419298e-02[&length_mean=1.61377688e-02,length_median=1.41929800e-02,length_95%HPD={2.09871200e-03,3.50383200e-02}],64[&prob=1.00000000e+00,prob_stddev=0.00000000e+00,prob_range={1.00000000e+00,1.00000000e+00},prob(percent)="100",prob+-sd="100+-0"]:2.113001e-01[&length_mean=2.10737689e-01,length_median=2.11300100e-01,length_95%HPD={9.44346300e-02,3.26035200e-01}])[&prob=8.10126582e-01,prob_stddev=9.02069554e-03,prob_range={7.98800799e-01,8.19453698e-01},prob(percent)="81",prob+-sd="81+-1"]:1.785969e-02[&length_mean=2.01659171e-02,length_median=1.78596900e-02,length_95%HPD={2.94560400e-03,4.24704600e-02}],(60[&prob=1.00000000e+00,prob_stddev=0.00000000e+00,prob_range={1.00000000e+00,1.00000000e+00},prob(percent)="100",prob+-sd="100+-0"]:8.192580e-02[&length_mean=8.38760597e-02,length_median=8.19258000e-02,length_95%HPD={3.22680700e-02,1.38102200e-01}],66[&prob=1.00000000e+00,prob_stddev=0.00000000e+00,prob_range={1.00000000e+00,1.00000000e+00},prob(percent)="100",prob+-sd="100+-0"]:1.846917e-01[&length_mean=1.85413757e-01,length_median=1.84691700e-01,length_95%HPD={8.31499400e-02,2.92334700e-01}])[&prob=9.95836109e-01,prob_stddev=9.99333777e-04,prob_range={9.95336442e-01,9.97335110e-01},prob(percent)="100",prob+-sd="100+-0"]:2.955879e-02[&length_mean=3.19183687e-02,length_median=2.95587900e-02,length_95%HPD={9.14155300e-03,6.16436000e-02}])[&prob=1.00000000e+00,prob_stddev=0.00000000e+00,prob_range={1.00000000e+00,1.00000000e+00},prob(percent)="100",prob+-sd="100+-0"]:5.408577e-02[&length_mean=5.68178986e-02,length_median=5.40857700e-02,length_95%HPD={2.01407800e-02,9.94975300e-02}],69[&prob=1.00000000e+00,prob_stddev=0.00000000e+00,prob_range={1.00000000e+00,1.00000000e+00},prob(percent)="100",prob+-sd="100+-0"]:2.790408e-01[&length_mean=2.76034146e-01,length_median=2.79040800e-01,length_95%HPD={1.21940700e-01,4.21734200e-01}])[&prob=1.00000000e+00,prob_stddev=0.00000000e+00,prob_range={1.00000000e+00,1.00000000e+00},prob(percent)="100",prob+-sd="100+-0"]:7.679098e-02[&length_mean=7.94648160e-02,length_median=7.67909800e-02,length_95%HPD={3.13636600e-02,1.31574900e-01}])[&prob=8.39773484e-01,prob_stddev=1.12075975e-02,prob_range={8.29447035e-01,8.55429714e-01},prob(percent)="84",prob+-sd="84+-1"]:1.297803e-02[&length_mean=1.48011629e-02,length_median=1.29780300e-02,length_95%HPD={1.39647200e-03,3.30272200e-02}],(61[&prob=1.00000000e+00,prob_stddev=0.00000000e+00,prob_range={1.00000000e+00,1.00000000e+00},prob(percent)="100",prob+-sd="100+-0"]:3.334156e-01[&length_mean=3.28371570e-01,length_median=3.33415600e-01,length_95%HPD={1.57759700e-01,5.11459500e-01}],((63[&prob=1.00000000e+00,prob_stddev=0.00000000e+00,prob_range={1.00000000e+00,1.00000000e+00},prob(percent)="100",prob+-sd="100+-0"]:2.532228e-01[&length_mean=2.53581575e-01,length_median=2.53222800e-01,length_95%HPD={1.14197100e-01,3.97348700e-01}],71[&prob=1.00000000e+00,prob_stddev=0.00000000e+00,prob_range={1.00000000e+00,1.00000000e+00},prob(percent)="100",prob+-sd="100+-0"]:3.013951e-01[&length_mean=2.99241437e-01,length_median=3.01395100e-01,length_95%HPD={1.33543500e-01,4.60933500e-01}])[&prob=8.70419720e-01,prob_stddev=1.23386137e-02,prob_range={8.54097268e-01,8.84077282e-01},prob(percent)="87",prob+-sd="87+-1"]:3.136233e-02[&length_mean=3.48177272e-02,length_median=3.13623300e-02,length_95%HPD={6.02177100e-03,7.46525400e-02}],68[&prob=1.00000000e+00,prob_stddev=0.00000000e+00,prob_range={1.00000000e+00,1.00000000e+00},prob(percent)="100",prob+-sd="100+-0"]:2.491308e-01[&length_mean=2.44910236e-01,length_median=2.49130800e-01,length_95%HPD={1.18875600e-01,3.66822500e-01}])[&prob=1.00000000e+00,prob_stddev=0.00000000e+00,prob_range={1.00000000e+00,1.00000000e+00},prob(percent)="100",prob+-sd="100+-0"]:4.384039e-02[&length_mean=4.69882645e-02,length_median=4.38403900e-02,length_95%HPD={1.47250000e-02,8.75122000e-02}])[&prob=9.87841439e-01,prob_stddev=3.18929749e-03,prob_range={9.83344437e-01,9.90672885e-01},prob(percent)="99",prob+-sd="99+-0"]:2.706987e-02[&length_mean=2.94699737e-02,length_median=2.70698700e-02,length_95%HPD={6.09402400e-03,5.85580400e-02}])[&prob=1.00000000e+00,prob_stddev=0.00000000e+00,prob_range={1.00000000e+00,1.00000000e+00},prob(percent)="100",prob+-sd="100+-0"]:5.536509e-02[&length_mean=5.67047958e-02,length_median=5.53650900e-02,length_95%HPD={2.53061300e-02,9.37460700e-02}])[&prob=3.54763491e-01,prob_stddev=1.61779168e-02,prob_range={3.35776149e-01,3.70419720e-01},prob(percent)="35",prob+-sd="35+-2"]:1.366916e-02[&length_mean=1.51235657e-02,length_median=1.36691600e-02,length_95%HPD={2.34892700e-03,3.09567600e-02}],(((28[&prob=1.00000000e+00,prob_stddev=0.00000000e+00,prob_range={1.00000000e+00,1.00000000e+00},prob(percent)="100",prob+-sd="100+-0"]:1.993587e-01[&length_mean=1.98046426e-01,length_median=1.99358700e-01,length_95%HPD={9.42397100e-02,3.03642900e-01}],53[&prob=1.00000000e+00,prob_stddev=0.00000000e+00,prob_range={1.00000000e+00,1.00000000e+00},prob(percent)="100",prob+-sd="100+-0"]:2.835636e-01[&length_mean=2.80737496e-01,length_median=2.83563600e-01,length_95%HPD={1.27099800e-01,4.32466800e-01}])[&prob=1.00000000e+00,prob_stddev=0.00000000e+00,prob_range={1.00000000e+00,1.00000000e+00},prob(percent)="100",prob+-sd="100+-0"]:6.493165e-02[&length_mean=6.75334759e-02,length_median=6.49316500e-02,length_95%HPD={2.59075300e-02,1.15707400e-01}],52[&prob=1.00000000e+00,prob_stddev=0.00000000e+00,prob_range={1.00000000e+00,1.00000000e+00},prob(percent)="100",prob+-sd="100+-0"]:2.765063e-01[&length_mean=2.72813567e-01,length_median=2.76506300e-01,length_95%HPD={1.28729700e-01,4.15525700e-01}])[&prob=1.00000000e+00,prob_stddev=0.00000000e+00,prob_range={1.00000000e+00,1.00000000e+00},prob(percent)="100",prob+-sd="100+-0"]:1.413752e-01[&length_mean=1.41152376e-01,length_median=1.41375200e-01,length_95%HPD={6.55623600e-02,2.20873000e-01}],(29[&prob=1.00000000e+00,prob_stddev=0.00000000e+00,prob_range={1.00000000e+00,1.00000000e+00},prob(percent)="100",prob+-sd="100+-0"]:3.274624e-01[&length_mean=3.16787728e-01,length_median=3.27462400e-01,length_95%HPD={1.58647600e-01,4.67454900e-01}],(56[&prob=1.00000000e+00,prob_stddev=0.00000000e+00,prob_range={1.00000000e+00,1.00000000e+00},prob(percent)="100",prob+-sd="100+-0"]:2.093221e-01[&length_mean=2.10376452e-01,length_median=2.09322100e-01,length_95%HPD={9.30297100e-02,3.32890900e-01}],((59[&prob=1.00000000e+00,prob_stddev=0.00000000e+00,prob_range={1.00000000e+00,1.00000000e+00},prob(percent)="100",prob+-sd="100+-0"]:1.631318e-01[&length_mean=1.63850373e-01,length_median=1.63131800e-01,length_95%HPD={8.08311700e-02,2.56666300e-01}],((67[&prob=1.00000000e+00,prob_stddev=0.00000000e+00,prob_range={1.00000000e+00,1.00000000e+00},prob(percent)="100",prob+-sd="100+-0"]:1.562685e-02[&length_mean=1.93485208e-02,length_median=1.56268500e-02,length_95%HPD={4.62169300e-06,4.89765700e-02}],73[&prob=1.00000000e+00,prob_stddev=0.00000000e+00,prob_range={1.00000000e+00,1.00000000e+00},prob(percent)="100",prob+-sd="100+-0"]:1.151182e-01[&length_mean=1.26942871e-01,length_median=1.15118200e-01,length_95%HPD={2.80036500e-02,2.50608500e-01}])[&prob=9.63357761e-01,prob_stddev=4.95578805e-03,prob_range={9.58694204e-01,9.70019987e-01},prob(percent)="96",prob+-sd="96+-0"]:3.623225e-02[&length_mean=4.05293226e-02,length_median=3.62322500e-02,length_95%HPD={4.45462300e-03,8.67657500e-02}],72[&prob=1.00000000e+00,prob_stddev=0.00000000e+00,prob_range={1.00000000e+00,1.00000000e+00},prob(percent)="100",prob+-sd="100+-0"]:1.557470e-01[&length_mean=1.67525374e-01,length_median=1.55747000e-01,length_95%HPD={4.23649100e-02,3.21675500e-01}])[&prob=9.98834111e-01,prob_stddev=8.38311618e-04,prob_range={9.98001332e-01,1.00000000e+00},prob(percent)="100",prob+-sd="100+-0"]:5.054370e-02[&length_mean=5.49478870e-02,length_median=5.05437000e-02,length_95%HPD={1.10325400e-02,1.08825200e-01}])[&prob=9.28547635e-01,prob_stddev=4.15611088e-03,prob_range={9.24716855e-01,9.34043971e-01},prob(percent)="93",prob+-sd="93+-0"]:2.221967e-02[&length_mean=2.44840518e-02,length_median=2.22196700e-02,length_95%HPD={4.97040300e-03,4.75745600e-02}],70[&prob=1.00000000e+00,prob_stddev=0.00000000e+00,prob_range={1.00000000e+00,1.00000000e+00},prob(percent)="100",prob+-sd="100+-0"]:1.885308e-01[&length_mean=1.87609534e-01,length_median=1.88530800e-01,length_95%HPD={8.35766900e-02,2.96312600e-01}])[&prob=8.78580946e-01,prob_stddev=7.54480457e-03,prob_range={8.68087941e-01,8.84077282e-01},prob(percent)="88",prob+-sd="88+-1"]:1.797644e-02[&length_mean=2.04688376e-02,length_median=1.79764400e-02,length_95%HPD={1.80519200e-03,4.58228300e-02}])[&prob=1.00000000e+00,prob_stddev=0.00000000e+00,prob_range={1.00000000e+00,1.00000000e+00},prob(percent)="100",prob+-sd="100+-0"]:1.265329e-01[&length_mean=1.27730751e-01,length_median=1.26532900e-01,length_95%HPD={5.69928300e-02,2.03073500e-01}])[&prob=1.00000000e+00,prob_stddev=0.00000000e+00,prob_range={1.00000000e+00,1.00000000e+00},prob(percent)="100",prob+-sd="100+-0"]:1.342213e-01[&length_mean=1.32552454e-01,length_median=1.34221300e-01,length_95%HPD={6.22675900e-02,2.02015600e-01}])[&prob=7.21685543e-01,prob_stddev=1.56845535e-02,prob_range={6.98201199e-01,7.30846103e-01},prob(percent)="72",prob+-sd="72+-2"]:1.314356e-02[&length_mean=1.42146500e-02,length_median=1.31435600e-02,length_95%HPD={3.17562800e-03,2.72227600e-02}])[&prob=1.00000000e+00,prob_stddev=0.00000000e+00,prob_range={1.00000000e+00,1.00000000e+00},prob(percent)="100",prob+-sd="100+-0"]:1.353399e-01[&length_mean=1.33799477e-01,length_median=1.35339900e-01,length_95%HPD={6.33605700e-02,2.06182300e-01}],((30[&prob=1.00000000e+00,prob_stddev=0.00000000e+00,prob_range={1.00000000e+00,1.00000000e+00},prob(percent)="100",prob+-sd="100+-0"]:6.602682e-02[&length_mean=6.94496745e-02,length_median=6.60268200e-02,length_95%HPD={2.60947400e-02,1.21148200e-01}],(77[&prob=1.00000000e+00,prob_stddev=0.00000000e+00,prob_range={1.00000000e+00,1.00000000e+00},prob(percent)="100",prob+-sd="100+-0"]:1.651777e-01[&length_mean=1.74516510e-01,length_median=1.65177700e-01,length_95%HPD={5.64750900e-02,3.07603800e-01}],78[&prob=1.00000000e+00,prob_stddev=0.00000000e+00,prob_range={1.00000000e+00,1.00000000e+00},prob(percent)="100",prob+-sd="100+-0"]:1.113447e-01[&length_mean=1.16016818e-01,length_median=1.11344700e-01,length_95%HPD={4.09421000e-02,1.97250100e-01}])[&prob=8.85409727e-01,prob_stddev=1.65441533e-02,prob_range={8.60759494e-01,8.96069287e-01},prob(percent)="89",prob+-sd="89+-2"]:2.855164e-02[&length_mean=3.19588151e-02,length_median=2.85516400e-02,length_95%HPD={5.15899400e-03,6.84901400e-02}])[&prob=8.88407728e-01,prob_stddev=1.48922218e-02,prob_range={8.66089274e-01,8.96735510e-01},prob(percent)="89",prob+-sd="89+-1"]:1.390088e-01[&length_mean=1.38211968e-01,length_median=1.39008800e-01,length_95%HPD={6.16460600e-02,2.17922500e-01}],(74[&prob=1.00000000e+00,prob_stddev=0.00000000e+00,prob_range={1.00000000e+00,1.00000000e+00},prob(percent)="100",prob+-sd="100+-0"]:1.656181e-01[&length_mean=1.64352641e-01,length_median=1.65618100e-01,length_95%HPD={7.56971200e-02,2.50572100e-01}],(75[&prob=1.00000000e+00,prob_stddev=0.00000000e+00,prob_range={1.00000000e+00,1.00000000e+00},prob(percent)="100",prob+-sd="100+-0"]:1.037482e-01[&length_mean=1.10475963e-01,length_median=1.03748200e-01,length_95%HPD={2.74850800e-02,2.06018700e-01}],76[&prob=1.00000000e+00,prob_stddev=0.00000000e+00,prob_range={1.00000000e+00,1.00000000e+00},prob(percent)="100",prob+-sd="100+-0"]:4.693580e-02[&length_mean=5.56147040e-02,length_median=4.69358000e-02,length_95%HPD={2.85339100e-03,1.29504400e-01}])[&prob=8.74250500e-01,prob_stddev=1.34611575e-02,prob_range={8.54097268e-01,8.82078614e-01},prob(percent)="87",prob+-sd="87+-1"]:6.508963e-02[&length_mean=7.16966151e-02,length_median=6.50896300e-02,length_95%HPD={1.11274900e-02,1.46250900e-01}])[&prob=6.24583611e-01,prob_stddev=1.54001674e-02,prob_range={6.12924717e-01,6.46235843e-01},prob(percent)="62",prob+-sd="62+-2"]:1.791515e-02[&length_mean=2.08355762e-02,length_median=1.79151500e-02,length_95%HPD={1.28758400e-06,4.69483600e-02}])[&prob=1.00000000e+00,prob_stddev=0.00000000e+00,prob_range={1.00000000e+00,1.00000000e+00},prob(percent)="100",prob+-sd="100+-0"]:3.606133e-01[&length_mean=3.48122177e-01,length_median=3.60613300e-01,length_95%HPD={1.70546500e-01,5.15748300e-01}])[&prob=1.00000000e+00,prob_stddev=0.00000000e+00,prob_range={1.00000000e+00,1.00000000e+00},prob(percent)="100",prob+-sd="100+-0"]:1.380306e-01[&length_mean=1.36828639e-01,length_median=1.38030600e-01,length_95%HPD={6.34737100e-02,2.11677800e-01}])[&prob=1.00000000e+00,prob_stddev=0.00000000e+00,prob_range={1.00000000e+00,1.00000000e+00},prob(percent)="100",prob+-sd="100+-0"]:3.617378e-01[&length_mean=3.55560988e-01,length_median=3.61737800e-01,length_95%HPD={1.64912900e-01,5.40531300e-01}])[&prob=9.95336442e-01,prob_stddev=5.43968408e-04,prob_range={9.94670220e-01,9.96002665e-01},prob(percent)="100",prob+-sd="100+-0"]:1.724535e-01[&length_mean=1.82637107e-01,length_median=1.72453500e-01,length_95%HPD={5.63535800e-02,3.12835200e-01}])[&prob=1.00000000e+00,prob_stddev=0.00000000e+00,prob_range={1.00000000e+00,1.00000000e+00},prob(percent)="100",prob+-sd="100+-0"]:8.708231e-01[&length_mean=8.43244776e-01,length_median=8.70823100e-01,length_95%HPD={3.79435700e-01,1.29858500e+00}])[&prob=1.00000000e+00,prob_stddev=0.00000000e+00,prob_range={1.00000000e+00,1.00000000e+00},prob(percent)="100",prob+-sd="100+-0"]:1.530441e-01[&length_mean=1.58176103e-01,length_median=1.53044100e-01,length_95%HPD={6.37211600e-02,2.71616400e-01}])[&prob=1.00000000e+00,prob_stddev=0.00000000e+00,prob_range={1.00000000e+00,1.00000000e+00},prob(percent)="100",prob+-sd="100+-0"]:1.237897e+00[&length_mean=1.19210664e+00,length_median=1.23789700e+00,length_95%HPD={5.46687500e-01,1.78685200e+00}],(2[&prob=1.00000000e+00,prob_stddev=0.00000000e+00,prob_range={1.00000000e+00,1.00000000e+00},prob(percent)="100",prob+-sd="100+-0"]:1.045722e+00[&length_mean=1.01192148e+00,length_median=1.04572200e+00,length_95%HPD={4.74421300e-01,1.49702900e+00}],3[&prob=1.00000000e+00,prob_stddev=0.00000000e+00,prob_range={1.00000000e+00,1.00000000e+00},prob(percent)="100",prob+-sd="100+-0"]:7.550666e-01[&length_mean=7.31425427e-01,length_median=7.55066600e-01,length_95%HPD={3.31267100e-01,1.09655400e+00}])[&prob=1.00000000e+00,prob_stddev=0.00000000e+00,prob_range={1.00000000e+00,1.00000000e+00},prob(percent)="100",prob+-sd="100+-0"]:2.880746e-01[&length_mean=2.88041672e-01,length_median=2.88074600e-01,length_95%HPD={1.22517600e-01,4.60757300e-01}]);

end;

**Nuclear genes – undated Bayesian analysis (majority rule consensus with all compatible clades)**

***
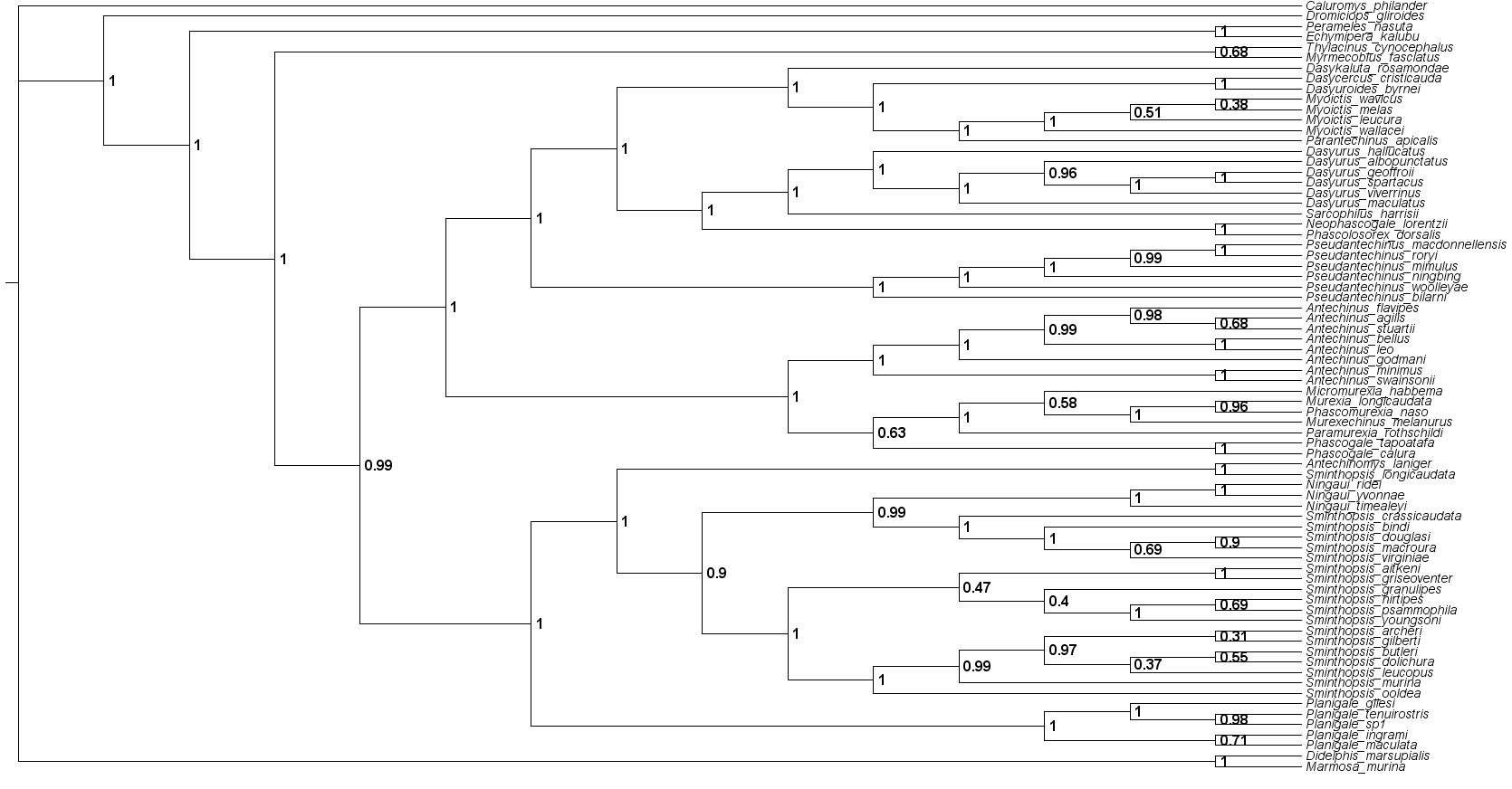
***

#NEXUS

begin taxa;

dimensions ntax=74;

taxlabels

Caluromys_philander

Didelphis_marsupialis

Marmosa_murina

Dromiciops_gliroides

Perameles_nasuta

Echymipera_kalubu

Thylacinus_cynocephalus

Myrmecobius_fasciatus

Dasykaluta_rosamondae

Dasycercus_cristicauda

Dasyuroides_byrnei

Dasyurus_hallucatus

Myoictis_wavicus

Neophascogale_lorentzii

Phascolosorex_dorsalis

Parantechinus_apicalis

Pseudantechinus_macdonnellensis

Sarcophilus_harrisii

Antechinus_flavipes

Micromurexia_habbema

Murexia_longicaudata

Murexechinus_melanurus

Phascomurexia_naso

Paramurexia_rothschildi

Phascogale_tapoatafa

Antechinomys_laniger

Ningaui_ridei

Sminthopsis_crassicaudata

Planigale_gilesi

Dasyurus_albopunctatus

Dasyurus_geoffroii

Dasyurus_maculatus

Dasyurus_spartacus

Dasyurus_viverrinus

Myoictis_leucura

Myoictis_melas

Myoictis_wallacei

Pseudantechinus_bilarni

Pseudantechinus_mimulus

Pseudantechinus_ningbing

Pseudantechinus_roryi

Pseudantechinus_woolleyae

Antechinus_agilis

Antechinus_bellus

Antechinus_godmani

Antechinus_leo

Antechinus_minimus

Antechinus_stuartii

Antechinus_swainsonii

Phascogale_calura

Ningaui_timealeyi

Ningaui_yvonnae

Sminthopsis_aitkeni

Sminthopsis_archeri

Sminthopsis_bindi

Sminthopsis_butleri

Sminthopsis_dolichura

Sminthopsis_douglasi

Sminthopsis_gilberti

Sminthopsis_granulipes

Sminthopsis_griseoventer

Sminthopsis_hirtipes

Sminthopsis_leucopus

Sminthopsis_longicaudata

Sminthopsis_murina

Sminthopsis_macroura

Sminthopsis_psammophila

Sminthopsis_ooldea

Sminthopsis_virginiae

Sminthopsis_youngsoni

Planigale_ingrami

Planigale_maculata

Planigale_tenuirostris

Planigale_sp1

;

end;

begin trees;

translate

1 Caluromys_philander,

2 Didelphis_marsupialis,

3 Marmosa_murina,

4 Dromiciops_gliroides,

5 Perameles_nasuta,

6 Echymipera_kalubu,

7 Thylacinus_cynocephalus,

8 Myrmecobius_fasciatus,

9 Dasykaluta_rosamondae,

10 Dasycercus_cristicauda,

11 Dasyuroides_byrnei,

12 Dasyurus_hallucatus,

13 Myoictis_wavicus,

14 Neophascogale_lorentzii,

15 Phascolosorex_dorsalis,

16 Parantechinus_apicalis,

17 Pseudantechinus_macdonnellensis,

18 Sarcophilus_harrisii,

19 Antechinus_flavipes,

20 Micromurexia_habbema,

21 Murexia_longicaudata,

22 Murexechinus_melanurus,

23 Phascomurexia_naso,

24 Paramurexia_rothschildi,

25 Phascogale_tapoatafa,

26 Antechinomys_laniger,

27 Ningaui_ridei,

28 Sminthopsis_crassicaudata,

29 Planigale_gilesi,

30 Dasyurus_albopunctatus,

31 Dasyurus_geoffroii,

32 Dasyurus_maculatus,

33 Dasyurus_spartacus,

34 Dasyurus_viverrinus,

35 Myoictis_leucura,

36 Myoictis_melas,

37 Myoictis_wallacei,

38 Pseudantechinus_bilarni,

39 Pseudantechinus_mimulus,

40 Pseudantechinus_ningbing,

41 Pseudantechinus_roryi,

42 Pseudantechinus_woolleyae,

43 Antechinus_agilis,

44 Antechinus_bellus,

45 Antechinus_godmani,

46 Antechinus_leo,

47 Antechinus_minimus,

48 Antechinus_stuartii,

49 Antechinus_swainsonii,

50 Phascogale_calura,

51 Ningaui_timealeyi,

52 Ningaui_yvonnae,

53 Sminthopsis_aitkeni,

54 Sminthopsis_archeri,

55 Sminthopsis_bindi,

56 Sminthopsis_butleri,

57 Sminthopsis_dolichura,

58 Sminthopsis_douglasi,

59 Sminthopsis_gilberti,

60 Sminthopsis_granulipes,

61 Sminthopsis_griseoventer,

62 Sminthopsis_hirtipes,

63 Sminthopsis_leucopus,

64 Sminthopsis_longicaudata,

65 Sminthopsis_murina,

66 Sminthopsis_macroura,

67 Sminthopsis_psammophila,

68 Sminthopsis_ooldea,

69 Sminthopsis_virginiae,

70 Sminthopsis_youngsoni,

71 Planigale_ingrami,

72 Planigale_maculata,

73 Planigale_tenuirostris,

74 Planigale_sp1

;

tree con_all_compat = [&U] (1[&prob=1.00000000e+00,prob_stddev=0.00000000e+00,prob_range={1.00000000e+00,1.00000000e+00},prob(percent)="100",prob+-sd="100+-0"]:3.722270e-02[&length_mean=3.72862050e-02,length_median=3.72227000e-02,length_95%HPD={2.98686900e-02,4.44611400e-02}],(4[&prob=1.00000000e+00,prob_stddev=0.00000000e+00,prob_range={1.00000000e+00,1.00000000e+00},prob(percent)="100",prob+-sd="100+-0"]:8.196875e-02[&length_mean=8.21130720e-02,length_median=8.19687500e-02,length_95%HPD={7.26267300e-02,9.25251800e-02}],((5[&prob=1.00000000e+00,prob_stddev=0.00000000e+00,prob_range={1.00000000e+00,1.00000000e+00},prob(percent)="100",prob+-sd="100+-0"]:1.736714e-02[&length_mean=1.74441106e-02,length_median=1.73671400e-02,length_95%HPD={1.32932300e-02,2.18517300e-02}],6[&prob=1.00000000e+00,prob_stddev=0.00000000e+00,prob_range={1.00000000e+00,1.00000000e+00},prob(percent)="100",prob+-sd="100+-0"]:1.782497e-02[&length_mean=1.79127596e-02,length_median=1.78249700e-02,length_95%HPD={1.38385200e-02,2.22590200e-02}])[&prob=1.00000000e+00,prob_stddev=0.00000000e+00,prob_range={1.00000000e+00,1.00000000e+00},prob(percent)="100",prob+-sd="100+-0"]:9.807617e-02[&length_mean=9.81675759e-02,length_median=9.80761700e-02,length_95%HPD={8.74134700e-02,1.10400500e-01}],((7[&prob=1.00000000e+00,prob_stddev=0.00000000e+00,prob_range={1.00000000e+00,1.00000000e+00},prob(percent)="100",prob+-sd="100+-0"]:4.509136e-02[&length_mean=4.58942960e-02,length_median=4.50913600e-02,length_95%HPD={2.75142100e-02,6.62453200e-02}],8[&prob=1.00000000e+00,prob_stddev=0.00000000e+00,prob_range={1.00000000e+00,1.00000000e+00},prob(percent)="100",prob+-sd="100+-0"]:5.459709e-02[&length_mean=5.33808778e-02,length_median=5.45970900e-02,length_95%HPD={3.48128300e-02,6.78330500e-02}])[&prob=6.76715523e-01,prob_stddev=9.66022652e-03,prob_range={6.66888741e-01,6.88207861e-01},prob(percent)="68",prob+-sd="68+-1"]:1.223263e-02[&length_mean=1.32160227e-02,length_median=1.22326300e-02,length_95%HPD={1.83714700e-04,2.76859800e-02}],(((((9[&prob=1.00000000e+00,prob_stddev=0.00000000e+00,prob_range={1.00000000e+00,1.00000000e+00},prob(percent)="100",prob+-sd="100+-0"]:1.458587e-02[&length_mean=1.46598218e-02,length_median=1.45858700e-02,length_95%HPD={1.17250700e-02,1.75814100e-02}],((10[&prob=1.00000000e+00,prob_stddev=0.00000000e+00,prob_range={1.00000000e+00,1.00000000e+00},prob(percent)="100",prob+-sd="100+-0"]:8.550188e-03[&length_mean=8.62105621e-03,length_median=8.55018800e-03,length_95%HPD={6.01135700e-03,1.12117800e-02}],11[&prob=1.00000000e+00,prob_stddev=0.00000000e+00,prob_range={1.00000000e+00,1.00000000e+00},prob(percent)="100",prob+-sd="100+-0"]:8.205122e-03[&length_mean=8.25293491e-03,length_median=8.20512200e-03,length_95%HPD={6.03887600e-03,1.04901700e-02}])[&prob=1.00000000e+00,prob_stddev=0.00000000e+00,prob_range={1.00000000e+00,1.00000000e+00},prob(percent)="100",prob+-sd="100+-0"]:3.213749e-03[&length_mean=3.27663772e-03,length_median=3.21374900e-03,length_95%HPD={1.77341800e-03,5.05785200e-03}],((((13[&prob=1.00000000e+00,prob_stddev=0.00000000e+00,prob_range={1.00000000e+00,1.00000000e+00},prob(percent)="100",prob+-sd="100+-0"]:2.253316e-03[&length_mean=2.69699918e-03,length_median=2.25331600e-03,length_95%HPD={2.22279000e-06,6.74363800e-03}],36[&prob=1.00000000e+00,prob_stddev=0.00000000e+00,prob_range={1.00000000e+00,1.00000000e+00},prob(percent)="100",prob+-sd="100+-0"]:2.762869e-03[&length_mean=3.04976970e-03,length_median=2.76286900e-03,length_95%HPD={7.52496900e-07,6.65473100e-03}])[&prob=3.80079947e-01,prob_stddev=1.50405052e-02,prob_range={3.57761492e-01,3.90406396e-01},prob(percent)="38",prob+-sd="38+-2"]:2.407732e-03[&length_mean=2.60880315e-03,length_median=2.40773200e-03,length_95%HPD={4.48005600e-06,5.66370800e-03}],35[&prob=1.00000000e+00,prob_stddev=0.00000000e+00,prob_range={1.00000000e+00,1.00000000e+00},prob(percent)="100",prob+-sd="100+-0"]:4.850680e-03[&length_mean=5.76328984e-03,length_median=4.85068000e-03,length_95%HPD={4.72715900e-05,1.36497200e-02}])[&prob=5.07161892e-01,prob_stddev=8.97753899e-03,prob_range={4.95003331e-01,5.16322452e-01},prob(percent)="51",prob+-sd="51+-1"]:2.277802e-03[&length_mean=2.46692217e-03,length_median=2.27780200e-03,length_95%HPD={3.22215200e-06,5.27543800e-03}],37[&prob=1.00000000e+00,prob_stddev=0.00000000e+00,prob_range={1.00000000e+00,1.00000000e+00},prob(percent)="100",prob+-sd="100+-0"]:7.020641e-03[&length_mean=7.00156656e-03,length_median=7.02064100e-03,length_95%HPD={3.59591600e-03,1.07747000e-02}])[&prob=1.00000000e+00,prob_stddev=0.00000000e+00,prob_range={1.00000000e+00,1.00000000e+00},prob(percent)="100",prob+-sd="100+-0"]:6.713992e-03[&length_mean=6.78981426e-03,length_median=6.71399200e-03,length_95%HPD={4.06703300e-03,9.52600500e-03}],16[&prob=1.00000000e+00,prob_stddev=0.00000000e+00,prob_range={1.00000000e+00,1.00000000e+00},prob(percent)="100",prob+-sd="100+-0"]:1.230225e-02[&length_mean=1.23742986e-02,length_median=1.23022500e-02,length_95%HPD={9.25026800e-03,1.58250600e-02}])[&prob=9.99833444e-01,prob_stddev=3.33111259e-04,prob_range={9.99333777e-01,1.00000000e+00},prob(percent)="100",prob+-sd="100+-0"]:2.018610e-03[&length_mean=2.09828516e-03,length_median=2.01861000e-03,length_95%HPD={8.16414600e-04,3.55161700e-03}])[&prob=9.99666889e-01,prob_stddev=3.84643750e-04,prob_range={9.99333777e-01,1.00000000e+00},prob(percent)="100",prob+-sd="100+-0"]:1.277231e-03[&length_mean=1.32960571e-03,length_median=1.27723100e-03,length_95%HPD={4.25911000e-04,2.30699900e-03}])[&prob=9.99833444e-01,prob_stddev=3.33111259e-04,prob_range={9.99333777e-01,1.00000000e+00},prob(percent)="100",prob+-sd="100+-0"]:9.412779e-04[&length_mean=1.00286935e-03,length_median=9.41277900e-04,length_95%HPD={2.67230500e-04,1.82579000e-03}],(((12[&prob=1.00000000e+00,prob_stddev=0.00000000e+00,prob_range={1.00000000e+00,1.00000000e+00},prob(percent)="100",prob+-sd="100+-0"]:1.426872e-02[&length_mean=1.43393494e-02,length_median=1.42687200e-02,length_95%HPD={1.10449900e-02,1.76167900e-02}],((30[&prob=1.00000000e+00,prob_stddev=0.00000000e+00,prob_range={1.00000000e+00,1.00000000e+00},prob(percent)="100",prob+-sd="100+-0"]:5.640269e-03[&length_mean=5.68878597e-03,length_median=5.64026900e-03,length_95%HPD={3.71180300e-03,7.69574200e-03}],((31[&prob=1.00000000e+00,prob_stddev=0.00000000e+00,prob_range={1.00000000e+00,1.00000000e+00},prob(percent)="100",prob+-sd="100+-0"]:2.378129e-03[&length_mean=2.46898300e-03,length_median=2.37812900e-03,length_95%HPD={9.99498800e-04,4.07061500e-03}],33[&prob=1.00000000e+00,prob_stddev=0.00000000e+00,prob_range={1.00000000e+00,1.00000000e+00},prob(percent)="100",prob+-sd="100+-0"]:3.702609e-03[&length_mean=3.79347597e-03,length_median=3.70260900e-03,length_95%HPD={1.87545700e-03,5.76626900e-03}])[&prob=1.00000000e+00,prob_stddev=0.00000000e+00,prob_range={1.00000000e+00,1.00000000e+00},prob(percent)="100",prob+-sd="100+-0"]:1.729778e-03[&length_mean=1.82525073e-03,length_median=1.72977800e-03,length_95%HPD={5.06295300e-04,3.32801200e-03}],34[&prob=1.00000000e+00,prob_stddev=0.00000000e+00,prob_range={1.00000000e+00,1.00000000e+00},prob(percent)="100",prob+-sd="100+-0"]:4.927413e-03[&length_mean=5.02976682e-03,length_median=4.92741300e-03,length_95%HPD={2.84311100e-03,7.32909300e-03}])[&prob=9.98334444e-01,prob_stddev=1.15393125e-03,prob_range={9.97335110e-01,9.99333777e-01},prob(percent)="100",prob+-sd="100+-0"]:1.707015e-03[&length_mean=1.80352574e-03,length_median=1.70701500e-03,length_95%HPD={5.65042000e-04,3.21182400e-03}])[&prob=9.64190540e-01,prob_stddev=6.04209099e-03,prob_range={9.56695536e-01,9.71352432e-01},prob(percent)="96",prob+-sd="96+-1"]:8.975074e-04[&length_mean=9.86598244e-04,length_median=8.97507400e-04,length_95%HPD={9.41560800e-05,2.01082000e-03}],32[&prob=1.00000000e+00,prob_stddev=0.00000000e+00,prob_range={1.00000000e+00,1.00000000e+00},prob(percent)="100",prob+-sd="100+-0"]:7.423898e-03[&length_mean=7.49879303e-03,length_median=7.42389800e-03,length_95%HPD={4.86720200e-03,1.01754600e-02}])[&prob=1.00000000e+00,prob_stddev=0.00000000e+00,prob_range={1.00000000e+00,1.00000000e+00},prob(percent)="100",prob+-sd="100+-0"]:3.464085e-03[&length_mean=3.51836629e-03,length_median=3.46408500e-03,length_95%HPD={1.83092100e-03,5.20709300e-03}])[&prob=1.00000000e+00,prob_stddev=0.00000000e+00,prob_range={1.00000000e+00,1.00000000e+00},prob(percent)="100",prob+-sd="100+-0"]:2.241609e-03[&length_mean=2.28202047e-03,length_median=2.24160900e-03,length_95%HPD={1.11728500e-03,3.59781400e-03}],18[&prob=1.00000000e+00,prob_stddev=0.00000000e+00,prob_range={1.00000000e+00,1.00000000e+00},prob(percent)="100",prob+-sd="100+-0"]:1.286650e-02[&length_mean=1.29251710e-02,length_median=1.28665000e-02,length_95%HPD={1.04625900e-02,1.57233700e-02}])[&prob=1.00000000e+00,prob_stddev=0.00000000e+00,prob_range={1.00000000e+00,1.00000000e+00},prob(percent)="100",prob+-sd="100+-0"]:3.323932e-03[&length_mean=3.37984568e-03,length_median=3.32393200e-03,length_95%HPD={2.08972400e-03,4.92236800e-03}],(14[&prob=1.00000000e+00,prob_stddev=0.00000000e+00,prob_range={1.00000000e+00,1.00000000e+00},prob(percent)="100",prob+-sd="100+-0"]:5.866570e-03[&length_mean=5.90948559e-03,length_median=5.86657000e-03,length_95%HPD={4.20454500e-03,7.67633800e-03}],15[&prob=1.00000000e+00,prob_stddev=0.00000000e+00,prob_range={1.00000000e+00,1.00000000e+00},prob(percent)="100",prob+-sd="100+-0"]:8.152887e-03[&length_mean=8.18290100e-03,length_median=8.15288700e-03,length_95%HPD={6.32860600e-03,1.04033000e-02}])[&prob=1.00000000e+00,prob_stddev=0.00000000e+00,prob_range={1.00000000e+00,1.00000000e+00},prob(percent)="100",prob+-sd="100+-0"]:6.405673e-03[&length_mean=6.45052262e-03,length_median=6.40567300e-03,length_95%HPD={4.61718700e-03,8.33223500e-03}])[&prob=1.00000000e+00,prob_stddev=0.00000000e+00,prob_range={1.00000000e+00,1.00000000e+00},prob(percent)="100",prob+-sd="100+-0"]:2.533264e-03[&length_mean=2.56985561e-03,length_median=2.53326400e-03,length_95%HPD={1.45456800e-03,3.81186700e-03}])[&prob=1.00000000e+00,prob_stddev=0.00000000e+00,prob_range={1.00000000e+00,1.00000000e+00},prob(percent)="100",prob+-sd="100+-0"]:1.793670e-03[&length_mean=1.83003193e-03,length_median=1.79367000e-03,length_95%HPD={8.13820300e-04,2.83796300e-03}],(((((17[&prob=1.00000000e+00,prob_stddev=0.00000000e+00,prob_range={1.00000000e+00,1.00000000e+00},prob(percent)="100",prob+-sd="100+-0"]:3.835334e-03[&length_mean=3.93116056e-03,length_median=3.83533400e-03,length_95%HPD={1.98313200e-03,5.94960300e-03}],41[&prob=1.00000000e+00,prob_stddev=0.00000000e+00,prob_range={1.00000000e+00,1.00000000e+00},prob(percent)="100",prob+-sd="100+-0"]:5.460713e-03[&length_mean=5.51682278e-03,length_median=5.46071300e-03,length_95%HPD={3.44926700e-03,7.73978900e-03}])[&prob=1.00000000e+00,prob_stddev=0.00000000e+00,prob_range={1.00000000e+00,1.00000000e+00},prob(percent)="100",prob+-sd="100+-0"]:1.687661e-03[&length_mean=1.76369278e-03,length_median=1.68766100e-03,length_95%HPD={5.73577100e-04,3.11406500e-03}],39[&prob=1.00000000e+00,prob_stddev=0.00000000e+00,prob_range={1.00000000e+00,1.00000000e+00},prob(percent)="100",prob+-sd="100+-0"]:4.175070e-03[&length_mean=4.28685477e-03,length_median=4.17507000e-03,length_95%HPD={2.22280400e-03,6.74650000e-03}])[&prob=9.88674217e-01,prob_stddev=2.60878084e-03,prob_range={9.85343105e-01,9.91339107e-01},prob(percent)="99",prob+-sd="99+-0"]:1.089109e-03[&length_mean=1.16418288e-03,length_median=1.08910900e-03,length_95%HPD={2.31490700e-04,2.20269600e-03}],40[&prob=1.00000000e+00,prob_stddev=0.00000000e+00,prob_range={1.00000000e+00,1.00000000e+00},prob(percent)="100",prob+-sd="100+-0"]:4.245983e-03[&length_mean=4.31538554e-03,length_median=4.24598300e-03,length_95%HPD={2.27218200e-03,6.46285400e-03}])[&prob=1.00000000e+00,prob_stddev=0.00000000e+00,prob_range={1.00000000e+00,1.00000000e+00},prob(percent)="100",prob+-sd="100+-0"]:2.161298e-03[&length_mean=2.23402364e-03,length_median=2.16129800e-03,length_95%HPD={9.46134900e-04,3.72045100e-03}],42[&prob=1.00000000e+00,prob_stddev=0.00000000e+00,prob_range={1.00000000e+00,1.00000000e+00},prob(percent)="100",prob+-sd="100+-0"]:8.349009e-03[&length_mean=8.45488399e-03,length_median=8.34900900e-03,length_95%HPD={5.66153500e-03,1.16064800e-02}])[&prob=1.00000000e+00,prob_stddev=0.00000000e+00,prob_range={1.00000000e+00,1.00000000e+00},prob(percent)="100",prob+-sd="100+-0"]:2.009498e-03[&length_mean=2.08097095e-03,length_median=2.00949800e-03,length_95%HPD={8.09669000e-04,3.50126700e-03}],38[&prob=1.00000000e+00,prob_stddev=0.00000000e+00,prob_range={1.00000000e+00,1.00000000e+00},prob(percent)="100",prob+-sd="100+-0"]:1.075233e-02[&length_mean=1.07917519e-02,length_median=1.07523300e-02,length_95%HPD={8.37563200e-03,1.30936100e-02}])[&prob=9.99000666e-01,prob_stddev=8.60089573e-04,prob_range={9.98001332e-01,1.00000000e+00},prob(percent)="100",prob+-sd="100+-0"]:1.059353e-03[&length_mean=1.11108899e-03,length_median=1.05935300e-03,length_95%HPD={2.54231700e-04,1.99760100e-03}])[&prob=1.00000000e+00,prob_stddev=0.00000000e+00,prob_range={1.00000000e+00,1.00000000e+00},prob(percent)="100",prob+-sd="100+-0"]:1.144298e-02[&length_mean=1.14690666e-02,length_median=1.14429800e-02,length_95%HPD={8.80953500e-03,1.40115900e-02}],(((((19[&prob=1.00000000e+00,prob_stddev=0.00000000e+00,prob_range={1.00000000e+00,1.00000000e+00},prob(percent)="100",prob+-sd="100+-0"]:4.672786e-03[&length_mean=4.74550200e-03,length_median=4.67278600e-03,length_95%HPD={2.66446300e-03,6.76314600e-03}],(43[&prob=1.00000000e+00,prob_stddev=0.00000000e+00,prob_range={1.00000000e+00,1.00000000e+00},prob(percent)="100",prob+-sd="100+-0"]:6.380477e-03[&length_mean=6.46290006e-03,length_median=6.38047700e-03,length_95%HPD={4.07525000e-03,9.09344300e-03}],48[&prob=1.00000000e+00,prob_stddev=0.00000000e+00,prob_range={1.00000000e+00,1.00000000e+00},prob(percent)="100",prob+-sd="100+-0"]:3.130171e-03[&length_mean=3.23069762e-03,length_median=3.13017100e-03,length_95%HPD={1.48072000e-03,5.28126100e-03}])[&prob=6.77215190e-01,prob_stddev=9.81407053e-03,prob_range={6.65556296e-01,6.89540306e-01},prob(percent)="68",prob+-sd="68+-1"]:6.055966e-04[&length_mean=7.09656319e-04,length_median=6.05596600e-04,length_95%HPD={1.32356800e-06,1.66267800e-03}])[&prob=9.75849434e-01,prob_stddev=5.19980878e-03,prob_range={9.71352432e-01,9.83344437e-01},prob(percent)="98",prob+-sd="98+-1"]:6.825585e-04[&length_mean=7.64798930e-04,length_median=6.82558500e-04,length_95%HPD={3.58691100e-05,1.67082800e-03}],(44[&prob=1.00000000e+00,prob_stddev=0.00000000e+00,prob_range={1.00000000e+00,1.00000000e+00},prob(percent)="100",prob+-sd="100+-0"]:4.780645e-03[&length_mean=4.88498607e-03,length_median=4.78064500e-03,length_95%HPD={2.79495900e-03,7.35971300e-03}],46[&prob=1.00000000e+00,prob_stddev=0.00000000e+00,prob_range={1.00000000e+00,1.00000000e+00},prob(percent)="100",prob+-sd="100+-0"]:4.283120e-03[&length_mean=4.39690099e-03,length_median=4.28312000e-03,length_95%HPD={2.32013700e-03,6.75670100e-03}])[&prob=1.00000000e+00,prob_stddev=0.00000000e+00,prob_range={1.00000000e+00,1.00000000e+00},prob(percent)="100",prob+-sd="100+-0"]:4.112113e-03[&length_mean=4.20387359e-03,length_median=4.11211300e-03,length_95%HPD={2.07305500e-03,6.34419000e-03}])[&prob=9.93337775e-01,prob_stddev=3.12486060e-03,prob_range={9.90006662e-01,9.97335110e-01},prob(percent)="99",prob+-sd="99+-0"]:1.286585e-03[&length_mean=1.36508969e-03,length_median=1.28658500e-03,length_95%HPD={2.87767600e-04,2.59425200e-03}],45[&prob=1.00000000e+00,prob_stddev=0.00000000e+00,prob_range={1.00000000e+00,1.00000000e+00},prob(percent)="100",prob+-sd="100+-0"]:8.988453e-03[&length_mean=9.08701181e-03,length_median=8.98845300e-03,length_95%HPD={6.03015300e-03,1.23016400e-02}])[&prob=1.00000000e+00,prob_stddev=0.00000000e+00,prob_range={1.00000000e+00,1.00000000e+00},prob(percent)="100",prob+-sd="100+-0"]:4.509041e-03[&length_mean=4.58210216e-03,length_median=4.50904100e-03,length_95%HPD={2.64229200e-03,6.52984500e-03}],(47[&prob=1.00000000e+00,prob_stddev=0.00000000e+00,prob_range={1.00000000e+00,1.00000000e+00},prob(percent)="100",prob+-sd="100+-0"]:9.104333e-03[&length_mean=9.13627730e-03,length_median=9.10433300e-03,length_95%HPD={6.40353700e-03,1.19275400e-02}],49[&prob=1.00000000e+00,prob_stddev=0.00000000e+00,prob_range={1.00000000e+00,1.00000000e+00},prob(percent)="100",prob+-sd="100+-0"]:5.879518e-03[&length_mean=6.00149994e-03,length_median=5.87951800e-03,length_95%HPD={3.52111500e-03,8.55601700e-03}])[&prob=1.00000000e+00,prob_stddev=0.00000000e+00,prob_range={1.00000000e+00,1.00000000e+00},prob(percent)="100",prob+-sd="100+-0"]:3.944902e-03[&length_mean=4.02842207e-03,length_median=3.94490200e-03,length_95%HPD={2.08157600e-03,6.01405500e-03}])[&prob=1.00000000e+00,prob_stddev=0.00000000e+00,prob_range={1.00000000e+00,1.00000000e+00},prob(percent)="100",prob+-sd="100+-0"]:4.031388e-03[&length_mean=4.08072383e-03,length_median=4.03138800e-03,length_95%HPD={2.42577500e-03,5.78711800e-03}],(((20[&prob=1.00000000e+00,prob_stddev=0.00000000e+00,prob_range={1.00000000e+00,1.00000000e+00},prob(percent)="100",prob+-sd="100+-0"]:1.048076e-02[&length_mean=1.05995342e-02,length_median=1.04807600e-02,length_95%HPD={7.47789600e-03,1.41450000e-02}],((21[&prob=1.00000000e+00,prob_stddev=0.00000000e+00,prob_range={1.00000000e+00,1.00000000e+00},prob(percent)="100",prob+-sd="100+-0"]:3.577028e-03[&length_mean=3.65799704e-03,length_median=3.57702800e-03,length_95%HPD={1.88694400e-03,5.62114900e-03}],23[&prob=1.00000000e+00,prob_stddev=0.00000000e+00,prob_range={1.00000000e+00,1.00000000e+00},prob(percent)="100",prob+-sd="100+-0"]:6.983263e-03[&length_mean=7.08661608e-03,length_median=6.98326300e-03,length_95%HPD={4.63057600e-03,1.00453400e-02}])[&prob=9.64357095e-01,prob_stddev=5.66615626e-03,prob_range={9.56695536e-01,9.70019987e-01},prob(percent)="96",prob+-sd="96+-1"]:7.646705e-04[&length_mean=8.61548924e-04,length_median=7.64670500e-04,length_95%HPD={7.05366600e-05,1.85756100e-03}],22[&prob=1.00000000e+00,prob_stddev=0.00000000e+00,prob_range={1.00000000e+00,1.00000000e+00},prob(percent)="100",prob+-sd="100+-0"]:8.547103e-03[&length_mean=8.61532702e-03,length_median=8.54710300e-03,length_95%HPD={5.52807300e-03,1.16647100e-02}])[&prob=1.00000000e+00,prob_stddev=0.00000000e+00,prob_range={1.00000000e+00,1.00000000e+00},prob(percent)="100",prob+-sd="100+-0"]:3.010277e-03[&length_mean=3.10579884e-03,length_median=3.01027700e-03,length_95%HPD={1.38155000e-03,5.05483500e-03}])[&prob=5.82278481e-01,prob_stddev=8.21374284e-03,prob_range={5.70286476e-01,5.88940706e-01},prob(percent)="58",prob+-sd="58+-1"]:7.903232e-04[&length_mean=8.84555706e-04,length_median=7.90323200e-04,length_95%HPD={2.18101800e-05,1.96311200e-03}],24[&prob=1.00000000e+00,prob_stddev=0.00000000e+00,prob_range={1.00000000e+00,1.00000000e+00},prob(percent)="100",prob+-sd="100+-0"]:7.290471e-03[&length_mean=7.38883051e-03,length_median=7.29047100e-03,length_95%HPD={4.90939800e-03,1.03992400e-02}])[&prob=1.00000000e+00,prob_stddev=0.00000000e+00,prob_range={1.00000000e+00,1.00000000e+00},prob(percent)="100",prob+-sd="100+-0"]:7.420275e-03[&length_mean=7.46566683e-03,length_median=7.42027500e-03,length_95%HPD={4.82412100e-03,1.02412300e-02}],(25[&prob=1.00000000e+00,prob_stddev=0.00000000e+00,prob_range={1.00000000e+00,1.00000000e+00},prob(percent)="100",prob+-sd="100+-0"]:7.845710e-03[&length_mean=7.92127938e-03,length_median=7.84571000e-03,length_95%HPD={5.53298200e-03,1.04472500e-02}],50[&prob=1.00000000e+00,prob_stddev=0.00000000e+00,prob_range={1.00000000e+00,1.00000000e+00},prob(percent)="100",prob+-sd="100+-0"]:4.181545e-03[&length_mean=4.29522192e-03,length_median=4.18154500e-03,length_95%HPD={2.22837500e-03,6.58359100e-03}])[&prob=1.00000000e+00,prob_stddev=0.00000000e+00,prob_range={1.00000000e+00,1.00000000e+00},prob(percent)="100",prob+-sd="100+-0"]:6.101415e-03[&length_mean=6.16250099e-03,length_median=6.10141500e-03,length_95%HPD={3.89392200e-03,8.50674700e-03}])[&prob=6.25749500e-01,prob_stddev=8.02007585e-03,prob_range={6.19586942e-01,6.36908728e-01},prob(percent)="63",prob+-sd="63+-1"]:1.129234e-03[&length_mean=1.20505194e-03,length_median=1.12923400e-03,length_95%HPD={2.03414700e-04,2.29416600e-03}])[&prob=1.00000000e+00,prob_stddev=0.00000000e+00,prob_range={1.00000000e+00,1.00000000e+00},prob(percent)="100",prob+-sd="100+-0"]:1.133846e-02[&length_mean=1.13935950e-02,length_median=1.13384600e-02,length_95%HPD={8.79631000e-03,1.38482200e-02}])[&prob=1.00000000e+00,prob_stddev=0.00000000e+00,prob_range={1.00000000e+00,1.00000000e+00},prob(percent)="100",prob+-sd="100+-0"]:1.245378e-02[&length_mean=1.24732002e-02,length_median=1.24537800e-02,length_95%HPD={9.53922700e-03,1.53013800e-02}],(((26[&prob=1.00000000e+00,prob_stddev=0.00000000e+00,prob_range={1.00000000e+00,1.00000000e+00},prob(percent)="100",prob+-sd="100+-0"]:1.381026e-02[&length_mean=1.38349152e-02,length_median=1.38102600e-02,length_95%HPD={1.07723300e-02,1.67753300e-02}],64[&prob=1.00000000e+00,prob_stddev=0.00000000e+00,prob_range={1.00000000e+00,1.00000000e+00},prob(percent)="100",prob+-sd="100+-0"]:1.896418e-02[&length_mean=1.90400434e-02,length_median=1.89641800e-02,length_95%HPD={1.45097700e-02,2.39123800e-02}])[&prob=1.00000000e+00,prob_stddev=0.00000000e+00,prob_range={1.00000000e+00,1.00000000e+00},prob(percent)="100",prob+-sd="100+-0"]:3.811298e-03[&length_mean=3.87697629e-03,length_median=3.81129800e-03,length_95%HPD={2.11366300e-03,5.81674300e-03}],((((27[&prob=1.00000000e+00,prob_stddev=0.00000000e+00,prob_range={1.00000000e+00,1.00000000e+00},prob(percent)="100",prob+-sd="100+-0"]:1.214483e-02[&length_mean=1.22644211e-02,length_median=1.21448300e-02,length_95%HPD={8.83198000e-03,1.60328300e-02}],52[&prob=1.00000000e+00,prob_stddev=0.00000000e+00,prob_range={1.00000000e+00,1.00000000e+00},prob(percent)="100",prob+-sd="100+-0"]:1.619817e-02[&length_mean=1.62626259e-02,length_median=1.61981700e-02,length_95%HPD={1.21083500e-02,2.07805400e-02}])[&prob=1.00000000e+00,prob_stddev=0.00000000e+00,prob_range={1.00000000e+00,1.00000000e+00},prob(percent)="100",prob+-sd="100+-0"]:3.280222e-03[&length_mean=3.36635407e-03,length_median=3.28022200e-03,length_95%HPD={1.53949100e-03,5.46717800e-03}],51[&prob=1.00000000e+00,prob_stddev=0.00000000e+00,prob_range={1.00000000e+00,1.00000000e+00},prob(percent)="100",prob+-sd="100+-0"]:1.184831e-02[&length_mean=1.18937129e-02,length_median=1.18483100e-02,length_95%HPD={9.07897700e-03,1.50764000e-02}])[&prob=1.00000000e+00,prob_stddev=0.00000000e+00,prob_range={1.00000000e+00,1.00000000e+00},prob(percent)="100",prob+-sd="100+-0"]:7.992144e-03[&length_mean=8.06006009e-03,length_median=7.99214400e-03,length_95%HPD={5.47773400e-03,1.08468800e-02}],(28[&prob=1.00000000e+00,prob_stddev=0.00000000e+00,prob_range={1.00000000e+00,1.00000000e+00},prob(percent)="100",prob+-sd="100+-0"]:1.450130e-02[&length_mean=1.45373553e-02,length_median=1.45013000e-02,length_95%HPD={1.13362600e-02,1.74849200e-02}],(55[&prob=1.00000000e+00,prob_stddev=0.00000000e+00,prob_range={1.00000000e+00,1.00000000e+00},prob(percent)="100",prob+-sd="100+-0"]:8.644382e-03[&length_mean=8.76566137e-03,length_median=8.64438200e-03,length_95%HPD={5.32291600e-03,1.22144600e-02}],((58[&prob=1.00000000e+00,prob_stddev=0.00000000e+00,prob_range={1.00000000e+00,1.00000000e+00},prob(percent)="100",prob+-sd="100+-0"]:5.992292e-03[&length_mean=6.10412440e-03,length_median=5.99229200e-03,length_95%HPD={3.74972400e-03,8.68280400e-03}],66[&prob=1.00000000e+00,prob_stddev=0.00000000e+00,prob_range={1.00000000e+00,1.00000000e+00},prob(percent)="100",prob+-sd="100+-0"]:5.170875e-03[&length_mean=5.23871085e-03,length_median=5.17087500e-03,length_95%HPD={3.35548400e-03,7.27667400e-03}])[&prob=9.02398401e-01,prob_stddev=1.00081318e-02,prob_range={8.93404397e-01,9.14057295e-01},prob(percent)="90",prob+-sd="90+-1"]:9.398471e-04[&length_mean=1.01241498e-03,length_median=9.39847100e-04,length_95%HPD={1.82124800e-04,2.00444900e-03}],69[&prob=1.00000000e+00,prob_stddev=0.00000000e+00,prob_range={1.00000000e+00,1.00000000e+00},prob(percent)="100",prob+-sd="100+-0"]:6.786740e-03[&length_mean=6.86678690e-03,length_median=6.78674000e-03,length_95%HPD={4.20589000e-03,9.69288300e-03}])[&prob=6.86708861e-01,prob_stddev=6.13925647e-03,prob_range={6.82211859e-01,6.95536309e-01},prob(percent)="69",prob+-sd="69+-1"]:6.498909e-04[&length_mean=7.48087925e-04,length_median=6.49890900e-04,length_95%HPD={3.70187200e-07,1.79113400e-03}])[&prob=1.00000000e+00,prob_stddev=0.00000000e+00,prob_range={1.00000000e+00,1.00000000e+00},prob(percent)="100",prob+-sd="100+-0"]:3.805972e-03[&length_mean=3.85287288e-03,length_median=3.80597200e-03,length_95%HPD={2.01239200e-03,5.72803100e-03}])[&prob=1.00000000e+00,prob_stddev=0.00000000e+00,prob_range={1.00000000e+00,1.00000000e+00},prob(percent)="100",prob+-sd="100+-0"]:8.926983e-03[&length_mean=8.99244317e-03,length_median=8.92698300e-03,length_95%HPD={6.75288700e-03,1.16294600e-02}])[&prob=9.86175883e-01,prob_stddev=1.75213567e-03,prob_range={9.84676882e-01,9.88674217e-01},prob(percent)="99",prob+-sd="99+-0"]:9.385153e-04[&length_mean=9.97846971e-04,length_median=9.38515300e-04,length_95%HPD={2.01466600e-04,1.90985900e-03}],(((53[&prob=1.00000000e+00,prob_stddev=0.00000000e+00,prob_range={1.00000000e+00,1.00000000e+00},prob(percent)="100",prob+-sd="100+-0"]:3.944260e-03[&length_mean=4.04617627e-03,length_median=3.94426000e-03,length_95%HPD={2.06189100e-03,6.19965000e-03}],61[&prob=1.00000000e+00,prob_stddev=0.00000000e+00,prob_range={1.00000000e+00,1.00000000e+00},prob(percent)="100",prob+-sd="100+-0"]:5.546555e-03[&length_mean=5.63886243e-03,length_median=5.54655500e-03,length_95%HPD={3.28467400e-03,8.07841800e-03}])[&prob=1.00000000e+00,prob_stddev=0.00000000e+00,prob_range={1.00000000e+00,1.00000000e+00},prob(percent)="100",prob+-sd="100+-0"]:6.092187e-03[&length_mean=6.18943200e-03,length_median=6.09218700e-03,length_95%HPD={3.78852300e-03,8.87940000e-03}],(60[&prob=1.00000000e+00,prob_stddev=0.00000000e+00,prob_range={1.00000000e+00,1.00000000e+00},prob(percent)="100",prob+-sd="100+-0"]:1.699600e-02[&length_mean=1.71274634e-02,length_median=1.69960000e-02,length_95%HPD={1.30156900e-02,2.15115100e-02}],((62[&prob=1.00000000e+00,prob_stddev=0.00000000e+00,prob_range={1.00000000e+00,1.00000000e+00},prob(percent)="100",prob+-sd="100+-0"]:9.767066e-03[&length_mean=9.90135591e-03,length_median=9.76706600e-03,length_95%HPD={6.40020800e-03,1.41899000e-02}],67[&prob=1.00000000e+00,prob_stddev=0.00000000e+00,prob_range={1.00000000e+00,1.00000000e+00},prob(percent)="100",prob+-sd="100+-0"]:1.346019e-02[&length_mean=1.35575812e-02,length_median=1.34601900e-02,length_95%HPD={9.61396700e-03,1.77999500e-02}])[&prob=6.85876083e-01,prob_stddev=2.33621608e-02,prob_range={6.64890073e-01,7.18187875e-01},prob(percent)="69",prob+-sd="69+-2"]:1.533583e-03[&length_mean=1.68944710e-03,length_median=1.53358300e-03,length_95%HPD={1.95815800e-04,3.61076700e-03}],70[&prob=1.00000000e+00,prob_stddev=0.00000000e+00,prob_range={1.00000000e+00,1.00000000e+00},prob(percent)="100",prob+-sd="100+-0"]:1.470924e-02[&length_mean=1.48638051e-02,length_median=1.47092400e-02,length_95%HPD={1.02868200e-02,1.94929700e-02}])[&prob=1.00000000e+00,prob_stddev=0.00000000e+00,prob_range={1.00000000e+00,1.00000000e+00},prob(percent)="100",prob+-sd="100+-0"]:2.002568e-03[&length_mean=2.12142878e-03,length_median=2.00256800e-03,length_95%HPD={5.64341100e-04,3.80718300e-03}])[&prob=4.02898068e-01,prob_stddev=1.17976578e-02,prob_range={3.87075283e-01,4.13724184e-01},prob(percent)="40",prob+-sd="40+-1"]:7.198358e-04[&length_mean=8.20049260e-04,length_median=7.19835800e-04,length_95%HPD={1.89066900e-07,1.86453800e-03}])[&prob=4.66022652e-01,prob_stddev=1.67970891e-02,prob_range={4.41705530e-01,4.79013991e-01},prob(percent)="47",prob+-sd="47+-2"]:7.629364e-04[&length_mean=8.47547010e-04,length_median=7.62936400e-04,length_95%HPD={2.98143300e-05,1.85530300e-03}],((((54[&prob=1.00000000e+00,prob_stddev=0.00000000e+00,prob_range={1.00000000e+00,1.00000000e+00},prob(percent)="100",prob+-sd="100+-0"]:9.079907e-03[&length_mean=9.12857520e-03,length_median=9.07990700e-03,length_95%HPD={6.04647200e-03,1.25055800e-02}],59[&prob=1.00000000e+00,prob_stddev=0.00000000e+00,prob_range={1.00000000e+00,1.00000000e+00},prob(percent)="100",prob+-sd="100+-0"]:5.191033e-03[&length_mean=5.30609394e-03,length_median=5.19103300e-03,length_95%HPD={2.97274500e-03,7.95013200e-03}])[&prob=3.11292472e-01,prob_stddev=1.69188604e-02,prob_range={2.93137908e-01,3.33777482e-01},prob(percent)="31",prob+-sd="31+-2"]:6.246415e-04[&length_mean=7.43562878e-04,length_median=6.24641500e-04,length_95%HPD={2.71476300e-05,1.74526400e-03}],((56[&prob=1.00000000e+00,prob_stddev=0.00000000e+00,prob_range={1.00000000e+00,1.00000000e+00},prob(percent)="100",prob+-sd="100+-0"]:8.964994e-03[&length_mean=9.07471968e-03,length_median=8.96499400e-03,length_95%HPD={5.57311200e-03,1.23997500e-02}],57[&prob=1.00000000e+00,prob_stddev=0.00000000e+00,prob_range={1.00000000e+00,1.00000000e+00},prob(percent)="100",prob+-sd="100+-0"]:1.020804e-02[&length_mean=1.02904367e-02,length_median=1.02080400e-02,length_95%HPD={7.03636200e-03,1.37767600e-02}])[&prob=5.54463691e-01,prob_stddev=1.85856978e-02,prob_range={5.29646902e-01,5.73617588e-01},prob(percent)="55",prob+-sd="55+-2"]:9.062340e-04[&length_mean=9.84930374e-04,length_median=9.06234000e-04,length_95%HPD={4.21957000e-05,2.07718700e-03}],63[&prob=1.00000000e+00,prob_stddev=0.00000000e+00,prob_range={1.00000000e+00,1.00000000e+00},prob(percent)="100",prob+-sd="100+-0"]:1.078036e-02[&length_mean=1.08921420e-02,length_median=1.07803600e-02,length_95%HPD={7.42949700e-03,1.43549400e-02}])[&prob=3.73917388e-01,prob_stddev=1.76129461e-02,prob_range={3.58427715e-01,3.95069953e-01},prob(percent)="37",prob+-sd="37+-2"]:9.175683e-04[&length_mean=1.02383522e-03,length_median=9.17568300e-04,length_95%HPD={1.21426700e-04,2.14406400e-03}])[&prob=9.66022652e-01,prob_stddev=1.80413911e-03,prob_range={9.63357761e-01,9.67355097e-01},prob(percent)="97",prob+-sd="97+-0"]:1.687483e-03[&length_mean=1.78685755e-03,length_median=1.68748300e-03,length_95%HPD={4.71837700e-04,3.30630800e-03}],65[&prob=1.00000000e+00,prob_stddev=0.00000000e+00,prob_range={1.00000000e+00,1.00000000e+00},prob(percent)="100",prob+-sd="100+-0"]:1.434867e-02[&length_mean=1.44758218e-02,length_median=1.43486700e-02,length_95%HPD={1.05552400e-02,1.85472400e-02}])[&prob=9.94503664e-01,prob_stddev=1.47725235e-03,prob_range={9.92671552e-01,9.96002665e-01},prob(percent)="99",prob+-sd="99+-0"]:1.263518e-03[&length_mean=1.36109310e-03,length_median=1.26351800e-03,length_95%HPD={2.99881400e-04,2.71521000e-03}],68[&prob=1.00000000e+00,prob_stddev=0.00000000e+00,prob_range={1.00000000e+00,1.00000000e+00},prob(percent)="100",prob+-sd="100+-0"]:1.503728e-02[&length_mean=1.51021358e-02,length_median=1.50372800e-02,length_95%HPD={1.10847400e-02,1.91759300e-02}])[&prob=1.00000000e+00,prob_stddev=0.00000000e+00,prob_range={1.00000000e+00,1.00000000e+00},prob(percent)="100",prob+-sd="100+-0"]:4.249131e-03[&length_mean=4.33672310e-03,length_median=4.24913100e-03,length_95%HPD={2.18754700e-03,6.48861800e-03}])[&prob=1.00000000e+00,prob_stddev=0.00000000e+00,prob_range={1.00000000e+00,1.00000000e+00},prob(percent)="100",prob+-sd="100+-0"]:3.530789e-03[&length_mean=3.62786878e-03,length_median=3.53078900e-03,length_95%HPD={1.64704500e-03,5.70872000e-03}])[&prob=9.02398401e-01,prob_stddev=1.44995899e-02,prob_range={8.89407062e-01,9.22718188e-01},prob(percent)="90",prob+-sd="90+-1"]:1.111283e-03[&length_mean=1.16872141e-03,length_median=1.11128300e-03,length_95%HPD={2.53375600e-04,2.15295400e-03}])[&prob=1.00000000e+00,prob_stddev=0.00000000e+00,prob_range={1.00000000e+00,1.00000000e+00},prob(percent)="100",prob+-sd="100+-0"]:6.869386e-03[&length_mean=6.90332514e-03,length_median=6.86938600e-03,length_95%HPD={4.76594300e-03,8.93541700e-03}],((29[&prob=1.00000000e+00,prob_stddev=0.00000000e+00,prob_range={1.00000000e+00,1.00000000e+00},prob(percent)="100",prob+-sd="100+-0"]:3.892949e-03[&length_mean=4.05254357e-03,length_median=3.89294900e-03,length_95%HPD={1.33206100e-03,6.73251900e-03}],(73[&prob=1.00000000e+00,prob_stddev=0.00000000e+00,prob_range={1.00000000e+00,1.00000000e+00},prob(percent)="100",prob+-sd="100+-0"]:9.353466e-03[&length_mean=9.71890279e-03,length_median=9.35346600e-03,length_95%HPD={3.23624100e-03,1.67750700e-02}],74[&prob=1.00000000e+00,prob_stddev=0.00000000e+00,prob_range={1.00000000e+00,1.00000000e+00},prob(percent)="100",prob+-sd="100+-0"]:6.467500e-03[&length_mean=6.62933233e-03,length_median=6.46750000e-03,length_95%HPD={3.29993900e-03,1.01508800e-02}])[&prob=9.78347768e-01,prob_stddev=5.17485241e-03,prob_range={9.72018654e-01,9.84676882e-01},prob(percent)="98",prob+-sd="98+-1"]:1.813020e-03[&length_mean=1.94884092e-03,length_median=1.81302000e-03,length_95%HPD={3.85587200e-04,3.91835600e-03}])[&prob=1.00000000e+00,prob_stddev=0.00000000e+00,prob_range={1.00000000e+00,1.00000000e+00},prob(percent)="100",prob+-sd="100+-0"]:1.026551e-02[&length_mean=1.03502381e-02,length_median=1.02655100e-02,length_95%HPD={7.46172100e-03,1.34554800e-02}],(71[&prob=1.00000000e+00,prob_stddev=0.00000000e+00,prob_range={1.00000000e+00,1.00000000e+00},prob(percent)="100",prob+-sd="100+-0"]:6.226670e-03[&length_mean=6.33760470e-03,length_median=6.22667000e-03,length_95%HPD={3.57744400e-03,9.38977400e-03}],72[&prob=1.00000000e+00,prob_stddev=0.00000000e+00,prob_range={1.00000000e+00,1.00000000e+00},prob(percent)="100",prob+-sd="100+-0"]:7.733477e-03[&length_mean=7.74029209e-03,length_median=7.73347700e-03,length_95%HPD={5.05048300e-03,1.04559900e-02}])[&prob=7.05196536e-01,prob_stddev=7.08204251e-03,prob_range={6.99533644e-01,7.15522985e-01},prob(percent)="71",prob+-sd="71+-1"]:1.536524e-03[&length_mean=1.65572290e-03,length_median=1.53652400e-03,length_95%HPD={3.67950000e-05,3.44655900e-03}])[&prob=1.00000000e+00,prob_stddev=0.00000000e+00,prob_range={1.00000000e+00,1.00000000e+00},prob(percent)="100",prob+-sd="100+-0"]:2.141201e-02[&length_mean=2.14325891e-02,length_median=2.14120100e-02,length_95%HPD={1.79778500e-02,2.53606100e-02}])[&prob=9.98001332e-01,prob_stddev=1.63190523e-03,prob_range={9.96002665e-01,1.00000000e+00},prob(percent)="100",prob+-sd="100+-0"]:9.393820e-03[&length_mean=9.43485569e-03,length_median=9.39382000e-03,length_95%HPD={6.70586200e-03,1.21723600e-02}])[&prob=9.90506329e-01,prob_stddev=5.19980878e-03,prob_range={9.84010660e-01,9.96668887e-01},prob(percent)="99",prob+-sd="99+-1"]:2.243975e-02[&length_mean=2.18482127e-02,length_median=2.24397500e-02,length_95%HPD={1.21494100e-02,2.92453200e-02}])[&prob=1.00000000e+00,prob_stddev=0.00000000e+00,prob_range={1.00000000e+00,1.00000000e+00},prob(percent)="100",prob+-sd="100+-0"]:7.478996e-02[&length_mean=7.42284699e-02,length_median=7.47899600e-02,length_95%HPD={6.11627000e-02,8.75720000e-02}])[&prob=9.99833444e-01,prob_stddev=3.33111259e-04,prob_range={9.99333777e-01,1.00000000e+00},prob(percent)="100",prob+-sd="100+-0"]:9.934592e-03[&length_mean=9.99819742e-03,length_median=9.93459200e-03,length_95%HPD={5.12638800e-03,1.53967800e-02}])[&prob=1.00000000e+00,prob_stddev=0.00000000e+00,prob_range={1.00000000e+00,1.00000000e+00},prob(percent)="100",prob+-sd="100+-0"]:9.240967e-02[&length_mean=9.26865542e-02,length_median=9.24096700e-02,length_95%HPD={8.15538100e-02,1.04685400e-01}],(2[&prob=1.00000000e+00,prob_stddev=0.00000000e+00,prob_range={1.00000000e+00,1.00000000e+00},prob(percent)="100",prob+-sd="100+-0"]:5.364971e-02[&length_mean=5.37771839e-02,length_median=5.36497100e-02,length_95%HPD={4.51446300e-02,6.25232400e-02}],3[&prob=1.00000000e+00,prob_stddev=0.00000000e+00,prob_range={1.00000000e+00,1.00000000e+00},prob(percent)="100",prob+-sd="100+-0"]:3.882034e-02[&length_mean=3.89519765e-02,length_median=3.88203400e-02,length_95%HPD={3.17724700e-02,4.62001200e-02}])[&prob=1.00000000e+00,prob_stddev=0.00000000e+00,prob_range={1.00000000e+00,1.00000000e+00},prob(percent)="100",prob+-sd="100+-0"]:1.765612e-02[&length_mean=1.77845872e-02,length_median=1.76561200e-02,length_95%HPD={1.16847200e-02,2.35700000e-02}]);

end;

**Mitochondrial genes – undated Bayesian analysis (majority rule consensus with all compatible clades)**


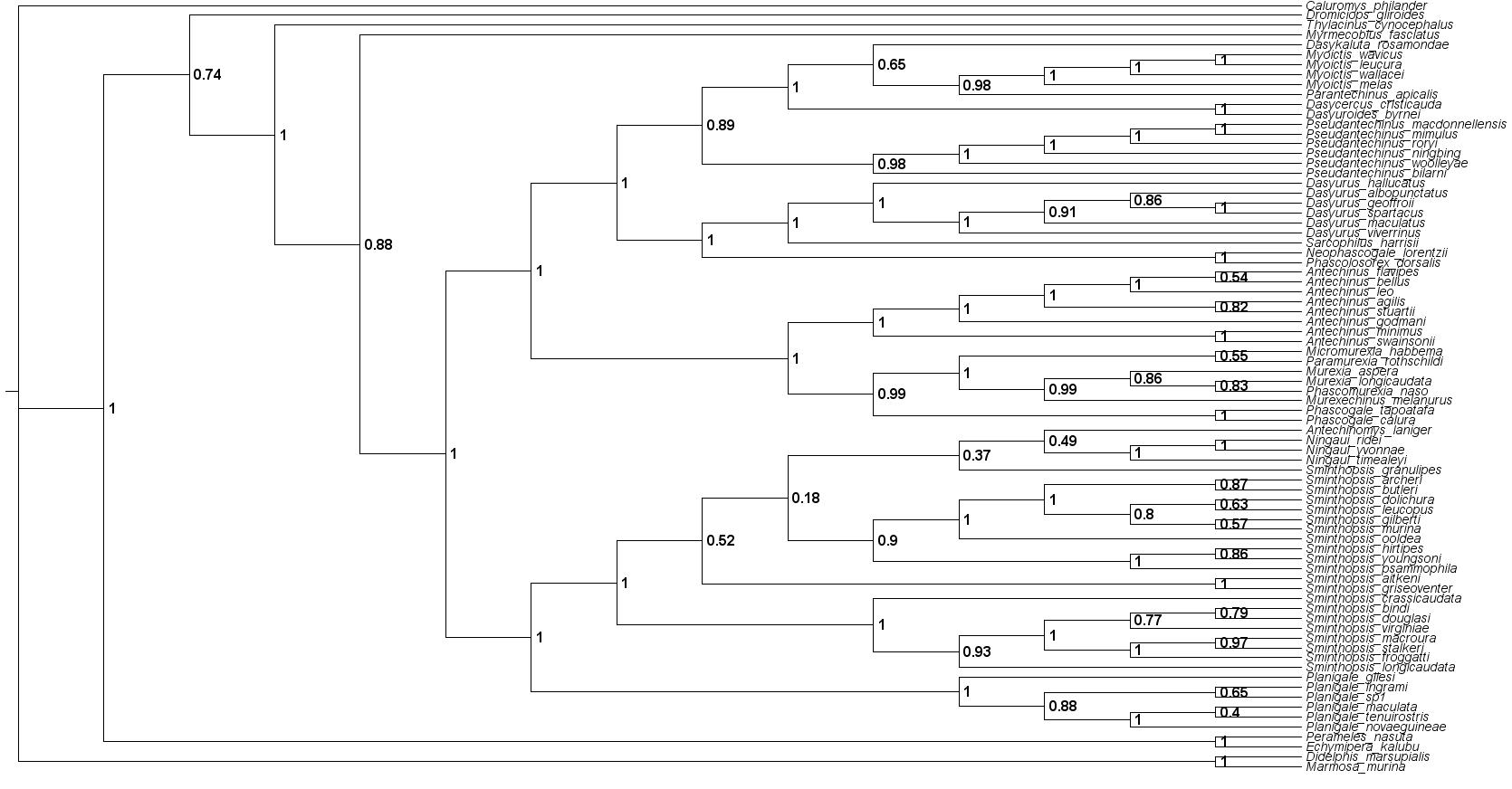


#NEXUS

begin taxa;

dimensions ntax=78;

taxlabels

Caluromys_philander

Didelphis_marsupialis

Marmosa_murina

Dromiciops_gliroides

Perameles_nasuta

Echymipera_kalubu

Thylacinus_cynocephalus

Myrmecobius_fasciatus

Dasykaluta_rosamondae

Dasycercus_cristicauda

Dasyuroides_byrnei

Dasyurus_hallucatus

Myoictis_wavicus

Neophascogale_lorentzii

Phascolosorex_dorsalis

Parantechinus_apicalis

Pseudantechinus_macdonnellensis

Sarcophilus_harrisii

Antechinus_flavipes

Micromurexia_habbema

Murexia_aspera

Murexia_longicaudata

Murexechinus_melanurus

Phascomurexia_naso

Paramurexia_rothschildi

Phascogale_tapoatafa

Antechinomys_laniger

Ningaui_ridei

Sminthopsis_crassicaudata

Planigale_gilesi

Dasyurus_albopunctatus

Dasyurus_geoffroii

Dasyurus_maculatus

Dasyurus_spartacus

Dasyurus_viverrinus

Myoictis_leucura

Myoictis_melas

Myoictis_wallacei

Pseudantechinus_bilarni

Pseudantechinus_mimulus

Pseudantechinus_ningbing

Pseudantechinus_roryi

Pseudantechinus_woolleyae

Antechinus_agilis

Antechinus_bellus

Antechinus_godmani

Antechinus_leo

Antechinus_minimus

Antechinus_stuartii

Antechinus_swainsonii

Phascogale_calura

Ningaui_timealeyi

Ningaui_yvonnae

Sminthopsis_aitkeni

Sminthopsis_archeri

Sminthopsis_bindi

Sminthopsis_butleri

Sminthopsis_dolichura

Sminthopsis_douglasi

Sminthopsis_gilberti

Sminthopsis_granulipes

Sminthopsis_griseoventer

Sminthopsis_hirtipes

Sminthopsis_leucopus

Sminthopsis_longicaudata

Sminthopsis_murina

Sminthopsis_macroura

Sminthopsis_psammophila

Sminthopsis_ooldea

Sminthopsis_virginiae

Sminthopsis_youngsoni

Sminthopsis_froggatti

Sminthopsis_stalkeri

Planigale_ingrami

Planigale_maculata

Planigale_novaeguineae

Planigale_tenuirostris

Planigale_sp1

;

end;

begin trees;

translate

1 Caluromys_philander,

2 Didelphis_marsupialis,

3 Marmosa_murina,

4 Dromiciops_gliroides,

5 Perameles_nasuta,

6 Echymipera_kalubu,

7 Thylacinus_cynocephalus,

8 Myrmecobius_fasciatus,

9 Dasykaluta_rosamondae,

10 Dasycercus_cristicauda,

11 Dasyuroides_byrnei,

12 Dasyurus_hallucatus,

13 Myoictis_wavicus,

14 Neophascogale_lorentzii,

15 Phascolosorex_dorsalis,

16 Parantechinus_apicalis,

17 Pseudantechinus_macdonnellensis,

18 Sarcophilus_harrisii,

19 Antechinus_flavipes,

20 Micromurexia_habbema,

21 Murexia_aspera,

22 Murexia_longicaudata,

23 Murexechinus_melanurus,

24 Phascomurexia_naso,

25 Paramurexia_rothschildi,

26 Phascogale_tapoatafa,

27 Antechinomys_laniger,

28 Ningaui_ridei,

29 Sminthopsis_crassicaudata,

30 Planigale_gilesi,

31 Dasyurus_albopunctatus,

32 Dasyurus_geoffroii,

33 Dasyurus_maculatus,

34 Dasyurus_spartacus,

35 Dasyurus_viverrinus,

36 Myoictis_leucura,

37 Myoictis_melas,

38 Myoictis_wallacei,

39 Pseudantechinus_bilarni,

40 Pseudantechinus_mimulus,

41 Pseudantechinus_ningbing,

42 Pseudantechinus_roryi,

43 Pseudantechinus_woolleyae,

44 Antechinus_agilis,

45 Antechinus_bellus,

46 Antechinus_godmani,

47 Antechinus_leo,

48 Antechinus_minimus,

49 Antechinus_stuartii,

50 Antechinus_swainsonii,

51 Phascogale_calura,

52 Ningaui_timealeyi,

53 Ningaui_yvonnae,

54 Sminthopsis_aitkeni,

55 Sminthopsis_archeri,

56 Sminthopsis_bindi,

57 Sminthopsis_butleri,

58 Sminthopsis_dolichura,

59 Sminthopsis_douglasi,

60 Sminthopsis_gilberti,

61 Sminthopsis_granulipes,

62 Sminthopsis_griseoventer,

63 Sminthopsis_hirtipes,

64 Sminthopsis_leucopus,

65 Sminthopsis_longicaudata,

66 Sminthopsis_murina,

67 Sminthopsis_macroura,

68 Sminthopsis_psammophila,

69 Sminthopsis_ooldea,

70 Sminthopsis_virginiae,

71 Sminthopsis_youngsoni,

72 Sminthopsis_froggatti,

73 Sminthopsis_stalkeri,

74 Planigale_ingrami,

75 Planigale_maculata,

76 Planigale_novaeguineae,

77 Planigale_tenuirostris,

78 Planigale_sp1

;

tree con_all_compat = [&U] (1[&prob=1.00000000e+00,prob_stddev=0.00000000e+00,prob_range={1.00000000e+00,1.00000000e+00},prob(percent)="100",prob+-sd="100+-0"]:1.318835e+00[&length_mean=1.33732715e+00,length_median=1.31883500e+00,length_95%HPD={9.32220700e-01,1.77238900e+00}],((4[&prob=1.00000000e+00,prob_stddev=0.00000000e+00,prob_range={1.00000000e+00,1.00000000e+00},prob(percent)="100",prob+-sd="100+-0"]:1.749135e+00[&length_mean=1.76277133e+00,length_median=1.74913500e+00,length_95%HPD={1.33905600e+00,2.24082700e+00}],(7[&prob=1.00000000e+00,prob_stddev=0.00000000e+00,prob_range={1.00000000e+00,1.00000000e+00},prob(percent)="100",prob+-sd="100+-0"]:2.281464e+00[&length_mean=2.30868575e+00,length_median=2.28146400e+00,length_95%HPD={1.76114400e+00,2.89800800e+00}],(8[&prob=1.00000000e+00,prob_stddev=0.00000000e+00,prob_range={1.00000000e+00,1.00000000e+00},prob(percent)="100",prob+-sd="100+-0"]:1.688112e+00[&length_mean=1.70464612e+00,length_median=1.68811200e+00,length_95%HPD={1.27552300e+00,2.13690300e+00}],((((((9[&prob=1.00000000e+00,prob_stddev=0.00000000e+00,prob_range={1.00000000e+00,1.00000000e+00},prob(percent)="100",prob+-sd="100+-0"]:7.081934e-01[&length_mean=7.15295203e-01,length_median=7.08193400e-01,length_95%HPD={5.48101600e-01,8.87060700e-01}],((((13[&prob=1.00000000e+00,prob_stddev=0.00000000e+00,prob_range={1.00000000e+00,1.00000000e+00},prob(percent)="100",prob+-sd="100+-0"]:1.754270e-01[&length_mean=1.79030742e-01,length_median=1.75427000e-01,length_95%HPD={1.06563400e-01,2.56803800e-01}],36[&prob=1.00000000e+00,prob_stddev=0.00000000e+00,prob_range={1.00000000e+00,1.00000000e+00},prob(percent)="100",prob+-sd="100+-0"]:1.216269e-01[&length_mean=1.24791556e-01,length_median=1.21626900e-01,length_95%HPD={6.40999800e-02,1.92378300e-01}])[&prob=1.00000000e+00,prob_stddev=0.00000000e+00,prob_range={1.00000000e+00,1.00000000e+00},prob(percent)="100",prob+-sd="100+-0"]:1.551855e-01[&length_mean=1.59033874e-01,length_median=1.55185500e-01,length_95%HPD={7.94709400e-02,2.41420100e-01}],38[&prob=1.00000000e+00,prob_stddev=0.00000000e+00,prob_range={1.00000000e+00,1.00000000e+00},prob(percent)="100",prob+-sd="100+-0"]:2.731148e-01[&length_mean=2.76949357e-01,length_median=2.73114800e-01,length_95%HPD={1.72411300e-01,3.90315500e-01}])[&prob=1.00000000e+00,prob_stddev=0.00000000e+00,prob_range={1.00000000e+00,1.00000000e+00},prob(percent)="100",prob+-sd="100+-0"]:1.247413e-01[&length_mean=1.27697365e-01,length_median=1.24741300e-01,length_95%HPD={5.72304300e-02,2.03995900e-01}],37[&prob=1.00000000e+00,prob_stddev=0.00000000e+00,prob_range={1.00000000e+00,1.00000000e+00},prob(percent)="100",prob+-sd="100+-0"]:3.813910e-01[&length_mean=3.85285002e-01,length_median=3.81391000e-01,length_95%HPD={2.48821300e-01,5.27004800e-01}])[&prob=1.00000000e+00,prob_stddev=0.00000000e+00,prob_range={1.00000000e+00,1.00000000e+00},prob(percent)="100",prob+-sd="100+-0"]:2.677303e-01[&length_mean=2.70745098e-01,length_median=2.67730300e-01,length_95%HPD={1.62874200e-01,3.81619600e-01}],16[&prob=1.00000000e+00,prob_stddev=0.00000000e+00,prob_range={1.00000000e+00,1.00000000e+00},prob(percent)="100",prob+-sd="100+-0"]:7.025876e-01[&length_mean=7.08400221e-01,length_median=7.02587600e-01,length_95%HPD={5.42968000e-01,8.81393300e-01}])[&prob=9.84177215e-01,prob_stddev=4.15611088e-03,prob_range={9.80013324e-01,9.88674217e-01},prob(percent)="98",prob+-sd="98+-0"]:7.256336e-02[&length_mean=7.43628215e-02,length_median=7.25633600e-02,length_95%HPD={2.38319800e-02,1.27350200e-01}])[&prob=6.52065290e-01,prob_stddev=9.91717218e-03,prob_range={6.40906063e-01,6.64223851e-01},prob(percent)="65",prob+-sd="65+-1"]:3.018382e-02[&length_mean=3.23478918e-02,length_median=3.01838200e-02,length_95%HPD={3.46479300e-03,6.52495300e-02}],(10[&prob=1.00000000e+00,prob_stddev=0.00000000e+00,prob_range={1.00000000e+00,1.00000000e+00},prob(percent)="100",prob+-sd="100+-0"]:6.062300e-01[&length_mean=6.10697744e-01,length_median=6.06230000e-01,length_95%HPD={4.58428700e-01,7.59372100e-01}],11[&prob=1.00000000e+00,prob_stddev=0.00000000e+00,prob_range={1.00000000e+00,1.00000000e+00},prob(percent)="100",prob+-sd="100+-0"]:6.238889e-01[&length_mean=6.29392473e-01,length_median=6.23888900e-01,length_95%HPD={4.86947000e-01,7.90917400e-01}])[&prob=1.00000000e+00,prob_stddev=0.00000000e+00,prob_range={1.00000000e+00,1.00000000e+00},prob(percent)="100",prob+-sd="100+-0"]:1.605754e-01[&length_mean=1.62377455e-01,length_median=1.60575400e-01,length_95%HPD={9.55315000e-02,2.37322000e-01}])[&prob=1.00000000e+00,prob_stddev=0.00000000e+00,prob_range={1.00000000e+00,1.00000000e+00},prob(percent)="100",prob+-sd="100+-0"]:8.958397e-02[&length_mean=9.15850553e-02,length_median=8.95839700e-02,length_95%HPD={4.97087100e-02,1.37781200e-01}],(((((17[&prob=1.00000000e+00,prob_stddev=0.00000000e+00,prob_range={1.00000000e+00,1.00000000e+00},prob(percent)="100",prob+-sd="100+-0"]:8.925425e-02[&length_mean=9.19893688e-02,length_median=8.92542500e-02,length_95%HPD={4.42002100e-02,1.44481800e-01}],40[&prob=1.00000000e+00,prob_stddev=0.00000000e+00,prob_range={1.00000000e+00,1.00000000e+00},prob(percent)="100",prob+-sd="100+-0"]:2.418026e-01[&length_mean=2.45649373e-01,length_median=2.41802600e-01,length_95%HPD={1.63259500e-01,3.44278500e-01}])[&prob=1.00000000e+00,prob_stddev=0.00000000e+00,prob_range={1.00000000e+00,1.00000000e+00},prob(percent)="100",prob+-sd="100+-0"]:1.147027e-01[&length_mean=1.17681236e-01,length_median=1.14702700e-01,length_95%HPD={5.69181600e-02,1.78032900e-01}],42[&prob=1.00000000e+00,prob_stddev=0.00000000e+00,prob_range={1.00000000e+00,1.00000000e+00},prob(percent)="100",prob+-sd="100+-0"]:9.849265e-02[&length_mean=1.02056509e-01,length_median=9.84926500e-02,length_95%HPD={4.72512100e-02,1.62059800e-01}])[&prob=1.00000000e+00,prob_stddev=0.00000000e+00,prob_range={1.00000000e+00,1.00000000e+00},prob(percent)="100",prob+-sd="100+-0"]:1.083232e-01[&length_mean=1.12840643e-01,length_median=1.08323200e-01,length_95%HPD={4.29867400e-02,1.87727900e-01}],41[&prob=1.00000000e+00,prob_stddev=0.00000000e+00,prob_range={1.00000000e+00,1.00000000e+00},prob(percent)="100",prob+-sd="100+-0"]:5.100356e-01[&length_mean=5.18956257e-01,length_median=5.10035600e-01,length_95%HPD={3.20285500e-01,7.13900400e-01}])[&prob=1.00000000e+00,prob_stddev=0.00000000e+00,prob_range={1.00000000e+00,1.00000000e+00},prob(percent)="100",prob+-sd="100+-0"]:1.306620e-01[&length_mean=1.33973852e-01,length_median=1.30662000e-01,length_95%HPD={5.56350100e-02,2.20049800e-01}],43[&prob=1.00000000e+00,prob_stddev=0.00000000e+00,prob_range={1.00000000e+00,1.00000000e+00},prob(percent)="100",prob+-sd="100+-0"]:5.319268e-01[&length_mean=5.39344533e-01,length_median=5.31926800e-01,length_95%HPD={3.77712200e-01,7.13726500e-01}])[&prob=1.00000000e+00,prob_stddev=0.00000000e+00,prob_range={1.00000000e+00,1.00000000e+00},prob(percent)="100",prob+-sd="100+-0"]:2.751371e-01[&length_mean=2.78044838e-01,length_median=2.75137100e-01,length_95%HPD={1.66672400e-01,3.94107200e-01}],39[&prob=1.00000000e+00,prob_stddev=0.00000000e+00,prob_range={1.00000000e+00,1.00000000e+00},prob(percent)="100",prob+-sd="100+-0"]:5.375157e-01[&length_mean=5.43213446e-01,length_median=5.37515700e-01,length_95%HPD={4.16886700e-01,6.91757300e-01}])[&prob=9.78347768e-01,prob_stddev=8.77965685e-03,prob_range={9.67355097e-01,9.86675550e-01},prob(percent)="98",prob+-sd="98+-1"]:7.071260e-02[&length_mean=7.25325965e-02,length_median=7.07126000e-02,length_95%HPD={3.35319300e-02,1.19485900e-01}])[&prob=8.89407062e-01,prob_stddev=4.64766812e-03,prob_range={8.85409727e-01,8.96069287e-01},prob(percent)="89",prob+-sd="89+-0"]:4.515613e-02[&length_mean=4.66857863e-02,length_median=4.51561300e-02,length_95%HPD={1.06018600e-02,8.41724800e-02}],(((12[&prob=1.00000000e+00,prob_stddev=0.00000000e+00,prob_range={1.00000000e+00,1.00000000e+00},prob(percent)="100",prob+-sd="100+-0"]:7.168641e-01[&length_mean=7.22645032e-01,length_median=7.16864100e-01,length_95%HPD={5.66472900e-01,9.06920000e-01}],(((31[&prob=1.00000000e+00,prob_stddev=0.00000000e+00,prob_range={1.00000000e+00,1.00000000e+00},prob(percent)="100",prob+-sd="100+-0"]:4.828232e-01[&length_mean=4.91269180e-01,length_median=4.82823200e-01,length_95%HPD={3.04449600e-01,6.86467600e-01}],(32[&prob=1.00000000e+00,prob_stddev=0.00000000e+00,prob_range={1.00000000e+00,1.00000000e+00},prob(percent)="100",prob+-sd="100+-0"]:5.200837e-02[&length_mean=5.50276972e-02,length_median=5.20083700e-02,length_95%HPD={1.25579600e-02,1.05041200e-01}],34[&prob=1.00000000e+00,prob_stddev=0.00000000e+00,prob_range={1.00000000e+00,1.00000000e+00},prob(percent)="100",prob+-sd="100+-0"]:1.419082e-01[&length_mean=1.46768327e-01,length_median=1.41908200e-01,length_95%HPD={6.55651700e-02,2.37915900e-01}])[&prob=1.00000000e+00,prob_stddev=0.00000000e+00,prob_range={1.00000000e+00,1.00000000e+00},prob(percent)="100",prob+-sd="100+-0"]:2.551327e-01[&length_mean=2.58147954e-01,length_median=2.55132700e-01,length_95%HPD={1.58884100e-01,3.61760700e-01}])[&prob=8.60093271e-01,prob_stddev=7.95757178e-03,prob_range={8.51432378e-01,8.70086609e-01},prob(percent)="86",prob+-sd="86+-1"]:5.242781e-02[&length_mean=5.63883264e-02,length_median=5.24278100e-02,length_95%HPD={6.60257300e-03,1.12771100e-01}],33[&prob=1.00000000e+00,prob_stddev=0.00000000e+00,prob_range={1.00000000e+00,1.00000000e+00},prob(percent)="100",prob+-sd="100+-0"]:4.541138e-01[&length_mean=4.61595936e-01,length_median=4.54113800e-01,length_95%HPD={3.08756400e-01,6.20280800e-01}])[&prob=9.10726183e-01,prob_stddev=6.50491606e-03,prob_range={9.04063957e-01,9.19387075e-01},prob(percent)="91",prob+-sd="91+-1"]:5.253805e-02[&length_mean=5.57525945e-02,length_median=5.25380500e-02,length_95%HPD={7.43809700e-03,1.12646100e-01}],35[&prob=1.00000000e+00,prob_stddev=0.00000000e+00,prob_range={1.00000000e+00,1.00000000e+00},prob(percent)="100",prob+-sd="100+-0"]:2.221867e-01[&length_mean=2.29387472e-01,length_median=2.22186700e-01,length_95%HPD={1.15738100e-01,3.68426000e-01}])[&prob=9.99500333e-01,prob_stddev=6.37859499e-04,prob_range={9.98667555e-01,1.00000000e+00},prob(percent)="100",prob+-sd="100+-0"]:1.116066e-01[&length_mean=1.14921250e-01,length_median=1.11606600e-01,length_95%HPD={5.01150800e-02,1.90366900e-01}])[&prob=1.00000000e+00,prob_stddev=0.00000000e+00,prob_range={1.00000000e+00,1.00000000e+00},prob(percent)="100",prob+-sd="100+-0"]:1.547640e-01[&length_mean=1.56986663e-01,length_median=1.54764000e-01,length_95%HPD={9.15536900e-02,2.29801900e-01}],18[&prob=1.00000000e+00,prob_stddev=0.00000000e+00,prob_range={1.00000000e+00,1.00000000e+00},prob(percent)="100",prob+-sd="100+-0"]:8.457901e-01[&length_mean=8.52253830e-01,length_median=8.45790100e-01,length_95%HPD={6.62765400e-01,1.04980400e+00}])[&prob=9.99333777e-01,prob_stddev=7.69287501e-04,prob_range={9.98667555e-01,1.00000000e+00},prob(percent)="100",prob+-sd="100+-0"]:9.502494e-02[&length_mean=9.72661928e-02,length_median=9.50249400e-02,length_95%HPD={4.80143900e-02,1.53016600e-01}],(14[&prob=1.00000000e+00,prob_stddev=0.00000000e+00,prob_range={1.00000000e+00,1.00000000e+00},prob(percent)="100",prob+-sd="100+-0"]:5.072348e-01[&length_mean=5.12897179e-01,length_median=5.07234800e-01,length_95%HPD={3.83361200e-01,6.43937200e-01}],15[&prob=1.00000000e+00,prob_stddev=0.00000000e+00,prob_range={1.00000000e+00,1.00000000e+00},prob(percent)="100",prob+-sd="100+-0"]:4.942345e-01[&length_mean=4.99073287e-01,length_median=4.94234500e-01,length_95%HPD={3.78661300e-01,6.31641700e-01}])[&prob=1.00000000e+00,prob_stddev=0.00000000e+00,prob_range={1.00000000e+00,1.00000000e+00},prob(percent)="100",prob+-sd="100+-0"]:4.247412e-01[&length_mean=4.30401376e-01,length_median=4.24741200e-01,length_95%HPD={3.14310600e-01,5.47627200e-01}])[&prob=1.00000000e+00,prob_stddev=0.00000000e+00,prob_range={1.00000000e+00,1.00000000e+00},prob(percent)="100",prob+-sd="100+-0"]:1.144244e-01[&length_mean=1.16349113e-01,length_median=1.14424400e-01,length_95%HPD={6.06769200e-02,1.74014600e-01}])[&prob=1.00000000e+00,prob_stddev=0.00000000e+00,prob_range={1.00000000e+00,1.00000000e+00},prob(percent)="100",prob+-sd="100+-0"]:5.432357e-01[&length_mean=5.48316235e-01,length_median=5.43235700e-01,length_95%HPD={4.05410100e-01,7.04719200e-01}],((((((19[&prob=1.00000000e+00,prob_stddev=0.00000000e+00,prob_range={1.00000000e+00,1.00000000e+00},prob(percent)="100",prob+-sd="100+-0"]:1.948042e-01[&length_mean=1.98260631e-01,length_median=1.94804200e-01,length_95%HPD={1.14955000e-01,3.01560900e-01}],45[&prob=1.00000000e+00,prob_stddev=0.00000000e+00,prob_range={1.00000000e+00,1.00000000e+00},prob(percent)="100",prob+-sd="100+-0"]:3.046369e-01[&length_mean=3.12173371e-01,length_median=3.04636900e-01,length_95%HPD={1.82140800e-01,4.64674500e-01}])[&prob=5.37974684e-01,prob_stddev=8.38311618e-03,prob_range={5.28314457e-01,5.45636243e-01},prob(percent)="54",prob+-sd="54+-1"]:3.971534e-02[&length_mean=4.39276660e-02,length_median=3.97153400e-02,length_95%HPD={1.02757000e-03,9.19645000e-02}],47[&prob=1.00000000e+00,prob_stddev=0.00000000e+00,prob_range={1.00000000e+00,1.00000000e+00},prob(percent)="100",prob+-sd="100+-0"]:2.245051e-01[&length_mean=2.30163739e-01,length_median=2.24505100e-01,length_95%HPD={1.23613100e-01,3.56445700e-01}])[&prob=1.00000000e+00,prob_stddev=0.00000000e+00,prob_range={1.00000000e+00,1.00000000e+00},prob(percent)="100",prob+-sd="100+-0"]:1.125230e-01[&length_mean=1.15110915e-01,length_median=1.12523000e-01,length_95%HPD={3.80239500e-02,1.93591400e-01}],(44[&prob=1.00000000e+00,prob_stddev=0.00000000e+00,prob_range={1.00000000e+00,1.00000000e+00},prob(percent)="100",prob+-sd="100+-0"]:2.369930e-01[&length_mean=2.42770199e-01,length_median=2.36993000e-01,length_95%HPD={1.23350100e-01,3.70389800e-01}],49[&prob=1.00000000e+00,prob_stddev=0.00000000e+00,prob_range={1.00000000e+00,1.00000000e+00},prob(percent)="100",prob+-sd="100+-0"]:3.888602e-01[&length_mean=3.97107558e-01,length_median=3.88860200e-01,length_95%HPD={2.41081300e-01,5.71180300e-01}])[&prob=8.23117921e-01,prob_stddev=8.71198989e-03,prob_range={8.13457695e-01,8.31445703e-01},prob(percent)="82",prob+-sd="82+-1"]:6.259152e-02[&length_mean=6.77721947e-02,length_median=6.25915200e-02,length_95%HPD={3.46972700e-04,1.35392000e-01}])[&prob=1.00000000e+00,prob_stddev=0.00000000e+00,prob_range={1.00000000e+00,1.00000000e+00},prob(percent)="100",prob+-sd="100+-0"]:1.277897e-01[&length_mean=1.31379681e-01,length_median=1.27789700e-01,length_95%HPD={5.46447400e-02,2.19541300e-01}],46[&prob=1.00000000e+00,prob_stddev=0.00000000e+00,prob_range={1.00000000e+00,1.00000000e+00},prob(percent)="100",prob+-sd="100+-0"]:4.418274e-01[&length_mean=4.46309192e-01,length_median=4.41827400e-01,length_95%HPD={3.31587300e-01,5.65356100e-01}])[&prob=1.00000000e+00,prob_stddev=0.00000000e+00,prob_range={1.00000000e+00,1.00000000e+00},prob(percent)="100",prob+-sd="100+-0"]:1.544740e-01[&length_mean=1.57404876e-01,length_median=1.54474000e-01,length_95%HPD={9.70682600e-02,2.26354900e-01}],(48[&prob=1.00000000e+00,prob_stddev=0.00000000e+00,prob_range={1.00000000e+00,1.00000000e+00},prob(percent)="100",prob+-sd="100+-0"]:2.731789e-01[&length_mean=2.81199718e-01,length_median=2.73178900e-01,length_95%HPD={1.49859800e-01,4.26311000e-01}],50[&prob=1.00000000e+00,prob_stddev=0.00000000e+00,prob_range={1.00000000e+00,1.00000000e+00},prob(percent)="100",prob+-sd="100+-0"]:3.583177e-01[&length_mean=3.61970253e-01,length_median=3.58317700e-01,length_95%HPD={2.43031000e-01,4.99981100e-01}])[&prob=1.00000000e+00,prob_stddev=0.00000000e+00,prob_range={1.00000000e+00,1.00000000e+00},prob(percent)="100",prob+-sd="100+-0"]:1.629406e-01[&length_mean=1.67356662e-01,length_median=1.62940600e-01,length_95%HPD={6.76726300e-02,2.72289300e-01}])[&prob=1.00000000e+00,prob_stddev=0.00000000e+00,prob_range={1.00000000e+00,1.00000000e+00},prob(percent)="100",prob+-sd="100+-0"]:2.153145e-01[&length_mean=2.18670429e-01,length_median=2.15314500e-01,length_95%HPD={1.39793500e-01,3.03612100e-01}],(((20[&prob=1.00000000e+00,prob_stddev=0.00000000e+00,prob_range={1.00000000e+00,1.00000000e+00},prob(percent)="100",prob+-sd="100+-0"]:5.345174e-01[&length_mean=5.40378239e-01,length_median=5.34517400e-01,length_95%HPD={4.11655500e-01,6.79410100e-01}],25[&prob=1.00000000e+00,prob_stddev=0.00000000e+00,prob_range={1.00000000e+00,1.00000000e+00},prob(percent)="100",prob+-sd="100+-0"]:7.491085e-01[&length_mean=7.56901964e-01,length_median=7.49108500e-01,length_95%HPD={5.78947200e-01,9.41832200e-01}])[&prob=5.52465023e-01,prob_stddev=1.08572470e-02,prob_range={5.36309127e-01,5.59626915e-01},prob(percent)="55",prob+-sd="55+-1"]:5.203380e-02[&length_mean=5.41850180e-02,length_median=5.20338000e-02,length_95%HPD={1.75559400e-02,9.42548800e-02}],((21[&prob=1.00000000e+00,prob_stddev=0.00000000e+00,prob_range={1.00000000e+00,1.00000000e+00},prob(percent)="100",prob+-sd="100+-0"]:1.436864e+00[&length_mean=1.46381643e+00,length_median=1.43686400e+00,length_95%HPD={8.77684000e-01,2.06508000e+00}],(22[&prob=1.00000000e+00,prob_stddev=0.00000000e+00,prob_range={1.00000000e+00,1.00000000e+00},prob(percent)="100",prob+-sd="100+-0"]:3.292375e-01[&length_mean=3.30127425e-01,length_median=3.29237500e-01,length_95%HPD={2.30660300e-01,4.29904200e-01}],24[&prob=1.00000000e+00,prob_stddev=0.00000000e+00,prob_range={1.00000000e+00,1.00000000e+00},prob(percent)="100",prob+-sd="100+-0"]:3.420555e-01[&length_mean=3.44819955e-01,length_median=3.42055500e-01,length_95%HPD={2.53575100e-01,4.43837000e-01}])[&prob=8.26115923e-01,prob_stddev=1.49566584e-02,prob_range={8.10126582e-01,8.40772818e-01},prob(percent)="83",prob+-sd="83+-1"]:1.428749e-01[&length_mean=1.47277896e-01,length_median=1.42874900e-01,length_95%HPD={1.82592400e-04,2.78336200e-01}])[&prob=8.63257828e-01,prob_stddev=1.61493126e-02,prob_range={8.45436376e-01,8.84077282e-01},prob(percent)="86",prob+-sd="86+-2"]:1.499289e-01[&length_mean=1.49863148e-01,length_median=1.49928900e-01,length_95%HPD={6.69271800e-03,2.78696000e-01}],23[&prob=1.00000000e+00,prob_stddev=0.00000000e+00,prob_range={1.00000000e+00,1.00000000e+00},prob(percent)="100",prob+-sd="100+-0"]:4.524357e-01[&length_mean=4.56230453e-01,length_median=4.52435700e-01,length_95%HPD={3.41270600e-01,5.77000400e-01}])[&prob=9.94003997e-01,prob_stddev=9.42180921e-04,prob_range={9.92671552e-01,9.94670220e-01},prob(percent)="99",prob+-sd="99+-0"]:1.947518e-01[&length_mean=1.95701307e-01,length_median=1.94751800e-01,length_95%HPD={1.21912600e-01,2.79067000e-01}])[&prob=1.00000000e+00,prob_stddev=0.00000000e+00,prob_range={1.00000000e+00,1.00000000e+00},prob(percent)="100",prob+-sd="100+-0"]:1.945695e-01[&length_mean=1.97589292e-01,length_median=1.94569500e-01,length_95%HPD={1.26193000e-01,2.71101900e-01}],(26[&prob=1.00000000e+00,prob_stddev=0.00000000e+00,prob_range={1.00000000e+00,1.00000000e+00},prob(percent)="100",prob+-sd="100+-0"]:5.076674e-01[&length_mean=5.10938658e-01,length_median=5.07667400e-01,length_95%HPD={3.84394700e-01,6.39706400e-01}],51[&prob=1.00000000e+00,prob_stddev=0.00000000e+00,prob_range={1.00000000e+00,1.00000000e+00},prob(percent)="100",prob+-sd="100+-0"]:4.061862e-01[&length_mean=4.09812313e-01,length_median=4.06186200e-01,length_95%HPD={3.04319000e-01,5.21778900e-01}])[&prob=1.00000000e+00,prob_stddev=0.00000000e+00,prob_range={1.00000000e+00,1.00000000e+00},prob(percent)="100",prob+-sd="100+-0"]:3.338414e-01[&length_mean=3.36693595e-01,length_median=3.33841400e-01,length_95%HPD={2.39215800e-01,4.32548500e-01}])[&prob=9.86842105e-01,prob_stddev=7.66155896e-03,prob_range={9.77348434e-01,9.96002665e-01},prob(percent)="99",prob+-sd="99+-1"]:1.118317e-01[&length_mean=1.14231158e-01,length_median=1.11831700e-01,length_95%HPD={5.53251000e-02,1.75232900e-01}])[&prob=1.00000000e+00,prob_stddev=0.00000000e+00,prob_range={1.00000000e+00,1.00000000e+00},prob(percent)="100",prob+-sd="100+-0"]:4.149934e-01[&length_mean=4.19300476e-01,length_median=4.14993400e-01,length_95%HPD={2.92249200e-01,5.51635100e-01}])[&prob=1.00000000e+00,prob_stddev=0.00000000e+00,prob_range={1.00000000e+00,1.00000000e+00},prob(percent)="100",prob+-sd="100+-0"]:3.630911e-01[&length_mean=3.68834548e-01,length_median=3.63091100e-01,length_95%HPD={2.55183400e-01,5.02541600e-01}],((((((27[&prob=1.00000000e+00,prob_stddev=0.00000000e+00,prob_range={1.00000000e+00,1.00000000e+00},prob(percent)="100",prob+-sd="100+-0"]:6.545737e-01[&length_mean=6.60423426e-01,length_median=6.54573700e-01,length_95%HPD={4.97171800e-01,8.38575400e-01}],((28[&prob=1.00000000e+00,prob_stddev=0.00000000e+00,prob_range={1.00000000e+00,1.00000000e+00},prob(percent)="100",prob+-sd="100+-0"]:3.184882e-01[&length_mean=3.24435404e-01,length_median=3.18488200e-01,length_95%HPD={1.86244700e-01,4.66193500e-01}],53[&prob=1.00000000e+00,prob_stddev=0.00000000e+00,prob_range={1.00000000e+00,1.00000000e+00},prob(percent)="100",prob+-sd="100+-0"]:4.186694e-01[&length_mean=4.28405334e-01,length_median=4.18669400e-01,length_95%HPD={2.60760700e-01,6.25560700e-01}])[&prob=1.00000000e+00,prob_stddev=0.00000000e+00,prob_range={1.00000000e+00,1.00000000e+00},prob(percent)="100",prob+-sd="100+-0"]:1.774195e-01[&length_mean=1.82707918e-01,length_median=1.77419500e-01,length_95%HPD={6.97482100e-02,3.09292500e-01}],52[&prob=1.00000000e+00,prob_stddev=0.00000000e+00,prob_range={1.00000000e+00,1.00000000e+00},prob(percent)="100",prob+-sd="100+-0"]:8.190376e-01[&length_mean=8.32054120e-01,length_median=8.19037600e-01,length_95%HPD={5.52684900e-01,1.13511900e+00}])[&prob=1.00000000e+00,prob_stddev=0.00000000e+00,prob_range={1.00000000e+00,1.00000000e+00},prob(percent)="100",prob+-sd="100+-0"]:3.054286e-01[&length_mean=3.10515834e-01,length_median=3.05428600e-01,length_95%HPD={1.68670000e-01,4.56206100e-01}])[&prob=4.90006662e-01,prob_stddev=4.69517533e-03,prob_range={4.83011326e-01,4.93004664e-01},prob(percent)="49",prob+-sd="49+-0"]:7.184506e-02[&length_mean=7.27986815e-02,length_median=7.18450600e-02,length_95%HPD={1.12046900e-03,1.31315800e-01}],61[&prob=1.00000000e+00,prob_stddev=0.00000000e+00,prob_range={1.00000000e+00,1.00000000e+00},prob(percent)="100",prob+-sd="100+-0"]:5.853269e-01[&length_mean=5.93937845e-01,length_median=5.85326900e-01,length_95%HPD={3.72833700e-01,8.33038800e-01}])[&prob=3.66422385e-01,prob_stddev=2.91670565e-02,prob_range={3.24450366e-01,3.91738841e-01},prob(percent)="37",prob+-sd="37+-3"]:7.286989e-02[&length_mean=7.42435272e-02,length_median=7.28698900e-02,length_95%HPD={1.26247700e-02,1.29653500e-01}],((((55[&prob=1.00000000e+00,prob_stddev=0.00000000e+00,prob_range={1.00000000e+00,1.00000000e+00},prob(percent)="100",prob+-sd="100+-0"]:4.000940e-01[&length_mean=4.07461523e-01,length_median=4.00094000e-01,length_95%HPD={2.35288800e-01,5.82858600e-01}],57[&prob=1.00000000e+00,prob_stddev=0.00000000e+00,prob_range={1.00000000e+00,1.00000000e+00},prob(percent)="100",prob+-sd="100+-0"]:5.697330e-01[&length_mean=5.77178034e-01,length_median=5.69733000e-01,length_95%HPD={3.81155800e-01,7.67941400e-01}])[&prob=8.74583611e-01,prob_stddev=1.12454862e-02,prob_range={8.62091939e-01,8.89407062e-01},prob(percent)="87",prob+-sd="87+-1"]:8.531094e-02[&length_mean=9.13678350e-02,length_median=8.53109400e-02,length_95%HPD={5.03493200e-04,1.83363200e-01}],((58[&prob=1.00000000e+00,prob_stddev=0.00000000e+00,prob_range={1.00000000e+00,1.00000000e+00},prob(percent)="100",prob+-sd="100+-0"]:2.362046e-01[&length_mean=2.39077959e-01,length_median=2.36204600e-01,length_95%HPD={1.23361600e-01,3.61472000e-01}],64[&prob=1.00000000e+00,prob_stddev=0.00000000e+00,prob_range={1.00000000e+00,1.00000000e+00},prob(percent)="100",prob+-sd="100+-0"]:4.195631e-01[&length_mean=4.28599667e-01,length_median=4.19563100e-01,length_95%HPD={2.68843500e-01,6.23780100e-01}])[&prob=6.25582945e-01,prob_stddev=9.05343865e-03,prob_range={6.15589607e-01,6.33577615e-01},prob(percent)="63",prob+-sd="63+-1"]:6.153715e-02[&length_mean=6.72901971e-02,length_median=6.15371500e-02,length_95%HPD={1.53419900e-04,1.40365200e-01}],(60[&prob=1.00000000e+00,prob_stddev=0.00000000e+00,prob_range={1.00000000e+00,1.00000000e+00},prob(percent)="100",prob+-sd="100+-0"]:1.682407e-01[&length_mean=1.73945533e-01,length_median=1.68240700e-01,length_95%HPD={7.91186600e-02,2.81981700e-01}],66[&prob=1.00000000e+00,prob_stddev=0.00000000e+00,prob_range={1.00000000e+00,1.00000000e+00},prob(percent)="100",prob+-sd="100+-0"]:1.475315e-01[&length_mean=1.53386748e-01,length_median=1.47531500e-01,length_95%HPD={6.84557700e-02,2.53748200e-01}])[&prob=5.70786143e-01,prob_stddev=1.20948902e-02,prob_range={5.52964690e-01,5.78947368e-01},prob(percent)="57",prob+-sd="57+-1"]:4.073377e-02[&length_mean=4.67821819e-02,length_median=4.07337700e-02,length_95%HPD={3.00067700e-05,1.04784700e-01}])[&prob=7.95802798e-01,prob_stddev=1.36589157e-02,prob_range={7.81479014e-01,8.14123917e-01},prob(percent)="80",prob+-sd="80+-1"]:7.693444e-02[&length_mean=8.26373632e-02,length_median=7.69344400e-02,length_95%HPD={9.68090400e-03,1.58143400e-01}])[&prob=1.00000000e+00,prob_stddev=0.00000000e+00,prob_range={1.00000000e+00,1.00000000e+00},prob(percent)="100",prob+-sd="100+-0"]:1.975252e-01[&length_mean=2.02343657e-01,length_median=1.97525200e-01,length_95%HPD={9.06117300e-02,3.20819400e-01}],69[&prob=1.00000000e+00,prob_stddev=0.00000000e+00,prob_range={1.00000000e+00,1.00000000e+00},prob(percent)="100",prob+-sd="100+-0"]:4.941713e-01[&length_mean=5.04156863e-01,length_median=4.94171300e-01,length_95%HPD={3.08175800e-01,7.09849300e-01}])[&prob=1.00000000e+00,prob_stddev=0.00000000e+00,prob_range={1.00000000e+00,1.00000000e+00},prob(percent)="100",prob+-sd="100+-0"]:1.439555e-01[&length_mean=1.49716523e-01,length_median=1.43955500e-01,length_95%HPD={5.26299000e-02,2.46294800e-01}],((63[&prob=1.00000000e+00,prob_stddev=0.00000000e+00,prob_range={1.00000000e+00,1.00000000e+00},prob(percent)="100",prob+-sd="100+-0"]:6.440968e-01[&length_mean=6.55014531e-01,length_median=6.44096800e-01,length_95%HPD={4.24647900e-01,9.23134200e-01}],71[&prob=1.00000000e+00,prob_stddev=0.00000000e+00,prob_range={1.00000000e+00,1.00000000e+00},prob(percent)="100",prob+-sd="100+-0"]:5.788573e-01[&length_mean=5.89680649e-01,length_median=5.78857300e-01,length_95%HPD={3.68558600e-01,8.17412800e-01}])[&prob=8.58594270e-01,prob_stddev=1.06924736e-02,prob_range={8.43437708e-01,8.66755496e-01},prob(percent)="86",prob+-sd="86+-1"]:9.522629e-02[&length_mean=1.02996611e-01,length_median=9.52262900e-02,length_95%HPD={8.74207000e-04,2.08819000e-01}],68[&prob=1.00000000e+00,prob_stddev=0.00000000e+00,prob_range={1.00000000e+00,1.00000000e+00},prob(percent)="100",prob+-sd="100+-0"]:4.565431e-01[&length_mean=4.61062605e-01,length_median=4.56543100e-01,length_95%HPD={3.04355300e-01,6.28601300e-01}])[&prob=9.97168554e-01,prob_stddev=1.66555630e-03,prob_range={9.95336442e-01,9.99333777e-01},prob(percent)="100",prob+-sd="100+-0"]:1.972031e-01[&length_mean=2.01461397e-01,length_median=1.97203100e-01,length_95%HPD={7.37047400e-02,3.36865700e-01}])[&prob=8.97235177e-01,prob_stddev=2.96469021e-02,prob_range={8.65423051e-01,9.25383078e-01},prob(percent)="90",prob+-sd="90+-3"]:1.108496e-01[&length_mean=1.11818470e-01,length_median=1.10849600e-01,length_95%HPD={4.84534800e-02,1.76337800e-01}])[&prob=1.81712192e-01,prob_stddev=8.18442004e-03,prob_range={1.76548967e-01,1.93870753e-01},prob(percent)="18",prob+-sd="18+-1"]:3.443169e-02[&length_mean=3.93741816e-02,length_median=3.44316900e-02,length_95%HPD={2.83341500e-04,9.10899700e-02}],(54[&prob=1.00000000e+00,prob_stddev=0.00000000e+00,prob_range={1.00000000e+00,1.00000000e+00},prob(percent)="100",prob+-sd="100+-0"]:2.556114e-01[&length_mean=2.62342115e-01,length_median=2.55611400e-01,length_95%HPD={1.42450100e-01,3.96737100e-01}],62[&prob=1.00000000e+00,prob_stddev=0.00000000e+00,prob_range={1.00000000e+00,1.00000000e+00},prob(percent)="100",prob+-sd="100+-0"]:1.762613e-01[&length_mean=1.82072456e-01,length_median=1.76261300e-01,length_95%HPD={8.83755100e-02,3.00062500e-01}])[&prob=1.00000000e+00,prob_stddev=0.00000000e+00,prob_range={1.00000000e+00,1.00000000e+00},prob(percent)="100",prob+-sd="100+-0"]:3.688758e-01[&length_mean=3.76590768e-01,length_median=3.68875800e-01,length_95%HPD={2.09968500e-01,5.50807000e-01}])[&prob=5.24816789e-01,prob_stddev=1.46101348e-02,prob_range={5.02998001e-01,5.33644237e-01},prob(percent)="52",prob+-sd="52+-1"]:7.260680e-02[&length_mean=7.37276105e-02,length_median=7.26068000e-02,length_95%HPD={2.09943800e-02,1.27295700e-01}],(29[&prob=1.00000000e+00,prob_stddev=0.00000000e+00,prob_range={1.00000000e+00,1.00000000e+00},prob(percent)="100",prob+-sd="100+-0"]:7.445528e-01[&length_mean=7.52831363e-01,length_median=7.44552800e-01,length_95%HPD={5.75702000e-01,9.48199300e-01}],((((56[&prob=1.00000000e+00,prob_stddev=0.00000000e+00,prob_range={1.00000000e+00,1.00000000e+00},prob(percent)="100",prob+-sd="100+-0"]:4.820800e-01[&length_mean=4.90412889e-01,length_median=4.82080000e-01,length_95%HPD={2.92860900e-01,6.84593700e-01}],59[&prob=1.00000000e+00,prob_stddev=0.00000000e+00,prob_range={1.00000000e+00,1.00000000e+00},prob(percent)="100",prob+-sd="100+-0"]:2.650731e-01[&length_mean=2.69369604e-01,length_median=2.65073100e-01,length_95%HPD={1.48892100e-01,3.95022800e-01}])[&prob=7.85642905e-01,prob_stddev=2.14669467e-02,prob_range={7.59493671e-01,8.07461692e-01},prob(percent)="79",prob+-sd="79+-2"]:6.537761e-02[&length_mean=7.10056861e-02,length_median=6.53776100e-02,length_95%HPD={1.09788100e-02,1.44208100e-01}],70[&prob=1.00000000e+00,prob_stddev=0.00000000e+00,prob_range={1.00000000e+00,1.00000000e+00},prob(percent)="100",prob+-sd="100+-0"]:4.560122e-01[&length_mean=4.64123834e-01,length_median=4.56012200e-01,length_95%HPD={2.88290000e-01,6.61296200e-01}])[&prob=7.68987342e-01,prob_stddev=1.72222065e-02,prob_range={7.44836775e-01,7.83477682e-01},prob(percent)="77",prob+-sd="77+-2"]:8.222952e-02[&length_mean=8.74019541e-02,length_median=8.22295200e-02,length_95%HPD={7.85298100e-03,1.72140900e-01}],((67[&prob=1.00000000e+00,prob_stddev=0.00000000e+00,prob_range={1.00000000e+00,1.00000000e+00},prob(percent)="100",prob+-sd="100+-0"]:4.636216e-02[&length_mean=5.55549902e-02,length_median=4.63621600e-02,length_95%HPD={3.03233800e-05,1.34154100e-01}],73[&prob=1.00000000e+00,prob_stddev=0.00000000e+00,prob_range={1.00000000e+00,1.00000000e+00},prob(percent)="100",prob+-sd="100+-0"]:2.433597e-01[&length_mean=2.58102691e-01,length_median=2.43359700e-01,length_95%HPD={8.63432200e-02,4.61476700e-01}])[&prob=9.65356429e-01,prob_stddev=6.41329295e-03,prob_range={9.57361759e-01,9.72684877e-01},prob(percent)="97",prob+-sd="97+-1"]:9.969916e-02[&length_mean=1.06936705e-01,length_median=9.96991600e-02,length_95%HPD={7.51132300e-03,2.14361400e-01}],72[&prob=1.00000000e+00,prob_stddev=0.00000000e+00,prob_range={1.00000000e+00,1.00000000e+00},prob(percent)="100",prob+-sd="100+-0"]:3.260622e-01[&length_mean=3.41731170e-01,length_median=3.26062200e-01,length_95%HPD={1.31392800e-01,5.93617000e-01}])[&prob=9.96502332e-01,prob_stddev=1.26113887e-03,prob_range={9.94670220e-01,9.97335110e-01},prob(percent)="100",prob+-sd="100+-0"]:1.357403e-01[&length_mean=1.43130930e-01,length_median=1.35740300e-01,length_95%HPD={3.15493500e-02,2.72094500e-01}])[&prob=1.00000000e+00,prob_stddev=0.00000000e+00,prob_range={1.00000000e+00,1.00000000e+00},prob(percent)="100",prob+-sd="100+-0"]:3.324607e-01[&length_mean=3.38319482e-01,length_median=3.32460700e-01,length_95%HPD={1.88237500e-01,4.86830100e-01}],65[&prob=1.00000000e+00,prob_stddev=0.00000000e+00,prob_range={1.00000000e+00,1.00000000e+00},prob(percent)="100",prob+-sd="100+-0"]:5.909023e-01[&length_mean=5.99218368e-01,length_median=5.90902300e-01,length_95%HPD={3.69516600e-01,8.32226100e-01}])[&prob=9.33877415e-01,prob_stddev=1.18227126e-02,prob_range={9.16722185e-01,9.42038641e-01},prob(percent)="93",prob+-sd="93+-1"]:1.063232e-01[&length_mean=1.11682266e-01,length_median=1.06323200e-01,length_95%HPD={3.13088000e-02,2.06513700e-01}])[&prob=9.96169221e-01,prob_stddev=4.49803000e-03,prob_range={9.91339107e-01,1.00000000e+00},prob(percent)="100",prob+-sd="100+-0"]:1.611165e-01[&length_mean=1.63631324e-01,length_median=1.61116500e-01,length_95%HPD={7.09713700e-02,2.59643100e-01}])[&prob=1.00000000e+00,prob_stddev=0.00000000e+00,prob_range={1.00000000e+00,1.00000000e+00},prob(percent)="100",prob+-sd="100+-0"]:2.695241e-01[&length_mean=2.71722932e-01,length_median=2.69524100e-01,length_95%HPD={1.59759500e-01,3.88452600e-01}],(30[&prob=1.00000000e+00,prob_stddev=0.00000000e+00,prob_range={1.00000000e+00,1.00000000e+00},prob(percent)="100",prob+-sd="100+-0"]:3.319322e-01[&length_mean=3.34440389e-01,length_median=3.31932200e-01,length_95%HPD={2.40059100e-01,4.41081300e-01}],((74[&prob=1.00000000e+00,prob_stddev=0.00000000e+00,prob_range={1.00000000e+00,1.00000000e+00},prob(percent)="100",prob+-sd="100+-0"]:3.268652e-01[&length_mean=3.29777916e-01,length_median=3.26865200e-01,length_95%HPD={1.94271300e-01,4.61888700e-01}],78[&prob=1.00000000e+00,prob_stddev=0.00000000e+00,prob_range={1.00000000e+00,1.00000000e+00},prob(percent)="100",prob+-sd="100+-0"]:1.753662e-01[&length_mean=1.88981409e-01,length_median=1.75366200e-01,length_95%HPD={4.66184700e-02,3.60165600e-01}])[&prob=6.50233178e-01,prob_stddev=8.72047698e-03,prob_range={6.38907395e-01,6.59560293e-01},prob(percent)="65",prob+-sd="65+-1"]:5.003771e-02[&length_mean=5.83576389e-02,length_median=5.00377100e-02,length_95%HPD={2.12666700e-05,1.36841000e-01}],((75[&prob=1.00000000e+00,prob_stddev=0.00000000e+00,prob_range={1.00000000e+00,1.00000000e+00},prob(percent)="100",prob+-sd="100+-0"]:1.871830e-01[&length_mean=1.93957799e-01,length_median=1.87183000e-01,length_95%HPD={6.72579600e-02,3.32772400e-01}],77[&prob=1.00000000e+00,prob_stddev=0.00000000e+00,prob_range={1.00000000e+00,1.00000000e+00},prob(percent)="100",prob+-sd="100+-0"]:2.793213e-01[&length_mean=2.95298605e-01,length_median=2.79321300e-01,length_95%HPD={1.06357800e-01,5.06951300e-01}])[&prob=3.96735510e-01,prob_stddev=1.93510597e-02,prob_range={3.71085943e-01,4.17721519e-01},prob(percent)="40",prob+-sd="40+-2"]:3.076541e-02[&length_mean=4.17448275e-02,length_median=3.07654100e-02,length_95%HPD={1.22556800e-05,1.18698100e-01}],76[&prob=1.00000000e+00,prob_stddev=0.00000000e+00,prob_range={1.00000000e+00,1.00000000e+00},prob(percent)="100",prob+-sd="100+-0"]:1.112980e-01[&length_mean=1.22341719e-01,length_median=1.11298000e-01,length_95%HPD={1.68615300e-02,2.49477100e-01}])[&prob=9.99333777e-01,prob_stddev=9.42180921e-04,prob_range={9.98001332e-01,1.00000000e+00},prob(percent)="100",prob+-sd="100+-0"]:1.577308e-01[&length_mean=1.65507109e-01,length_median=1.57730800e-01,length_95%HPD={4.11199200e-02,2.99116500e-01}])[&prob=8.78081279e-01,prob_stddev=1.42370157e-02,prob_range={8.63424384e-01,8.96069287e-01},prob(percent)="88",prob+-sd="88+-1"]:6.778173e-02[&length_mean=7.29226736e-02,length_median=6.77817300e-02,length_95%HPD={7.69769300e-03,1.49362300e-01}])[&prob=1.00000000e+00,prob_stddev=0.00000000e+00,prob_range={1.00000000e+00,1.00000000e+00},prob(percent)="100",prob+-sd="100+-0"]:6.412928e-01[&length_mean=6.45906991e-01,length_median=6.41292800e-01,length_95%HPD={4.77306900e-01,8.18779800e-01}])[&prob=1.00000000e+00,prob_stddev=0.00000000e+00,prob_range={1.00000000e+00,1.00000000e+00},prob(percent)="100",prob+-sd="100+-0"]:2.359392e-01[&length_mean=2.40264237e-01,length_median=2.35939200e-01,length_95%HPD={1.43866400e-01,3.43915900e-01}])[&prob=1.00000000e+00,prob_stddev=0.00000000e+00,prob_range={1.00000000e+00,1.00000000e+00},prob(percent)="100",prob+-sd="100+-0"]:5.930270e-01[&length_mean=6.00103358e-01,length_median=5.93027000e-01,length_95%HPD={4.05177000e-01,8.00361700e-01}])[&prob=8.83910726e-01,prob_stddev=1.19348082e-02,prob_range={8.66089274e-01,8.91405730e-01},prob(percent)="88",prob+-sd="88+-1"]:2.726232e-01[&length_mean=2.79598713e-01,length_median=2.72623200e-01,length_95%HPD={1.30160300e-01,4.37079300e-01}])[&prob=1.00000000e+00,prob_stddev=0.00000000e+00,prob_range={1.00000000e+00,1.00000000e+00},prob(percent)="100",prob+-sd="100+-0"]:1.080133e+00[&length_mean=1.09514776e+00,length_median=1.08013300e+00,length_95%HPD={7.55292600e-01,1.44071300e+00}])[&prob=7.40672885e-01,prob_stddev=1.32506841e-02,prob_range={7.26848767e-01,7.56828781e-01},prob(percent)="74",prob+-sd="74+-1"]:3.092500e-01[&length_mean=3.16319796e-01,length_median=3.09250000e-01,length_95%HPD={1.54160400e-01,4.99148400e-01}],(5[&prob=1.00000000e+00,prob_stddev=0.00000000e+00,prob_range={1.00000000e+00,1.00000000e+00},prob(percent)="100",prob+-sd="100+-0"]:8.124269e-01[&length_mean=8.22870084e-01,length_median=8.12426900e-01,length_95%HPD={5.54050000e-01,1.11904900e+00}],6[&prob=1.00000000e+00,prob_stddev=0.00000000e+00,prob_range={1.00000000e+00,1.00000000e+00},prob(percent)="100",prob+-sd="100+-0"]:7.902665e-01[&length_mean=8.01772326e-01,length_median=7.90266500e-01,length_95%HPD={5.38870000e-01,1.10713400e+00}])[&prob=1.00000000e+00,prob_stddev=0.00000000e+00,prob_range={1.00000000e+00,1.00000000e+00},prob(percent)="100",prob+-sd="100+-0"]:1.171015e+00[&length_mean=1.18215037e+00,length_median=1.17101500e+00,length_95%HPD={8.24298400e-01,1.56138200e+00}])[&prob=1.00000000e+00,prob_stddev=0.00000000e+00,prob_range={1.00000000e+00,1.00000000e+00},prob(percent)="100",prob+-sd="100+-0"]:9.541608e-01[&length_mean=9.69452332e-01,length_median=9.54160800e-01,length_95%HPD={6.29944600e-01,1.31566400e+00}],(2[&prob=1.00000000e+00,prob_stddev=0.00000000e+00,prob_range={1.00000000e+00,1.00000000e+00},prob(percent)="100",prob+-sd="100+-0"]:2.012559e+00[&length_mean=2.04370359e+00,length_median=2.01255900e+00,length_95%HPD={1.51386900e+00,2.67514300e+00}],3[&prob=1.00000000e+00,prob_stddev=0.00000000e+00,prob_range={1.00000000e+00,1.00000000e+00},prob(percent)="100",prob+-sd="100+-0"]:1.873410e+00[&length_mean=1.89715641e+00,length_median=1.87341000e+00,length_95%HPD={1.36696700e+00,2.43242300e+00}])[&prob=1.00000000e+00,prob_stddev=0.00000000e+00,prob_range={1.00000000e+00,1.00000000e+00},prob(percent)="100",prob+-sd="100+-0"]:5.188540e-01[&length_mean=5.29126660e-01,length_median=5.18854000e-01,length_95%HPD={2.80254300e-01,8.07540800e-01}]);

end;

**Total evidence – undated Bayesian analysis (50% majority rule consensus)**

***
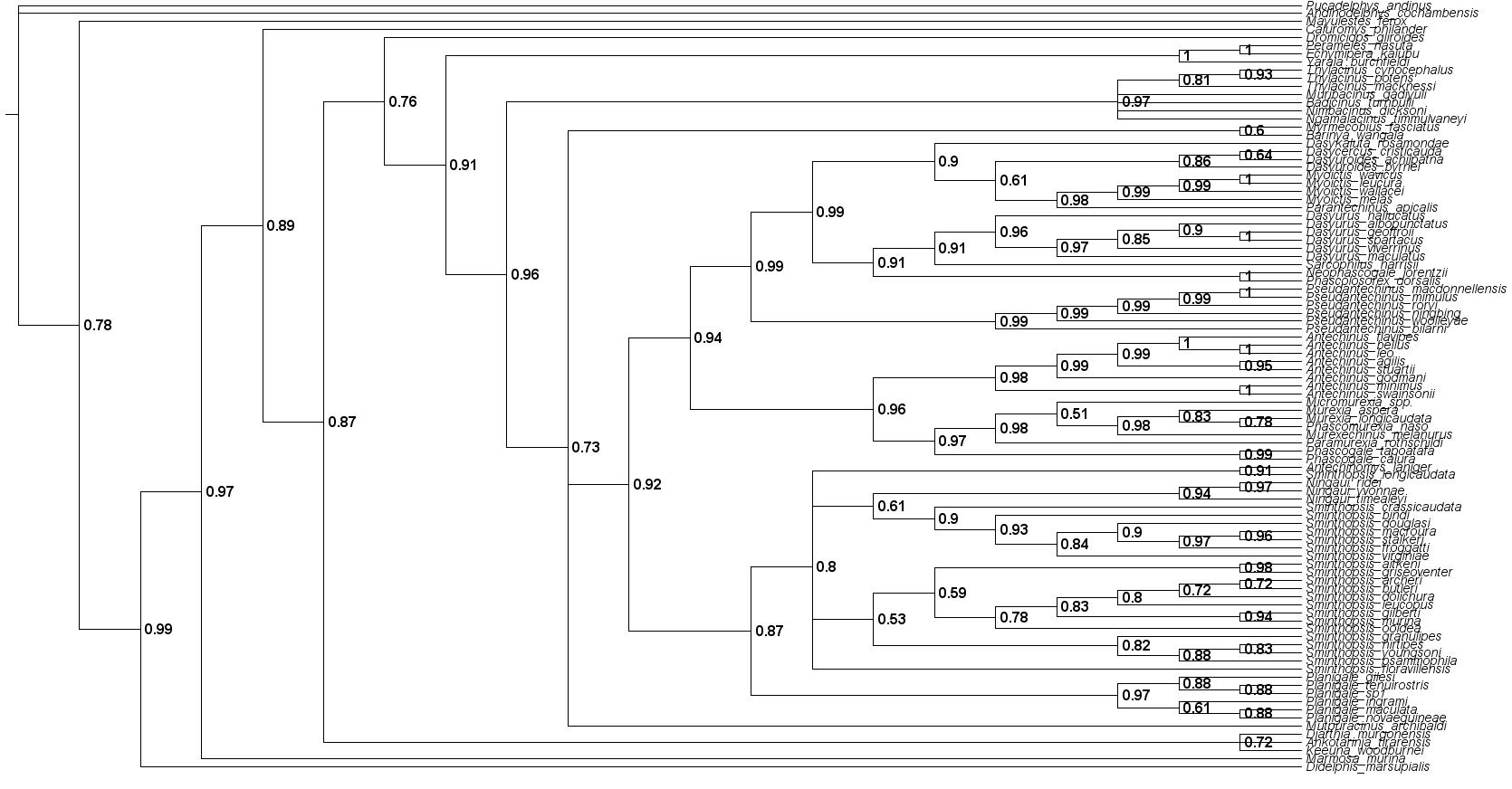
***

#NEXUS

begin taxa;

dimensions ntax=95;

taxlabels

Pucadelphys_andinus

Andinodelphys_cochambensis

Mayulestes_ferox

Caluromys_philander

Didelphis_marsupialis

Marmosa_murina

Dromiciops_gliroides

Perameles_nasuta

Echymipera_kalubu

Thylacinus_cynocephalus

Myrmecobius_fasciatus

Dasykaluta_rosamondae

Dasycercus_cristicauda

Dasyuroides_byrnei

Dasyurus_hallucatus

Myoictis_wavicus

Neophascogale_lorentzii

Phascolosorex_dorsalis

Parantechinus_apicalis

Pseudantechinus_macdonnellensis

Sarcophilus_harrisii

Antechinus_flavipes

Micromurexia_spp.

Murexia_aspera

Murexia_longicaudata

Murexechinus_melanurus

Phascomurexia_naso

Paramurexia_rothschildi

Phascogale_tapoatafa

Antechinomys_laniger

Ningaui_ridei

Sminthopsis_crassicaudata

Planigale_gilesi

Dasyurus_albopunctatus

Dasyurus_geoffroii

Dasyurus_maculatus

Dasyurus_spartacus

Dasyurus_viverrinus

Myoictis_leucura

Myoictis_melas

Myoictis_wallacei

Pseudantechinus_bilarni

Pseudantechinus_mimulus

Pseudantechinus_ningbing

Pseudantechinus_roryi

Pseudantechinus_woolleyae

Antechinus_agilis

Antechinus_bellus

Antechinus_godmani

Antechinus_leo

Antechinus_minimus

Antechinus_stuartii

Antechinus_swainsonii

Phascogale_calura

Ningaui_timealeyi

Ningaui_yvonnae

Sminthopsis_aitkeni

Sminthopsis_archeri

Sminthopsis_bindi

Sminthopsis_butleri

Sminthopsis_dolichura

Sminthopsis_douglasi

Sminthopsis_gilberti

Sminthopsis_granulipes

Sminthopsis_griseoventer

Sminthopsis_hirtipes

Sminthopsis_leucopus

Sminthopsis_longicaudata

Sminthopsis_murina

Sminthopsis_macroura

Sminthopsis_psammophila

Sminthopsis_ooldea

Sminthopsis_virginiae

Sminthopsis_youngsoni

Sminthopsis_froggatti

Sminthopsis_stalkeri

Planigale_ingrami

Planigale_maculata

Planigale_novaeguineae

Planigale_tenuirostris

Planigale_sp1

Djarthia_murgonensis

Ankotarinja_tirarensis

Keeuna_woodburnei

Yarala_burchfieldi

Mutpuracinus_archibaldi

Barinya_wangala

Sminthopsis_floravillensis

Dasyuroides_achilpatna

Muribacinus_gadiyuli

Badjcinus_turnbulli

Nimbacinus_dicksoni

Thylacinus_macknessi

Thylacinus_potens

Ngamalacinus_timmulvaneyi

;

end;

begin trees;

translate

1 Pucadelphys_andinus,

2 Andinodelphys_cochambensis,

3 Mayulestes_ferox,

4 Caluromys_philander,

5 Didelphis_marsupialis,

6 Marmosa_murina,

7 Dromiciops_gliroides,

8 Perameles_nasuta,

9 Echymipera_kalubu,

10 Thylacinus_cynocephalus,

11 Myrmecobius_fasciatus,

12 Dasykaluta_rosamondae,

13 Dasycercus_cristicauda,

14 Dasyuroides_byrnei,

15 Dasyurus_hallucatus,

16 Myoictis_wavicus,

17 Neophascogale_lorentzii,

18 Phascolosorex_dorsalis,

19 Parantechinus_apicalis,

20 Pseudantechinus_macdonnellensis,

21 Sarcophilus_harrisii,

22 Antechinus_flavipes,

23 Micromurexia_spp.,

24 Murexia_aspera,

25 Murexia_longicaudata,

26 Murexechinus_melanurus,

27 Phascomurexia_naso,

28 Paramurexia_rothschildi,

29 Phascogale_tapoatafa,

30 Antechinomys_laniger,

31 Ningaui_ridei,

32 Sminthopsis_crassicaudata,

33 Planigale_gilesi,

34 Dasyurus_albopunctatus,

35 Dasyurus_geoffroii,

36 Dasyurus_maculatus,

37 Dasyurus_spartacus,

38 Dasyurus_viverrinus,

39 Myoictis_leucura,

40 Myoictis_melas,

41 Myoictis_wallacei,

42 Pseudantechinus_bilarni,

43 Pseudantechinus_mimulus,

44 Pseudantechinus_ningbing,

45 Pseudantechinus_roryi,

46 Pseudantechinus_woolleyae,

47 Antechinus_agilis,

48 Antechinus_bellus,

49 Antechinus_godmani,

50 Antechinus_leo,

51 Antechinus_minimus,

52 Antechinus_stuartii,

53 Antechinus_swainsonii,

54 Phascogale_calura,

55 Ningaui_timealeyi,

56 Ningaui_yvonnae,

57 Sminthopsis_aitkeni,

58 Sminthopsis_archeri,

59 Sminthopsis_bindi,

60 Sminthopsis_butleri,

61 Sminthopsis_dolichura,

62 Sminthopsis_douglasi,

63 Sminthopsis_gilberti,

64 Sminthopsis_granulipes,

65 Sminthopsis_griseoventer,

66 Sminthopsis_hirtipes,

67 Sminthopsis_leucopus,

68 Sminthopsis_longicaudata,

69 Sminthopsis_murina,

70 Sminthopsis_macroura,

71 Sminthopsis_psammophila,

72 Sminthopsis_ooldea,

73 Sminthopsis_virginiae,

74 Sminthopsis_youngsoni,

75 Sminthopsis_froggatti,

76 Sminthopsis_stalkeri,

77 Planigale_ingrami,

78 Planigale_maculata,

79 Planigale_novaeguineae,

80 Planigale_tenuirostris,

81 Planigale_sp1,

82 Djarthia_murgonensis,

83 Ankotarinja_tirarensis,

84 Keeuna_woodburnei,

85 Yarala_burchfieldi,

86 Mutpuracinus_archibaldi,

87 Barinya_wangala,

88 Sminthopsis_floravillensis,

89 Dasyuroides_achilpatna,

90 Muribacinus_gadiyuli,

91 Badjcinus_turnbulli,

92 Nimbacinus_dicksoni,

93 Thylacinus_macknessi,

94 Thylacinus_potens,

95 Ngamalacinus_timmulvaneyi

;

tree con_50_majrule = [&U] (1[&prob=1.00000000e+000,prob_stddev=0.00000000e+000,prob_range={1.00000000e+000,1.00000000e+000},prob(percent)="100",prob+-sd="100+-0"]:6.238860e-002[&length_mean=8.69016410e-002,length_median=6.23886000e-002,length_95%HPD={6.95686300e-006,2.53985700e-001}],2[&prob=1.00000000e+000,prob_stddev=0.00000000e+000,prob_range={1.00000000e+000,1.00000000e+000},prob(percent)="100",prob+-sd="100+-0"]:5.076471e-002[&length_mean=7.71074121e-002,length_median=5.07647100e-002,length_95%HPD={9.92368300e-006,2.39807600e-001}],(3[&prob=1.00000000e+000,prob_stddev=0.00000000e+000,prob_range={1.00000000e+000,1.00000000e+000},prob(percent)="100",prob+-sd="100+-0"]:8.053418e-001[&length_mean=8.36197142e-001,length_median=8.05341800e-001,length_95%HPD={3.38651400e-001,1.36944100e+000}],(((4[&prob=1.00000000e+000,prob_stddev=0.00000000e+000,prob_range={1.00000000e+000,1.00000000e+000},prob(percent)="100",prob+-sd="100+-0"]:8.734891e-001[&length_mean=8.81781243e-001,length_median=8.73489100e-001,length_95%HPD={6.65573900e-001,1.11246900e+000}],((7[&prob=1.00000000e+000,prob_stddev=0.00000000e+000,prob_range={1.00000000e+000,1.00000000e+000},prob(percent)="100",prob+-sd="100+-0"]:1.470309e+000[&length_mean=1.44776204e+000,length_median=1.47030900e+000,length_95%HPD={8.95136500e-001,1.92809500e+000}],(((8[&prob=1.00000000e+000,prob_stddev=0.00000000e+000,prob_range={1.00000000e+000,1.00000000e+000},prob(percent)="100",prob+-sd="100+-0"]:3.820213e-001[&length_mean=3.85892207e-001,length_median=3.82021300e-001,length_95%HPD={2.86641200e-001,4.95819600e-001}],9[&prob=1.00000000e+000,prob_stddev=0.00000000e+000,prob_range={1.00000000e+000,1.00000000e+000},prob(percent)="100",prob+-sd="100+-0"]:4.377722e-001[&length_mean=4.40732760e-001,length_median=4.37772200e-001,length_95%HPD={3.27654100e-001,5.58286400e-001}])[&prob=1.00000000e+000,prob_stddev=0.00000000e+000,prob_range={1.00000000e+000,1.00000000e+000},prob(percent)="100",prob+-sd="100+-0"]:9.486455e-001[&length_mean=9.51786521e-001,length_median=9.48645500e-001,length_95%HPD={4.31139200e-001,1.46160600e+000}],85[&prob=1.00000000e+000,prob_stddev=0.00000000e+000,prob_range={1.00000000e+000,1.00000000e+000},prob(percent)="100",prob+-sd="100+-0"]:2.183996e-001[&length_mean=2.71286431e-001,length_median=2.18399600e-001,length_95%HPD={6.61065500e-007,6.82876600e-001}])[&prob=9.99276620e-001,prob_stddev=1.09546843e-003,prob_range={9.97685185e-001,1.00000000e+000},prob(percent)="100",prob+-sd="100+-0"]:7.159347e-001[&length_mean=7.28973149e-001,length_median=7.15934700e-001,length_95%HPD={2.34248800e-001,1.23828600e+000}],((((10[&prob=1.00000000e+000,prob_stddev=0.00000000e+000,prob_range={1.00000000e+000,1.00000000e+000},prob(percent)="100",prob+-sd="100+-0"]:1.366973e-001[&length_mean=1.64905868e-001,length_median=1.36697300e-001,length_95%HPD={1.70854300e-004,4.05735900e-001}],94[&prob=1.00000000e+000,prob_stddev=0.00000000e+000,prob_range={1.00000000e+000,1.00000000e+000},prob(percent)="100",prob+-sd="100+-0"]:9.244350e-002[&length_mean=1.35899218e-001,length_median=9.24435000e-002,length_95%HPD={1.43902500e-005,4.07052500e-001}])[&prob=9.32581019e-001,prob_stddev=8.43267477e-003,prob_range={9.23611111e-001,9.43287037e-001},prob(percent)="93",prob+-sd="93+-1"]:3.495854e-001[&length_mean=3.72841407e-001,length_median=3.49585400e-001,length_95%HPD={4.70477400e-002,7.66232700e-001}],93[&prob=1.00000000e+000,prob_stddev=0.00000000e+000,prob_range={1.00000000e+000,1.00000000e+000},prob(percent)="100",prob+-sd="100+-0"]:2.585367e-001[&length_mean=3.22045877e-001,length_median=2.58536700e-001,length_95%HPD={5.26107500e-005,8.34784400e-001}])[&prob=8.12500000e-001,prob_stddev=3.40633213e-002,prob_range={7.67939815e-001,8.40277778e-001},prob(percent)="81",prob+-sd="81+-3"]:2.927719e-001[&length_mean=3.27382409e-001,length_median=2.92771900e-001,length_95%HPD={8.56902100e-003,6.95365900e-001}],90[&prob=1.00000000e+000,prob_stddev=0.00000000e+000,prob_range={1.00000000e+000,1.00000000e+000},prob(percent)="100",prob+-sd="100+-0"]:8.458662e-002[&length_mean=1.22340370e-001,length_median=8.45866200e-002,length_95%HPD={1.87192300e-005,3.66560200e-001}],91[&prob=1.00000000e+000,prob_stddev=0.00000000e+000,prob_range={1.00000000e+000,1.00000000e+000},prob(percent)="100",prob+-sd="100+-0"]:2.963946e-001[&length_mean=3.35395747e-001,length_median=2.96394600e-001,length_95%HPD={1.46184100e-002,7.31290600e-001}],92[&prob=1.00000000e+000,prob_stddev=0.00000000e+000,prob_range={1.00000000e+000,1.00000000e+000},prob(percent)="100",prob+-sd="100+-0"]:1.275709e-001[&length_mean=1.65299734e-001,length_median=1.27570900e-001,length_95%HPD={3.22350900e-005,4.40669400e-001}],95[&prob=1.00000000e+000,prob_stddev=0.00000000e+000,prob_range={1.00000000e+000,1.00000000e+000},prob(percent)="100",prob+-sd="100+-0"]:1.325014e-001[&length_mean=1.73525550e-001,length_median=1.32501400e-001,length_95%HPD={6.27457900e-005,4.72937200e-001}])[&prob=9.66001157e-001,prob_stddev=1.35831100e-002,prob_range={9.53125000e-001,9.78587963e-001},prob(percent)="97",prob+-sd="97+-1"]:4.906336e-001[&length_mean=5.11896266e-001,length_median=4.90633600e-001,length_95%HPD={9.34505300e-002,9.40592300e-001}],((11[&prob=1.00000000e+000,prob_stddev=0.00000000e+000,prob_range={1.00000000e+000,1.00000000e+000},prob(percent)="100",prob+-sd="100+-0"]:1.142193e+000[&length_mean=1.12982101e+000,length_median=1.14219300e+000,length_95%HPD={6.48688900e-001,1.57065100e+000}],87[&prob=1.00000000e+000,prob_stddev=0.00000000e+000,prob_range={1.00000000e+000,1.00000000e+000},prob(percent)="100",prob+-sd="100+-0"]:2.084050e-001[&length_mean=2.44379468e-001,length_median=2.08405000e-001,length_95%HPD={4.94099300e-004,5.85080900e-001}])[&prob=6.01128472e-001,prob_stddev=3.64487636e-002,prob_range={5.52662037e-001,6.36574074e-001},prob(percent)="60",prob+-sd="60+-4"]:2.308802e-001[&length_mean=2.60271353e-001,length_median=2.30880200e-001,length_95%HPD={2.31278500e-004,5.89422900e-001}],(((((12[&prob=1.00000000e+000,prob_stddev=0.00000000e+000,prob_range={1.00000000e+000,1.00000000e+000},prob(percent)="100",prob+-sd="100+-0"]:4.067252e-001[&length_mean=4.08734133e-001,length_median=4.06725200e-001,length_95%HPD={3.24416700e-001,5.13561500e-001}],(((13[&prob=1.00000000e+000,prob_stddev=0.00000000e+000,prob_range={1.00000000e+000,1.00000000e+000},prob(percent)="100",prob+-sd="100+-0"]:2.008248e-001[&length_mean=2.00062331e-001,length_median=2.00824800e-001,length_95%HPD={5.66338800e-005,3.75716800e-001}],89[&prob=1.00000000e+000,prob_stddev=0.00000000e+000,prob_range={1.00000000e+000,1.00000000e+000},prob(percent)="100",prob+-sd="100+-0"]:1.044251e-001[&length_mean=1.56393102e-001,length_median=1.04425100e-001,length_95%HPD={6.91393400e-006,4.73309500e-001}])[&prob=6.41059028e-001,prob_stddev=4.19772988e-002,prob_range={5.99537037e-001,6.99074074e-001},prob(percent)="64",prob+-sd="64+-4"]:2.158100e-001[&length_mean=2.05276049e-001,length_median=2.15810000e-001,length_95%HPD={1.07121600e-002,3.49011100e-001}],14[&prob=1.00000000e+000,prob_stddev=0.00000000e+000,prob_range={1.00000000e+000,1.00000000e+000},prob(percent)="100",prob+-sd="100+-0"]:3.173994e-001[&length_mean=3.03829579e-001,length_median=3.17399400e-001,length_95%HPD={1.22255500e-001,4.24869100e-001}])[&prob=8.55613426e-001,prob_stddev=4.91784664e-002,prob_range={8.17129630e-001,9.27662037e-001},prob(percent)="86",prob+-sd="86+-5"]:8.600278e-002[&length_mean=8.64966544e-002,length_median=8.60027800e-002,length_95%HPD={5.47306000e-002,1.22326100e-001}],((((16[&prob=1.00000000e+000,prob_stddev=0.00000000e+000,prob_range={1.00000000e+000,1.00000000e+000},prob(percent)="100",prob+-sd="100+-0"]:9.563931e-002[&length_mean=9.73058631e-002,length_median=9.56393100e-002,length_95%HPD={5.74711000e-002,1.38309200e-001}],39[&prob=1.00000000e+000,prob_stddev=0.00000000e+000,prob_range={1.00000000e+000,1.00000000e+000},prob(percent)="100",prob+-sd="100+-0"]:7.627040e-002[&length_mean=7.80693376e-002,length_median=7.62704000e-002,length_95%HPD={4.34406900e-002,1.20093400e-001}])[&prob=9.96527778e-001,prob_stddev=4.65367982e-003,prob_range={9.90162037e-001,1.00000000e+000},prob(percent)="100",prob+-sd="100+-0"]:8.762869e-002[&length_mean=8.94389614e-002,length_median=8.76286900e-002,length_95%HPD={4.86226800e-002,1.31589500e-001}],41[&prob=1.00000000e+000,prob_stddev=0.00000000e+000,prob_range={1.00000000e+000,1.00000000e+000},prob(percent)="100",prob+-sd="100+-0"]:1.652300e-001[&length_mean=1.66428047e-001,length_median=1.65230000e-001,length_95%HPD={1.12042400e-001,2.25600500e-001}])[&prob=9.90306713e-001,prob_stddev=1.12152619e-002,prob_range={9.79745370e-001,1.00000000e+000},prob(percent)="99",prob+-sd="99+-1"]:5.758118e-002[&length_mean=5.88547853e-002,length_median=5.75811800e-002,length_95%HPD={2.65506200e-002,9.20146500e-002}],40[&prob=1.00000000e+000,prob_stddev=0.00000000e+000,prob_range={1.00000000e+000,1.00000000e+000},prob(percent)="100",prob+-sd="100+-0"]:1.948168e-001[&length_mean=1.96453293e-001,length_median=1.94816800e-001,length_95%HPD={1.39171900e-001,2.58944700e-001}])[&prob=9.89872685e-001,prob_stddev=1.17321383e-002,prob_range={9.78587963e-001,1.00000000e+000},prob(percent)="99",prob+-sd="99+-1"]:1.577020e-001[&length_mean=1.59267601e-001,length_median=1.57702000e-001,length_95%HPD={1.09800600e-001,2.12747000e-001}],19[&prob=1.00000000e+000,prob_stddev=0.00000000e+000,prob_range={1.00000000e+000,1.00000000e+000},prob(percent)="100",prob+-sd="100+-0"]:3.978784e-001[&length_mean=4.00862471e-001,length_median=3.97878400e-001,length_95%HPD={3.12062700e-001,4.95861500e-001}])[&prob=9.76851852e-001,prob_stddev=1.83002179e-002,prob_range={9.57175926e-001,9.98842593e-001},prob(percent)="98",prob+-sd="98+-2"]:4.483208e-002[&length_mean=4.57497290e-002,length_median=4.48320800e-002,length_95%HPD={2.42091200e-002,6.92429200e-002}])[&prob=6.10098380e-001,prob_stddev=1.19664863e-002,prob_range={5.97800926e-001,6.23263889e-001},prob(percent)="61",prob+-sd="61+-1"]:1.903894e-002[&length_mean=1.98368522e-002,length_median=1.90389400e-002,length_95%HPD={7.45035200e-003,3.39527000e-002}])[&prob=8.96412037e-001,prob_stddev=2.88695243e-002,prob_range={8.71527778e-001,9.38078704e-001},prob(percent)="90",prob+-sd="90+-3"]:3.817714e-002[&length_mean=3.90577443e-002,length_median=3.81771400e-002,length_95%HPD={2.14008700e-002,5.78341400e-002}],(((15[&prob=1.00000000e+000,prob_stddev=0.00000000e+000,prob_range={1.00000000e+000,1.00000000e+000},prob(percent)="100",prob+-sd="100+-0"]:4.091299e-001[&length_mean=4.12101945e-001,length_median=4.09129900e-001,length_95%HPD={3.25982500e-001,5.09727700e-001}],(((34[&prob=1.00000000e+000,prob_stddev=0.00000000e+000,prob_range={1.00000000e+000,1.00000000e+000},prob(percent)="100",prob+-sd="100+-0"]:1.982118e-001[&length_mean=1.99558712e-001,length_median=1.98211800e-001,length_95%HPD={1.38981600e-001,2.63533100e-001}],(35[&prob=1.00000000e+000,prob_stddev=0.00000000e+000,prob_range={1.00000000e+000,1.00000000e+000},prob(percent)="100",prob+-sd="100+-0"]:4.953117e-002[&length_mean=5.08026800e-002,length_median=4.95311700e-002,length_95%HPD={2.53783700e-002,7.83657400e-002}],37[&prob=1.00000000e+000,prob_stddev=0.00000000e+000,prob_range={1.00000000e+000,1.00000000e+000},prob(percent)="100",prob+-sd="100+-0"]:8.876657e-002[&length_mean=9.09593167e-002,length_median=8.87665700e-002,length_95%HPD={5.20042200e-002,1.30224300e-001}])[&prob=9.95370370e-001,prob_stddev=3.59852587e-003,prob_range={9.91319444e-001,9.99421296e-001},prob(percent)="100",prob+-sd="100+-0"]:1.108366e-001[&length_mean=1.12130173e-001,length_median=1.10836600e-001,length_95%HPD={7.21600300e-002,1.55527300e-001}])[&prob=9.00028935e-001,prob_stddev=1.71955519e-002,prob_range={8.83680556e-001,9.20717593e-001},prob(percent)="90",prob+-sd="90+-2"]:3.203439e-002[&length_mean=3.31104365e-002,length_median=3.20343900e-002,length_95%HPD={1.32674500e-002,5.54022200e-002}],38[&prob=1.00000000e+000,prob_stddev=0.00000000e+000,prob_range={1.00000000e+000,1.00000000e+000},prob(percent)="100",prob+-sd="100+-0"]:1.662579e-001[&length_mean=1.68607794e-001,length_median=1.66257900e-001,length_95%HPD={1.11131400e-001,2.29707300e-001}])[&prob=8.54166667e-001,prob_stddev=1.67391151e-002,prob_range={8.38541667e-001,8.77893519e-001},prob(percent)="85",prob+-sd="85+-2"]:1.579258e-002[&length_mean=1.71731869e-002,length_median=1.57925800e-002,length_95%HPD={1.37041600e-003,3.43332100e-002}],36[&prob=1.00000000e+000,prob_stddev=0.00000000e+000,prob_range={1.00000000e+000,1.00000000e+000},prob(percent)="100",prob+-sd="100+-0"]:2.606631e-001[&length_mean=2.62902362e-001,length_median=2.60663100e-001,length_95%HPD={1.93386500e-001,3.36088500e-001}])[&prob=9.66145833e-001,prob_stddev=2.15833984e-002,prob_range={9.43287037e-001,9.94791667e-001},prob(percent)="97",prob+-sd="97+-2"]:7.294877e-002[&length_mean=7.39637737e-002,length_median=7.29487700e-002,length_95%HPD={4.40007700e-002,1.03405200e-001}])[&prob=9.61950231e-001,prob_stddev=2.75046654e-002,prob_range={9.28240741e-001,9.94791667e-001},prob(percent)="96",prob+-sd="96+-3"]:7.210631e-002[&length_mean=7.29560982e-002,length_median=7.21063100e-002,length_95%HPD={4.63527900e-002,1.01485700e-001}],21[&prob=1.00000000e+000,prob_stddev=0.00000000e+000,prob_range={1.00000000e+000,1.00000000e+000},prob(percent)="100",prob+-sd="100+-0"]:4.173472e-001[&length_mean=4.16624765e-001,length_median=4.17347200e-001,length_95%HPD={3.25010700e-001,5.50093500e-001}])[&prob=9.14062500e-001,prob_stddev=2.69309745e-002,prob_range={8.78472222e-001,9.43865741e-001},prob(percent)="91",prob+-sd="91+-3"]:7.247733e-002[&length_mean=7.33363442e-002,length_median=7.24773300e-002,length_95%HPD={4.83222400e-002,1.00140500e-001}],(17[&prob=1.00000000e+000,prob_stddev=0.00000000e+000,prob_range={1.00000000e+000,1.00000000e+000},prob(percent)="100",prob+-sd="100+-0"]:2.437182e-001[&length_mean=2.45946739e-001,length_median=2.43718200e-001,length_95%HPD={1.89908300e-001,3.04443400e-001}],18[&prob=1.00000000e+000,prob_stddev=0.00000000e+000,prob_range={1.00000000e+000,1.00000000e+000},prob(percent)="100",prob+-sd="100+-0"]:2.603525e-001[&length_mean=2.62746229e-001,length_median=2.60352500e-001,length_95%HPD={2.05169600e-001,3.23020900e-001}])[&prob=9.99276620e-001,prob_stddev=1.44675926e-003,prob_range={9.97106481e-001,1.00000000e+000},prob(percent)="100",prob+-sd="100+-0"]:2.172577e-001[&length_mean=2.19634864e-001,length_median=2.17257700e-001,length_95%HPD={1.69066000e-001,2.75193100e-001}])[&prob=9.11313657e-001,prob_stddev=2.83953304e-002,prob_range={8.73842593e-001,9.42708333e-001},prob(percent)="91",prob+-sd="91+-3"]:7.436448e-002[&length_mean=7.54385476e-002,length_median=7.43644800e-002,length_95%HPD={5.08707900e-002,1.01707100e-001}])[&prob=9.85098380e-001,prob_stddev=1.32502930e-002,prob_range={9.72800926e-001,9.97685185e-001},prob(percent)="99",prob+-sd="99+-1"]:2.945572e-002[&length_mean=3.01008598e-002,length_median=2.94557200e-002,length_95%HPD={1.49992300e-002,4.49112400e-002}],(((((20[&prob=1.00000000e+000,prob_stddev=0.00000000e+000,prob_range={1.00000000e+000,1.00000000e+000},prob(percent)="100",prob+-sd="100+-0"]:7.304316e-002[&length_mean=7.41952029e-002,length_median=7.30431600e-002,length_95%HPD={4.09124900e-002,1.07776600e-001}],43[&prob=1.00000000e+000,prob_stddev=0.00000000e+000,prob_range={1.00000000e+000,1.00000000e+000},prob(percent)="100",prob+-sd="100+-0"]:1.310083e-001[&length_mean=1.32944108e-001,length_median=1.31008300e-001,length_95%HPD={9.04618900e-002,1.81817400e-001}])[&prob=9.97395833e-001,prob_stddev=3.55168839e-003,prob_range={9.92476852e-001,1.00000000e+000},prob(percent)="100",prob+-sd="100+-0"]:7.418307e-002[&length_mean=7.55646711e-002,length_median=7.41830700e-002,length_95%HPD={4.39481300e-002,1.10402500e-001}],45[&prob=1.00000000e+000,prob_stddev=0.00000000e+000,prob_range={1.00000000e+000,1.00000000e+000},prob(percent)="100",prob+-sd="100+-0"]:8.099761e-002[&length_mean=8.24144893e-002,length_median=8.09976100e-002,length_95%HPD={5.02690000e-002,1.16575900e-001}])[&prob=9.94646991e-001,prob_stddev=7.12495275e-003,prob_range={9.84953704e-001,1.00000000e+000},prob(percent)="99",prob+-sd="99+-1"]:6.493540e-002[&length_mean=6.61831995e-002,length_median=6.49354000e-002,length_95%HPD={3.49928000e-002,1.01203100e-001}],44[&prob=1.00000000e+000,prob_stddev=0.00000000e+000,prob_range={1.00000000e+000,1.00000000e+000},prob(percent)="100",prob+-sd="100+-0"]:2.116737e-001[&length_mean=2.14388491e-001,length_median=2.11673700e-001,length_95%HPD={1.47005100e-001,2.85327000e-001}])[&prob=9.93778935e-001,prob_stddev=8.01004811e-003,prob_range={9.83217593e-001,1.00000000e+000},prob(percent)="99",prob+-sd="99+-1"]:6.224777e-002[&length_mean=6.33606730e-002,length_median=6.22477700e-002,length_95%HPD={3.10264200e-002,9.64681000e-002}],46[&prob=1.00000000e+000,prob_stddev=0.00000000e+000,prob_range={1.00000000e+000,1.00000000e+000},prob(percent)="100",prob+-sd="100+-0"]:2.893786e-001[&length_mean=2.92444635e-001,length_median=2.89378600e-001,length_95%HPD={2.16913100e-001,3.74185600e-001}])[&prob=9.90162037e-001,prob_stddev=1.29143019e-002,prob_range={9.72800926e-001,1.00000000e+000},prob(percent)="99",prob+-sd="99+-1"]:1.199514e-001[&length_mean=1.21109756e-001,length_median=1.19951400e-001,length_95%HPD={7.88644000e-002,1.66578300e-001}],42[&prob=1.00000000e+000,prob_stddev=0.00000000e+000,prob_range={1.00000000e+000,1.00000000e+000},prob(percent)="100",prob+-sd="100+-0"]:2.957170e-001[&length_mean=2.98223360e-001,length_median=2.95717000e-001,length_95%HPD={2.37713200e-001,3.70496400e-001}])[&prob=9.88136574e-001,prob_stddev=1.61242818e-002,prob_range={9.65856481e-001,1.00000000e+000},prob(percent)="99",prob+-sd="99+-2"]:3.176697e-002[&length_mean=3.26546260e-002,length_median=3.17669700e-002,length_95%HPD={1.57254200e-002,5.12121500e-002}])[&prob=9.92476852e-001,prob_stddev=4.86478403e-003,prob_range={9.87268519e-001,9.97685185e-001},prob(percent)="99",prob+-sd="99+-0"]:2.976802e-001[&length_mean=3.00906475e-001,length_median=2.97680200e-001,length_95%HPD={2.30326500e-001,3.76401600e-001}],(((((22[&prob=1.00000000e+000,prob_stddev=0.00000000e+000,prob_range={1.00000000e+000,1.00000000e+000},prob(percent)="100",prob+-sd="100+-0"]:1.364004e-001[&length_mean=1.37953529e-001,length_median=1.36400400e-001,length_95%HPD={9.42263200e-002,1.87175600e-001}],(48[&prob=1.00000000e+000,prob_stddev=0.00000000e+000,prob_range={1.00000000e+000,1.00000000e+000},prob(percent)="100",prob+-sd="100+-0"]:1.604263e-001[&length_mean=1.61936856e-001,length_median=1.60426300e-001,length_95%HPD={1.07037600e-001,2.22693700e-001}],50[&prob=1.00000000e+000,prob_stddev=0.00000000e+000,prob_range={1.00000000e+000,1.00000000e+000},prob(percent)="100",prob+-sd="100+-0"]:1.171729e-001[&length_mean=1.19484337e-001,length_median=1.17172900e-001,length_95%HPD={7.12059900e-002,1.68625400e-001}])[&prob=9.98119213e-001,prob_stddev=1.72805487e-003,prob_range={9.95949074e-001,1.00000000e+000},prob(percent)="100",prob+-sd="100+-0"]:6.028098e-002[&length_mean=6.18908842e-002,length_median=6.02809800e-002,length_95%HPD={2.86588900e-002,9.70999800e-002}])[&prob=9.96238426e-001,prob_stddev=3.15203214e-003,prob_range={9.92476852e-001,1.00000000e+000},prob(percent)="100",prob+-sd="100+-0"]:4.905061e-002[&length_mean=5.03685692e-002,length_median=4.90506100e-002,length_95%HPD={2.13011000e-002,8.22621900e-002}],(47[&prob=1.00000000e+000,prob_stddev=0.00000000e+000,prob_range={1.00000000e+000,1.00000000e+000},prob(percent)="100",prob+-sd="100+-0"]:1.567991e-001[&length_mean=1.59054484e-001,length_median=1.56799100e-001,length_95%HPD={1.05196300e-001,2.21476800e-001}],52[&prob=1.00000000e+000,prob_stddev=0.00000000e+000,prob_range={1.00000000e+000,1.00000000e+000},prob(percent)="100",prob+-sd="100+-0"]:1.578388e-001[&length_mean=1.60617047e-001,length_median=1.57838800e-001,length_95%HPD={1.04898300e-001,2.25155700e-001}])[&prob=9.48350694e-001,prob_stddev=3.82046727e-003,prob_range={9.43287037e-001,9.52546296e-001},prob(percent)="95",prob+-sd="95+-0"]:2.736449e-002[&length_mean=2.88377137e-002,length_median=2.73644900e-002,length_95%HPD={6.09041300e-003,5.42215900e-002}])[&prob=9.90740741e-001,prob_stddev=9.76392804e-003,prob_range={9.76851852e-001,9.99421296e-001},prob(percent)="99",prob+-sd="99+-1"]:5.758663e-002[&length_mean=5.90707137e-002,length_median=5.75866300e-002,length_95%HPD={2.85109400e-002,9.25771300e-002}],49[&prob=1.00000000e+000,prob_stddev=0.00000000e+000,prob_range={1.00000000e+000,1.00000000e+000},prob(percent)="100",prob+-sd="100+-0"]:2.566450e-001[&length_mean=2.58474642e-001,length_median=2.56645000e-001,length_95%HPD={1.93817700e-001,3.21735200e-001}])[&prob=9.89438657e-001,prob_stddev=9.23964696e-003,prob_range={9.76851852e-001,9.98842593e-001},prob(percent)="99",prob+-sd="99+-1"]:9.352096e-002[&length_mean=9.45849850e-002,length_median=9.35209600e-002,length_95%HPD={6.46729200e-002,1.27800300e-001}],(51[&prob=1.00000000e+000,prob_stddev=0.00000000e+000,prob_range={1.00000000e+000,1.00000000e+000},prob(percent)="100",prob+-sd="100+-0"]:1.962169e-001[&length_mean=1.98892260e-001,length_median=1.96216900e-001,length_95%HPD={1.38608100e-001,2.66879400e-001}],53[&prob=1.00000000e+000,prob_stddev=0.00000000e+000,prob_range={1.00000000e+000,1.00000000e+000},prob(percent)="100",prob+-sd="100+-0"]:1.900848e-001[&length_mean=1.92002698e-001,length_median=1.90084800e-001,length_95%HPD={1.36487300e-001,2.51732800e-001}])[&prob=9.97685185e-001,prob_stddev=1.63682125e-003,prob_range={9.96527778e-001,1.00000000e+000},prob(percent)="100",prob+-sd="100+-0"]:1.112982e-001[&length_mean=1.12990986e-001,length_median=1.11298200e-001,length_95%HPD={7.19328100e-002,1.59843500e-001}])[&prob=9.84519676e-001,prob_stddev=8.23001311e-003,prob_range={9.73379630e-001,9.92476852e-001},prob(percent)="98",prob+-sd="98+-1"]:1.160759e-001[&length_mean=1.17247906e-001,length_median=1.16075900e-001,length_95%HPD={8.14985200e-002,1.56228100e-001}],(((23[&prob=1.00000000e+000,prob_stddev=0.00000000e+000,prob_range={1.00000000e+000,1.00000000e+000},prob(percent)="100",prob+-sd="100+-0"]:3.167026e-001[&length_mean=3.18769537e-001,length_median=3.16702600e-001,length_95%HPD={2.46610300e-001,4.00902500e-001}],((24[&prob=1.00000000e+000,prob_stddev=0.00000000e+000,prob_range={1.00000000e+000,1.00000000e+000},prob(percent)="100",prob+-sd="100+-0"]:8.466432e-001[&length_mean=8.60071387e-001,length_median=8.46643200e-001,length_95%HPD={5.00016000e-001,1.23302600e+000}],(25[&prob=1.00000000e+000,prob_stddev=0.00000000e+000,prob_range={1.00000000e+000,1.00000000e+000},prob(percent)="100",prob+-sd="100+-0"]:1.728770e-001[&length_mean=1.73524010e-001,length_median=1.72877000e-001,length_95%HPD={1.23943100e-001,2.26434300e-001}],27[&prob=1.00000000e+000,prob_stddev=0.00000000e+000,prob_range={1.00000000e+000,1.00000000e+000},prob(percent)="100",prob+-sd="100+-0"]:2.010754e-001[&length_mean=2.02295960e-001,length_median=2.01075400e-001,length_95%HPD={1.54201900e-001,2.56224700e-001}])[&prob=7.82262731e-001,prob_stddev=1.90262136e-002,prob_range={7.64467593e-001,8.08449074e-001},prob(percent)="78",prob+-sd="78+-2"]:6.684266e-002[&length_mean=6.83745811e-002,length_median=6.68426600e-002,length_95%HPD={9.09093500e-004,1.30129500e-001}])[&prob=8.30439815e-001,prob_stddev=2.02187734e-002,prob_range={8.07870370e-001,8.57060185e-001},prob(percent)="83",prob+-sd="83+-2"]:7.241995e-002[&length_mean=7.24466570e-002,length_median=7.24199500e-002,length_95%HPD={1.08451900e-003,1.31299700e-001}],26[&prob=1.00000000e+000,prob_stddev=0.00000000e+000,prob_range={1.00000000e+000,1.00000000e+000},prob(percent)="100",prob+-sd="100+-0"]:2.629354e-001[&length_mean=2.65165866e-001,length_median=2.62935400e-001,length_95%HPD={2.08234100e-001,3.30032000e-001}])[&prob=9.76851852e-001,prob_stddev=1.11516269e-002,prob_range={9.65277778e-001,9.90162037e-001},prob(percent)="98",prob+-sd="98+-1"]:9.556692e-002[&length_mean=9.55268177e-002,length_median=9.55669200e-002,length_95%HPD={5.81517900e-002,1.34831600e-001}])[&prob=5.10995370e-001,prob_stddev=8.35954810e-003,prob_range={5.03472222e-001,5.18518519e-001},prob(percent)="51",prob+-sd="51+-1"]:2.822418e-002[&length_mean=2.87876588e-002,length_median=2.82241800e-002,length_95%HPD={1.23623300e-002,4.84469900e-002}],28[&prob=1.00000000e+000,prob_stddev=0.00000000e+000,prob_range={1.00000000e+000,1.00000000e+000},prob(percent)="100",prob+-sd="100+-0"]:3.803021e-001[&length_mean=3.83701351e-001,length_median=3.80302100e-001,length_95%HPD={3.03900800e-001,4.75431200e-001}])[&prob=9.80324074e-001,prob_stddev=1.08059133e-002,prob_range={9.71064815e-001,9.95949074e-001},prob(percent)="98",prob+-sd="98+-1"]:1.238219e-001[&length_mean=1.24785393e-001,length_median=1.23821900e-001,length_95%HPD={8.81860300e-002,1.64821900e-001}],(29[&prob=1.00000000e+000,prob_stddev=0.00000000e+000,prob_range={1.00000000e+000,1.00000000e+000},prob(percent)="100",prob+-sd="100+-0"]:2.607458e-001[&length_mean=2.62663018e-001,length_median=2.60745800e-001,length_95%HPD={2.07144900e-001,3.27256200e-001}],54[&prob=1.00000000e+000,prob_stddev=0.00000000e+000,prob_range={1.00000000e+000,1.00000000e+000},prob(percent)="100",prob+-sd="100+-0"]:2.154715e-001[&length_mean=2.16901354e-001,length_median=2.15471500e-001,length_95%HPD={1.62681700e-001,2.73558600e-001}])[&prob=9.93778935e-001,prob_stddev=6.65509259e-003,prob_range={9.83796296e-001,9.97106481e-001},prob(percent)="99",prob+-sd="99+-1"]:1.787772e-001[&length_mean=1.80756191e-001,length_median=1.78777200e-001,length_95%HPD={1.33432400e-001,2.30899300e-001}])[&prob=9.71643519e-001,prob_stddev=2.00468843e-003,prob_range={9.68750000e-001,9.73379630e-001},prob(percent)="97",prob+-sd="97+-0"]:4.660042e-002[&length_mean=4.75757526e-002,length_median=4.66004200e-002,length_95%HPD={2.49486900e-002,7.13771500e-002}])[&prob=9.55005787e-001,prob_stddev=9.25172099e-003,prob_range={9.43865741e-001,9.64699074e-001},prob(percent)="96",prob+-sd="96+-1"]:2.375988e-001[&length_mean=2.39077596e-001,length_median=2.37598800e-001,length_95%HPD={1.79299700e-001,3.01501000e-001}])[&prob=9.36197917e-001,prob_stddev=1.89968544e-002,prob_range={9.18981481e-001,9.57175926e-001},prob(percent)="94",prob+-sd="94+-2"]:2.466530e-001[&length_mean=2.47912156e-001,length_median=2.46653000e-001,length_95%HPD={1.81654200e-001,3.20745200e-001}],(((30[&prob=1.00000000e+000,prob_stddev=0.00000000e+000,prob_range={1.00000000e+000,1.00000000e+000},prob(percent)="100",prob+-sd="100+-0"]:3.427389e-001[&length_mean=3.40422279e-001,length_median=3.42738900e-001,length_95%HPD={2.58723500e-001,4.62936800e-001}],68[&prob=1.00000000e+000,prob_stddev=0.00000000e+000,prob_range={1.00000000e+000,1.00000000e+000},prob(percent)="100",prob+-sd="100+-0"]:4.538443e-001[&length_mean=4.55054160e-001,length_median=4.53844300e-001,length_95%HPD={3.27528400e-001,5.98522300e-001}])[&prob=9.11892361e-001,prob_stddev=4.23268784e-002,prob_range={8.55324074e-001,9.55439815e-001},prob(percent)="91",prob+-sd="91+-4"]:8.181175e-002[&length_mean=8.27699807e-002,length_median=8.18117500e-002,length_95%HPD={4.49730100e-002,1.25081900e-001}],(((31[&prob=1.00000000e+000,prob_stddev=0.00000000e+000,prob_range={1.00000000e+000,1.00000000e+000},prob(percent)="100",prob+-sd="100+-0"]:2.479393e-001[&length_mean=2.49780238e-001,length_median=2.47939300e-001,length_95%HPD={1.78242900e-001,3.28897500e-001}],56[&prob=1.00000000e+000,prob_stddev=0.00000000e+000,prob_range={1.00000000e+000,1.00000000e+000},prob(percent)="100",prob+-sd="100+-0"]:3.414752e-001[&length_mean=3.42713570e-001,length_median=3.41475200e-001,length_95%HPD={2.39689500e-001,4.50899600e-001}])[&prob=9.69473380e-001,prob_stddev=7.91189064e-003,prob_range={9.57754630e-001,9.74537037e-001},prob(percent)="97",prob+-sd="97+-1"]:8.187014e-002[&length_mean=8.33590480e-002,length_median=8.18701400e-002,length_95%HPD={4.41784800e-002,1.26016000e-001}],55[&prob=1.00000000e+000,prob_stddev=0.00000000e+000,prob_range={1.00000000e+000,1.00000000e+000},prob(percent)="100",prob+-sd="100+-0"]:3.393383e-001[&length_mean=3.42417819e-001,length_median=3.39338300e-001,length_95%HPD={2.52096800e-001,4.50844200e-001}])[&prob=9.37065972e-001,prob_stddev=2.15568748e-002,prob_range={9.09143519e-001,9.59490741e-001},prob(percent)="94",prob+-sd="94+-2"]:1.737029e-001[&length_mean=1.74166872e-001,length_median=1.73702900e-001,length_95%HPD={1.16983200e-001,2.35873400e-001}],(32[&prob=1.00000000e+000,prob_stddev=0.00000000e+000,prob_range={1.00000000e+000,1.00000000e+000},prob(percent)="100",prob+-sd="100+-0"]:3.900473e-001[&length_mean=3.91207499e-001,length_median=3.90047300e-001,length_95%HPD={3.02237700e-001,4.88581700e-001}],(59[&prob=1.00000000e+000,prob_stddev=0.00000000e+000,prob_range={1.00000000e+000,1.00000000e+000},prob(percent)="100",prob+-sd="100+-0"]:2.613824e-001[&length_mean=2.64290197e-001,length_median=2.61382400e-001,length_95%HPD={1.78339800e-001,3.58934500e-001}],((62[&prob=1.00000000e+000,prob_stddev=0.00000000e+000,prob_range={1.00000000e+000,1.00000000e+000},prob(percent)="100",prob+-sd="100+-0"]:2.030083e-001[&length_mean=2.05074937e-001,length_median=2.03008300e-001,length_95%HPD={1.42579700e-001,2.70582000e-001}],((70[&prob=1.00000000e+000,prob_stddev=0.00000000e+000,prob_range={1.00000000e+000,1.00000000e+000},prob(percent)="100",prob+-sd="100+-0"]:2.137297e-002[&length_mean=2.45696831e-002,length_median=2.13729700e-002,length_95%HPD={1.13093600e-007,5.61566600e-002}],76[&prob=1.00000000e+000,prob_stddev=0.00000000e+000,prob_range={1.00000000e+000,1.00000000e+000},prob(percent)="100",prob+-sd="100+-0"]:1.492319e-001[&length_mean=1.57696890e-001,length_median=1.49231900e-001,length_95%HPD={4.68312900e-002,2.75173200e-001}])[&prob=9.58333333e-001,prob_stddev=6.66556811e-003,prob_range={9.50810185e-001,9.65277778e-001},prob(percent)="96",prob+-sd="96+-1"]:4.898999e-002[&length_mean=5.15029541e-002,length_median=4.89899900e-002,length_95%HPD={6.38959700e-003,9.84694500e-002}],75[&prob=1.00000000e+000,prob_stddev=0.00000000e+000,prob_range={1.00000000e+000,1.00000000e+000},prob(percent)="100",prob+-sd="100+-0"]:1.991864e-001[&length_mean=2.07840843e-001,length_median=1.99186400e-001,length_95%HPD={7.74322600e-002,3.59188000e-001}])[&prob=9.73234954e-001,prob_stddev=1.84483080e-002,prob_range={9.46759259e-001,9.86689815e-001},prob(percent)="97",prob+-sd="97+-2"]:6.694163e-002[&length_mean=6.86090006e-002,length_median=6.69416300e-002,length_95%HPD={1.68790200e-002,1.20618200e-001}])[&prob=8.99160880e-001,prob_stddev=1.71760650e-002,prob_range={8.76157407e-001,9.14930556e-001},prob(percent)="90",prob+-sd="90+-2"]:2.907483e-002[&length_mean=3.04987334e-002,length_median=2.90748300e-002,length_95%HPD={9.01490700e-003,5.40405800e-002}],73[&prob=1.00000000e+000,prob_stddev=0.00000000e+000,prob_range={1.00000000e+000,1.00000000e+000},prob(percent)="100",prob+-sd="100+-0"]:2.331376e-001[&length_mean=2.35773314e-001,length_median=2.33137600e-001,length_95%HPD={1.60289900e-001,3.18721800e-001}])[&prob=8.37384259e-001,prob_stddev=1.94189372e-002,prob_range={8.15393519e-001,8.61689815e-001},prob(percent)="84",prob+-sd="84+-2"]:2.382814e-002[&length_mean=2.55934312e-002,length_median=2.38281400e-002,length_95%HPD={2.02349700e-003,5.09414800e-002}])[&prob=9.32725694e-001,prob_stddev=2.28929558e-002,prob_range={9.08564815e-001,9.56597222e-001},prob(percent)="93",prob+-sd="93+-2"]:1.589225e-001[&length_mean=1.60770790e-001,length_median=1.58922500e-001,length_95%HPD={1.04663700e-001,2.18018700e-001}])[&prob=9.03645833e-001,prob_stddev=2.78038875e-002,prob_range={8.80787037e-001,9.42129630e-001},prob(percent)="90",prob+-sd="90+-3"]:1.630228e-001[&length_mean=1.63190568e-001,length_median=1.63022800e-001,length_95%HPD={1.16507900e-001,2.14292700e-001}])[&prob=6.12123843e-001,prob_stddev=2.50758621e-002,prob_range={5.81597222e-001,6.35416667e-001},prob(percent)="61",prob+-sd="61+-3"]:1.643605e-002[&length_mean=1.71525011e-002,length_median=1.64360500e-002,length_95%HPD={5.06306700e-003,3.04359800e-002}],(((57[&prob=1.00000000e+000,prob_stddev=0.00000000e+000,prob_range={1.00000000e+000,1.00000000e+000},prob(percent)="100",prob+-sd="100+-0"]:1.241764e-001[&length_mean=1.26432438e-001,length_median=1.24176400e-001,length_95%HPD={7.74642200e-002,1.80329000e-001}],65[&prob=1.00000000e+000,prob_stddev=0.00000000e+000,prob_range={1.00000000e+000,1.00000000e+000},prob(percent)="100",prob+-sd="100+-0"]:1.214163e-001[&length_mean=1.23345383e-001,length_median=1.21416300e-001,length_95%HPD={7.67070700e-002,1.75128000e-001}])[&prob=9.84809028e-001,prob_stddev=8.06560177e-003,prob_range={9.72800926e-001,9.90162037e-001},prob(percent)="98",prob+-sd="98+-1"]:1.992303e-001[&length_mean=2.00841401e-001,length_median=1.99230300e-001,length_95%HPD={1.31342200e-001,2.82005600e-001}],(((((58[&prob=1.00000000e+000,prob_stddev=0.00000000e+000,prob_range={1.00000000e+000,1.00000000e+000},prob(percent)="100",prob+-sd="100+-0"]:2.456012e-001[&length_mean=2.46534428e-001,length_median=2.45601200e-001,length_95%HPD={1.67403400e-001,3.38025400e-001}],60[&prob=1.00000000e+000,prob_stddev=0.00000000e+000,prob_range={1.00000000e+000,1.00000000e+000},prob(percent)="100",prob+-sd="100+-0"]:3.039398e-001[&length_mean=3.05194185e-001,length_median=3.03939800e-001,length_95%HPD={2.15202600e-001,4.06971700e-001}])[&prob=7.20630787e-001,prob_stddev=1.69733905e-002,prob_range={6.96759259e-001,7.34375000e-001},prob(percent)="72",prob+-sd="72+-2"]:3.187594e-002[&length_mean=3.39166160e-002,length_median=3.18759400e-002,length_95%HPD={2.93433800e-003,6.80202300e-002}],61[&prob=1.00000000e+000,prob_stddev=0.00000000e+000,prob_range={1.00000000e+000,1.00000000e+000},prob(percent)="100",prob+-sd="100+-0"]:2.063995e-001[&length_mean=2.07333058e-001,length_median=2.06399500e-001,length_95%HPD={1.48012200e-001,2.75216300e-001}])[&prob=7.21643519e-001,prob_stddev=1.91235088e-002,prob_range={6.94444444e-001,7.36111111e-001},prob(percent)="72",prob+-sd="72+-2"]:1.889408e-002[&length_mean=2.03811598e-002,length_median=1.88940800e-002,length_95%HPD={3.72838100e-003,4.07749900e-002}],67[&prob=1.00000000e+000,prob_stddev=0.00000000e+000,prob_range={1.00000000e+000,1.00000000e+000},prob(percent)="100",prob+-sd="100+-0"]:2.606003e-001[&length_mean=2.62173753e-001,length_median=2.60600300e-001,length_95%HPD={1.81720900e-001,3.52499200e-001}])[&prob=7.95138889e-001,prob_stddev=1.24746113e-002,prob_range={7.76620370e-001,8.03819444e-001},prob(percent)="80",prob+-sd="80+-1"]:2.479507e-002[&length_mean=2.61378373e-002,length_median=2.47950700e-002,length_95%HPD={5.55092700e-003,4.88340200e-002}],(63[&prob=1.00000000e+000,prob_stddev=0.00000000e+000,prob_range={1.00000000e+000,1.00000000e+000},prob(percent)="100",prob+-sd="100+-0"]:1.042182e-001[&length_mean=1.05608398e-001,length_median=1.04218200e-001,length_95%HPD={6.23215800e-002,1.57062100e-001}],69[&prob=1.00000000e+000,prob_stddev=0.00000000e+000,prob_range={1.00000000e+000,1.00000000e+000},prob(percent)="100",prob+-sd="100+-0"]:2.191427e-001[&length_mean=2.17486307e-001,length_median=2.19142700e-001,length_95%HPD={1.40750700e-001,3.01571300e-001}])[&prob=9.40104167e-001,prob_stddev=1.67357803e-002,prob_range={9.20717593e-001,9.59490741e-001},prob(percent)="94",prob+-sd="94+-2"]:3.646851e-002[&length_mean=3.78557404e-002,length_median=3.64685100e-002,length_95%HPD={1.29977800e-002,6.46532600e-002}])[&prob=8.27690972e-001,prob_stddev=1.40516997e-002,prob_range={8.10185185e-001,8.42592593e-001},prob(percent)="83",prob+-sd="83+-1"]:6.663056e-002[&length_mean=6.78904111e-002,length_median=6.66305600e-002,length_95%HPD={3.26845400e-002,1.06225500e-001}],72[&prob=1.00000000e+000,prob_stddev=0.00000000e+000,prob_range={1.00000000e+000,1.00000000e+000},prob(percent)="100",prob+-sd="100+-0"]:3.423886e-001[&length_mean=3.43733004e-001,length_median=3.42388600e-001,length_95%HPD={2.35549200e-001,4.63340800e-001}])[&prob=7.75462963e-001,prob_stddev=1.35718049e-002,prob_range={7.62152778e-001,7.89351852e-001},prob(percent)="78",prob+-sd="78+-1"]:9.388382e-002[&length_mean=9.47470478e-002,length_median=9.38838200e-002,length_95%HPD={5.34212600e-002,1.42853600e-001}])[&prob=5.85792824e-001,prob_stddev=2.34993373e-002,prob_range={5.63078704e-001,6.18055556e-001},prob(percent)="59",prob+-sd="59+-2"]:1.612164e-002[&length_mean=1.75366687e-002,length_median=1.61216400e-002,length_95%HPD={2.21543700e-003,3.65628500e-002}],(64[&prob=1.00000000e+000,prob_stddev=0.00000000e+000,prob_range={1.00000000e+000,1.00000000e+000},prob(percent)="100",prob+-sd="100+-0"]:4.073865e-001[&length_mean=4.07934900e-001,length_median=4.07386500e-001,length_95%HPD={2.85254700e-001,5.53123600e-001}],((66[&prob=1.00000000e+000,prob_stddev=0.00000000e+000,prob_range={1.00000000e+000,1.00000000e+000},prob(percent)="100",prob+-sd="100+-0"]:3.140098e-001[&length_mean=3.15628997e-001,length_median=3.14009800e-001,length_95%HPD={2.09091700e-001,4.33997900e-001}],74[&prob=1.00000000e+000,prob_stddev=0.00000000e+000,prob_range={1.00000000e+000,1.00000000e+000},prob(percent)="100",prob+-sd="100+-0"]:3.707288e-001[&length_mean=3.72079894e-001,length_median=3.70728800e-001,length_95%HPD={2.56387200e-001,5.03821100e-001}])[&prob=8.34490741e-001,prob_stddev=2.47470557e-002,prob_range={8.02083333e-001,8.57060185e-001},prob(percent)="83",prob+-sd="83+-2"]:4.162715e-002[&length_mean=4.39780263e-002,length_median=4.16271500e-002,length_95%HPD={6.82458700e-003,8.18025500e-002}],71[&prob=1.00000000e+000,prob_stddev=0.00000000e+000,prob_range={1.00000000e+000,1.00000000e+000},prob(percent)="100",prob+-sd="100+-0"]:3.012371e-001[&length_mean=3.01050142e-001,length_median=3.01237100e-001,length_95%HPD={2.24035300e-001,4.05118000e-001}])[&prob=8.81799769e-001,prob_stddev=2.33802742e-002,prob_range={8.50694444e-001,9.07407407e-001},prob(percent)="88",prob+-sd="88+-2"]:5.775351e-002[&length_mean=5.96030743e-002,length_median=5.77535100e-002,length_95%HPD={2.29069600e-002,9.76451600e-002}])[&prob=8.19155093e-001,prob_stddev=2.79720093e-002,prob_range={7.88194444e-001,8.55324074e-001},prob(percent)="82",prob+-sd="82+-3"]:3.471774e-002[&length_mean=3.62749566e-002,length_median=3.47177400e-002,length_95%HPD={1.05413700e-002,6.62925900e-002}])[&prob=5.27199074e-001,prob_stddev=5.11337475e-002,prob_range={4.51967593e-001,5.62500000e-001},prob(percent)="53",prob+-sd="53+-5"]:6.720696e-002[&length_mean=6.79599111e-002,length_median=6.72069600e-002,length_95%HPD={4.03254400e-002,1.02261200e-001}],88[&prob=1.00000000e+000,prob_stddev=0.00000000e+000,prob_range={1.00000000e+000,1.00000000e+000},prob(percent)="100",prob+-sd="100+-0"]:8.360814e-002[&length_mean=1.24893387e-001,length_median=8.36081400e-002,length_95%HPD={9.38509700e-006,3.85879900e-001}])[&prob=7.98900463e-001,prob_stddev=3.67176745e-002,prob_range={7.66203704e-001,8.46643519e-001},prob(percent)="80",prob+-sd="80+-4"]:1.679313e-001[&length_mean=1.67890130e-001,length_median=1.67931300e-001,length_95%HPD={1.16044000e-001,2.28005900e-001}],((33[&prob=1.00000000e+000,prob_stddev=0.00000000e+000,prob_range={1.00000000e+000,1.00000000e+000},prob(percent)="100",prob+-sd="100+-0"]:8.536092e-002[&length_mean=8.69142911e-002,length_median=8.53609200e-002,length_95%HPD={4.38177100e-002,1.33416900e-001}],(80[&prob=1.00000000e+000,prob_stddev=0.00000000e+000,prob_range={1.00000000e+000,1.00000000e+000},prob(percent)="100",prob+-sd="100+-0"]:2.150406e-001[&length_mean=2.20732182e-001,length_median=2.15040600e-001,length_95%HPD={1.02746000e-001,3.47098500e-001}],81[&prob=1.00000000e+000,prob_stddev=0.00000000e+000,prob_range={1.00000000e+000,1.00000000e+000},prob(percent)="100",prob+-sd="100+-0"]:1.428338e-001[&length_mean=1.46822057e-001,length_median=1.42833800e-001,length_95%HPD={7.66656200e-002,2.19615400e-001}])[&prob=8.82378472e-001,prob_stddev=6.07526353e-002,prob_range={7.98032407e-001,9.40972222e-001},prob(percent)="88",prob+-sd="88+-6"]:3.768136e-002[&length_mean=4.03375238e-002,length_median=3.76813600e-002,length_95%HPD={9.40075300e-003,7.86190600e-002}])[&prob=8.83680556e-001,prob_stddev=5.94405052e-002,prob_range={8.01504630e-001,9.40972222e-001},prob(percent)="88",prob+-sd="88+-6"]:1.709425e-001[&length_mean=1.72173624e-001,length_median=1.70942500e-001,length_95%HPD={1.15967700e-001,2.33015800e-001}],(77[&prob=1.00000000e+000,prob_stddev=0.00000000e+000,prob_range={1.00000000e+000,1.00000000e+000},prob(percent)="100",prob+-sd="100+-0"]:2.031530e-001[&length_mean=2.05455929e-001,length_median=2.03153000e-001,length_95%HPD={1.49183800e-001,2.69035300e-001}],(78[&prob=1.00000000e+000,prob_stddev=0.00000000e+000,prob_range={1.00000000e+000,1.00000000e+000},prob(percent)="100",prob+-sd="100+-0"]:1.327613e-001[&length_mean=1.37880418e-001,length_median=1.32761300e-001,length_95%HPD={5.26755000e-002,2.38439300e-001}],79[&prob=1.00000000e+000,prob_stddev=0.00000000e+000,prob_range={1.00000000e+000,1.00000000e+000},prob(percent)="100",prob+-sd="100+-0"]:6.129468e-002[&length_mean=6.89629825e-002,length_median=6.12946800e-002,length_95%HPD={6.26518600e-003,1.46152300e-001}])[&prob=8.75723380e-001,prob_stddev=5.79482019e-002,prob_range={8.01504630e-001,9.40972222e-001},prob(percent)="88",prob+-sd="88+-6"]:8.486983e-002[&length_mean=8.76131986e-002,length_median=8.48698300e-002,length_95%HPD={1.74749300e-002,1.62179700e-001}])[&prob=6.05324074e-001,prob_stddev=4.39256062e-002,prob_range={5.52662037e-001,6.57986111e-001},prob(percent)="61",prob+-sd="61+-4"]:2.403657e-002[&length_mean=2.57847898e-002,length_median=2.40365700e-002,length_95%HPD={6.78032500e-004,5.16738600e-002}])[&prob=9.73813657e-001,prob_stddev=6.29298703e-003,prob_range={9.69907407e-001,9.83217593e-001},prob(percent)="97",prob+-sd="97+-1"]:4.305186e-001[&length_mean=4.29560548e-001,length_median=4.30518600e-001,length_95%HPD={3.28735900e-001,5.50386400e-001}])[&prob=8.73987269e-001,prob_stddev=3.26044461e-002,prob_range={8.32175926e-001,9.05671296e-001},prob(percent)="87",prob+-sd="87+-3"]:1.713404e-001[&length_mean=1.71746457e-001,length_median=1.71340400e-001,length_95%HPD={1.16875100e-001,2.32402200e-001}])[&prob=9.15943287e-001,prob_stddev=3.97115432e-002,prob_range={8.57638889e-001,9.43287037e-001},prob(percent)="92",prob+-sd="92+-4"]:3.975811e-001[&length_mean=3.71800289e-001,length_median=3.97581100e-001,length_95%HPD={1.16825700e-001,5.70866100e-001}],86[&prob=1.00000000e+000,prob_stddev=0.00000000e+000,prob_range={1.00000000e+000,1.00000000e+000},prob(percent)="100",prob+-sd="100+-0"]:1.969703e-001[&length_mean=2.38912128e-001,length_median=1.96970300e-001,length_95%HPD={4.34804900e-004,5.75653000e-001}])[&prob=7.27575231e-001,prob_stddev=7.45583235e-002,prob_range={6.47569444e-001,8.15393519e-001},prob(percent)="73",prob+-sd="73+-7"]:2.046121e-001[&length_mean=2.01516542e-001,length_median=2.04612100e-001,length_95%HPD={2.83295100e-002,3.39763700e-001}])[&prob=9.62528935e-001,prob_stddev=4.06368554e-002,prob_range={9.06828704e-001,9.93055556e-001},prob(percent)="96",prob+-sd="96+-4"]:1.033537e+000[&length_mean=1.01771350e+000,length_median=1.03353700e+000,length_95%HPD={5.67603300e-001,1.41323100e+000}])[&prob=9.14351852e-001,prob_stddev=5.16485626e-002,prob_range={8.43750000e-001,9.58333333e-001},prob(percent)="91",prob+-sd="91+-5"]:2.018632e-001[&length_mean=2.03527342e-001,length_median=2.01863200e-001,length_95%HPD={1.19447500e-001,2.96513200e-001}])[&prob=7.56076389e-001,prob_stddev=3.27040136e-002,prob_range={7.07754630e-001,7.77777778e-001},prob(percent)="76",prob+-sd="76+-3"]:6.653975e-001[&length_mean=7.49023003e-001,length_median=6.65397500e-001,length_95%HPD={6.49707600e-002,1.62264200e+000}],(82[&prob=1.00000000e+000,prob_stddev=0.00000000e+000,prob_range={1.00000000e+000,1.00000000e+000},prob(percent)="100",prob+-sd="100+-0"]:7.161903e-002[&length_mean=1.09032735e-001,length_median=7.16190300e-002,length_95%HPD={2.10206300e-005,3.35468700e-001}],83[&prob=1.00000000e+000,prob_stddev=0.00000000e+000,prob_range={1.00000000e+000,1.00000000e+000},prob(percent)="100",prob+-sd="100+-0"]:7.337965e-002[&length_mean=1.09114363e-001,length_median=7.33796500e-002,length_95%HPD={3.45819600e-005,3.33520100e-001}],84[&prob=1.00000000e+000,prob_stddev=0.00000000e+000,prob_range={1.00000000e+000,1.00000000e+000},prob(percent)="100",prob+-sd="100+-0"]:8.480846e-002[&length_mean=1.25484673e-001,length_median=8.48084600e-002,length_95%HPD={3.60155600e-005,3.93478900e-001}])[&prob=7.16001157e-001,prob_stddev=8.89492254e-003,prob_range={7.05439815e-001,7.26273148e-001},prob(percent)="72",prob+-sd="72+-1"]:3.203338e-001[&length_mean=3.75290747e-001,length_median=3.20333800e-001,length_95%HPD={2.33260700e-003,8.90478200e-001}])[&prob=8.73119213e-001,prob_stddev=3.33692653e-002,prob_range={8.48958333e-001,9.22453704e-001},prob(percent)="87",prob+-sd="87+-3"]:9.831910e-001[&length_mean=1.00202152e+000,length_median=9.83191000e-001,length_95%HPD={2.18734300e-001,1.73692800e+000}])[&prob=8.89612269e-001,prob_stddev=3.21666785e-002,prob_range={8.61689815e-001,9.35763889e-001},prob(percent)="89",prob+-sd="89+-3"]:3.487760e-001[&length_mean=3.51337163e-001,length_median=3.48776000e-001,length_95%HPD={2.34012200e-001,4.80688400e-001}],6[&prob=1.00000000e+000,prob_stddev=0.00000000e+000,prob_range={1.00000000e+000,1.00000000e+000},prob(percent)="100",prob+-sd="100+-0"]:8.980597e-001[&length_mean=8.93588961e-001,length_median=8.98059700e-001,length_95%HPD={6.56637900e-001,1.16986300e+000}])[&prob=9.66001157e-001,prob_stddev=1.48549607e-002,prob_range={9.50810185e-001,9.84375000e-001},prob(percent)="97",prob+-sd="97+-1"]:6.947925e-001[&length_mean=6.97717945e-001,length_median=6.94792500e-001,length_95%HPD={2.43797300e-001,1.20217400e+000}],5[&prob=1.00000000e+000,prob_stddev=0.00000000e+000,prob_range={1.00000000e+000,1.00000000e+000},prob(percent)="100",prob+-sd="100+-0"]:5.637049e-001[&length_mean=5.80839842e-001,length_median=5.63704900e-001,length_95%HPD={1.62042100e-001,1.03114000e+000}])[&prob=9.91464120e-001,prob_stddev=8.91998754e-003,prob_range={9.80902778e-001,9.99421296e-001},prob(percent)="99",prob+-sd="99+-1"]:6.132395e-001[&length_mean=6.43200027e-001,length_median=6.13239500e-001,length_95%HPD={1.89930300e-001,1.15722700e+000}])[&prob=7.84143519e-001,prob_stddev=4.35632322e-003,prob_range={7.78935185e-001,7.88773148e-001},prob(percent)="78",prob+-sd="78+-0"]:2.188122e-001[&length_mean=2.48461212e-001,length_median=2.18812200e-001,length_95%HPD={5.05710200e-004,5.68969800e-001}]);

end;

**NodeCalib1 (Maximum Clade Credibility tree)**

***
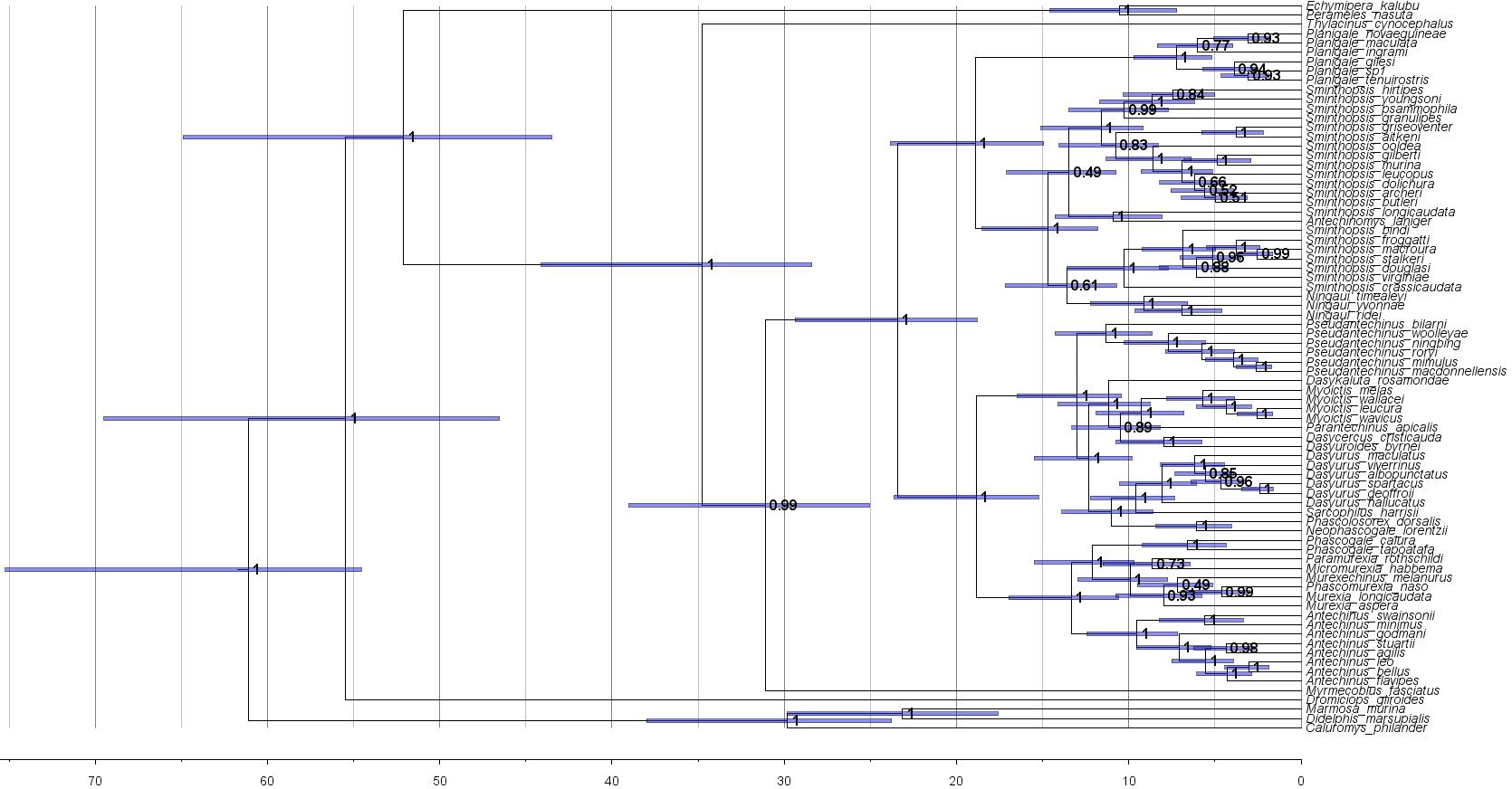
***

#NEXUS

Begin taxa;

Dimensions ntax=78;

Taxlabels

Caluromys_philander

Didelphis_marsupialis

Marmosa_murina

Dromiciops_gliroides

Perameles_nasuta

Echymipera_kalubu

Thylacinus_cynocephalus

Myrmecobius_fasciatus

Dasykaluta_rosamondae

Dasycercus_cristicauda

Dasyuroides_byrnei

Dasyurus_hallucatus

Myoictis_wavicus

Neophascogale_lorentzii

Phascolosorex_dorsalis

Parantechinus_apicalis

Pseudantechinus_macdonnellensis

Sarcophilus_harrisii

Antechinus_flavipes

Micromurexia_habbema

Murexia_aspera

Murexia_longicaudata

Murexechinus_melanurus

Phascomurexia_naso

Paramurexia_rothschildi

Phascogale_tapoatafa

Antechinomys_laniger

Ningaui_ridei

Sminthopsis_crassicaudata

Planigale_gilesi

Dasyurus_albopunctatus

Dasyurus_geoffroii

Dasyurus_maculatus

Dasyurus_spartacus

Dasyurus_viverrinus

Myoictis_leucura

Myoictis_melas

Myoictis_wallacei

Pseudantechinus_bilarni

Pseudantechinus_mimulus

Pseudantechinus_ningbing

Pseudantechinus_roryi

Pseudantechinus_woolleyae

Antechinus_agilis

Antechinus_bellus

Antechinus_godmani

Antechinus_leo

Antechinus_minimus

Antechinus_stuartii

Antechinus_swainsonii

Phascogale_calura

Ningaui_timealeyi

Ningaui_yvonnae

Sminthopsis_aitkeni

Sminthopsis_archeri

Sminthopsis_bindi

Sminthopsis_butleri

Sminthopsis_dolichura

Sminthopsis_douglasi

Sminthopsis_gilberti

Sminthopsis_granulipes

Sminthopsis_griseoventer

Sminthopsis_hirtipes

Sminthopsis_leucopus

Sminthopsis_longicaudata

Sminthopsis_murina

Sminthopsis_macroura

Sminthopsis_psammophila

Sminthopsis_ooldea

Sminthopsis_virginiae

Sminthopsis_youngsoni

Sminthopsis_froggatti

Sminthopsis_stalkeri

Planigale_ingrami

Planigale_maculata

Planigale_novaeguineae

Planigale_tenuirostris

Planigale_sp1

;

End;

Begin trees;

Translate

1 Caluromys_philander,

2 Didelphis_marsupialis,

3 Marmosa_murina,

4 Dromiciops_gliroides,

5 Perameles_nasuta,

6 Echymipera_kalubu,

7 Thylacinus_cynocephalus,

8 Myrmecobius_fasciatus,

9 Dasykaluta_rosamondae,

10 Dasycercus_cristicauda,

11 Dasyuroides_byrnei,

12 Dasyurus_hallucatus,

13 Myoictis_wavicus,

14 Neophascogale_lorentzii,

15 Phascolosorex_dorsalis,

16 Parantechinus_apicalis,

17 Pseudantechinus_macdonnellensis,

18 Sarcophilus_harrisii,

19 Antechinus_flavipes,

20 Micromurexia_habbema,

21 Murexia_aspera,

22 Murexia_longicaudata,

23 Murexechinus_melanurus,

24 Phascomurexia_naso,

25 Paramurexia_rothschildi,

26 Phascogale_tapoatafa,

27 Antechinomys_laniger,

28 Ningaui_ridei,

29 Sminthopsis_crassicaudata,

30 Planigale_gilesi,

31 Dasyurus_albopunctatus,

32 Dasyurus_geoffroii,

33 Dasyurus_maculatus,

34 Dasyurus_spartacus,

35 Dasyurus_viverrinus,

36 Myoictis_leucura,

37 Myoictis_melas,

38 Myoictis_wallacei,

39 Pseudantechinus_bilarni,

40 Pseudantechinus_mimulus,

41 Pseudantechinus_ningbing,

42 Pseudantechinus_roryi,

43 Pseudantechinus_woolleyae,

44 Antechinus_agilis,

45 Antechinus_bellus,

46 Antechinus_godmani,

47 Antechinus_leo,

48 Antechinus_minimus,

49 Antechinus_stuartii,

50 Antechinus_swainsonii,

51 Phascogale_calura,

52 Ningaui_timealeyi,

53 Ningaui_yvonnae,

54 Sminthopsis_aitkeni,

55 Sminthopsis_archeri,

56 Sminthopsis_bindi,

57 Sminthopsis_butleri,

58 Sminthopsis_dolichura,

59 Sminthopsis_douglasi,

60 Sminthopsis_gilberti,

61 Sminthopsis_granulipes,

62 Sminthopsis_griseoventer,

63 Sminthopsis_hirtipes,

64 Sminthopsis_leucopus,

65 Sminthopsis_longicaudata,

66 Sminthopsis_murina,

67 Sminthopsis_macroura,

68 Sminthopsis_psammophila,

69 Sminthopsis_ooldea,

70 Sminthopsis_virginiae,

71 Sminthopsis_youngsoni,

72 Sminthopsis_froggatti,

73 Sminthopsis_stalkeri,

74 Planigale_ingrami,

75 Planigale_maculata,

76 Planigale_novaeguineae,

77 Planigale_tenuirostris,

78 Planigale_sp1

;

tree TREE1 = [&R] ((((6[&length_range={5.326779,21.962128},height_95%_HPD={0.0,2.40000000104601E-5},length_95%_HPD={7.202771,14.562595},IgrBrlens{all}_range={0.2682911,0.6692465},length=10.715378240629668,IgrBrlens{all}_median=0.4386085,length_median=10.552051500000001,IgrBrlens{all}=0.44181962959770094,IgrBrlens{all}_95%_HPD={0.3363429,0.5515353},height_median=9.000000005698894E-6,height_range={0.0,4.199999999343618E-5},height=1.0055347326817217E-5]:10.552053999999995,5[&length_range={5.326779,21.962128},height_95%_HPD={0.0,2.40000000104601E-5},length_95%_HPD={7.202771,14.562595},IgrBrlens{all}_range={0.2337869,0.6210369},length=10.715378240629668,IgrBrlens{all}_median=0.3923871,length_median=10.552051500000001,IgrBrlens{all}=0.39439399740129877,IgrBrlens{all}_95%_HPD={0.302923,0.495304},height_median=9.000000005698894E-6,height_range={0.0,4.199999999343618E-5},height=1.0055347326817217E-5]:10.552053999999995)[&length_range={30.692311,73.002763},length_95%_HPD={34.050633,52.856021},length=42.378914948400656,posterior=1.0,IgrBrlens{all}_median=1.6040405,height_median=10.552063,height_range={5.326782000000009,21.962128},height_95%_HPD={7.202795999999999,14.562611000000004},IgrBrlens{all}_range={1.135162,2.283196},length_median=41.684593,IgrBrlens{all}=1.6145754765117426,IgrBrlens{all}_95%_HPD={1.290054,1.947952},height=10.715388295977071]:41.55054700000001,(7[&length_range={22.718807,60.55053},height_95%_HPD={0.0,2.400000000335467E-5},length_95%_HPD={28.124038,44.038901},IgrBrlens{all}_range={0.8668103,2.002214},length=35.36387718290845,IgrBrlens{all}_median=1.372519,length_median=34.7362705,IgrBrlens{all}=1.3787000958770634,IgrBrlens{all}_95%_HPD={1.07791,1.664107},height_median=9.000000005698894E-6,height_range={0.0,4.999999998744897E-5},height=1.0053098451287308E-5]:34.7415635,((((((76[&length_range={1.375617,9.585059},height_95%_HPD={9.999999974752427E-7,1.8999999994662176E-5},length_95%_HPD={1.686063,5.064036},IgrBrlens{all}_range={0.008367094,0.2910997},length=3.2240704641429345,IgrBrlens{all}_median=0.08672334000000001,length_median=3.056425,IgrBrlens{all}=0.0924011879852572,IgrBrlens{all}_95%_HPD={0.02440529,0.1629807},height_median=1.0000000003174137E-5,height_range={0.0,3.499999999689862E-5},height=1.0117191404862671E-5]:3.085203999999994,75[&length_range={1.375617,9.592944},height_95%_HPD={9.999999974752427E-7,1.8999999994662176E-5},length_95%_HPD={1.677992,5.94823},IgrBrlens{all}_range={0.02745364,0.324309},length=3.4381310843328365,IgrBrlens{all}_median=0.11816789999999999,length_median=3.1822905,IgrBrlens{all}=0.12412145278735624,IgrBrlens{all}_95%_HPD={0.05309723,0.2124568},height_median=1.0000000003174137E-5,height_range={0.0,3.499999999689862E-5},height=1.011569215448188E-5]:3.085203999999994)[&length_range={0.063012,8.261949},length_95%_HPD={0.835886,5.211982},length=2.9907129105121353,posterior=0.9270364817591205,IgrBrlens{all}_median=0.09292218,height_median=3.085213999999997,height_range={1.3756230000000045,8.904102000000009},height_95%_HPD={1.7489300000000085,5.062240000000003},IgrBrlens{all}_range={0.001094099,0.2248007},length_median=2.938484,IgrBrlens{all}=0.09482291285283011,IgrBrlens{all}_95%_HPD={0.02867145,0.1623896},height=3.237525354851752]:2.9202660000000016,74[&length_range={2.841002,13.316458},height_95%_HPD={9.999999974752427E-7,1.8000000011397788E-5},length_95%_HPD={3.950315,8.585189},IgrBrlens{all}_range={0.1072181,0.3303884},length=6.237809434407792,IgrBrlens{all}_median=0.19925005,length_median=6.1572,IgrBrlens{all}=0.20074678143428257,IgrBrlens{all}_95%_HPD={0.1464377,0.2558343},height_median=1.0000000003174137E-5,height_range={0.0,3.699999999184911E-5},height=1.013568215944134E-5]:6.005469999999995)[&length_range={0.004349,5.881976},length_95%_HPD={0.0071,2.728323},length=1.2927386746968477,posterior=0.7727386306846576,IgrBrlens{all}_median=0.02603858,height_median=6.005479999999999,height_range={2.8410160000000033,11.34739599999999},height_95%_HPD={3.948174999999985,8.34885899999999},IgrBrlens{all}_range={3.538506E-8,0.1027529},length_median=1.184078,IgrBrlens{all}=0.027479502245136903,IgrBrlens{all}_95%_HPD={3.538506E-8,0.05566633},height=6.086540691188357]:1.2268895000000093,(30[&length_range={1.903725,8.724997},height_95%_HPD={9.999999974752427E-7,1.8999999994662176E-5},length_95%_HPD={2.472956,5.67616},IgrBrlens{all}_range={0.03366899,0.1993381},length=3.9587173494502883,IgrBrlens{all}_median=0.09726299,length_median=3.8737725000000003,IgrBrlens{all}=0.09852082205272393,IgrBrlens{all}_95%_HPD={0.05837325,0.1410231},height_median=1.0000000003174137E-5,height_range={0.0,3.699999999184911E-5},height=1.012443778171101E-5]:3.869779499999993,(78[&length_range={1.566629,6.921593},height_95%_HPD={9.999999974752427E-7,1.8999999994662176E-5},length_95%_HPD={1.925552,4.680449},IgrBrlens{all}_range={0.04380637,0.338024},length=3.1863343214642597,IgrBrlens{all}_median=0.13548264999999998,length_median=3.0983634999999996,IgrBrlens{all}=0.13831712297601173,IgrBrlens{all}_95%_HPD={0.0817989,0.2009709},height_median=1.0000000003174137E-5,height_range={0.0,3.5999999994373866E-5},height=1.0148425787550685E-5]:3.073752000000006,77[&length_range={1.504041,6.921593},height_95%_HPD={9.999999974752427E-7,1.8999999994662176E-5},length_95%_HPD={1.861076,4.624043},IgrBrlens{all}_range={0.04709968,0.3912204},length=3.132789173413288,IgrBrlens{all}_median=0.1680375,length_median=3.045804,IgrBrlens{all}=0.1714213433308345,IgrBrlens{all}_95%_HPD={0.09211356,0.2543087},height_median=1.0000000003174137E-5,height_range={0.0,3.5999999994373866E-5},height=1.0149550225344933E-5]:3.073752000000006)[&length_range={0.01116,3.968514},length_95%_HPD={0.051703,1.676833},length=0.7917241582253107,posterior=0.9349075462268865,IgrBrlens{all}_median=0.03003659,height_median=3.073762000000009,height_range={1.5666390000000092,6.921596000000022},height_95%_HPD={1.8909209999999987,4.626884000000004},IgrBrlens{all}_range={2.228233E-8,0.1648907},length_median=0.710847,IgrBrlens{all}=0.03235686038249121,IgrBrlens{all}_95%_HPD={0.00654281,0.06267467},height=3.161490561004937]:0.7960274999999868)[&length_range={0.032171,8.521381},length_95%_HPD={1.298866,5.285491},length=3.298598092883791,posterior=0.9375312343828086,IgrBrlens{all}_median=0.15573105,height_median=3.869789499999996,height_range={1.9037389999999945,8.725019999999986},height_95%_HPD={2.5002349999999964,5.689076999999983},IgrBrlens{all}_range={1.318529E-7,0.2686658},length_median=3.2504165,IgrBrlens{all}=0.15656105814103868,IgrBrlens{all}_95%_HPD={0.109197,0.2079616},height=3.9558614513592847]:3.362580000000012)[&length_range={6.1979,21.27353},length_95%_HPD={8.520133,15.459597},length=11.832742221014461,posterior=1.0,IgrBrlens{all}_median=0.42825630000000003,height_median=7.232369500000008,height_range={4.128060000000005,13.565342999999999},height_95%_HPD={5.182684000000009,9.714885999999993},IgrBrlens{all}_range={0.3015056,0.6402134},length_median=11.65646,IgrBrlens{all}=0.4305894297476255,IgrBrlens{all}_95%_HPD={0.3459855,0.5215858},height=7.3593689147926025]:11.67190849999999,((((((63[&length_range={3.703881,14.754019},height_95%_HPD={9.999999903698154E-7,1.8000000011397788E-5},length_95%_HPD={4.997526,10.410197},IgrBrlens{all}_range={0.1754712,0.5009398},length=7.689635818465767,IgrBrlens{all}_median=0.3062465,length_median=7.581322500000001,IgrBrlens{all}=0.3091210164667662,IgrBrlens{all}_95%_HPD={0.2198483,0.3978818},height_median=1.0000000003174137E-5,height_range={0.0,3.6000000022795575E-5},height=1.0132683658706385E-5]:7.45973399999999,71[&length_range={3.703881,14.754019},height_95%_HPD={9.99999983264388E-7,1.8000000018503215E-5},length_95%_HPD={5.002707,10.408512},IgrBrlens{all}_range={0.2100445,0.5777199},length=7.663173204147916,IgrBrlens{all}_median=0.35023614999999997,length_median=7.55739,IgrBrlens{all}=0.3544820808845575,IgrBrlens{all}_95%_HPD={0.2628638,0.4571036},height_median=1.0000000003174137E-5,height_range={0.0,3.6000000022795575E-5},height=1.0138055972566083E-5]:7.45973399999999)[&length_range={0.025755,5.514358},length_95%_HPD={0.115494,2.583665},length=1.2310399072334057,posterior=0.8377061469265368,IgrBrlens{all}_median=0.03763356,height_median=7.4597439999999935,height_range={3.703891999999996,14.754025000000013},height_95%_HPD={5.002711999999995,10.328756999999996},IgrBrlens{all}_range={5.9088E-5,0.1381698},length_median=1.109672,IgrBrlens{all}=0.03933450134262493,IgrBrlens{all}_95%_HPD={0.004632917,0.07392579},height=7.566586712900812]:1.1834695000000046,68[&length_range={4.599713,16.063504},height_95%_HPD={9.999999903698154E-7,1.899999998045132E-5},length_95%_HPD={5.871019,11.517687},IgrBrlens{all}_range={0.1964022,0.4761756},length=8.640277216516761,IgrBrlens{all}_median=0.3001624,length_median=8.502314,IgrBrlens{all}=0.30309801841579104,IgrBrlens{all}_95%_HPD={0.2324108,0.3773311},height_median=1.0000000003174137E-5,height_range={0.0,3.500000001110948E-5},height=1.013680659725867E-5]:8.643203499999995)[&length_range={0.022002,5.744632},length_95%_HPD={0.253907,3.193991},length=1.6812826063218496,posterior=1.0,IgrBrlens{all}_median=0.054335055,height_median=8.643213499999998,height_range={4.599723999999995,16.063506999999987},height_95%_HPD={6.18917900000001,11.689772000000005},IgrBrlens{all}_range={0.009279521,0.1730415},length_median=1.5823195,IgrBrlens{all}=0.05580541001061957,IgrBrlens{all}_95%_HPD={0.02361973,0.0915938},height=8.7750413348326]:1.6226990000000079,61[&length_range={6.374951,18.720522},height_95%_HPD={9.999999974752427E-7,1.8000000011397788E-5},length_95%_HPD={7.696878,13.48623},IgrBrlens{all}_range={0.2478121,0.6264822},length=10.458185694527714,IgrBrlens{all}_median=0.4025653,length_median=10.279868,IgrBrlens{all}=0.40580905162418945,IgrBrlens{all}_95%_HPD={0.3077722,0.5093868},height_median=1.0000000003174137E-5,height_range={0.0,3.4000000013634235E-5},height=1.0140304848044504E-5]:10.265902500000003)[&length_range={0.048098,5.221784},length_95%_HPD={0.154948,2.501334},length=1.2311714342973077,posterior=0.9850074962518741,IgrBrlens{all}_median=0.0334511,height_median=10.265912500000006,height_range={6.374953999999988,18.720551999999998},height_95%_HPD={7.710168000000003,13.48624499999999},IgrBrlens{all}_range={4.381703E-4,0.09598821},length_median=1.137513,IgrBrlens{all}=0.03471609338196351,IgrBrlens{all}_95%_HPD={0.009331652,0.06218305},height=10.444710536022324]:1.3386024999999933,((62[&length_range={1.517431,9.070054},height_95%_HPD={9.999999974752427E-7,1.8999999994662176E-5},length_95%_HPD={2.188481,5.778278},IgrBrlens{all}_range={0.05422759,0.2501122},length=3.8735816311844062,IgrBrlens{all}_median=0.12220545,length_median=3.7682785,IgrBrlens{all}=0.12409969863568239,IgrBrlens{all}_95%_HPD={0.07864507,0.1707061},height_median=1.0000000003174137E-5,height_range={0.0,3.4999999968476914E-5},height=1.0163918041343799E-5]:3.7682774999999964,54[&length_range={1.517431,9.070054},height_95%_HPD={9.999999974752427E-7,1.8999999994662176E-5},length_95%_HPD={2.188481,5.778278},IgrBrlens{all}_range={0.05258221,0.2384398},length=3.8735816311844062,IgrBrlens{all}_median=0.12331625,length_median=3.7682785,IgrBrlens{all}=0.12528920226761645,IgrBrlens{all}_95%_HPD={0.08170892,0.1737576},height_median=1.0000000003174137E-5,height_range={0.0,3.4999999968476914E-5},height=1.0163918041343799E-5]:3.7682774999999964)[&length_range={2.362146,14.539064},length_95%_HPD={4.556701,9.832067},length=7.09069750362317,posterior=1.0,IgrBrlens{all}_median=0.20770405,height_median=3.7682874999999996,height_range={1.5174500000000037,9.07006699999998},height_95%_HPD={2.188490999999999,5.778288000000003},IgrBrlens{all}_range={0.114536,0.3684746},length_median=6.9754594999999995,IgrBrlens{all}=0.21053077661169414,IgrBrlens{all}_95%_HPD={0.1458433,0.2779358},height=3.8735917951024397]:7.005725500000004,(69[&length_range={4.948674,16.301195},height_95%_HPD={9.99999983264388E-7,1.899999998045132E-5},length_95%_HPD={6.361108,11.317636},IgrBrlens{all}_range={0.1846065,0.5594184},length=8.740699003123444,IgrBrlens{all}_median=0.33533999999999997,length_median=8.6179895,IgrBrlens{all}=0.33759152331334313,IgrBrlens{all}_95%_HPD={0.2557388,0.4289783},height_median=1.0000000003174137E-5,height_range={0.0,3.3999999985212526E-5},height=1.0139430285391101E-5]:8.617988500000003,((60[&length_range={2.206456,9.402262},height_95%_HPD={9.999999974752427E-7,1.8999999994662176E-5},length_95%_HPD={2.901371,6.832132},IgrBrlens{all}_range={0.04911447,0.2221853},length=4.914772507496244,IgrBrlens{all}_median=0.11392340000000001,length_median=4.864935,IgrBrlens{all}=0.11613172475262354,IgrBrlens{all}_95%_HPD={0.07281323,0.164065},height_median=1.0000000003174137E-5,height_range={0.0,3.6000000022795575E-5},height=1.0135307346838816E-5]:4.863905999999979,66[&length_range={2.206456,9.402262},height_95%_HPD={9.999999974752427E-7,1.8999999994662176E-5},length_95%_HPD={2.882858,6.835374},IgrBrlens{all}_range={0.120112,0.3698132},length=4.921268906546717,IgrBrlens{all}_median=0.2185342,length_median=4.867796,IgrBrlens{all}=0.22059577987256349,IgrBrlens{all}_95%_HPD={0.1574358,0.2876262},height_median=1.0000000003174137E-5,height_range={0.0,3.6000000022795575E-5},height=1.0134432784117058E-5]:4.863905999999979)[&length_range={0.043985,5.899683},length_95%_HPD={0.315095,3.362724},length=1.825719709968652,posterior=0.9963768115942029,IgrBrlens{all}_median=0.04179592,height_median=4.863915999999982,height_range={2.2064689999999985,9.402279},height_95%_HPD={2.8953659999999957,6.822739999999996},IgrBrlens{all}_range={0.008165066,0.1073596},length_median=1.752824,IgrBrlens{all}=0.043242249657429545,IgrBrlens{all}_95%_HPD={0.01745976,0.07300257},height=4.9144546099059525]:2.028052000000013,(64[&length_range={2.242778,10.41389},height_95%_HPD={9.999999974752427E-7,1.8999999994662176E-5},length_95%_HPD={3.725515,7.990408},IgrBrlens{all}_range={0.1433064,0.4219891},length=5.798752380809614,IgrBrlens{all}_median=0.24851865,length_median=5.751251,IgrBrlens{all}=0.25097238538230876,IgrBrlens{all}_95%_HPD={0.1797957,0.3223909},height_median=1.0000000003174137E-5,height_range={0.0,3.500000001110948E-5},height=1.0153048476276357E-5]:6.197588999999994,(58[&length_range={2.589398,9.840078},height_95%_HPD={9.999999974752427E-7,1.8999999994662176E-5},length_95%_HPD={3.522704,7.328131},IgrBrlens{all}_range={0.1159579,0.3208125},length=5.427592577711146,IgrBrlens{all}_median=0.20418535,length_median=5.3694865,IgrBrlens{all}=0.20559350539730137,IgrBrlens{all}_95%_HPD={0.1470114,0.2593785},height_median=1.0000000003174137E-5,height_range={0.0,3.500000001110948E-5},height=1.0147801099983087E-5]:5.584246,(55[&length_range={2.50801,11.766052},height_95%_HPD={9.999999974752427E-7,1.8999999994662176E-5},length_95%_HPD={3.274384,8.357312},IgrBrlens{all}_range={0.1138776,0.4002019},length=5.767845061219392,IgrBrlens{all}_median=0.24113635,length_median=5.6486575000000006,IgrBrlens{all}=0.2431365510869571,IgrBrlens{all}_95%_HPD={0.1698345,0.319199},height_median=1.0000000003174137E-5,height_range={0.0,3.4000000013634235E-5},height=1.0159045477928247E-5]:4.9518950000000075,57[&length_range={2.242778,10.367889},height_95%_HPD={9.999999974752427E-7,1.8999999994662176E-5},length_95%_HPD={3.516234,7.666876},IgrBrlens{all}_range={0.1546227,0.4824709},length=5.348808836081949,IgrBrlens{all}_median=0.2881195,length_median=5.2935680000000005,IgrBrlens{all}=0.29081506516741545,IgrBrlens{all}_95%_HPD={0.210069,0.3680472},height_median=1.0000000003174137E-5,height_range={0.0,3.4000000013634235E-5},height=1.0156796602303351E-5]:4.9518950000000075)[&length_range={0.0033,3.75166},length_95%_HPD={0.011067,1.777644},length=0.8008992593957271,posterior=0.5086206896551724,IgrBrlens{all}_median=0.02698831,height_median=4.951905000000011,height_range={2.508032000000007,9.689686000000023},height_95%_HPD={3.127440000000007,6.950979000000004},IgrBrlens{all}_range={1.507138E-8,0.1035639},length_median=0.71128,IgrBrlens{all}=0.02847410578432731,IgrBrlens{all}_95%_HPD={1.507138E-8,0.05717058},height=5.0393987543601]:0.6323509999999928)[&length_range={0.004304,2.756579},length_95%_HPD={0.033008,1.346032},length=0.608967674612636,posterior=0.5241129435282359,IgrBrlens{all}_median=0.01605212,height_median=5.584256000000003,height_range={2.9970969999999966,10.167110999999991},height_95%_HPD={3.922013000000014,7.564179999999993},IgrBrlens{all}_range={1.419875E-6,0.07650798},length_median=0.536517,IgrBrlens{all}=0.01750017309495423,IgrBrlens{all}_95%_HPD={0.002947701,0.03552621},height=5.680107018116795]:0.6133429999999933)[&length_range={0.01077,3.052291},length_95%_HPD={0.033164,1.461961},length=0.6772328156244064,posterior=0.657296351824088,IgrBrlens{all}_median=0.02090702,height_median=6.197598999999997,height_range={3.4757029999999958,10.950101000000004},height_95%_HPD={4.412748999999998,8.208264000000014},IgrBrlens{all}_range={5.877048E-9,0.07978326},length_median=0.601393,IgrBrlens{all}=0.022043202429366315,IgrBrlens{all}_95%_HPD={0.002954649,0.042598},height=6.287770635050375]:0.6943689999999982)[&length_range={0.019589,5.496578},length_95%_HPD={0.315504,3.252656},length=1.742646045352326,posterior=1.0,IgrBrlens{all}_median=0.06655367000000001,height_median=6.891967999999995,height_range={4.159741999999987,11.774225000000015},height_95%_HPD={5.149598999999988,9.283909999999992},IgrBrlens{all}_range={0.0175733,0.1662795},length_median=1.6870265,IgrBrlens{all}=0.06768924780734645,IgrBrlens{all}_95%_HPD={0.03531883,0.1025248},height=6.9980630972014035]:1.726030500000011)[&length_range={0.09475,7.222837},length_95%_HPD={0.721311,4.228712},length=2.3560678227136402,posterior=1.0,IgrBrlens{all}_median=0.09389483500000001,height_median=8.617998500000006,height_range={4.948675999999999,16.30120500000001},height_95%_HPD={6.361125000000001,11.31765200000001},IgrBrlens{all}_range={0.0370466,0.1864636},length_median=2.2746795,IgrBrlens{all}=0.0953690228023489,IgrBrlens{all}_95%_HPD={0.05816705,0.1361715},height=8.740709142553753]:2.1560144999999977)[&length_range={0.006148,4.886286},length_95%_HPD={0.048152,1.851843},length=0.8511027526100806,posterior=0.8257121439280359,IgrBrlens{all}_median=0.016299,height_median=10.774013000000004,height_range={6.688714000000012,19.517449},height_95%_HPD={8.302570000000003,14.039183999999985},IgrBrlens{all}_range={4.765107E-5,0.07548409},length_median=0.757638,IgrBrlens{all}=0.017811409757674406,IgrBrlens{all}_95%_HPD={9.696452E-4,0.03643023},height=10.957514733847754]:0.8305019999999956)[&length_range={0.20989,6.205766},length_95%_HPD={0.473356,3.770625},length=2.1029510730884535,posterior=1.0,IgrBrlens{all}_median=0.06763504000000001,height_median=11.604515,height_range={7.807877000000005,20.49709999999999},height_95%_HPD={9.162633,15.102215999999999},IgrBrlens{all}_range={0.02710746,0.1336263},length_median=2.0121415000000002,IgrBrlens{all}=0.06860000355322338,IgrBrlens{all}_95%_HPD={0.04333681,0.09811184},height=11.810189948400803]:1.862060999999997,(65[&length_range={5.992409,18.880463},height_95%_HPD={9.999999974752427E-7,1.8000000011397788E-5},length_95%_HPD={8.059959,14.292496},IgrBrlens{all}_range={0.2795165,0.6942171},length=11.06216576424288,IgrBrlens{all}_median=0.4418952,length_median=10.928730999999999,IgrBrlens{all}=0.4451794283858078,IgrBrlens{all}_95%_HPD={0.3364383,0.5567865},height_median=1.0000000003174137E-5,height_range={0.0,3.700000002027082E-5},height=1.0148800600288142E-5]:10.928729999999998,27[&length_range={5.992409,18.880463},height_95%_HPD={9.999999974752427E-7,1.8000000011397788E-5},length_95%_HPD={8.059959,14.292496},IgrBrlens{all}_range={0.2193946,0.4873072},length=11.06216576424288,IgrBrlens{all}_median=0.33854874999999995,length_median=10.928730999999999,IgrBrlens{all}=0.3414044207646182,IgrBrlens{all}_95%_HPD={0.2692996,0.4215241},height_median=1.0000000003174137E-5,height_range={0.0,3.700000002027082E-5},height=1.0148800600288142E-5]:10.928729999999998)[&length_range={0.128999,7.968446},length_95%_HPD={0.905588,4.993051},length=2.8366015211144497,posterior=1.0,IgrBrlens{all}_median=0.0786308,height_median=10.928740000000001,height_range={5.992417999999994,18.880475000000004},height_95%_HPD={8.059971999999995,14.292504000000008},IgrBrlens{all}_range={0.02837356,0.1641002},length_median=2.7376095,IgrBrlens{all}=0.07996399817466295,IgrBrlens{all}_95%_HPD={0.04424605,0.1151875},height=11.062175913043479]:2.537835999999995)[&length_range={0.005689,3.464193},length_95%_HPD={0.056359,1.854552},length=0.8965645269143281,posterior=0.4943778110944528,IgrBrlens{all}_median=0.01761975,height_median=13.466575999999996,height_range={9.630717999999995,23.210600999999997},height_95%_HPD={10.729264999999998,17.085192},IgrBrlens{all}_range={7.681296E-4,0.05888069},length_median=0.815101,IgrBrlens{all}=0.01832262062590348,IgrBrlens{all}_95%_HPD={0.003764452,0.03416721},height=13.697567047005345]:1.207265500000009,(((56[&length_range={3.087155,13.375514},height_95%_HPD={9.999999974752427E-7,1.8999999994662176E-5},length_95%_HPD={4.759143,9.299878},IgrBrlens{all}_range={0.1427582,0.448612},length=6.862389189780131,IgrBrlens{all}_median=0.25666425,length_median=6.759522499999999,IgrBrlens{all}=0.25943036153173504,IgrBrlens{all}_95%_HPD={0.1861151,0.3373322},height_median=1.0000000003174137E-5,height_range={0.0,3.499999999689862E-5},height=1.0147801099947578E-5]:6.833938,(((72[&length_range={1.678425,8.550456},height_95%_HPD={9.999999974752427E-7,1.8999999994662176E-5},length_95%_HPD={2.34249,5.489589},IgrBrlens{all}_range={0.04757957,0.4253383},length=3.851256849700146,IgrBrlens{all}_median=0.1623564,length_median=3.772795,IgrBrlens{all}=0.1670627642778609,IgrBrlens{all}_95%_HPD={0.0817754,0.2553167},height_median=1.0000000003174137E-5,height_range={0.0,3.699999999184911E-5},height=1.013705647222552E-5]:3.77486099999998,(67[&length_range={1.159001,6.275629},height_95%_HPD={9.999999974752427E-7,1.8999999994662176E-5},length_95%_HPD={1.644313,3.790681},IgrBrlens{all}_range={0.004761941,0.1386806},length=2.619336172913556,IgrBrlens{all}_median=0.047182395,length_median=2.536752,IgrBrlens{all}=0.04915994205959509,IgrBrlens{all}_95%_HPD={0.01469185,0.08757854},height_median=1.0000000003174137E-5,height_range={0.0,3.699999999184911E-5},height=1.013230884599556E-5]:2.5302149999999983,73[&length_range={1.159001,6.275629},height_95%_HPD={9.999999974752427E-7,1.8999999994662176E-5},length_95%_HPD={1.644313,3.7891},IgrBrlens{all}_range={0.02228205,0.3276558},length=2.618045508370826,IgrBrlens{all}_median=0.11316955000000001,length_median=2.5354525,IgrBrlens{all}=0.11729616034357847,IgrBrlens{all}_95%_HPD={0.04980594,0.1892882},height_median=1.0000000003174137E-5,height_range={0.0,3.699999999184911E-5},height=1.0130059970429253E-5]:2.5302149999999983)[&length_range={0.033652,4.979889},length_95%_HPD={0.0891,2.472909},length=1.2516223992915039,posterior=0.9875062468765617,IgrBrlens{all}_median=0.038802125,height_median=2.5302250000000015,height_range={1.1590050000000147,6.275628999999995},height_95%_HPD={1.6360719999999986,3.7604490000000084},IgrBrlens{all}_range={6.531449E-5,0.140383},length_median=1.1677205,IgrBrlens{all}=0.04078618861168908,IgrBrlens{all}_95%_HPD={0.004787486,0.0804974},height=2.610967957110323]:1.2446459999999817)[&length_range={0.021434,5.040772},length_95%_HPD={0.138179,2.805158},length=1.4440358631078907,posterior=0.999375312343828,IgrBrlens{all}_median=0.0530325,height_median=3.774870999999983,height_range={1.678436000000005,8.55046999999999},height_95%_HPD={2.371750000000006,5.491749999999996},IgrBrlens{all}_range={4.686421E-4,0.1443062},length_median=1.353459,IgrBrlens{all}=0.054520054647793394,IgrBrlens{all}_95%_HPD={0.01377641,0.09694714},height=3.8567527319664965]:1.369907000000019,59[&length_range={2.521579,10.035943},height_95%_HPD={9.999999974752427E-7,1.8999999994662176E-5},length_95%_HPD={3.486587,7.056965},IgrBrlens{all}_range={0.114293,0.3372112},length=5.230164449150446,IgrBrlens{all}_median=0.19998505,length_median=5.1361735,IgrBrlens{all}=0.20222781641679105,IgrBrlens{all}_95%_HPD={0.1465494,0.2612383},height_median=1.0000000003174137E-5,height_range={0.0,3.699999999184911E-5},height=1.0148050974954377E-5]:5.144767999999999)[&length_range={0.007364,4.081971},length_95%_HPD={0.088905,1.932317},length=0.937822144273415,posterior=0.9577711144427786,IgrBrlens{all}_median=0.027447915000000003,height_median=5.144778000000002,height_range={2.5215840000000043,10.035956999999996},height_95%_HPD={3.479836000000006,7.0260920000000056},IgrBrlens{all}_range={8.709958E-4,0.08212227},length_median=0.8555345000000001,IgrBrlens{all}=0.028770077463435847,IgrBrlens{all}_95%_HPD={0.009125241,0.05250023},height=5.241657405948339]:0.9058490000000035,70[&length_range={2.82398,11.750881},height_95%_HPD={9.999999974752427E-7,1.8999999994662176E-5},length_95%_HPD={4.188808,8.212805},IgrBrlens{all}_range={0.1258965,0.4031621},length=6.120821068465768,IgrBrlens{all}_median=0.2285674,length_median=6.0340535,IgrBrlens{all}=0.23099965049974996,IgrBrlens{all}_95%_HPD={0.1642923,0.3007868},height_median=1.0000000003174137E-5,height_range={0.0,3.699999999184911E-5},height=1.0159920040458256E-5]:6.050617000000003)[&length_range={0.011115,4.54635},length_95%_HPD={0.024122,1.823507},length=0.8371748147728882,posterior=0.8829335332333833,IgrBrlens{all}_median=0.02177074,height_median=6.050627000000006,height_range={3.4703949999999963,11.750891999999993},height_95%_HPD={4.253968999999998,8.203913999999997},IgrBrlens{all}_range={1.066979E-8,0.09360897},length_median=0.74842,IgrBrlens{all}=0.023187451441045472,IgrBrlens{all}_95%_HPD={1.066979E-8,0.0461676},height=6.14755695684166]:0.7833209999999973)[&length_range={0.445114,8.469991},length_95%_HPD={1.481261,5.773994},length=3.4992476461769253,posterior=1.0,IgrBrlens{all}_median=0.1527203,height_median=6.833948000000003,height_range={3.7582579999999908,13.375526999999991},height_95%_HPD={4.963248000000007,9.225795999999988},IgrBrlens{all}_range={0.07605657,0.2893933},length_median=3.396921,IgrBrlens{all}=0.15374587587081384,IgrBrlens{all}_95%_HPD={0.1059092,0.205386},height=6.9501923329585455]:3.4296859999999967,29[&length_range={6.274286,17.409682},height_95%_HPD={9.999999903698154E-7,1.8000000011397788E-5},length_95%_HPD={7.716945,13.593192},IgrBrlens{all}_range={0.2529617,0.5549658},length=10.449429851074495,IgrBrlens{all}_median=0.3888304,length_median=10.263628,IgrBrlens{all}=0.3912162411669175,IgrBrlens{all}_95%_HPD={0.3129515,0.4746594},height_median=1.0000000003174137E-5,height_range={0.0,3.500000001110948E-5},height=1.012806096991147E-5]:10.263623999999997)[&length_range={0.404298,9.2244},length_95%_HPD={1.782809,6.044346},length=3.81592011169416,posterior=1.0,IgrBrlens{all}_median=0.15762135,height_median=10.263634,height_range={6.274293000000007,17.40969100000001},height_95%_HPD={7.716951999999999,13.593200999999993},IgrBrlens{all}_range={0.09180177,0.2706318},length_median=3.7583640000000003,IgrBrlens{all}=0.15900102955647133,IgrBrlens{all}_95%_HPD={0.1168845,0.1998493},height=10.449439979135477]:3.3248030000000135,(52[&length_range={5.116482,18.870635},height_95%_HPD={9.999999903698154E-7,1.8000000011397788E-5},length_95%_HPD={6.569821,12.216997},IgrBrlens{all}_range={0.2047969,0.5045487},length=9.25003879335335,IgrBrlens{all}_median=0.33599095,length_median=9.141983499999998,IgrBrlens{all}=0.3395554806721642,IgrBrlens{all}_95%_HPD={0.2608373,0.4286781},height_median=1.0000000003174137E-5,height_range={0.0,3.5999999994373866E-5},height=1.0112318841005917E-5]:9.141982500000001,(53[&length_range={2.083563,14.380512},height_95%_HPD={9.999999974752427E-7,1.8999999994662176E-5},length_95%_HPD={4.592437,9.63816},IgrBrlens{all}_range={0.1899442,0.549464},length=7.029345113568226,IgrBrlens{all}_median=0.32762899999999995,length_median=6.906966000000001,IgrBrlens{all}=0.33030136403048477,IgrBrlens{all}_95%_HPD={0.2444367,0.4226389},height_median=1.0000000003174137E-5,height_range={0.0,3.699999999184911E-5},height=1.0100824588192446E-5]:6.906967499999993,28[&length_range={2.083563,14.380512},height_95%_HPD={9.999999974752427E-7,1.8999999994662176E-5},length_95%_HPD={4.592437,9.63816},IgrBrlens{all}_range={0.1441393,0.4006095},length=7.029345113568226,IgrBrlens{all}_median=0.24523055,length_median=6.906966000000001,IgrBrlens{all}=0.24698285865817077,IgrBrlens{all}_95%_HPD={0.1853252,0.3150814},height_median=1.0000000003174137E-5,height_range={0.0,3.699999999184911E-5},height=1.0100824588192446E-5]:6.906967499999993)[&length_range={0.077616,6.969365},length_95%_HPD={0.504335,4.09862},length=2.220693691279364,posterior=1.0,IgrBrlens{all}_median=0.07877479500000001,height_median=6.906977499999996,height_range={2.0835680000000067,14.380528999999981},height_95%_HPD={4.59244799999999,9.638170999999993},IgrBrlens{all}_range={0.02335424,0.1675665},length_median=2.1315325,IgrBrlens{all}=0.08033678116316842,IgrBrlens{all}_95%_HPD={0.04381468,0.1205271},height=7.0293552143927664]:2.2350150000000077)[&length_range={0.913537,11.31232},length_95%_HPD={2.237171,6.881801},length=4.519708934407808,posterior=1.0,IgrBrlens{all}_median=0.17091030000000001,height_median=9.141992500000004,height_range={5.116485999999988,18.870643},height_95%_HPD={6.569833000000003,12.217011999999997},IgrBrlens{all}_range={0.08912797,0.303003},length_median=4.418638,IgrBrlens{all}=0.17230115088580658,IgrBrlens{all}_95%_HPD={0.1227772,0.2252523},height=9.250048905672095]:4.446444500000009)[&length_range={0.005111,4.675314},length_95%_HPD={0.005325,1.507298},length=0.7064374807417976,posterior=0.6130684657671165,IgrBrlens{all}_median=0.01546227,height_median=13.588437000000013,height_range={9.717366999999996,23.44022900000001},height_95%_HPD={10.709742000000006,17.170634},IgrBrlens{all}_range={0.001833758,0.05285845},length_median=0.631262,IgrBrlens{all}=0.016243220117179526,IgrBrlens{all}_95%_HPD={0.004587899,0.02916035},height=13.781188487059323]:1.0854044999999921)[&length_range={1.212458,10.605286},length_95%_HPD={2.156807,6.807045},length=4.283636654422767,posterior=1.0,IgrBrlens{all}_median=0.16252655,height_median=14.673841500000005,height_range={10.907865000000001,24.286952000000007},height_95%_HPD={11.800439000000004,18.50382900000001},IgrBrlens{all}_range={0.09631196,0.2777229},length_median=4.179655,IgrBrlens{all}=0.16391725205272323,IgrBrlens{all}_95%_HPD={0.120895,0.2079274},height=14.9084744813843]:4.230436499999993)[&length_range={0.941987,10.699417},length_95%_HPD={2.224872,7.104665},length=4.580172914542726,posterior=1.0,IgrBrlens{all}_median=0.1681831,height_median=18.904277999999998,height_range={13.231000999999992,33.043244},height_95%_HPD={14.940663,23.84238},IgrBrlens{all}_range={0.09960466,0.2731698},length_median=4.52522,IgrBrlens{all}=0.16947163460269862,IgrBrlens{all}_95%_HPD={0.1229709,0.21844},height=19.192111135807153]:4.527476500000006,(((39[&length_range={7.404347,20.660088},height_95%_HPD={1.9999999949504854E-6,1.900000000887303E-5},length_95%_HPD={8.657359,14.273588},IgrBrlens{all}_range={0.208944,0.4075469},length=11.470894901049492,IgrBrlens{all}_median=0.2961614,length_median=11.3191095,IgrBrlens{all}=0.29854297604947505,IgrBrlens{all}_95%_HPD={0.2394364,0.3602516},height_median=1.0000000003174137E-5,height_range={0.0,3.5999999994373866E-5},height=1.015579710196989E-5]:11.31911349999999,(43[&length_range={4.477076,15.750401},height_95%_HPD={9.999999974752427E-7,1.8999999994662176E-5},length_95%_HPD={5.56685,10.249381},IgrBrlens{all}_range={0.1779777,0.4417257},length=7.817881591329317,IgrBrlens{all}_median=0.28463649999999996,length_median=7.71439,IgrBrlens{all}=0.2872730891179405,IgrBrlens{all}_95%_HPD={0.2170695,0.3604301},height_median=1.0000000003174137E-5,height_range={0.0,3.500000001110948E-5},height=1.0176661669715025E-5]:7.714393999999995,(41[&length_range={2.915417,12.034694},height_95%_HPD={9.999999974752427E-7,1.8999999994662176E-5},length_95%_HPD={3.885259,7.869401},IgrBrlens{all}_range={0.1047327,0.3413199},length=5.8180695122438975,IgrBrlens{all}_median=0.20775375,length_median=5.733513,IgrBrlens{all}=0.20977140133683111,IgrBrlens{all}_95%_HPD={0.1453003,0.2728319},height_median=1.0000000003174137E-5,height_range={0.0,3.399999999942338E-5},height=1.0159920040448492E-5]:5.733515000000011,(42[&length_range={1.795713,8.693201},height_95%_HPD={9.999999974752427E-7,1.8999999994662176E-5},length_95%_HPD={2.514008,5.571199},IgrBrlens{all}_range={0.03992336,0.1619341},length=3.9865666990254787,IgrBrlens{all}_median=0.087533155,length_median=3.923321,IgrBrlens{all}=0.088943935964518,IgrBrlens{all}_95%_HPD={0.05813909,0.1222698},height_median=1.0000000003174137E-5,height_range={0.0,3.499999999689862E-5},height=1.0178035982502757E-5]:3.9242184999999985,(40[&length_range={1.297501,5.948662},height_95%_HPD={9.999999974752427E-7,1.8999999994662176E-5},length_95%_HPD={1.712376,3.779057},IgrBrlens{all}_range={0.06398053,0.247775},length=2.6494483355822083,IgrBrlens{all}_median=0.12150069999999999,length_median=2.588743,IgrBrlens{all}=0.12307908169915002,IgrBrlens{all}_95%_HPD={0.08507082,0.1640674},height_median=1.0000000003174137E-5,height_range={0.0,3.499999999689862E-5},height=1.0165792104513537E-5]:2.5871170000000063,17[&length_range={1.297501,5.941608},height_95%_HPD={9.999999974752427E-7,1.8999999994662176E-5},length_95%_HPD={1.716433,3.779057},IgrBrlens{all}_range={0.03084476,0.1620753},length=2.647367941529234,IgrBrlens{all}_median=0.07690227,length_median=2.5871755,IgrBrlens{all}=0.07801033158920546,IgrBrlens{all}_95%_HPD={0.04655086,0.1094986},height_median=1.0000000003174137E-5,height_range={0.0,3.499999999689862E-5},height=1.016529235438631E-5]:2.5871170000000063)[&length_range={0.058683,4.846851},length_95%_HPD={0.259635,2.509732},length=1.3417132253097992,posterior=0.9981259370314842,IgrBrlens{all}_median=0.06568873,height_median=2.5871270000000095,height_range={1.2975130000000021,5.94162},height_95%_HPD={1.6964169999999967,3.755365999999988},IgrBrlens{all}_range={0.02505934,0.139576},length_median=1.274361,IgrBrlens{all}=0.06681454569783438,IgrBrlens{all}_95%_HPD={0.03869162,0.09798992},height=2.646729130930033]:1.3371014999999922)[&length_range={0.083098,6.262722},length_95%_HPD={0.426287,3.312383},length=1.8294224005497202,posterior=1.0,IgrBrlens{all}_median=0.060803590000000005,height_median=3.9242285000000017,height_range={1.9311690000000041,8.693218000000002},height_95%_HPD={2.5140150000000077,5.568182999999991},IgrBrlens{all}_range={0.014863,0.1377162},length_median=1.752874,IgrBrlens{all}=0.06180031595327326,IgrBrlens{all}_95%_HPD={0.03249404,0.09451048},height=3.9886572716141906]:1.8092965000000127)[&length_range={0.134275,6.737011},length_95%_HPD={0.478864,3.660488},length=1.999812095827079,posterior=1.0,IgrBrlens{all}_median=0.061258975,height_median=5.733525000000014,height_range={2.9154219999999924,12.03469800000002},height_95%_HPD={3.8852699999999984,7.8694090000000045},IgrBrlens{all}_range={0.01606418,0.1382719},length_median=1.913431,IgrBrlens{all}=0.0626821834232883,IgrBrlens{all}_95%_HPD={0.03291007,0.09551024},height=5.818079672163924]:1.9808789999999838)[&length_range={0.610859,9.2569},length_95%_HPD={1.601532,5.794895},length=3.6530132888555658,posterior=1.0,IgrBrlens{all}_median=0.1182681,height_median=7.714403999999998,height_range={4.477080999999991,15.750405000000015},height_95%_HPD={5.566856000000001,10.249397000000002},IgrBrlens{all}_range={0.0550042,0.2114764},length_median=3.5814675,IgrBrlens{all}=0.1196784756171916,IgrBrlens{all}_95%_HPD={0.08077992,0.1622367},height=7.817891767990982]:3.6047194999999945)[&length_range={0.073104,6.476189},length_95%_HPD={0.349721,3.333368},length=1.7264099888805615,posterior=1.0,IgrBrlens{all}_median=0.03198692,height_median=11.319123499999993,height_range={7.404358000000009,20.66011300000001},height_95%_HPD={8.657364000000001,14.273603999999999},IgrBrlens{all}_range={0.007633645,0.07694306},length_median=1.6218655,IgrBrlens{all}=0.032567926394177944,IgrBrlens{all}_95%_HPD={0.01619619,0.05071964},height=11.470905056846556]:1.6743725000000182,((9[&length_range={6.939726,21.605256},height_95%_HPD={9.999999974752427E-7,1.8000000011397788E-5},length_95%_HPD={8.61739,14.072135},IgrBrlens{all}_range={0.2867837,0.5557662},length=11.248956285482276,IgrBrlens{all}_median=0.4028977,length_median=11.0964235,IgrBrlens{all}=0.40475909367816093,IgrBrlens{all}_95%_HPD={0.3267616,0.4839208},height_median=1.0000000003174137E-5,height_range={0.0,3.499999999689862E-5},height=1.0167291354797565E-5]:11.171188999999995,(((37[&length_range={2.868805,13.30583},height_95%_HPD={9.999999974752427E-7,1.8999999994662176E-5},length_95%_HPD={3.838633,7.788301},IgrBrlens{all}_range={0.113506,0.3294139},length=5.788981324212869,IgrBrlens{all}_median=0.19295345,length_median=5.721434,IgrBrlens{all}=0.1944292723638191,IgrBrlens{all}_95%_HPD={0.1388675,0.2506057},height_median=1.0000000003174137E-5,height_range={0.0,3.499999999689862E-5},height=1.0172038981042617E-5]:5.721431500000001,(38[&length_range={2.150051,10.491327},height_95%_HPD={9.999999974752427E-7,1.8999999994662176E-5},length_95%_HPD={2.875276,6.077424},IgrBrlens{all}_range={0.08266552,0.2882155},length=4.404948399800102,IgrBrlens{all}_median=0.1609032,length_median=4.3494145,IgrBrlens{all}=0.1625955587393802,IgrBrlens{all}_95%_HPD={0.1141419,0.2143849},height_median=1.0000000003174137E-5,height_range={0.0,3.5999999994373866E-5},height=1.0171164418320858E-5]:4.349419000000001,(36[&length_range={1.250294,6.573836},height_95%_HPD={9.99999983264388E-7,1.899999998045132E-5},length_95%_HPD={1.640727,3.706494},IgrBrlens{all}_range={0.02730215,0.1602105},length=2.633667184532731,IgrBrlens{all}_median=0.07692278999999999,length_median=2.5635890000000003,IgrBrlens{all}=0.07854619232133947,IgrBrlens{all}_95%_HPD={0.04526669,0.1147814},height_median=1.0000000003174137E-5,height_range={0.0,3.5999999994373866E-5},height=1.0161919040965389E-5]:2.563594000000009,13[&length_range={1.250294,6.573836},height_95%_HPD={9.99999983264388E-7,1.899999998045132E-5},length_95%_HPD={1.640727,3.706494},IgrBrlens{all}_range={0.04102256,0.1979674},length=2.633667184532731,IgrBrlens{all}_median=0.09464412,length_median=2.5635890000000003,IgrBrlens{all}=0.09606448026111954,IgrBrlens{all}_95%_HPD={0.05882081,0.1319117},height_median=1.0000000003174137E-5,height_range={0.0,3.5999999994373866E-5},height=1.0161919040965389E-5]:2.563594000000009)[&length_range={0.127535,5.216107},length_95%_HPD={0.533958,3.12944},length=1.7712812245127412,posterior=1.0,IgrBrlens{all}_median=0.08072389499999999,height_median=2.563604000000012,height_range={1.2503009999999932,6.573863000000031},height_95%_HPD={1.640745999999993,3.7065100000000086},IgrBrlens{all}_range={0.0252796,0.173765},length_median=1.7190325,IgrBrlens{all}=0.08221447228010972,IgrBrlens{all}_95%_HPD={0.04487311,0.1200372},height=2.6336773464517664]:1.785824999999992)[&length_range={0.058239,4.762472},length_95%_HPD={0.260627,2.68604},length=1.3840329252873529,posterior=1.0,IgrBrlens{all}_median=0.0540613,height_median=4.349429000000004,height_range={2.150058999999999,10.491333000000012},height_95%_HPD={2.8752860000000027,6.0774319999999875},IgrBrlens{all}_range={0.008971877,0.1331577},length_median=1.2968039999999998,IgrBrlens{all}=0.05515720613655708,IgrBrlens{all}_95%_HPD={0.02470039,0.08654033},height=4.40495857096451]:1.3720125000000003)[&length_range={0.40845,8.181325},length_95%_HPD={1.683175,5.710836},length=3.5977667717391304,posterior=1.0,IgrBrlens{all}_median=0.15394845000000001,height_median=5.7214415000000045,height_range={2.8688120000000126,13.305837000000011},height_95%_HPD={3.838644000000002,7.788319000000008},IgrBrlens{all}_range={0.08337206,0.2592297},length_median=3.5432385,IgrBrlens{all}=0.15523321501124376,IgrBrlens{all}_95%_HPD={0.1100131,0.2022146},height=5.788991496251856]:3.553902000000001,16[&length_range={4.658829,18.734128},height_95%_HPD={9.999999903698154E-7,1.800000000429236E-5},length_95%_HPD={6.79717,11.889703},IgrBrlens{all}_range={0.2783062,0.5467778},length=9.386748092578701,IgrBrlens{all}_median=0.38186115,length_median=9.2753365,IgrBrlens{all}=0.3837950904422809,IgrBrlens{all}_95%_HPD={0.3099345,0.4629627},height_median=1.0000000003174137E-5,height_range={0.0,3.499999999689862E-5},height=1.0175412294448448E-5]:9.275333500000002)[&length_range={0.026458,4.567584},length_95%_HPD={0.209602,2.483993},length=1.2980320667166427,posterior=1.0,IgrBrlens{all}_median=0.042245254999999995,height_median=9.275343500000005,height_range={4.658845000000007,18.734154000000018},height_95%_HPD={6.797189000000003,11.889703999999988},IgrBrlens{all}_range={0.01435033,0.1036776},length_median=1.234135,IgrBrlens{all}=0.04297522074962512,IgrBrlens{all}_95%_HPD={0.02171304,0.06419949},height=9.386758267990967]:1.2112405000000024,(10[&length_range={4.245568,17.179977},height_95%_HPD={9.99999983264388E-7,1.8000000011397788E-5},length_95%_HPD={5.758202,10.757366},IgrBrlens{all}_range={0.2104433,0.4638745},length=8.080115655922066,IgrBrlens{all}_median=0.31770865000000004,length_median=7.962108000000001,IgrBrlens{all}=0.31941225396052036,IgrBrlens{all}_95%_HPD={0.2553024,0.3860915},height_median=1.0000000003174137E-5,height_range={0.0,3.399999999942338E-5},height=1.016766616751283E-5]:7.962104,11[&length_range={4.245568,17.179977},height_95%_HPD={9.99999983264388E-7,1.8000000011397788E-5},length_95%_HPD={5.758202,10.757366},IgrBrlens{all}_range={0.2127557,0.4712776},length=8.080115655922066,IgrBrlens{all}_median=0.31059435,length_median=7.962108000000001,IgrBrlens{all}=0.31299432196401733,IgrBrlens{all}_95%_HPD={0.2491063,0.376376},height_median=1.0000000003174137E-5,height_range={0.0,3.399999999942338E-5},height=1.016766616751283E-5]:7.962104)[&length_range={0.453928,5.996416},length_95%_HPD={0.889069,4.225001},length=2.5126581493003584,posterior=1.0,IgrBrlens{all}_median=0.084534765,height_median=7.962114000000003,height_range={4.245578999999999,17.18000600000002},height_95%_HPD={5.758211000000003,10.757375000000003},IgrBrlens{all}_range={0.03257514,0.1472669},length_median=2.4439215,IgrBrlens{all}=0.08529946265742129,IgrBrlens{all}_95%_HPD={0.05810439,0.1152875},height=8.08012582358821]:2.5244700000000044)[&length_range={0.00371,2.974402},length_95%_HPD={0.029321,1.554876},length=0.7365634248878908,posterior=0.8915542228885557,IgrBrlens{all}_median=0.01753644,height_median=10.486584000000008,height_range={7.336314000000002,19.14591300000002},height_95%_HPD={8.170482,13.300222999999988},IgrBrlens{all}_range={0.001276104,0.05219922},length_median=0.6679475,IgrBrlens{all}=0.01818095983716365,IgrBrlens{all}_95%_HPD={0.005351484,0.0309054},height=10.625308960482046]:0.6846149999999902)[&length_range={0.047603,3.839338},length_95%_HPD={0.19331,2.303854},length=1.1682892091454282,posterior=1.0,IgrBrlens{all}_median=0.036573485,height_median=11.171198999999998,height_range={8.016871000000002,21.605267999999995},height_95%_HPD={8.762880000000003,14.120749000000004},IgrBrlens{all}_range={0.01293999,0.07793082},length_median=1.0939835,IgrBrlens{all}=0.037283099873813036,IgrBrlens{all}_95%_HPD={0.02188947,0.0550388},height=11.341980318590693]:1.1477525000000028,((((33[&length_range={3.016123,11.053377},height_95%_HPD={9.999999974752427E-7,1.8999999994662176E-5},length_95%_HPD={4.33855,8.109352},IgrBrlens{all}_range={0.1463043,0.3813557},length=6.1606854886306746,IgrBrlens{all}_median=0.2344929,length_median=6.09919,IgrBrlens{all}=0.23696876143178425,IgrBrlens{all}_95%_HPD={0.180005,0.3020971},height_median=1.0000000003174137E-5,height_range={0.0,3.5999999994373866E-5},height=1.0179160420261498E-5]:6.1645619999999965,(35[&length_range={2.163904,10.428717},height_95%_HPD={1.00000000458067E-6,1.8999999994662176E-5},length_95%_HPD={3.872314,7.482984},IgrBrlens{all}_range={0.0880924,0.3106159},length=5.62134027398799,IgrBrlens{all}_median=0.1726564,length_median=5.573321,IgrBrlens{all}=0.17477046197651303,IgrBrlens{all}_95%_HPD={0.1202295,0.2319701},height_median=1.0000000003174137E-5,height_range={0.0,3.499999998268777E-5},height=1.0157921040039898E-5]:5.545933499999997,(31[&length_range={2.028963,9.165415},height_95%_HPD={9.999999974752427E-7,1.8999999994662176E-5},length_95%_HPD={3.139349,6.376444},IgrBrlens{all}_range={0.1083617,0.3072099},length=4.769658361069465,IgrBrlens{all}_median=0.18415395,length_median=4.70944,IgrBrlens{all}=0.18580106384307832,IgrBrlens{all}_95%_HPD={0.1377875,0.2402098},height_median=1.0000000003174137E-5,height_range={0.0,3.5999999994373866E-5},height=1.0155672164528632E-5]:4.672531999999997,(34[&length_range={1.17007,5.187725},height_95%_HPD={9.999999974752427E-7,1.8999999994662176E-5},length_95%_HPD={1.625611,3.459807},IgrBrlens{all}_range={0.03287441,0.1700711},length=2.471067935282367,IgrBrlens{all}_median=0.083232745,length_median=2.4116865,IgrBrlens{all}=0.085156148223388,IgrBrlens{all}_95%_HPD={0.05006977,0.119325},height_median=1.0000000003174137E-5,height_range={0.0,3.3999999985212526E-5},height=1.0167291354923624E-5]:2.411681499999993,32[&length_range={1.17007,5.187725},height_95%_HPD={9.999999974752427E-7,1.8999999994662176E-5},length_95%_HPD={1.625611,3.459807},IgrBrlens{all}_range={0.01910881,0.1143635},length=2.471067935282367,IgrBrlens{all}_median=0.055356395,length_median=2.4116865,IgrBrlens{all}=0.05642077612943508,IgrBrlens{all}_95%_HPD={0.03145759,0.08246984},height_median=1.0000000003174137E-5,height_range={0.0,3.3999999985212526E-5},height=1.0167291354923624E-5]:2.411681499999993)[&length_range={0.184917,5.453744},length_95%_HPD={0.940664,3.721164},length=2.262593438405789,posterior=1.0,IgrBrlens{all}_median=0.10546645,height_median=2.4116914999999963,height_range={1.1700769999999991,5.187726999999995},height_95%_HPD={1.6256140000000059,3.459817000000001},IgrBrlens{all}_range={0.04675766,0.1949413},length_median=2.2226825,IgrBrlens{all}=0.1067032438268368,IgrBrlens{all}_95%_HPD={0.07168814,0.1450883},height=2.4710781025737125]:2.2608505000000036)[&length_range={0.028132,4.037594},length_95%_HPD={0.089857,1.840668},length=0.8775877098967202,posterior=0.9556471764117941,IgrBrlens{all}_median=0.02800759,height_median=4.672542,height_range={2.028967999999999,9.16544500000002},height_95%_HPD={3.2416330000000073,6.383796000000004},IgrBrlens{all}_range={0.003431397,0.07821055},length_median=0.807152,IgrBrlens{all}=0.028970550047065075,IgrBrlens{all}_95%_HPD={0.01067692,0.04842515},height=4.732027807425807]:0.8734014999999999)[&length_range={0.002582,3.860216},length_95%_HPD={0.027503,1.47424},length=0.6582011248903158,posterior=0.8543228385807097,IgrBrlens{all}_median=0.0126753,height_median=5.5459435,height_range={3.024016000000003,9.75891500000003},height_95%_HPD={3.906521000000005,7.314301999999998},IgrBrlens{all}_range={3.374136E-8,0.05526852},length_median=0.582416,IgrBrlens{all}=0.013950803268137635,IgrBrlens{all}_95%_HPD={2.28687E-4,0.02896402},height=5.595148178122271]:0.6186284999999998)[&length_range={0.084098,6.20704},length_95%_HPD={0.506179,3.481606},length=1.9582851640429673,posterior=1.0,IgrBrlens{all}_median=0.075152375,height_median=6.164572,height_range={3.5708170000000052,11.053382000000006},height_95%_HPD={4.441760000000009,8.148800999999992},IgrBrlens{all}_range={0.03116502,0.1426183},length_median=1.9005,IgrBrlens{all}=0.07605942305097438,IgrBrlens{all}_95%_HPD={0.04835301,0.1064158},height=6.236124925787111]:1.9065809999999992,12[&length_range={4.92243,15.519101},height_95%_HPD={9.999999974752427E-7,1.8000000011397788E-5},length_95%_HPD={6.070192,10.53616},IgrBrlens{all}_range={0.2778762,0.5652412},length=8.194399926161894,IgrBrlens{all}_median=0.3956931,length_median=8.0711415,IgrBrlens{all}=0.3983223781609198,IgrBrlens{all}_95%_HPD={0.3180777,0.4797109},height_median=1.0000000003174137E-5,height_range={0.0,3.5999999994373866E-5},height=1.0163668166368958E-5]:8.071142999999996)[&length_range={0.08992,5.344313},length_95%_HPD={0.359422,2.782463},length=1.5194966144427833,posterior=1.0,IgrBrlens{all}_median=0.06996353,height_median=8.071152999999999,height_range={4.9224419999999895,15.519115000000014},height_95%_HPD={6.0702,10.536164999999983},IgrBrlens{all}_range={0.02641747,0.1330122},length_median=1.4534405,IgrBrlens{all}=0.07078364205647154,IgrBrlens{all}_95%_HPD={0.04633021,0.09824605},height=8.194410089830072]:1.5025799999999983,18[&length_range={6.292398,17.948285},height_95%_HPD={9.999999903698154E-7,1.8000000011397788E-5},length_95%_HPD={7.342646,12.235003},IgrBrlens{all}_range={0.2886772,0.5727067},length=9.71389653598199,IgrBrlens{all}_median=0.40535525,length_median=9.5737215,IgrBrlens{all}=0.4078649298600715,IgrBrlens{all}_95%_HPD={0.3287982,0.4929572},height_median=1.0000000003174137E-5,height_range={0.0,3.700000000605996E-5},height=1.016829085498544E-5]:9.573722999999994)[&length_range={0.055315,5.231575},length_95%_HPD={0.163963,2.679118},length=1.4587698621939065,posterior=1.0,IgrBrlens{all}_median=0.066982925,height_median=9.573732999999997,height_range={6.292417,17.94829800000001},height_95%_HPD={7.342666999999992,12.235019000000008},IgrBrlens{all}_range={0.03150649,0.1287093},length_median=1.407106,IgrBrlens{all}=0.06776880422163904,IgrBrlens{all}_95%_HPD={0.04415165,0.09118007},height=9.71390670427286]:1.4171109999999985,(15[&length_range={2.886571,11.837605},height_95%_HPD={9.999999974752427E-7,1.8999999994662176E-5},length_95%_HPD={4.028496,8.410214},IgrBrlens{all}_range={0.1779179,0.3765348},length=6.1372273117191645,IgrBrlens{all}_median=0.25487210000000005,length_median=6.057227,IgrBrlens{all}=0.25629217140179883,IgrBrlens{all}_95%_HPD={0.2016354,0.3084395},height_median=1.0000000003174137E-5,height_range={0.0,3.699999999184911E-5},height=1.018253373362738E-5]:6.057229499999991,14[&length_range={2.886571,11.837605},height_95%_HPD={9.999999974752427E-7,1.8999999994662176E-5},length_95%_HPD={4.028496,8.410214},IgrBrlens{all}_range={0.1573039,0.3578357},length=6.1372273117191645,IgrBrlens{all}_median=0.23541505000000001,length_median=6.057227,IgrBrlens{all}=0.2367059231384306,IgrBrlens{all}_95%_HPD={0.1883,0.2914649},height_median=1.0000000003174137E-5,height_range={0.0,3.699999999184911E-5},height=1.018253373362738E-5]:6.057229499999991)[&length_range={1.531024,10.896474},length_95%_HPD={2.935468,7.384267},length=5.035439072213883,posterior=1.0,IgrBrlens{all}_median=0.20797955,height_median=6.057239499999994,height_range={2.8865830000000017,11.83762200000001},height_95%_HPD={4.028505000000003,8.410226000000002},IgrBrlens{all}_range={0.1323918,0.3120778},length_median=4.981469,IgrBrlens{all}=0.20963009410294908,IgrBrlens{all}_95%_HPD={0.1645081,0.2600061},height=6.137237494252886]:4.933604500000001)[&length_range={0.080141,4.554558},length_95%_HPD={0.246967,2.437847},length=1.3375929612693676,posterior=1.0,IgrBrlens{all}_median=0.06975382,height_median=10.990843999999996,height_range={7.6663250000000005,19.555741999999995},height_95%_HPD={8.609668999999997,13.877419000000003},IgrBrlens{all}_range={0.03567268,0.1228483},length_median=1.261549,IgrBrlens{all}=0.07058077971014498,IgrBrlens{all}_95%_HPD={0.04697708,0.09468091},height=11.17267656646676]:1.328107500000005)[&length_range={0.035493,3.344804},length_95%_HPD={0.071663,1.470387},length=0.6870455179910044,posterior=1.0,IgrBrlens{all}_median=0.025527535,height_median=12.3189515,height_range={8.895754999999994,23.257481999999996},height_95%_HPD={9.806248000000004,15.494346},IgrBrlens{all}_range={0.007943524,0.05860771},length_median=0.6133765,IgrBrlens{all}=0.026002981671914054,IgrBrlens{all}_95%_HPD={0.01264883,0.03874627},height=12.510269527736096]:0.6745445000000103)[&length_range={1.838152,11.597342},length_95%_HPD={3.467171,8.444047},length=5.931738601949018,posterior=1.0,IgrBrlens{all}_median=0.2894433,height_median=12.993496000000011,height_range={9.219664000000002,23.96060399999999},height_95%_HPD={10.427447999999998,16.483869999999996},IgrBrlens{all}_range={0.1973905,0.4305673},length_median=5.895545500000001,IgrBrlens{all}=0.2911464960269863,IgrBrlens{all}_95%_HPD={0.2294,0.3561199},height=13.197315045727144]:5.849146499999989,(((51[&length_range={2.981549,12.170133},height_95%_HPD={9.999999974752427E-7,1.8999999994662176E-5},length_95%_HPD={4.347928,9.200029},IgrBrlens{all}_range={0.1364919,0.3337564},length=6.667631032358816,IgrBrlens{all}_median=0.2155088,length_median=6.585167500000001,IgrBrlens{all}=0.21693903063468284,IgrBrlens{all}_95%_HPD={0.1689019,0.2693473},height_median=1.0000000003174137E-5,height_range={0.0,3.8999999986799594E-5},height=1.0114317841507723E-5]:6.585165499999999,26[&length_range={2.981549,12.170133},height_95%_HPD={9.999999974752427E-7,1.8999999994662176E-5},length_95%_HPD={4.347928,9.200029},IgrBrlens{all}_range={0.173448,0.3698039},length=6.667631032358816,IgrBrlens{all}_median=0.25049135,length_median=6.585167500000001,IgrBrlens{all}=0.2523391618065972,IgrBrlens{all}_95%_HPD={0.2017785,0.3080564},height_median=1.0000000003174137E-5,height_range={0.0,3.8999999986799594E-5},height=1.0114317841507723E-5]:6.585165499999999)[&length_range={1.950154,11.629787},length_95%_HPD={3.281693,8.115728},length=5.646215930284874,posterior=1.0,IgrBrlens{all}_median=0.17707115,height_median=6.585175500000002,height_range={2.9815599999999876,12.17015099999999},height_95%_HPD={4.347936000000011,9.200057000000001},IgrBrlens{all}_range={0.1154927,0.2693605},length_median=5.5680534999999995,IgrBrlens{all}=0.17839188718140966,IgrBrlens{all}_95%_HPD={0.1340457,0.2214999},height=6.667641146676637]:5.5402914999999915,((25[&length_range={5.299822,18.153075},height_95%_HPD={9.999999974752427E-7,1.8999999994662176E-5},length_95%_HPD={6.430191,12.01814},IgrBrlens{all}_range={0.2682838,0.5482517},length=9.167686881684183,IgrBrlens{all}_median=0.37463855,length_median=8.997194,IgrBrlens{all}=0.3777555158670665,IgrBrlens{all}_95%_HPD={0.299174,0.4552624},height_median=1.0000000003174137E-5,height_range={0.0,3.5999999994373866E-5},height=1.0145177411713057E-5]:8.643280999999998,20[&length_range={5.299822,17.733778},height_95%_HPD={9.999999903698154E-7,1.8999999987556748E-5},length_95%_HPD={6.512458,11.651037},IgrBrlens{all}_range={0.1987695,0.4655291},length=8.952598066966543,IgrBrlens{all}_median=0.3120878,length_median=8.7893595,IgrBrlens{all}=0.3140755620939524,IgrBrlens{all}_95%_HPD={0.2505378,0.3842583},height_median=1.0000000003174137E-5,height_range={0.0,3.5999999994373866E-5},height=1.0152548726068346E-5]:8.643280999999998)[&length_range={0.017784,4.90857},length_95%_HPD={0.12246,2.444839},length=1.1972321908097039,posterior=0.731384307846077,IgrBrlens{all}_median=0.029890609999999998,height_median=8.643291000000001,height_range={5.299825000000006,17.238179000000017},height_95%_HPD={6.430201999999987,11.487409000000007},IgrBrlens{all}_range={5.843016E-9,0.07734443},length_median=1.098201,IgrBrlens{all}=0.03064081838810929,IgrBrlens{all}_95%_HPD={0.01192442,0.04999484},height=8.794297524769416]:1.2650979999999912,((23[&length_range={4.467484,14.470178},height_95%_HPD={9.999999974752427E-7,1.8999999994662176E-5},length_95%_HPD={5.276352,9.870938},IgrBrlens{all}_range={0.1493482,0.3974316},length=7.519057811594223,IgrBrlens{all}_median=0.2613391,length_median=7.4038915,IgrBrlens{all}=0.26209146043228393,IgrBrlens{all}_95%_HPD={0.2053018,0.3179915},height_median=1.0000000003174137E-5,height_range={0.0,3.699999999184911E-5},height=1.0111819090888455E-5]:7.162613999999998,(24[&length_range={2.046462,10.396806},height_95%_HPD={9.999999974752427E-7,1.8999999994662176E-5},length_95%_HPD={2.966157,6.640364},IgrBrlens{all}_range={0.1181554,0.2876932},length=4.689548060844595,IgrBrlens{all}_median=0.188483,length_median=4.616798,IgrBrlens{all}=0.18950317337581143,IgrBrlens{all}_95%_HPD={0.1467426,0.2345849},height_median=1.0000000003174137E-5,height_range={0.0,3.599999998016301E-5},height=1.0126186906985858E-5]:4.611879000000002,22[&length_range={2.046462,10.396806},height_95%_HPD={9.999999974752427E-7,1.8999999994662176E-5},length_95%_HPD={2.966157,6.640364},IgrBrlens{all}_range={0.1099678,0.2637357},length=4.689339333333351,IgrBrlens{all}_median=0.16720465,length_median=4.6181895,IgrBrlens{all}=0.16831406368065913,IgrBrlens{all}_95%_HPD={0.1293783,0.2112794},height_median=1.0000000003174137E-5,height_range={0.0,3.599999998016301E-5},height=1.0126311844515889E-5]:4.611879000000002)[&length_range={0.005626,7.392645},length_95%_HPD={0.581108,4.584679},length=2.5086889019780765,posterior=0.9916291854072964,IgrBrlens{all}_median=0.09961861,height_median=4.611889000000005,height_range={2.0464720000000085,10.396817000000013},height_95%_HPD={2.967353999999993,6.640388999999999},IgrBrlens{all}_range={2.061252E-6,0.1760079},length_median=2.442355,IgrBrlens{all}=0.09508952621457123,IgrBrlens{all}_95%_HPD={0.03077105,0.1416331},height=4.683779719541376]:2.550734999999996)[&length_range={0.004454,7.124854},length_95%_HPD={0.009294,3.261825},length=1.3662378897677994,posterior=0.48963018490754623,IgrBrlens{all}_median=0.03320869,height_median=7.162624000000001,height_range={4.4674949999999995,13.293541000000005},height_95%_HPD={5.132787999999998,9.50065699999999},IgrBrlens{all}_range={9.842161E-10,0.1454971},length_median=1.162909,IgrBrlens{all}=0.04122830625681077,IgrBrlens{all}_95%_HPD={9.842161E-10,0.1017565},height=7.270597743812205]:0.8237880000000004,21[&length_range={3.161388,19.800603},height_95%_HPD={1.4210854715202004E-14,1.8000000011397788E-5},length_95%_HPD={4.88728,11.211844},IgrBrlens{all}_range={0.2629949,1.035569},length=7.85024190967017,IgrBrlens{all}_median=0.52218935,length_median=7.6911645,IgrBrlens{all}=0.5298768578210894,IgrBrlens{all}_95%_HPD={0.3416917,0.728703},height_median=1.0000000003174137E-5,height_range={0.0,3.799999998932435E-5},height=1.0117316342363407E-5]:7.986401999999998)[&length_range={0.008071,5.605725},length_95%_HPD={0.198391,3.271111},length=1.7373338439306354,posterior=0.9294102948525738,IgrBrlens{all}_median=0.08633787,height_median=7.986412000000001,height_range={4.856642999999991,14.744226000000012},height_95%_HPD={5.74243899999999,10.763700999999998},IgrBrlens{all}_range={4.10926E-10,0.1601399},length_median=1.688888,IgrBrlens{all}=0.08204942010397064,IgrBrlens{all}_95%_HPD={0.02167347,0.1294332},height=8.11302493910475]:1.9219769999999912)[&length_range={0.197828,5.703571},length_95%_HPD={0.69304,3.849471},length=2.2290413040752277,posterior=0.9963768115942029,IgrBrlens{all}_median=0.1178236,height_median=9.908388999999993,height_range={6.647374999999997,18.153081},height_95%_HPD={7.730422000000004,12.958438999999998},IgrBrlens{all}_range={0.01728728,0.1980925},length_median=2.180169,IgrBrlens{all}=0.11868647467711622,IgrBrlens{all}_95%_HPD={0.08237221,0.1569826},height=10.085800618181803]:2.2170780000000008)[&length_range={0.047406,4.920874},length_95%_HPD={0.099664,2.390285},length=1.2496953191329412,posterior=0.9971264367816092,IgrBrlens{all}_median=0.04448127,height_median=12.125466999999993,height_range={8.508018,19.59029800000002},height_95%_HPD={9.690380999999995,15.485416999999998},IgrBrlens{all}_range={0.002387072,0.09676912},length_median=1.178921,IgrBrlens{all}=0.04519186496942748,IgrBrlens{all}_95%_HPD={0.02363176,0.06802501},height=12.314772712191436]:1.2239600000000088,((50[&length_range={1.913903,11.84769},height_95%_HPD={9.999999974752427E-7,1.8999999994662176E-5},length_95%_HPD={3.36087,8.221763},IgrBrlens{all}_range={0.1117577,0.3237624},length=5.673912790104966,IgrBrlens{all}_median=0.1864963,length_median=5.59169,IgrBrlens{all}=0.1878918477761119,IgrBrlens{all}_95%_HPD={0.1370676,0.2413801},height_median=1.0000000003174137E-5,height_range={0.0,3.700000000605996E-5},height=1.0144052974072386E-5]:5.591695499999993,48[&length_range={1.913903,11.84769},height_95%_HPD={9.999999974752427E-7,1.8999999994662176E-5},length_95%_HPD={3.36087,8.221763},IgrBrlens{all}_range={0.09987391,0.3284325},length=5.673912790104966,IgrBrlens{all}_median=0.1945137,length_median=5.59169,IgrBrlens{all}=0.19609612251499373,IgrBrlens{all}_95%_HPD={0.1403594,0.2607962},height_median=1.0000000003174137E-5,height_range={0.0,3.700000000605996E-5},height=1.0144052974072386E-5]:5.591695499999993)[&length_range={0.194189,10.159307},length_95%_HPD={1.690896,6.444308},length=4.00346525037482,posterior=1.0,IgrBrlens{all}_median=0.1118403,height_median=5.591705499999996,height_range={1.9139050000000069,11.847701999999998},height_95%_HPD={3.3608880000000028,8.221784},IgrBrlens{all}_range={0.04651228,0.2005963},length_median=3.9303695000000003,IgrBrlens{all}=0.11305891602823623,IgrBrlens{all}_95%_HPD={0.07055594,0.156232},height=5.673922934157924]:3.9421159999999986,(46[&length_range={4.457039,14.331798},height_95%_HPD={9.999999974752427E-7,1.8999999994662176E-5},length_95%_HPD={5.218254,9.562802},IgrBrlens{all}_range={0.1731195,0.3655248},length=7.217799888930559,IgrBrlens{all}_median=0.2520702,length_median=7.0839845,IgrBrlens{all}=0.25369033079710185,IgrBrlens{all}_95%_HPD={0.2007766,0.3105067},height_median=1.0000000003174137E-5,height_range={0.0,3.699999999184911E-5},height=1.0142428786110073E-5]:7.0839840000000045,((49[&length_range={1.776634,9.679865},height_95%_HPD={9.999999974752427E-7,1.8999999994662176E-5},length_95%_HPD={2.69977,6.277896},IgrBrlens{all}_range={0.08205758,0.2817689},length=4.4401921942778495,IgrBrlens{all}_median=0.1559063,length_median=4.3766915,IgrBrlens{all}=0.1574325854622688,IgrBrlens{all}_95%_HPD={0.1035638,0.2106353},height_median=1.0000000003174137E-5,height_range={0.0,3.8000000003535206E-5},height=1.0169790105358242E-5]:4.359088999999997,44[&length_range={1.776634,9.679865},height_95%_HPD={9.999999974752427E-7,1.8999999994662176E-5},length_95%_HPD={2.671649,6.214413},IgrBrlens{all}_range={0.07796615,0.3006484},length=4.430898042603686,IgrBrlens{all}_median=0.1548002,length_median=4.371938999999999,IgrBrlens{all}=0.1566667355622183,IgrBrlens{all}_95%_HPD={0.1032996,0.2087473},height_median=1.0000000003174137E-5,height_range={0.0,3.8000000003535206E-5},height=1.0169040480177167E-5]:4.359088999999997)[&length_range={0.022807,5.446948},length_95%_HPD={0.099476,2.451805},length=1.2080500851118183,posterior=0.9776361819090454,IgrBrlens{all}_median=0.027616,height_median=4.3590990000000005,height_range={1.776637000000001,9.679876000000007},height_95%_HPD={2.7025070000000113,6.214423999999994},IgrBrlens{all}_range={1.723166E-5,0.09372326},length_median=1.113713,IgrBrlens{all}=0.02898128709137513,IgrBrlens{all}_95%_HPD={0.006061818,0.05531836},height=4.416796988115019]:1.1738025000000007,((47[&length_range={1.089117,7.163573},height_95%_HPD={9.999999974752427E-7,1.8999999994662176E-5},length_95%_HPD={1.861555,4.422573},IgrBrlens{all}_range={0.05838351,0.2158418},length=3.1054705737131294,IgrBrlens{all}_median=0.11390865,length_median=3.0288294999999996,IgrBrlens{all}=0.11553152485757123,IgrBrlens{all}_95%_HPD={0.07320296,0.1581685},height_median=1.0000000003174137E-5,height_range={0.0,3.799999998932435E-5},height=1.0154172914058178E-5]:3.028822999999999,45[&length_range={1.089117,7.163573},height_95%_HPD={9.999999974752427E-7,1.8999999994662176E-5},length_95%_HPD={1.861555,4.422573},IgrBrlens{all}_range={0.06778317,0.2582016},length=3.1054705737131294,IgrBrlens{all}_median=0.14686475,length_median=3.0288294999999996,IgrBrlens{all}=0.14902327400424725,IgrBrlens{all}_95%_HPD={0.1018059,0.2021816},height_median=1.0000000003174137E-5,height_range={0.0,3.799999998932435E-5},height=1.0154172914058178E-5]:3.028822999999999)[&length_range={0.057875,5.092489},length_95%_HPD={0.130249,2.374051},length=1.2569597045227416,posterior=1.0,IgrBrlens{all}_median=0.053487885,height_median=3.0288330000000023,height_range={1.089129000000007,7.163582000000005},height_95%_HPD={1.8615679999999983,4.422575000000002},IgrBrlens{all}_range={0.01397777,0.1349343},length_median=1.1757575,IgrBrlens{all}=0.05500896612193894,IgrBrlens{all}_95%_HPD={0.02754107,0.0864752},height=3.1054807278860634]:1.2634739999999987,19[&length_range={2.015678,9.160016},height_95%_HPD={9.999999974752427E-7,1.8999999994662176E-5},length_95%_HPD={2.86114,6.045538},IgrBrlens{all}_range={0.07022194,0.2702527},length=4.362430288980514,IgrBrlens{all}_median=0.14051605,length_median=4.2922915,IgrBrlens{all}=0.14186270699775078,IgrBrlens{all}_95%_HPD={0.09976565,0.1872979},height_median=1.0000000003174137E-5,height_range={0.0,3.799999998932435E-5},height=1.0143428286292622E-5]:4.292296999999998)[&length_range={0.032388,4.4071},length_95%_HPD={0.156073,2.377497},length=1.2488057542478745,posterior=1.0,IgrBrlens{all}_median=0.044170694999999996,height_median=4.292307000000001,height_range={2.0156849999999977,9.160029000000009},height_95%_HPD={2.861150000000009,6.0455499999999915},IgrBrlens{all}_range={0.00388951,0.111848},length_median=1.1825424999999998,IgrBrlens{all}=0.04534064638118433,IgrBrlens{all}_95%_HPD={0.01763768,0.07296873},height=4.362440432408814]:1.2405945000000003)[&length_range={0.041712,5.274967},length_95%_HPD={0.324728,3.11558},length=1.5958786967766136,posterior=1.0,IgrBrlens{all}_median=0.054308880000000004,height_median=5.532901500000001,height_range={3.108438999999997,10.932908999999995},height_95%_HPD={3.9261080000000135,7.5065409999999915},IgrBrlens{all}_range={0.01216903,0.1241621},length_median=1.5086870000000001,IgrBrlens{all}=0.055450385670914475,IgrBrlens{all}_95%_HPD={0.02786865,0.08805565},height=5.621931334582716]:1.5510925000000064)[&length_range={0.192319,6.532518},length_95%_HPD={0.876423,4.221332},length=2.459578153173414,posterior=1.0,IgrBrlens{all}_median=0.090377555,height_median=7.083994000000008,height_range={4.457048,14.33179899999999},height_95%_HPD={5.218261999999996,9.562821},IgrBrlens{all}_range={0.04838813,0.165225},length_median=2.3955219999999997,IgrBrlens{all}=0.09125169990254882,IgrBrlens{all}_95%_HPD={0.06183763,0.1214109},height=7.2178100313593205]:2.449827499999987)[&length_range={0.978657,8.73946},length_95%_HPD={1.756128,6.018629},length=3.88699755659671,posterior=1.0,IgrBrlens{all}_median=0.116759,height_median=9.533821499999995,height_range={6.2925869999999975,17.863524999999996},height_95%_HPD={7.166833999999994,12.417816000000002},IgrBrlens{all}_range={0.05629359,0.1936747},length_median=3.833173,IgrBrlens{all}=0.11764576151674187,IgrBrlens{all}_95%_HPD={0.08289853,0.1539386},height=9.677388184532733]:3.8156055000000073)[&length_range={1.668769,12.38644},length_95%_HPD={3.003055,7.964409},length=5.561570962643666,posterior=1.0,IgrBrlens{all}_median=0.2359135,height_median=13.349427000000002,height_range={9.289627999999986,24.182512000000017},height_95%_HPD={10.608408000000004,16.970853000000005},IgrBrlens{all}_range={0.1097634,0.3509742},length_median=5.532163000000001,IgrBrlens{all}=0.23761053849325375,IgrBrlens{all}_95%_HPD={0.1847566,0.2963248},height=13.567482685032498]:5.493215499999998)[&length_range={1.3799,10.891188},length_95%_HPD={2.1984,7.22297},length=4.64323040267368,posterior=1.0,IgrBrlens{all}_median=0.2428629,height_median=18.8426425,height_range={13.578085999999999,32.119339},height_95%_HPD={15.235992000000003,23.642489000000005},IgrBrlens{all}_range={0.1580433,0.3844837},length_median=4.4875395000000005,IgrBrlens{all}=0.24472465987006567,IgrBrlens{all}_95%_HPD={0.1839408,0.3039598},height=19.12905364767607]:4.589112000000004)[&length_range={2.359117,15.918881},length_95%_HPD={4.153105,11.290105},length=7.821314597951006,posterior=1.0,IgrBrlens{all}_median=0.4165438,height_median=23.431754500000004,height_range={16.47010300000001,40.307567000000006},height_95%_HPD={18.799684,29.375572000000012},IgrBrlens{all}_range={0.2580881,0.6500284},length_median=7.7191455,IgrBrlens{all}=0.41970719676411866,IgrBrlens{all}_95%_HPD={0.3222843,0.5235541},height=23.772284050349903]:7.6806334999999955,8[&length_range={22.718807,56.031009},height_95%_HPD={0.0,2.3999999996249244E-5},length_95%_HPD={25.006789,39.030116},IgrBrlens{all}_range={0.9223141,1.783877},length=31.574503235882094,IgrBrlens{all}_median=1.277272,length_median=31.098271,IgrBrlens{all}=1.2855266224262847,IgrBrlens{all}_95%_HPD={1.030242,1.527504},height_median=9.000000012804321E-6,height_range={0.0,4.799999999249849E-5},height=1.0087331334713825E-5]:31.112378999999986)[&length_range={0.099358,10.262922},length_95%_HPD={1.042,6.703138},length=3.8194369472358702,posterior=0.9921289355322339,IgrBrlens{all}_median=0.1991884,height_median=31.112388,height_range={22.827609000000002,56.03102200000001},height_95%_HPD={25.050813000000005,39.02465900000001},IgrBrlens{all}_range={0.04221908,0.4566005},length_median=3.68877,IgrBrlens{all}=0.20261491320362704,IgrBrlens{all}_95%_HPD={0.09888046,0.3144147},height=31.591014378541715]:3.6291845000000045)[&length_range={8.83441,34.226347},length_95%_HPD={12.134168,23.568833},length=17.7116408645677,posterior=1.0,IgrBrlens{all}_median=0.99138675,height_median=34.741572500000004,height_range={24.570936999999997,60.550532999999994},height_95%_HPD={28.396621000000003,44.113529},IgrBrlens{all}_range={0.6030109,1.52967},length_median=17.4571735,IgrBrlens{all}=0.9983118708270873,IgrBrlens{all}_95%_HPD={0.7686091,1.238156},height=35.38297256109429]:17.36103750000001)[&length_range={0.113055,9.182394},length_95%_HPD={0.998408,6.065603},length=3.4454228949144086,posterior=0.9998750624687656,IgrBrlens{all}_median=0.1676027,height_median=52.10261000000001,height_range={40.697592,91.485593},height_95%_HPD={43.492763,64.905568},IgrBrlens{all}_range={0.06249683,0.3565051},length_median=3.337161,IgrBrlens{all}=0.16927086229163996,IgrBrlens{all}_95%_HPD={0.09859355,0.2445623},height=53.09365166974879]:3.3668674999999837,4[&length_range={44.583249,97.372208},height_95%_HPD={0.0,2.400000000335467E-5},length_95%_HPD={46.529821,69.510524},IgrBrlens{all}_range={1.090601,2.349777},length=56.539285351074604,IgrBrlens{all}_median=1.5949395,length_median=55.469463000000005,IgrBrlens{all}=1.605154721764115,IgrBrlens{all}_95%_HPD={1.290477,1.941041},height_median=9.999999996068709E-6,height_range={0.0,4.0999999995960934E-5},height=1.0325587206885555E-5]:55.4694675)[&length_range={0.015069,19.414717},length_95%_HPD={0.474959,11.223692},length=6.09078667753624,posterior=1.0,IgrBrlens{all}_median=0.22954585,height_median=55.469477499999996,height_range={44.58326600000001,97.372218},height_95%_HPD={46.529824999999995,69.51053900000002},IgrBrlens{all}_range={1.407838E-8,0.738757},length_median=5.9957075,IgrBrlens{all}=0.23385205787182134,IgrBrlens{all}_95%_HPD={1.407838E-8,0.4266531},height=56.53960585794631]:5.6540655000000015,((3[&length_range={14.20571,49.239644},height_95%_HPD={0.0,2.2999999998774E-5},length_95%_HPD={17.585023,29.842023},IgrBrlens{all}_range={0.6279098,1.375332},length=23.54222406184409,IgrBrlens{all}_median=0.9127464000000001,length_median=23.136119,IgrBrlens{all}=0.9190847619190412,IgrBrlens{all}_95%_HPD={0.7247097,1.128923},height_median=9.999999996068709E-6,height_range={0.0,4.0999999995960934E-5},height=1.0150924538179715E-5]:23.136113,2[&length_range={14.20571,49.239644},height_95%_HPD={0.0,2.2999999998774E-5},length_95%_HPD={17.585023,29.842023},IgrBrlens{all}_range={0.8278465,1.804981},length=23.54222406184409,IgrBrlens{all}_median=1.1761955,length_median=23.136119,IgrBrlens{all}=1.1842727209145454,IgrBrlens{all}_95%_HPD={0.9448662,1.446001},height_median=9.999999996068709E-6,height_range={0.0,4.0999999995960934E-5},height=1.0150924538179715E-5]:23.136113)[&length_range={1.671163,17.141371},length_95%_HPD={3.312454,10.560745},length=6.809558791604187,posterior=1.0,IgrBrlens{all}_median=0.3203372,height_median=23.136122999999998,height_range={14.205726000000006,49.23965599999999},height_95%_HPD={17.585029999999996,29.842027},IgrBrlens{all}_range={0.1643512,0.5920309},length_median=6.63619,IgrBrlens{all}=0.32338264588955584,IgrBrlens{all}_95%_HPD={0.2202247,0.4301308},height=23.54223421276856]:6.666166500000003,1[&length_range={20.389094,57.088909},height_95%_HPD={0.0,2.69999999957804E-5},length_95%_HPD={23.758693,37.996231},IgrBrlens{all}_range={0.6406204,1.357023},length=30.351782750124965,IgrBrlens{all}_median=0.92956805,length_median=29.8022775,IgrBrlens{all}=0.9365384406421777,IgrBrlens{all}_95%_HPD={0.7343009,1.151993},height_median=9.000000005698894E-6,height_range={0.0,4.5999999997548E-5},height=1.0254247876533759E-5]:29.802280499999995)[&length_range={20.532819,57.713884},length_95%_HPD={25.174825,41.045338},length=32.27859953110929,posterior=1.0,IgrBrlens{all}_median=1.2566945,height_median=29.8022895,height_range={20.389109000000005,57.08894899999999},height_95%_HPD={23.758693,37.99625699999999},IgrBrlens{all}_range={0.7061226,1.884178},length_median=31.679751500000002,IgrBrlens{all}=1.2622337537356267,IgrBrlens{all}_95%_HPD={0.9308058,1.584049},height=30.351793004372848]:31.321253499999997)[&height_95%_HPD={54.551598,75.211129},length=0.0,posterior=1.0,height_median=61.123543,height_range={54.551598,108.721853},height=62.630392535482244];

End;

**NodeCalib2 (Maximum Clade Credibility tree)**


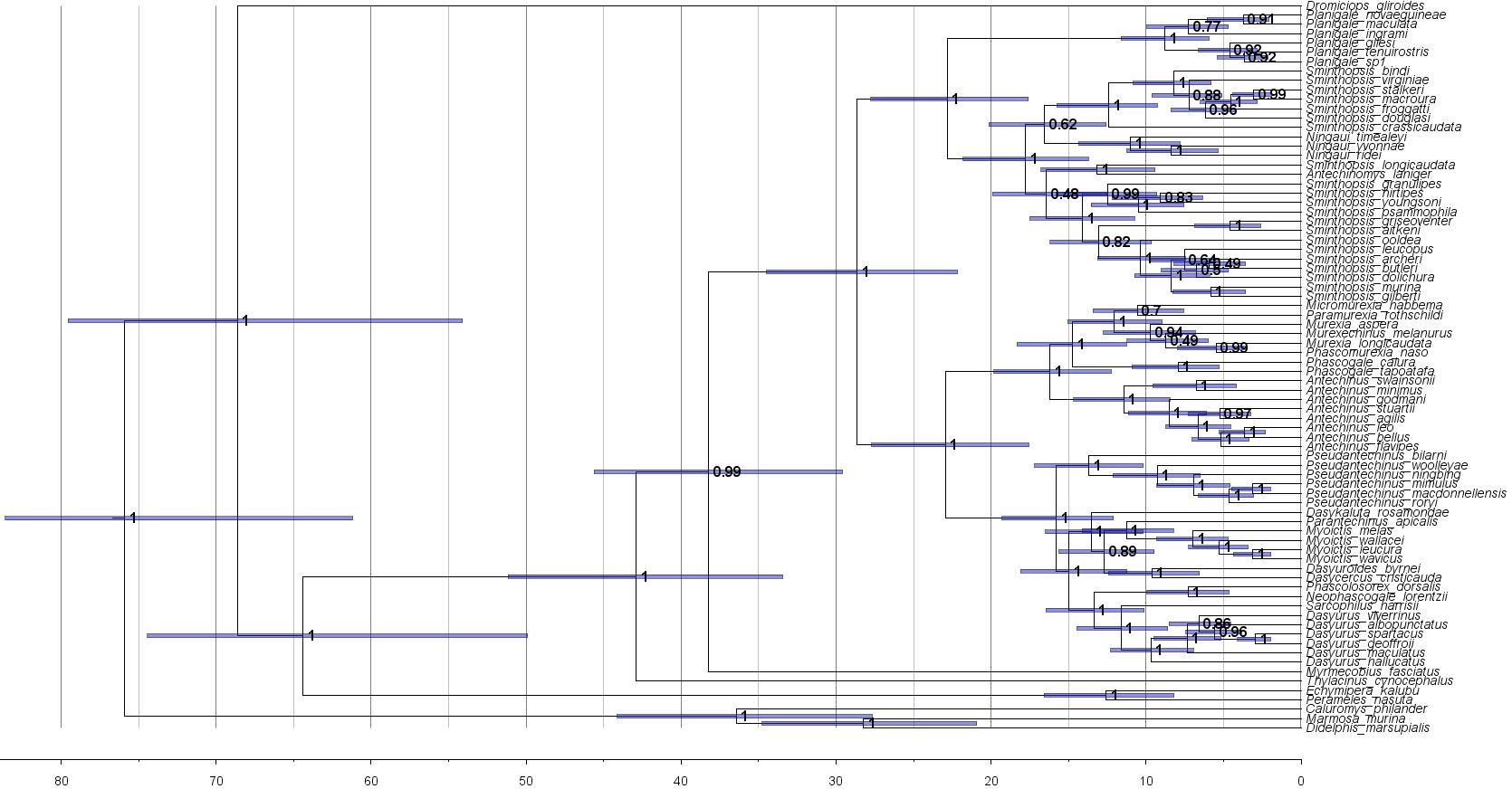


#NEXUS

Begin taxa;

Dimensions ntax=78;

Taxlabels

Caluromys_philander

Didelphis_marsupialis

Marmosa_murina

Dromiciops_gliroides

Perameles_nasuta

Echymipera_kalubu

Thylacinus_cynocephalus

Myrmecobius_fasciatus

Dasykaluta_rosamondae

Dasycercus_cristicauda

Dasyuroides_byrnei

Dasyurus_hallucatus

Myoictis_wavicus

Neophascogale_lorentzii

Phascolosorex_dorsalis

Parantechinus_apicalis

Pseudantechinus_macdonnellensis

Sarcophilus_harrisii

Antechinus_flavipes

Micromurexia_habbema

Murexia_aspera

Murexia_longicaudata

Murexechinus_melanurus

Phascomurexia_naso

Paramurexia_rothschildi

Phascogale_tapoatafa

Antechinomys_laniger

Ningaui_ridei

Sminthopsis_crassicaudata

Planigale_gilesi

Dasyurus_albopunctatus

Dasyurus_geoffroii

Dasyurus_maculatus

Dasyurus_spartacus

Dasyurus_viverrinus

Myoictis_leucura

Myoictis_melas

Myoictis_wallacei

Pseudantechinus_bilarni

Pseudantechinus_mimulus

Pseudantechinus_ningbing

Pseudantechinus_roryi

Pseudantechinus_woolleyae

Antechinus_agilis

Antechinus_bellus

Antechinus_godmani

Antechinus_leo

Antechinus_minimus

Antechinus_stuartii

Antechinus_swainsonii

Phascogale_calura

Ningaui_timealeyi

Ningaui_yvonnae

Sminthopsis_aitkeni

Sminthopsis_archeri

Sminthopsis_bindi

Sminthopsis_butleri

Sminthopsis_dolichura

Sminthopsis_douglasi

Sminthopsis_gilberti

Sminthopsis_granulipes

Sminthopsis_griseoventer

Sminthopsis_hirtipes

Sminthopsis_leucopus

Sminthopsis_longicaudata

Sminthopsis_murina

Sminthopsis_macroura

Sminthopsis_psammophila

Sminthopsis_ooldea

Sminthopsis_virginiae

Sminthopsis_youngsoni

Sminthopsis_froggatti

Sminthopsis_stalkeri

Planigale_ingrami

Planigale_maculata

Planigale_novaeguineae

Planigale_tenuirostris

Planigale_sp1

;

End;

Begin trees;

Translate

1 Caluromys_philander,

2 Didelphis_marsupialis,

3 Marmosa_murina,

4 Dromiciops_gliroides,

5 Perameles_nasuta,

6 Echymipera_kalubu,

7 Thylacinus_cynocephalus,

8 Myrmecobius_fasciatus,

9 Dasykaluta_rosamondae,

10 Dasycercus_cristicauda,

11 Dasyuroides_byrnei,

12 Dasyurus_hallucatus,

13 Myoictis_wavicus,

14 Neophascogale_lorentzii,

15 Phascolosorex_dorsalis,

16 Parantechinus_apicalis,

17 Pseudantechinus_macdonnellensis,

18 Sarcophilus_harrisii,

19 Antechinus_flavipes,

20 Micromurexia_habbema,

21 Murexia_aspera,

22 Murexia_longicaudata,

23 Murexechinus_melanurus,

24 Phascomurexia_naso,

25 Paramurexia_rothschildi,

26 Phascogale_tapoatafa,

27 Antechinomys_laniger,

28 Ningaui_ridei,

29 Sminthopsis_crassicaudata,

30 Planigale_gilesi,

31 Dasyurus_albopunctatus,

32 Dasyurus_geoffroii,

33 Dasyurus_maculatus,

34 Dasyurus_spartacus,

35 Dasyurus_viverrinus,

36 Myoictis_leucura,

37 Myoictis_melas,

38 Myoictis_wallacei,

39 Pseudantechinus_bilarni,

40 Pseudantechinus_mimulus,

41 Pseudantechinus_ningbing,

42 Pseudantechinus_roryi,

43 Pseudantechinus_woolleyae,

44 Antechinus_agilis,

45 Antechinus_bellus,

46 Antechinus_godmani,

47 Antechinus_leo,

48 Antechinus_minimus,

49 Antechinus_stuartii,

50 Antechinus_swainsonii,

51 Phascogale_calura,

52 Ningaui_timealeyi,

53 Ningaui_yvonnae,

54 Sminthopsis_aitkeni,

55 Sminthopsis_archeri,

56 Sminthopsis_bindi,

57 Sminthopsis_butleri,

58 Sminthopsis_dolichura,

59 Sminthopsis_douglasi,

60 Sminthopsis_gilberti,

61 Sminthopsis_granulipes,

62 Sminthopsis_griseoventer,

63 Sminthopsis_hirtipes,

64 Sminthopsis_leucopus,

65 Sminthopsis_longicaudata,

66 Sminthopsis_murina,

67 Sminthopsis_macroura,

68 Sminthopsis_psammophila,

69 Sminthopsis_ooldea,

70 Sminthopsis_virginiae,

71 Sminthopsis_youngsoni,

72 Sminthopsis_froggatti,

73 Sminthopsis_stalkeri,

74 Planigale_ingrami,

75 Planigale_maculata,

76 Planigale_novaeguineae,

77 Planigale_tenuirostris,

78 Planigale_sp1

;

tree TREE1 = [&R] ((4[&length_range={44.627388,82.909808},height_95%_HPD={0.0,2.8999999983625457E-5},length_95%_HPD={54.100398,79.563717},IgrBrlens{all}_range={1.091024,2.444558},length=67.74701173200894,IgrBrlens{all}_median=1.6309535,length_median=68.637444,IgrBrlens{all}=1.6474628263368327,IgrBrlens{all}_95%_HPD={1.319228,1.978799},height_median=1.100000000064938E-5,height_range={0.0,4.800000000670934E-5},height=1.19371564223734E-5]:68.642339,((((((((76[&length_range={1.552144,11.123138},height_95%_HPD={9.999999974752427E-7,2.200000000129876E-5},length_95%_HPD={1.943666,6.024887},IgrBrlens{all}_range={0.008418064,0.2902278},length=3.870195917541213,IgrBrlens{all}_median=0.089641935,length_median=3.6747715000000003,IgrBrlens{all}=0.09489132033408287,IgrBrlens{all}_95%_HPD={0.02970757,0.1699924},height_median=1.1999999998124622E-5,height_range={0.0,4.100000001017179E-5},height=1.1931159420935452E-5]:3.7149980000000085,75[&length_range={1.552144,11.421072},height_95%_HPD={9.999999974752427E-7,2.200000000129876E-5},length_95%_HPD={2.040966,7.472242},IgrBrlens{all}_range={0.02791683,0.324118},length=4.189071470764601,IgrBrlens{all}_median=0.1226304,length_median=3.8530715,IgrBrlens{all}=0.12946001096451742,IgrBrlens{all}_95%_HPD={0.05805622,0.2194792},height_median=1.1999999998124622E-5,height_range={0.0,4.100000001017179E-5},height=1.191841579277018E-5]:3.7149980000000085)[&length_range={0.097903,8.822898},length_95%_HPD={0.909254,6.280739},length=3.5595436372992983,posterior=0.9104197901049476,IgrBrlens{all}_median=0.09476884,height_median=3.7150100000000066,height_range={1.5521530000000112,8.991656000000006},height_95%_HPD={2.0692649999999873,6.073577000000014},IgrBrlens{all}_range={5.093033E-8,0.311312},length_median=3.459604,IgrBrlens{all}=0.09622381277208496,IgrBrlens{all}_95%_HPD={0.03164043,0.1660023},height=3.888582039385206]:3.533861999999992,74[&length_range={3.392029,13.985044},height_95%_HPD={9.999999974752427E-7,2.200000000129876E-5},length_95%_HPD={4.683379,10.383552},IgrBrlens{all}_range={0.11332,0.3757342},length=7.458551589205391,IgrBrlens{all}_median=0.20402195,length_median=7.38073,IgrBrlens{all}=0.20547917511244435,IgrBrlens{all}_95%_HPD={0.1517162,0.2609114},height_median=1.1999999998124622E-5,height_range={0.0,3.8999999986799594E-5},height=1.1917416292465126E-5]:7.2488600000000005)[&length_range={0.003776,7.503328},length_95%_HPD={0.0214,3.270255},length=1.549848437773811,posterior=0.7699900049975013,IgrBrlens{all}_median=0.02698468,height_median=7.248871999999999,height_range={3.5019689999999883,12.766092},height_95%_HPD={4.683386999999996,9.947022000000004},IgrBrlens{all}_range={8.624478E-10,0.1037459},length_median=1.418435,IgrBrlens{all}=0.028296562270598773,IgrBrlens{all}_95%_HPD={8.624478E-10,0.05657358},height=7.296024591594983]:1.5395265000000151,(30[&length_range={1.711667,9.271614},height_95%_HPD={1.9999999949504854E-6,2.2999999998774E-5},length_95%_HPD={2.85799,6.673462},IgrBrlens{all}_range={0.03987301,0.1960265},length=4.7033934091703795,IgrBrlens{all}_median=0.099717135,length_median=4.6220755,IgrBrlens{all}=0.1011510787293849,IgrBrlens{all}_95%_HPD={0.05831323,0.1447995},height_median=1.1999999998124622E-5,height_range={0.0,3.900000000101045E-5},height=1.1970139930612347E-5]:4.605717000000013,(77[&length_range={1.390182,7.531187},height_95%_HPD={9.999999974752427E-7,2.200000000129876E-5},length_95%_HPD={2.121141,5.406773},IgrBrlens{all}_range={0.04458673,0.4073776},length=3.727174077836092,IgrBrlens{all}_median=0.171863,length_median=3.628234,IgrBrlens{all}=0.17589360805972,IgrBrlens{all}_95%_HPD={0.09805998,0.2642217},height_median=1.1999999998124622E-5,height_range={0.0,4.0000000012696546E-5},height=1.1984382809137107E-5]:3.679112999999994,78[&length_range={1.390182,7.877916},height_95%_HPD={9.999999974752427E-7,2.200000000129876E-5},length_95%_HPD={2.217817,5.550469},IgrBrlens{all}_range={0.05238758,0.3070743},length=3.8067597794852652,IgrBrlens{all}_median=0.13864735,length_median=3.711936,IgrBrlens{all}=0.14168095956646615,IgrBrlens{all}_95%_HPD={0.08304982,0.2065176},height_median=1.1999999998124622E-5,height_range={0.0,4.0000000012696546E-5},height=1.1990254873104501E-5]:3.679112999999994)[&length_range={0.004682,3.888549},length_95%_HPD={0.028204,1.990541},length=0.9131144657552812,posterior=0.9230384807596201,IgrBrlens{all}_median=0.030673425,height_median=3.679124999999992,height_range={1.390190000000004,7.531189000000026},height_95%_HPD={2.1211510000000047,5.408484999999999},IgrBrlens{all}_range={1.183001E-9,0.1187998},length_median=0.816397,IgrBrlens{all}=0.032999701196696325,IgrBrlens{all}_95%_HPD={0.005466913,0.06378765},height=3.770694682593408]:0.9266040000000189)[&length_range={0.025299,10.198883},length_95%_HPD={1.702559,6.511929},length=4.011858864282815,posterior=0.9224137931034483,IgrBrlens{all}_median=0.1594795,height_median=4.605729000000011,height_range={1.8723000000000027,9.271620999999996},height_95%_HPD={2.8310229999999947,6.609078000000011},IgrBrlens{all}_range={2.622676E-9,0.2847746},length_median=3.982731,IgrBrlens{all}=0.15998224607838712,IgrBrlens{all}_95%_HPD={0.1132892,0.2137743},height=4.685217845455773]:4.182669500000003)[&length_range={6.948159,22.482301},length_95%_HPD={9.977072,17.996605},length=13.954832559345286,posterior=1.0,IgrBrlens{all}_median=0.43658545000000004,height_median=8.788398500000014,height_range={4.8695220000000035,15.083327000000011},height_95%_HPD={5.944198,11.619415000000004},IgrBrlens{all}_range={0.2987117,0.6603004},length_median=13.9177045,IgrBrlens{all}=0.44085142200150024,IgrBrlens{all}_95%_HPD={0.3556008,0.5312575},height=8.830067192528757]:14.048586999999994,((((56[&length_range={3.267612,13.894468},height_95%_HPD={9.999999974752427E-7,2.2000000015509613E-5},length_95%_HPD={5.486607,10.837884},IgrBrlens{all}_range={0.1550017,0.4562169},length=8.162053143928008,IgrBrlens{all}_median=0.26219329999999996,length_median=8.089628000000001,IgrBrlens{all}=0.26508754230384735,IgrBrlens{all}_95%_HPD={0.1868817,0.3445545},height_median=1.1999999998124622E-5,height_range={0.0,4.2000000021857886E-5},height=1.1904672664401055E-5]:8.187108500000008,(70[&length_range={3.193976,12.796182},height_95%_HPD={9.99999983264388E-7,2.200000000129876E-5},length_95%_HPD={4.971852,9.654005},IgrBrlens{all}_range={0.1385107,0.3995057},length=7.288695914792594,IgrBrlens{all}_median=0.23416535,length_median=7.2228025,IgrBrlens{all}=0.23602906181909042,IgrBrlens{all}_95%_HPD={0.1645852,0.3052452},height_median=1.1999999998124622E-5,height_range={0.0,4.300000001933313E-5},height=1.189155422366877E-5]:7.2256009999999975,(((73[&length_range={1.313601,6.732549},height_95%_HPD={9.999999974752427E-7,2.2000000015509613E-5},length_95%_HPD={1.925738,4.451682},IgrBrlens{all}_range={0.03152872,0.3388787},length=3.123162926411801,IgrBrlens{all}_median=0.11625225,length_median=3.0462925,IgrBrlens{all}=0.12041645394427775,IgrBrlens{all}_95%_HPD={0.05127457,0.1932012},height_median=1.1999999998124622E-5,height_range={0.0,4.100000001017179E-5},height=1.1931159421084593E-5]:3.0411140000000074,67[&length_range={1.313601,6.931242},height_95%_HPD={9.999999974752427E-7,2.2000000015509613E-5},length_95%_HPD={1.87036,4.405302},IgrBrlens{all}_range={0.003747885,0.1481575},length=3.1246478941779174,IgrBrlens{all}_median=0.04918164999999999,length_median=3.047234,IgrBrlens{all}=0.05115475370527257,IgrBrlens{all}_95%_HPD={0.0170368,0.09155822},height_median=1.1999999998124622E-5,height_range={0.0,4.100000001017179E-5},height=1.1930784608505151E-5]:3.0411140000000074)[&length_range={0.014459,5.621821},length_95%_HPD={0.162513,2.982877},length=1.4932211118695495,posterior=0.9883808095952024,IgrBrlens{all}_median=0.03947664,height_median=3.0411260000000055,height_range={1.3136120000000062,6.732570999999979},height_95%_HPD={1.9047020000000074,4.405304000000001},IgrBrlens{all}_range={6.270995E-5,0.1563604},length_median=1.397647,IgrBrlens{all}=0.041939445837738475,IgrBrlens{all}_95%_HPD={0.005929594,0.08302524},height=3.115524208317532]:1.5042959999999894,72[&length_range={1.959264,9.491162},height_95%_HPD={5.6843418860808015E-14,2.2000000029720468E-5},length_95%_HPD={2.790826,6.490041},IgrBrlens{all}_range={0.05190014,0.3960609},length=4.595903685282357,IgrBrlens{all}_median=0.16613955,length_median=4.542127,IgrBrlens{all}=0.17034706690404774,IgrBrlens{all}_95%_HPD={0.08683539,0.2667145},height_median=1.1999999991019195E-5,height_range={0.0,3.90000000152213E-5},height=1.193565717220389E-5]:4.545410000000004)[&length_range={0.009697,7.53194},length_95%_HPD={0.205731,3.38522},length=1.7252673171189254,posterior=0.9991254372813593,IgrBrlens{all}_median=0.05303969,height_median=4.545421999999995,height_range={1.9592779999999834,9.491176999999979},height_95%_HPD={2.8168070000000114,6.488883000000001},IgrBrlens{all}_range={0.002818869,0.174786},length_median=1.610848,IgrBrlens{all}=0.054928631113542584,IgrBrlens{all}_95%_HPD={0.01269698,0.0980497},height=4.601451422408371]:1.6444860000000077,59[&length_range={3.193976,11.122335},height_95%_HPD={9.999999974752427E-7,2.2000000015509613E-5},length_95%_HPD={4.236689,8.396411},IgrBrlens{all}_range={0.1258842,0.3795759},length=6.238023721264376,IgrBrlens{all}_median=0.20545635,length_median=6.176544,IgrBrlens{all}=0.2075842835082457,IgrBrlens{all}_95%_HPD={0.1511284,0.2684208},height_median=1.1999999991019195E-5,height_range={0.0,4.0000000012696546E-5},height=1.1899175412979366E-5]:6.189896000000012)[&length_range={0.014262,5.133928},length_95%_HPD={0.104497,2.289369},length=1.104039863179859,posterior=0.957896051974013,IgrBrlens{all}_median=0.02804293,height_median=6.189908000000003,height_range={3.289603999999997,11.122362999999979},height_95%_HPD={4.253069999999994,8.391295},IgrBrlens{all}_range={0.001621398,0.0904323},length_median=1.011675,IgrBrlens{all}=0.029439170656840936,IgrBrlens{all}_95%_HPD={0.008795614,0.05285049},height=6.2513454351115145]:1.035704999999993)[&length_range={0.008091,4.729737},length_95%_HPD={0.040662,2.200504},length=0.9990010139791028,posterior=0.8848075962018991,IgrBrlens{all}_median=0.022284844999999998,height_median=7.225612999999996,height_range={3.927847,12.796197000000006},height_95%_HPD={5.083250999999997,9.62287599999999},IgrBrlens{all}_range={2.403185E-8,0.09157725},length_median=0.8884575,IgrBrlens{all}=0.023775869493247385,IgrBrlens{all}_95%_HPD={2.403185E-8,0.04764702},height=7.301282824343411]:0.9615075000000104)[&length_range={0.711318,10.153257},length_95%_HPD={1.804947,6.566438},length=4.178457371439291,posterior=1.0,IgrBrlens{all}_median=0.15527705000000003,height_median=8.187120500000006,height_range={4.359967999999995,13.894478999999997},height_95%_HPD={5.797021999999998,10.844791999999998},IgrBrlens{all}_range={0.07639741,0.3082334},length_median=4.1122125,IgrBrlens{all}=0.15693569997751075,IgrBrlens{all}_95%_HPD={0.1046984,0.2073719},height=8.281151424037981]:4.2095754999999855,29[&length_range={6.881958,19.968545},height_95%_HPD={9.999999974752427E-7,2.200000000129876E-5},length_95%_HPD={9.271879,15.741749},IgrBrlens{all}_range={0.2774075,0.6250044},length=12.45959686994002,IgrBrlens{all}_median=0.39726265,length_median=12.396677,IgrBrlens{all}=0.39982021690405,IgrBrlens{all}_95%_HPD={0.321248,0.4826635},height_median=1.1999999998124622E-5,height_range={0.0,4.200000000764703E-5},height=1.192553723201303E-5]:12.396683999999993)[&length_range={1.20271,10.792169},length_95%_HPD={2.02285,7.184405},length=4.502587919165403,posterior=1.0,IgrBrlens{all}_median=0.1608351,height_median=12.396695999999991,height_range={6.8819680000000005,19.968561000000008},height_95%_HPD={9.271889000000009,15.741764000000003},IgrBrlens{all}_range={0.09214191,0.2815891},length_median=4.407096,IgrBrlens{all}=0.1626321316229387,IgrBrlens{all}_95%_HPD={0.1222174,0.2056908},height=12.459608795477244]:4.141557000000006,(52[&length_range={5.794978,17.752663},height_95%_HPD={9.999999974752427E-7,2.200000000129876E-5},length_95%_HPD={7.803299,14.334318},IgrBrlens{all}_range={0.2012682,0.6209592},length=11.016921544102946,IgrBrlens{all}_median=0.3462422,length_median=11.000421,IgrBrlens{all}=0.3496144311094447,IgrBrlens{all}_95%_HPD={0.262428,0.4357551},height_median=1.1999999998124622E-5,height_range={0.0,4.100000001017179E-5},height=1.1920664668356017E-5]:11.000418500000002,(53[&length_range={3.730371,14.719802},height_95%_HPD={9.99999983264388E-7,2.200000000129876E-5},length_95%_HPD={5.344761,11.245841},IgrBrlens{all}_range={0.2019605,0.5630623},length=8.391022240629672,IgrBrlens{all}_median=0.33335064999999997,length_median=8.355285,IgrBrlens{all}=0.33688677012743623,IgrBrlens{all}_95%_HPD={0.2523997,0.4301483},height_median=1.1999999998124622E-5,height_range={0.0,4.200000000764703E-5},height=1.1921664168514594E-5]:8.355283500000006,28[&length_range={3.730371,14.719802},height_95%_HPD={9.99999983264388E-7,2.200000000129876E-5},length_95%_HPD={5.344761,11.245841},IgrBrlens{all}_range={0.1447928,0.3966076},length=8.391022240629672,IgrBrlens{all}_median=0.24925175,length_median=8.355285,IgrBrlens{all}=0.25156258483258354,IgrBrlens{all}_95%_HPD={0.1893464,0.3209348},height_median=1.1999999998124622E-5,height_range={0.0,4.200000000764703E-5},height=1.1921664168514594E-5]:8.355283500000006)[&length_range={0.068666,7.224111},length_95%_HPD={0.667776,4.781306},length=2.6258993024737616,posterior=1.0,IgrBrlens{all}_median=0.08083483999999999,height_median=8.355295500000004,height_range={3.7303739999999976,14.719814999999997},height_95%_HPD={5.344778000000005,11.24585500000002},IgrBrlens{all}_range={0.02544052,0.1914996},length_median=2.5127610000000002,IgrBrlens{all}=0.08213169113193423,IgrBrlens{all}_95%_HPD={0.04346581,0.1214848},height=8.391034162293852]:2.6451349999999962)[&length_range={1.078071,10.684764},length_95%_HPD={2.536029,8.186387},length=5.347673818590697,posterior=1.0,IgrBrlens{all}_median=0.17481405,height_median=11.0004305,height_range={5.794986999999985,17.752685999999983},height_95%_HPD={7.803305000000002,14.334330999999992},IgrBrlens{all}_range={0.09745995,0.3115582},length_median=5.2872695,IgrBrlens{all}=0.1763152014330332,IgrBrlens{all}_95%_HPD={0.1251006,0.2305291},height=11.0169334647676]:5.537822499999997)[&length_range={0.013509,3.586871},length_95%_HPD={0.034708,1.83981},length=0.8274901468857769,posterior=0.6158170914542729,IgrBrlens{all}_median=0.01597012,height_median=16.538252999999997,height_range={10.288920000000005,23.134559000000003},height_95%_HPD={12.567715000000007,20.152910000000013},IgrBrlens{all}_range={3.408851E-4,0.05905879},length_median=0.731648,IgrBrlens{all}=0.01673148387869752,IgrBrlens{all}_95%_HPD={0.005704745,0.03058585},height=16.472480983566655]:1.2386399999999966,((65[&length_range={6.907161,20.258113},height_95%_HPD={1.0000000116860974E-6,2.200000000129876E-5},length_95%_HPD={9.412499,16.775363},IgrBrlens{all}_range={0.2748866,0.7068394},length=13.128133797351298,IgrBrlens{all}_median=0.44919605,length_median=13.1513315,IgrBrlens{all}=0.4544032421289357,IgrBrlens{all}_95%_HPD={0.3434676,0.5700468},height_median=1.1999999998124622E-5,height_range={0.0,3.8000000003535206E-5},height=1.1921789106063268E-5]:13.151331500000001,27[&length_range={6.907161,20.258113},height_95%_HPD={1.0000000116860974E-6,2.200000000129876E-5},length_95%_HPD={9.412499,16.775363},IgrBrlens{all}_range={0.2281251,0.540616},length=13.128133797351298,IgrBrlens{all}_median=0.34585785,length_median=13.1513315,IgrBrlens{all}=0.34884231772863605,IgrBrlens{all}_95%_HPD={0.2756883,0.4245122},height_median=1.1999999998124622E-5,height_range={0.0,3.8000000003535206E-5},height=1.1921789106063268E-5]:13.151331500000001)[&length_range={0.355443,9.567817},length_95%_HPD={1.030999,5.892675},length=3.4182307717391316,posterior=1.0,IgrBrlens{all}_median=0.08041586,height_median=13.1513435,height_range={6.907173,20.258117000000006},height_95%_HPD={9.412511999999992,16.775378999999994},IgrBrlens{all}_range={0.03249327,0.1638714},length_median=3.2860709999999997,IgrBrlens{all}=0.08184879043728124,IgrBrlens{all}_95%_HPD={0.04704317,0.1196539},height=13.128145719140429]:3.277958999999999,((61[&length_range={7.381666,18.462127},height_95%_HPD={9.999999974752427E-7,2.200000000129876E-5},length_95%_HPD={9.239597,15.747475},IgrBrlens{all}_range={0.2540788,0.6633361},length=12.509315737381304,IgrBrlens{all}_median=0.41035505,length_median=12.4875985,IgrBrlens{all}=0.41463725693403164,IgrBrlens{all}_95%_HPD={0.3103163,0.5199835},height_median=1.1999999998124622E-5,height_range={0.0,4.0000000012696546E-5},height=1.1909795103038235E-5]:12.47787300000001,((63[&length_range={3.588091,15.404849},height_95%_HPD={9.999999974752427E-7,2.200000000129876E-5},length_95%_HPD={6.350718,12.347912},IgrBrlens{all}_range={0.1835371,0.5722421},length=9.257735981384304,IgrBrlens{all}_median=0.3147429,length_median=9.218729,IgrBrlens{all}=0.3182190536856585,IgrBrlens{all}_95%_HPD={0.2287499,0.4098274},height_median=1.1999999998124622E-5,height_range={0.0,3.8000000003535206E-5},height=1.193703148488243E-5]:9.067689999999999,71[&length_range={3.588091,14.711293},height_95%_HPD={9.999999974752427E-7,2.200000000129876E-5},length_95%_HPD={6.333292,12.307771},IgrBrlens{all}_range={0.1956145,0.6317215},length=9.227967970639678,IgrBrlens{all}_median=0.3572728,length_median=9.190196499999999,IgrBrlens{all}=0.36222484838830726,IgrBrlens{all}_95%_HPD={0.2667137,0.4661893},height_median=1.1999999998124622E-5,height_range={0.0,3.8000000003535206E-5},height=1.1930784608327605E-5]:9.067689999999999)[&length_range={0.021512,5.535481},length_95%_HPD={0.125342,2.970244},length=1.4235170483460586,posterior=0.8347076461769115,IgrBrlens{all}_median=0.03827406,height_median=9.067701999999997,height_range={3.5880949999999814,14.441029},height_95%_HPD={6.350722000000005,12.178100999999998},IgrBrlens{all}_range={1.173866E-4,0.1343831},length_median=1.294309,IgrBrlens{all}=0.04030987339664714,IgrBrlens{all}_95%_HPD={0.006641,0.07578635},height=9.098283930848673]:1.4268814999999968,68[&length_range={4.82493,16.087532},height_95%_HPD={9.999999974752427E-7,2.200000000129876E-5},length_95%_HPD={7.214457,13.33581},IgrBrlens{all}_range={0.1959434,0.4841309},length=10.348974988130907,IgrBrlens{all}_median=0.30812645,length_median=10.322500999999999,IgrBrlens{all}=0.31086590665917,IgrBrlens{all}_95%_HPD={0.237134,0.3871554},height_median=1.1999999998124622E-5,height_range={0.0,3.900000000101045E-5},height=1.1921664168627337E-5]:10.494571499999996)[&length_range={0.028334,7.15614},length_95%_HPD={0.305616,3.809363},length=1.9935217011494235,posterior=1.0,IgrBrlens{all}_median=0.05491965,height_median=10.494583499999994,height_range={5.670076999999992,16.087542000000006},height_95%_HPD={7.551863000000004,13.551260999999997},IgrBrlens{all}_range={0.008627871,0.1458532},length_median=1.8835465,IgrBrlens{all}=0.056643281109569975,IgrBrlens{all}_95%_HPD={0.02457803,0.09366311},height=10.513182039480222]:1.9833015000000138)[&length_range={0.036994,6.037029},length_95%_HPD={0.173276,2.926244},length=1.4345495474295815,posterior=0.9891304347826086,IgrBrlens{all}_median=0.03404097,height_median=12.477885000000008,height_range={7.381672999999999,18.462135000000004},height_95%_HPD={9.294047000000006,15.789395999999996},IgrBrlens{all}_range={8.439178E-7,0.122917},length_median=1.320118,IgrBrlens{all}=0.03538224182556651,IgrBrlens{all}_95%_HPD={0.009203892,0.06339918},height=12.499373269925519]:1.6127499999999912,((62[&length_range={2.003701,9.863526},height_95%_HPD={9.999999974752427E-7,2.200000000129876E-5},length_95%_HPD={2.611469,6.858789},IgrBrlens{all}_range={0.06024092,0.2506255},length=4.644833011244385,IgrBrlens{all}_median=0.12437085,length_median=4.568545,IgrBrlens{all}=0.1267459044252872,IgrBrlens{all}_95%_HPD={0.08435145,0.1762101},height_median=1.1999999998124622E-5,height_range={0.0,3.999999999848569E-5},height=1.1924412794068753E-5]:4.568547000000013,54[&length_range={2.003701,9.863526},height_95%_HPD={9.999999974752427E-7,2.200000000129876E-5},length_95%_HPD={2.611469,6.858789},IgrBrlens{all}_range={0.06098283,0.2590177},length=4.644833011244385,IgrBrlens{all}_median=0.12700774999999997,length_median=4.568545,IgrBrlens{all}=0.12888643050099954,IgrBrlens{all}_95%_HPD={0.08134695,0.1758113},height_median=1.1999999998124622E-5,height_range={0.0,3.999999999848569E-5},height=1.1924412794068753E-5]:4.568547000000013)[&length_range={3.533317,14.11686},length_95%_HPD={5.444561,11.544284},length=8.443045214018031,posterior=1.0,IgrBrlens{all}_median=0.21251114999999998,height_median=4.568559000000011,height_range={2.0037049999999965,9.863542999999993},height_95%_HPD={2.6114710000000088,6.858807999999996},IgrBrlens{all}_range={0.1152503,0.379981},length_median=8.409621,IgrBrlens{all}=0.21531624558970539,IgrBrlens{all}_95%_HPD={0.1525996,0.2857743},height=4.644844935657169]:8.503408999999994,(69[&length_range={5.608109,16.352904},height_95%_HPD={9.999999974752427E-7,2.200000000129876E-5},length_95%_HPD={7.468701,13.124094},IgrBrlens{all}_range={0.218435,0.5891571},length=10.412628127686155,IgrBrlens{all}_median=0.34230815000000003,length_median=10.36875,IgrBrlens{all}=0.34601465462268866,IgrBrlens{all}_95%_HPD={0.2576465,0.4432095},height_median=1.1999999998124622E-5,height_range={0.0,4.100000001017179E-5},height=1.1919165417930837E-5]:10.368744000000003,((64[&length_range={2.858112,12.49572},height_95%_HPD={9.999999974752427E-7,2.200000000129876E-5},length_95%_HPD={4.306416,9.334961},IgrBrlens{all}_range={0.1456787,0.4570258},length=6.901915187031485,IgrBrlens{all}_median=0.25443075,length_median=6.883429,IgrBrlens{all}=0.2571701891179414,IgrBrlens{all}_95%_HPD={0.1876322,0.3340837},height_median=1.1999999998124622E-5,height_range={0.0,4.200000000764703E-5},height=1.1923538231550286E-5]:7.507521500000003,((55[&length_range={2.637956,13.279132},height_95%_HPD={9.999999974752427E-7,2.200000000129876E-5},length_95%_HPD={3.940007,10.14804},IgrBrlens{all}_range={0.1402358,0.4471237},length=6.918633037106423,IgrBrlens{all}_median=0.2460039,length_median=6.823315,IgrBrlens{all}=0.249178108145927,IgrBrlens{all}_95%_HPD={0.17873,0.3294875},height_median=1.1999999998124622E-5,height_range={0.0,3.90000000152213E-5},height=1.1905547227033153E-5]:5.941124500000001,57[&length_range={2.637956,12.268557},height_95%_HPD={9.999999974752427E-7,2.200000000129876E-5},length_95%_HPD={3.853199,8.763713},IgrBrlens{all}_range={0.1768336,0.56084},length=6.387729156421756,IgrBrlens{all}_median=0.29534214999999997,length_median=6.377383999999999,IgrBrlens{all}=0.29823323704397836,IgrBrlens{all}_95%_HPD={0.2234547,0.3801478},height_median=1.1999999998124622E-5,height_range={0.0,3.90000000152213E-5},height=1.192478760679467E-5]:5.941124500000001)[&length_range={0.006001,4.553216},length_95%_HPD={0.054488,2.103432},length=0.9592878093285497,posterior=0.48750624687656174,IgrBrlens{all}_median=0.02748143,height_median=5.941136499999999,height_range={2.63797000000001,10.818082000000004},height_95%_HPD={3.581975,8.187932000000004},IgrBrlens{all}_range={2.025736E-7,0.105281},length_median=0.846758,IgrBrlens{all}=0.029210684199772907,IgrBrlens{all}_95%_HPD={2.025736E-7,0.05838509},height=5.981235980522805]:0.7977864999999724,58[&length_range={2.858112,11.389709},height_95%_HPD={9.999999974752427E-7,2.200000000129876E-5},length_95%_HPD={4.244922,8.730973},IgrBrlens{all}_range={0.1149568,0.3529134},length=6.449178694277857,IgrBrlens{all}_median=0.2074192,length_median=6.4170045,IgrBrlens{all}=0.20923190296101962,IgrBrlens{all}_95%_HPD={0.1541676,0.2674723},height_median=1.1999999998124622E-5,height_range={0.0,3.90000000152213E-5},height=1.1919165417877572E-5]:6.738910999999973)[&length_range={0.011777,3.486672},length_95%_HPD={0.032623,1.68221},length=0.7617240044720511,posterior=0.5028735632183908,IgrBrlens{all}_median=0.01591192,height_median=6.738922999999971,height_range={3.5311559999999957,11.291967},height_95%_HPD={4.6789090000000115,9.013062000000005},IgrBrlens{all}_range={2.038227E-5,0.06727957},length_median=0.676302,IgrBrlens{all}=0.017533550396029857,IgrBrlens{all}_95%_HPD={0.001977126,0.03545482},height=6.753390535155265]:0.7686105000000296)[&length_range={0.02052,3.292772},length_95%_HPD={0.02052,1.804839},length=0.8268077861598411,posterior=0.6409295352323838,IgrBrlens{all}_median=0.021461135,height_median=7.507533500000001,height_range={3.916234000000003,12.846845000000016},height_95%_HPD={5.281001000000003,9.803792000000016},IgrBrlens{all}_range={6.104068E-7,0.0807499},length_median=0.7276,IgrBrlens{all}=0.02261941954351905,IgrBrlens{all}_95%_HPD={0.003324969,0.04353698},height=7.500864191423021]:0.8695085000000091,(66[&length_range={2.288732,11.011646},height_95%_HPD={9.999999974752427E-7,2.200000000129876E-5},length_95%_HPD={3.415544,8.139903},IgrBrlens{all}_range={0.1124017,0.427929},length=5.865924380934539,IgrBrlens{all}_median=0.22245104999999998,length_median=5.827286,IgrBrlens{all}=0.22510708960519712,IgrBrlens{all}_95%_HPD={0.1624772,0.2973326},height_median=1.1999999998124622E-5,height_range={0.0,4.199999999343618E-5},height=1.1928660670273575E-5]:5.82345500000001,60[&length_range={2.288732,10.820355},height_95%_HPD={9.999999974752427E-7,2.200000000129876E-5},length_95%_HPD={3.573278,8.268669},IgrBrlens{all}_range={0.05280136,0.2539361},length=5.859665644177915,IgrBrlens{all}_median=0.11799019999999999,length_median=5.824388000000001,IgrBrlens{all}=0.11949308771114453,IgrBrlens{all}_95%_HPD={0.0784714,0.1686827},height_median=1.1999999998124622E-5,height_range={0.0,4.199999999343618E-5},height=1.1927161419896334E-5]:5.82345500000001)[&length_range={0.10025,6.529872},length_95%_HPD={0.445992,4.098789},length=2.199243128680611,posterior=0.9971264367816092,IgrBrlens{all}_median=0.04223559,height_median=5.823467000000008,height_range={2.2887409999999946,10.820371999999978},height_95%_HPD={3.5696020000000033,8.266615000000002},IgrBrlens{all}_range={0.008254663,0.1435451},length_median=2.088213,IgrBrlens{all}=0.04403834593045962,IgrBrlens{all}_95%_HPD={0.01764122,0.07405143},height=5.858778117654412]:2.553575000000002)[&length_range={0.115295,5.891106},length_95%_HPD={0.482019,3.798923},length=2.0430708394552735,posterior=1.0,IgrBrlens{all}_median=0.06754655000000001,height_median=8.37704200000001,height_range={4.313542000000005,13.503613999999999},height_95%_HPD={5.89698400000001,10.748640000000002},IgrBrlens{all}_range={0.0199833,0.1731878},length_median=1.9497325,IgrBrlens{all}=0.06889384009620181,IgrBrlens{all}_95%_HPD={0.03631843,0.1045798},height=8.369569207396314]:1.991713999999991)[&length_range={0.257252,8.927457},length_95%_HPD={0.850067,4.909474},length=2.8181067351324254,posterior=1.0,IgrBrlens{all}_median=0.09640185500000001,height_median=10.368756000000001,height_range={5.608131,16.35292600000001},height_95%_HPD={7.4687100000000015,13.124102000000008},IgrBrlens{all}_range={0.04373039,0.1918005},length_median=2.7305335,IgrBrlens{all}=0.09800938503498269,IgrBrlens{all}_95%_HPD={0.05873282,0.1380761},height=10.412640046851582]:2.703212000000004)[&length_range={0.004046,4.170275},length_95%_HPD={0.03582,2.248801},length=1.022779980490779,posterior=0.8197151424287856,IgrBrlens{all}_median=0.0167602,height_median=13.071968000000005,height_range={7.131821000000002,18.670677999999988},height_95%_HPD={9.650023000000004,16.184608999999988},IgrBrlens{all}_range={1.512585E-9,0.07116231},length_median=0.908751,IgrBrlens{all}=0.018184929606859003,IgrBrlens{all}_95%_HPD={1.512585E-9,0.03657294},height=13.036518225118115]:1.0186669999999935)[&length_range={0.197083,7.626303},length_95%_HPD={0.673436,4.427988},length=2.4778137889805008,posterior=1.0,IgrBrlens{all}_median=0.069103585,height_median=14.090634999999999,height_range={8.690677,21.034413000000008},height_95%_HPD={10.745116000000003,17.491355999999996},IgrBrlens{all}_range={0.02455477,0.1323252},length_median=2.384926,IgrBrlens{all}=0.07020133893928047,IgrBrlens{all}_95%_HPD={0.04314563,0.09896169},height=14.080429425787095]:2.3386674999999997)[&length_range={0.029565,4.170865},length_95%_HPD={0.048008,2.234414},length=1.056453434251558,posterior=0.48075962018990503,IgrBrlens{all}_median=0.01784351,height_median=16.4293025,height_range={10.699044,23.04818300000001},height_95%_HPD={12.534669000000001,19.909949999999988},IgrBrlens{all}_range={3.650835E-4,0.05231208},length_median=0.948217,IgrBrlens{all}=0.018447336670374258,IgrBrlens{all}_95%_HPD={0.003347091,0.03401376},height=16.355286044178772]:1.3475904999999955)[&length_range={1.694571,10.970079},length_95%_HPD={2.568215,7.841433},length=5.06442514167916,posterior=1.0,IgrBrlens{all}_median=0.1658483,height_median=17.776892999999994,height_range={11.355872999999995,25.443713999999993},height_95%_HPD={13.72203600000001,21.794283000000007},IgrBrlens{all}_range={0.09910023,0.2799506},length_median=4.9320855,IgrBrlens{all}=0.16773226434657582,IgrBrlens{all}_95%_HPD={0.123441,0.2141569},height=17.72047461019493]:5.060092500000014)[&length_range={1.166134,12.9899},length_95%_HPD={2.736021,8.713518},length=5.679090556846589,posterior=1.0,IgrBrlens{all}_median=0.17221579999999997,height_median=22.836985500000008,height_range={14.336404000000002,32.31582300000001},height_95%_HPD={17.591468,27.760377000000005},IgrBrlens{all}_range={0.1018913,0.2842552},length_median=5.575296,IgrBrlens{all}=0.17370601512993522,IgrBrlens{all}_95%_HPD={0.1260314,0.2245653},height=22.784899751874025]:5.807486999999995,(((((20[&length_range={5.739689,16.540662},height_95%_HPD={9.999999974752427E-7,2.200000000129876E-5},length_95%_HPD={7.900938,13.829704},IgrBrlens{all}_range={0.2334491,0.5431412},length=10.77054060819592,IgrBrlens{all}_median=0.3186211,length_median=10.760202,IgrBrlens{all}=0.3215960101949036,IgrBrlens{all}_95%_HPD={0.2575812,0.393502},height_median=1.1999999998124622E-5,height_range={0.0,4.0000000012696546E-5},height=1.1930909545767974E-5]:10.561188000000005,25[&length_range={5.739689,16.838809},height_95%_HPD={1.00000000458067E-6,2.200000000129876E-5},length_95%_HPD={7.726544,14.232335},IgrBrlens{all}_range={0.2815942,0.5658032},length=11.059949631934023,IgrBrlens{all}_median=0.38171089999999996,length_median=11.019136,IgrBrlens{all}=0.3854989754497748,IgrBrlens{all}_95%_HPD={0.3080283,0.466386},height_median=1.1999999998124622E-5,height_range={0.0,4.0000000012696546E-5},height=1.194040479811515E-5]:10.561188000000005)[&length_range={0.036574,4.934097},length_95%_HPD={0.182508,2.836653},length=1.3679204511198035,posterior=0.7028985507246377,IgrBrlens{all}_median=0.03012981,height_median=10.561200000000003,height_range={5.739712000000004,16.540683},height_95%_HPD={7.569057000000001,13.42423699999999},IgrBrlens{all}_range={8.704084E-6,0.0768577},length_median=1.261829,IgrBrlens{all}=0.03097712702840451,IgrBrlens{all}_95%_HPD={0.01224878,0.05214654},height=10.60370832260933]:1.476504000000002,(21[&length_range={3.374019,18.974056},height_95%_HPD={9.999999974752427E-7,2.200000000129876E-5},length_95%_HPD={5.782208,13.176591},IgrBrlens{all}_range={0.2518274,1.218117},length=9.444363240004986,IgrBrlens{all}_median=0.53043375,length_median=9.313358000000001,IgrBrlens{all}=0.5377303522488754,IgrBrlens{all}_95%_HPD={0.3373325,0.7327667},height_median=1.1999999998124622E-5,height_range={0.0,3.90000000152213E-5},height=1.195577211464988E-5]:9.712196500000005,(23[&length_range={4.49639,15.109949},height_95%_HPD={9.999999974752427E-7,2.200000000129876E-5},length_95%_HPD={6.171469,11.706861},IgrBrlens{all}_range={0.1468456,0.3986765},length=9.031803123063488,IgrBrlens{all}_median=0.26559639999999995,length_median=9.0380575,IgrBrlens{all}=0.2677877982133936,IgrBrlens{all}_95%_HPD={0.2100542,0.3265287},height_median=1.1999999998124622E-5,height_range={0.0,4.0000000012696546E-5},height=1.1945027486897639E-5]:8.723013999999992,(22[&length_range={2.432056,10.678654},height_95%_HPD={9.999999974752427E-7,2.2000000015509613E-5},length_95%_HPD={3.452258,7.954589},IgrBrlens{all}_range={0.1086399,0.2804397},length=5.55138992953522,IgrBrlens{all}_median=0.170571,length_median=5.479323,IgrBrlens{all}=0.171915785207397,IgrBrlens{all}_95%_HPD={0.1337176,0.212909},height_median=1.1999999998124622E-5,height_range={0.0,4.0000000012696546E-5},height=1.1933158421376004E-5]:5.475589999999997,24[&length_range={2.432056,10.678654},height_95%_HPD={9.999999974752427E-7,2.2000000015509613E-5},length_95%_HPD={3.452258,7.954589},IgrBrlens{all}_range={0.1222117,0.3041434},length=5.5518733960519615,IgrBrlens{all}_median=0.1923045,length_median=5.4799845000000005,IgrBrlens{all}=0.1941482923038485,IgrBrlens{all}_95%_HPD={0.1527647,0.239898},height_median=1.1999999998124622E-5,height_range={0.0,4.0000000012696546E-5},height=1.1934157921619805E-5]:5.475589999999997)[&length_range={0.010101,8.499586},length_95%_HPD={0.792832,5.337548},length=3.1108506734616888,posterior=0.9928785607196402,IgrBrlens{all}_median=0.1023657,height_median=5.475601999999995,height_range={2.4320690000000127,10.678685000000002},height_95%_HPD={3.4554529999999986,7.954605999999998},IgrBrlens{all}_range={3.000992E-9,0.197082},length_median=3.096064,IgrBrlens{all}=0.09779209253295662,IgrBrlens{all}_95%_HPD={0.03306738,0.1457132},height=5.545369281741529]:3.247423999999995)[&length_range={9.72E-4,6.775227},length_95%_HPD={0.019154,3.834083},length=1.6374711144316731,posterior=0.4891304347826087,IgrBrlens{all}_median=0.03385541,height_median=8.72302599999999,height_range={4.496404000000005,13.791184999999999},height_95%_HPD={5.977894000000006,11.224109999999996},IgrBrlens{all}_range={5.280827E-9,0.1545316},length_median=1.424954,IgrBrlens{all}=0.04146798149625942,IgrBrlens{all}_95%_HPD={5.280827E-9,0.1043121},height=8.723289077139214]:0.9891825000000125)[&length_range={0.012273,7.241278},length_95%_HPD={0.167808,3.768484},length=2.0024922421165092,posterior=0.935032483758121,IgrBrlens{all}_median=0.08751138,height_median=9.712208500000003,height_range={4.913667000000004,18.974072999999997},height_95%_HPD={6.8297489999999925,12.759822},IgrBrlens{all}_range={2.670252E-9,0.1677338},length_median=1.947578,IgrBrlens{all}=0.08368449286120835,IgrBrlens{all}_95%_HPD={0.02472845,0.1331623},height=9.759010499866365]:2.3254955000000024)[&length_range={0.086929,7.389781},length_95%_HPD={0.625799,4.58653},length=2.6482614659390196,posterior=0.9958770614692654,IgrBrlens{all}_median=0.120752,height_median=12.037704000000005,height_range={7.156749000000012,20.68378},height_95%_HPD={8.956921999999999,15.037655},IgrBrlens{all}_range={2.950698E-8,0.205556},length_median=2.627642,IgrBrlens{all}=0.12133874906067164,IgrBrlens{all}_95%_HPD={0.08499953,0.1621228},height=12.068920257182269]:2.703806999999994,(51[&length_range={4.000753,15.50668},height_95%_HPD={9.99999983264388E-7,2.200000000129876E-5},length_95%_HPD={5.295122,10.902397},IgrBrlens{all}_range={0.1421182,0.3566446},length=7.98770910319839,IgrBrlens{all}_median=0.21930739999999999,length_median=7.936894499999999,IgrBrlens{all}=0.22097048619440346,IgrBrlens{all}_95%_HPD={0.1690079,0.2714104},height_median=1.1999999998124622E-5,height_range={0.0,3.999999999848569E-5},height=1.1951274363403639E-5]:7.936890000000005,26[&length_range={4.000753,15.50668},height_95%_HPD={9.99999983264388E-7,2.200000000129876E-5},length_95%_HPD={5.295122,10.902397},IgrBrlens{all}_range={0.1803232,0.3746759},length=7.98770910319839,IgrBrlens{all}_median=0.25602605,length_median=7.936894499999999,IgrBrlens{all}=0.25815095339830035,IgrBrlens{all}_95%_HPD={0.2079312,0.3174645},height_median=1.1999999998124622E-5,height_range={0.0,3.999999999848569E-5},height=1.1951274363403639E-5]:7.936890000000005)[&length_range={2.111222,13.678989},length_95%_HPD={4.047533,9.820418},length=6.723834000374785,posterior=1.0,IgrBrlens{all}_median=0.18018685,height_median=7.9369020000000035,height_range={4.000765000000001,15.506703000000002},height_95%_HPD={5.29513399999999,10.902408000000008},IgrBrlens{all}_range={0.1064825,0.2941495},length_median=6.674196,IgrBrlens{all}=0.18194132291354306,IgrBrlens{all}_95%_HPD={0.1385702,0.2309281},height=7.987721054472741]:6.804608999999996)[&length_range={0.033193,4.733903},length_95%_HPD={0.144465,2.915062},length=1.479793531203007,posterior=0.9970014992503748,IgrBrlens{all}_median=0.045486165,height_median=14.741511,height_range={9.598605,23.305317000000002},height_95%_HPD={11.267822999999993,18.329288},IgrBrlens{all}_range={0.01089111,0.1089887},length_median=1.392054,IgrBrlens{all}=0.04628845164285709,IgrBrlens{all}_95%_HPD={0.02491757,0.06945122},height=14.716599085714279]:1.4572244999999988,((50[&length_range={2.377516,13.502895},height_95%_HPD={9.999999974752427E-7,2.2000000015509613E-5},length_95%_HPD={4.167293,9.558796},IgrBrlens{all}_range={0.1163694,0.3198258},length=6.810568104947537,IgrBrlens{all}_median=0.19044815,length_median=6.7513295,IgrBrlens{all}=0.19164260348575735,IgrBrlens{all}_95%_HPD={0.137985,0.2466446},height_median=1.1999999998124622E-5,height_range={0.0,4.0000000012696546E-5},height=1.1941279360765005E-5]:6.751330500000002,48[&length_range={2.377516,13.502895},height_95%_HPD={9.999999974752427E-7,2.2000000015509613E-5},length_95%_HPD={4.167293,9.558796},IgrBrlens{all}_range={0.1011298,0.3733559},length=6.810568104947537,IgrBrlens{all}_median=0.19778465,length_median=6.7513295,IgrBrlens{all}=0.20015692370065047,IgrBrlens{all}_95%_HPD={0.1460902,0.2618365},height_median=1.1999999998124622E-5,height_range={0.0,4.0000000012696546E-5},height=1.1941279360765005E-5]:6.751330500000002)[&length_range={0.865742,11.095894},length_95%_HPD={2.014742,7.452011},length=4.658002481384327,posterior=1.0,IgrBrlens{all}_median=0.11421970000000001,height_median=6.7513425,height_range={2.377521999999999,13.502915999999999},height_95%_HPD={4.167302999999997,9.558805000000007},IgrBrlens{all}_range={0.04644005,0.2201807},length_median=4.5696034999999995,IgrBrlens{all}=0.11586240871439321,IgrBrlens{all}_95%_HPD={0.07459287,0.1607484},height=6.810580046226883]:4.698839999999997,(46[&length_range={4.448147,13.847525},height_95%_HPD={9.999999974752427E-7,2.200000000129876E-5},length_95%_HPD={6.107248,11.143864},IgrBrlens{all}_range={0.1746978,0.408309},length=8.53571392878564,IgrBrlens{all}_median=0.25719205,length_median=8.503829,IgrBrlens{all}=0.2588833264367816,IgrBrlens{all}_95%_HPD={0.2052238,0.3147072},height_median=1.1999999998124622E-5,height_range={0.0,4.3000000005122274E-5},height=1.1939905047941763E-5]:8.503835500000015,((49[&length_range={2.271919,9.308901},height_95%_HPD={9.99999983264388E-7,2.200000000129876E-5},length_95%_HPD={3.29543,7.389439},IgrBrlens{all}_range={0.0773236,0.302415},length=5.2945907614942485,IgrBrlens{all}_median=0.1594332,length_median=5.258547500000001,IgrBrlens{all}=0.16126724082958507,IgrBrlens{all}_95%_HPD={0.107846,0.2173568},height_median=1.1999999998124622E-5,height_range={0.0,4.400000001680837E-5},height=1.1932658671193737E-5]:5.235006999999996,44[&length_range={2.271919,9.301324},height_95%_HPD={9.999999974752427E-7,2.2000000015509613E-5},length_95%_HPD={3.201367,7.271224},IgrBrlens{all}_range={0.08085552,0.2947566},length=5.281936632058966,IgrBrlens{all}_median=0.15825115,length_median=5.250399,IgrBrlens{all}=0.1600539873788104,IgrBrlens{all}_95%_HPD={0.1075926,0.2152355},height_median=1.1999999998124622E-5,height_range={0.0,4.400000001680837E-5},height=1.1935282359346587E-5]:5.235006999999996)[&length_range={0.036816,5.672884},length_95%_HPD={0.156999,2.914148},length=1.4277077761825383,posterior=0.9746376811594203,IgrBrlens{all}_median=0.02812011,height_median=5.235018999999994,height_range={2.271929,9.301332000000016},height_95%_HPD={3.236672999999996,7.280913000000012},IgrBrlens{all}_range={1.95328E-6,0.1059845},length_median=1.329981,IgrBrlens{all}=0.029554310748385302,IgrBrlens{all}_95%_HPD={0.005191011,0.05530921},height=5.264196005512124]:1.4205005000000028,((47[&length_range={1.616859,7.554078},height_95%_HPD={9.999999974752427E-7,2.2000000015509613E-5},length_95%_HPD={2.289256,5.289503},IgrBrlens{all}_range={0.05017757,0.2474828},length=3.7140792288855518,IgrBrlens{all}_median=0.11617939999999999,length_median=3.642812,IgrBrlens{all}=0.11802589485507231,IgrBrlens{all}_95%_HPD={0.07382085,0.1623357},height_median=1.1999999998124622E-5,height_range={0.0,4.0999999995960934E-5},height=1.1931284358180521E-5]:3.6428125000000016,45[&length_range={1.616859,7.554078},height_95%_HPD={9.999999974752427E-7,2.2000000015509613E-5},length_95%_HPD={2.289256,5.289503},IgrBrlens{all}_range={0.07495458,0.2976325},length=3.7140792288855518,IgrBrlens{all}_median=0.1499317,length_median=3.642812,IgrBrlens{all}=0.15217013727761122,IgrBrlens{all}_95%_HPD={0.1008544,0.2040823},height_median=1.1999999998124622E-5,height_range={0.0,4.0999999995960934E-5},height=1.1931284358180521E-5]:3.6428125000000016)[&length_range={0.094729,5.142248},length_95%_HPD={0.32679,2.886018},length=1.5251389956271846,posterior=1.0,IgrBrlens{all}_median=0.054731385,height_median=3.6428244999999997,height_range={1.6168699999999916,7.5540869999999956},height_95%_HPD={2.2892639999999957,5.289504000000008},IgrBrlens{all}_range={0.01313934,0.1666581},length_median=1.4545385,IgrBrlens{all}=0.05636088276736612,IgrBrlens{all}_95%_HPD={0.02741525,0.0877402},height=3.7140911601699185]:1.5520504999999893,19[&length_range={2.405352,9.184934},height_95%_HPD={9.99999983264388E-7,2.200000000129876E-5},length_95%_HPD={3.360387,7.033885},IgrBrlens{all}_range={0.08076574,0.2744736},length=5.239218224137929,IgrBrlens{all}_median=0.14405995,length_median=5.194868,IgrBrlens{all}=0.14526089721764127,IgrBrlens{all}_95%_HPD={0.100041,0.189467},height_median=1.1999999998124622E-5,height_range={0.0,4.100000001017179E-5},height=1.1931659170973018E-5]:5.194862999999991)[&length_range={0.050639,4.940513},length_95%_HPD={0.170464,2.802642},length=1.433787309095454,posterior=1.0,IgrBrlens{all}_median=0.044970085,height_median=5.194874999999989,height_range={2.4053539999999956,9.184957000000011},height_95%_HPD={3.3603999999999985,7.0338899999999995},IgrBrlens{all}_range={0.007934739,0.1335983},length_median=1.347324,IgrBrlens{all}=0.046249961979760085,IgrBrlens{all}_95%_HPD={0.01911121,0.07607629},height=5.239230155797116]:1.460644500000008)[&length_range={0.022739,5.643362},length_95%_HPD={0.268141,3.473109},length=1.8491964793853115,posterior=1.0,IgrBrlens{all}_median=0.05555245,height_median=6.655519499999997,height_range={3.561360999999991,10.726307000000006},height_95%_HPD={4.5550210000000035,8.717823999999993},IgrBrlens{all}_range={0.01249706,0.1414745},length_median=1.777382,IgrBrlens{all}=0.05676574098575702,IgrBrlens{all}_95%_HPD={0.02733372,0.08847294},height=6.6865293893053925]:1.8483280000000164)[&length_range={0.13889,8.530071},length_95%_HPD={1.003197,5.029661},length=2.932856658920552,posterior=1.0,IgrBrlens{all}_median=0.092216255,height_median=8.503847500000013,height_range={4.448157000000002,13.847541000000007},height_95%_HPD={6.107265000000005,11.143882999999988},IgrBrlens{all}_range={0.04428481,0.1610074},length_median=2.8780954999999997,IgrBrlens{all}=0.09328215380434773,IgrBrlens{all}_95%_HPD={0.06300697,0.1236025},height=8.535725868690646]:2.9463349999999835)[&length_range={0.60945,10.61985},length_95%_HPD={2.206043,7.174868},length=4.722826330834607,posterior=1.0,IgrBrlens{all}_median=0.11977689999999999,height_median=11.450182499999997,height_range={5.7359440000000035,17.86533999999999},height_95%_HPD={8.45277800000001,14.677053999999998},IgrBrlens{all}_range={0.06483207,0.198815},length_median=4.632916,IgrBrlens{all}=0.12086327458770631,IgrBrlens{all}_95%_HPD={0.08700849,0.1554969},height=11.46858252761119]:4.748553000000001)[&length_range={1.808265,13.158966},length_95%_HPD={3.779141,9.614899},length=6.642420644802612,posterior=1.0,IgrBrlens{all}_median=0.24087625000000001,height_median=16.198735499999998,height_range={10.047576,25.66583},height_95%_HPD={12.247426000000004,19.810829000000005},IgrBrlens{all}_range={0.1562177,0.3740808},length_median=6.567724999999999,IgrBrlens{all}=0.24304030183658173,IgrBrlens{all}_95%_HPD={0.1869822,0.3022934},height=16.19463108508247]:6.744300500000005,((39[&length_range={8.350849,20.641638},height_95%_HPD={1.00000000458067E-6,2.200000000129876E-5},length_95%_HPD={10.177881,17.198729},IgrBrlens{all}_range={0.2121953,0.4537228},length=13.67484693303348,IgrBrlens{all}_median=0.30238735,length_median=13.687065,IgrBrlens{all}=0.30479147157671305,IgrBrlens{all}_95%_HPD={0.2422097,0.3686758},height_median=1.1999999998124622E-5,height_range={0.0,3.90000000152213E-5},height=1.1945902049442739E-5]:13.687065999999994,(43[&length_range={4.692778,15.09367},height_95%_HPD={9.999999974752427E-7,2.200000000129876E-5},length_95%_HPD={6.52437,12.127453},IgrBrlens{all}_range={0.1885678,0.48216},length=9.33702088118439,IgrBrlens{all}_median=0.29186965,length_median=9.283924500000001,IgrBrlens{all}=0.29446410744627605,IgrBrlens{all}_95%_HPD={0.2190555,0.3653466},height_median=1.1999999998124622E-5,height_range={0.0,3.800000001774606E-5},height=1.19445277365831E-5]:9.283927000000006,(41[&length_range={3.580144,12.718823},height_95%_HPD={9.999999974752427E-7,2.200000000129876E-5},length_95%_HPD={4.572309,9.311042},IgrBrlens{all}_range={0.1244118,0.3872633},length=6.978078813718135,IgrBrlens{all}_median=0.21247575000000002,length_median=6.926299,IgrBrlens{all}=0.2146530353448265,IgrBrlens{all}_95%_HPD={0.1495922,0.2767211},height_median=1.1999999998124622E-5,height_range={0.0,3.800000001774606E-5},height=1.1960269865559672E-5]:6.926300499999996,((40[&length_range={1.455229,6.53116},height_95%_HPD={9.999999974752427E-7,2.200000000129876E-5},length_95%_HPD={1.976863,4.483231},IgrBrlens{all}_range={0.06893283,0.2231798},length=3.186747263243368,IgrBrlens{all}_median=0.124942,length_median=3.1143590000000003,IgrBrlens{all}=0.12639665422663587,IgrBrlens{all}_95%_HPD={0.08803373,0.1688062},height_median=1.1999999998124622E-5,height_range={0.0,3.90000000152213E-5},height=1.196451774151859E-5]:3.1135989999999936,17[&length_range={1.455229,6.53116},height_95%_HPD={9.999999974752427E-7,2.200000000129876E-5},length_95%_HPD={1.982556,4.483231},IgrBrlens{all}_range={0.03437214,0.1719423},length=3.184906614817582,IgrBrlens{all}_median=0.07839128000000001,length_median=3.1132825000000004,IgrBrlens{all}=0.07973905861944018,IgrBrlens{all}_95%_HPD={0.04943061,0.1131831},height_median=1.1999999998124622E-5,height_range={0.0,3.90000000152213E-5},height=1.1964017991391364E-5]:3.1135989999999936)[&length_range={0.054491,5.725594},length_95%_HPD={0.193016,2.886637},length=1.5277214843026952,posterior=0.9988755622188905,IgrBrlens{all}_median=0.06724665,height_median=3.1136109999999917,height_range={1.4552360000000064,6.5311710000000005},height_95%_HPD={1.9825599999999923,4.483213000000006},IgrBrlens{all}_range={0.02385286,0.1734945},length_median=1.453513,IgrBrlens{all}=0.06837255832770447,IgrBrlens{all}_95%_HPD={0.0373088,0.09847621},height=3.1850868492808124]:1.5183120000000159,42[&length_range={1.895418,9.500306},height_95%_HPD={9.99999983264388E-7,2.200000000129876E-5},length_95%_HPD={3.065148,6.624988},IgrBrlens{all}_range={0.0451586,0.1811344},length=4.710910274987499,IgrBrlens{all}_median=0.08935011,length_median=4.6306650000000005,IgrBrlens{all}=0.0906888256521737,IgrBrlens{all}_95%_HPD={0.05963812,0.1231272},height_median=1.1999999998124622E-5,height_range={0.0,3.900000002943216E-5},height=1.196039480300803E-5]:4.631911000000009)[&length_range={0.189896,7.542905},length_95%_HPD={0.503084,4.065368},length=2.2653278896801528,posterior=1.0,IgrBrlens{all}_median=0.06226296,height_median=4.631923000000008,height_range={2.1496780000000015,9.50032499999999},height_95%_HPD={3.0651500000000027,6.620028000000005},IgrBrlens{all}_range={0.01595714,0.1577353},length_median=2.1959215,IgrBrlens{all}=0.06356201065842075,IgrBrlens{all}_95%_HPD={0.03154027,0.09616334},height=4.712762884307846]:2.294389499999987)[&length_range={0.215523,7.832561},length_95%_HPD={0.602699,4.315189},length=2.358942051724131,posterior=1.0,IgrBrlens{all}_median=0.06316176500000001,height_median=6.9263124999999945,height_range={3.5801560000000023,12.718837999999991},height_95%_HPD={4.572324000000009,9.311047000000002},IgrBrlens{all}_range={0.01726204,0.1390512},length_median=2.2613205,IgrBrlens{all}=0.06440713307471278,IgrBrlens{all}_95%_HPD={0.03379189,0.0980894},height=6.978090773988018]:2.357626500000009)[&length_range={1.005572,9.186715},length_95%_HPD={1.92209,6.794885},length=4.337826053223387,posterior=1.0,IgrBrlens{all}_median=0.1209292,height_median=9.283939000000004,height_range={4.692791,15.093691000000021},height_95%_HPD={6.524375999999997,12.127471999999997},IgrBrlens{all}_range={0.05153193,0.2169529},length_median=4.2720395,IgrBrlens{all}=0.12238820230759594,IgrBrlens{all}_95%_HPD={0.08184496,0.1652347},height=9.337032825712123]:4.403138999999989)[&length_range={0.109463,6.880784},length_95%_HPD={0.406662,3.892535},length=2.0468651453023523,posterior=1.0,IgrBrlens{all}_median=0.032727864999999995,height_median=13.687077999999993,height_range={8.350856,20.641642999999988},height_95%_HPD={10.177888999999993,17.19873300000002},IgrBrlens{all}_range={0.007763083,0.07550029},length_median=1.924807,IgrBrlens{all}=0.03330896065042485,IgrBrlens{all}_95%_HPD={0.01598821,0.05092843},height=13.674858878935556]:2.094847000000005,((9[&length_range={8.183689,20.653998},height_95%_HPD={1.0000000116860974E-6,2.200000000129876E-5},length_95%_HPD={10.078356,16.542754},IgrBrlens{all}_range={0.2984856,0.6052528},length=13.376064541104387,IgrBrlens{all}_median=0.41220924999999997,length_median=13.4280625,IgrBrlens{all}=0.4152422745377305,IgrBrlens{all}_95%_HPD={0.3374953,0.5001472},height_median=1.1999999998124622E-5,height_range={0.0,4.100000001017179E-5},height=1.1949400300458497E-5]:13.54093450000001,((16[&length_range={6.411423,17.01312},height_95%_HPD={1.0000000116860974E-6,2.200000000129876E-5},length_95%_HPD={8.226851,14.105149},IgrBrlens{all}_range={0.2733699,0.6117197},length=11.228564573963032,IgrBrlens{all}_median=0.38975004999999996,length_median=11.2699485,IgrBrlens{all}=0.39337928282108975,IgrBrlens{all}_95%_HPD={0.3164939,0.4771711},height_median=1.1999999998124622E-5,height_range={0.0,3.900000000101045E-5},height=1.195989505307877E-5]:11.269947500000008,(37[&length_range={3.382968,11.86724},height_95%_HPD={9.999999974752427E-7,2.200000000129876E-5},length_95%_HPD={4.702515,9.306053},IgrBrlens{all}_range={0.1139436,0.3338797},length=6.991866275862094,IgrBrlens{all}_median=0.19684875000000002,length_median=6.954431,IgrBrlens{all}=0.19935185804597713,IgrBrlens{all}_95%_HPD={0.146348,0.2611229},height_median=1.1999999998124622E-5,height_range={0.0,4.3000000005122274E-5},height=1.1956896552316297E-5]:6.954432000000001,(38[&length_range={2.441404,9.629006},height_95%_HPD={9.999999974752427E-7,2.200000000129876E-5},length_95%_HPD={3.420261,7.245912},IgrBrlens{all}_range={0.08713113,0.2833815},length=5.312341220764616,IgrBrlens{all}_median=0.1646844,length_median=5.2637495,IgrBrlens{all}=0.1666210403948021,IgrBrlens{all}_95%_HPD={0.1183927,0.2188578},height_median=1.1999999998124622E-5,height_range={0.0,3.90000000152213E-5},height=1.1971139430809986E-5]:5.263753000000001,(36[&length_range={1.50134,6.821527},height_95%_HPD={9.999999974752427E-7,2.200000000129876E-5},length_95%_HPD={1.934559,4.358794},IgrBrlens{all}_range={0.02291136,0.1744557},length=3.164370897801106,IgrBrlens{all}_median=0.07883427500000001,length_median=3.1052014999999997,IgrBrlens{all}=0.08058250048600674,IgrBrlens{all}_95%_HPD={0.04577504,0.1176788},height_median=1.1999999998124622E-5,height_range={0.0,4.0000000012696546E-5},height=1.1967391304811398E-5]:3.1051974999999885,13[&length_range={1.50134,6.821527},height_95%_HPD={9.999999974752427E-7,2.200000000129876E-5},length_95%_HPD={1.934559,4.358794},IgrBrlens{all}_range={0.0412449,0.1852347},length=3.164370897801106,IgrBrlens{all}_median=0.09638643999999999,length_median=3.1052014999999997,IgrBrlens{all}=0.09804983307471316,IgrBrlens{all}_95%_HPD={0.06395418,0.1376118},height_median=1.1999999998124622E-5,height_range={0.0,4.0000000012696546E-5},height=1.1967391304811398E-5]:3.1051974999999885)[&length_range={0.077731,6.797318},length_95%_HPD={0.600617,3.791154},length=2.1479703267116435,posterior=1.0,IgrBrlens{all}_median=0.08350516499999999,height_median=3.1052094999999866,height_range={1.5013520000000113,6.821548000000021},height_95%_HPD={1.934572000000017,4.358803999999992},IgrBrlens{all}_range={0.02603077,0.1986201},length_median=2.095621,IgrBrlens{all}=0.08471770826461762,IgrBrlens{all}_95%_HPD={0.04639134,0.1235407},height=3.1643828651923975]:2.1585555000000127)[&length_range={0.022031,6.488762},length_95%_HPD={0.264869,3.158038},length=1.6795250408545794,posterior=1.0,IgrBrlens{all}_median=0.055054305,height_median=5.263764999999999,height_range={2.4414189999999962,9.629024000000001},height_95%_HPD={3.4202769999999987,7.245931000000006},IgrBrlens{all}_range={0.01250458,0.1397102},length_median=1.590142,IgrBrlens{all}=0.05625181391804104,IgrBrlens{all}_95%_HPD={0.02647728,0.08941073},height=5.31235319190403]:1.6906789999999994)[&length_range={0.632875,11.037171},length_95%_HPD={1.845595,6.474357},length=4.236698301099461,posterior=1.0,IgrBrlens{all}_median=0.15756525,height_median=6.954443999999999,height_range={3.3829779999999943,11.867253000000005},height_95%_HPD={4.702524999999994,9.30606299999998},IgrBrlens{all}_range={0.08327109,0.2697745},length_median=4.179262,IgrBrlens{all}=0.15890628843703108,IgrBrlens{all}_95%_HPD={0.1138774,0.2073295},height=6.99187823275863]:4.315515500000007)[&length_range={0.041331,5.918426},length_95%_HPD={0.169003,2.929244},length=1.482609631434285,posterior=1.0,IgrBrlens{all}_median=0.04339016,height_median=11.269959500000006,height_range={6.411430000000003,17.013141999999988},height_95%_HPD={8.226864000000013,14.105162000000007},IgrBrlens{all}_range={0.009674592,0.1009513},length_median=1.3896415,IgrBrlens{all}=0.04415971236406807,IgrBrlens{all}_95%_HPD={0.02291863,0.0665606},height=11.22857653385807]:1.4257894999999863,(11[&length_range={4.847942,15.424626},height_95%_HPD={9.999999974752427E-7,2.200000000129876E-5},length_95%_HPD={6.553135,12.442475},IgrBrlens{all}_range={0.2235402,0.4669741},length=9.644166772988523,IgrBrlens{all}_median=0.3169729,length_median=9.6228075,IgrBrlens{all}=0.31964909633932914,IgrBrlens{all}_95%_HPD={0.2576596,0.3883314},height_median=1.1999999998124622E-5,height_range={0.0,3.900000000101045E-5},height=1.1964142929145105E-5]:9.622807000000002,10[&length_range={4.847942,15.424626},height_95%_HPD={9.999999974752427E-7,2.200000000129876E-5},length_95%_HPD={6.553135,12.442475},IgrBrlens{all}_range={0.2171691,0.4806966},length=9.644166772988523,IgrBrlens{all}_median=0.32425975,length_median=9.6228075,IgrBrlens{all}=0.3275058643303352,IgrBrlens{all}_95%_HPD={0.262222,0.3995414},height_median=1.1999999998124622E-5,height_range={0.0,3.900000000101045E-5},height=1.1964142929145105E-5]:9.622807000000002)[&length_range={0.310012,7.987307},length_95%_HPD={0.919943,5.060022},length=2.9545258601949147,posterior=1.0,IgrBrlens{all}_median=0.085886205,height_median=9.622819,height_range={4.847950000000004,15.424632000000017},height_95%_HPD={6.553138000000004,12.442491000000004},IgrBrlens{all}_range={0.04430385,0.1648095},length_median=2.874198,IgrBrlens{all}=0.08702137842828607,IgrBrlens{all}_95%_HPD={0.05741914,0.1164848},height=9.644178737131444]:3.0729299999999924)[&length_range={0.010048,4.52489},length_95%_HPD={0.071618,1.871447},length=0.8732676282428832,posterior=0.8909295352323838,IgrBrlens{all}_median=0.01804502,height_median=12.695748999999992,height_range={7.544873000000003,20.209990000000005},height_95%_HPD={9.467011,15.619890999999996},IgrBrlens{all}_range={0.001930418,0.06564131},length_median=0.79768,IgrBrlens{all}=0.018671805229140425,IgrBrlens{all}_95%_HPD={0.006091189,0.03189493},height=12.639274612396598]:0.8451975000000154)[&length_range={0.021773,5.068294},length_95%_HPD={0.228913,2.826653},length=1.429586791104448,posterior=1.0,IgrBrlens{all}_median=0.037600259999999996,height_median=13.540946500000008,height_range={8.25306599999999,20.654006000000003},height_95%_HPD={10.218553000000007,16.489053},IgrBrlens{all}_range={0.01534796,0.08439408},length_median=1.3407974999999999,IgrBrlens{all}=0.03824835554097952,IgrBrlens{all}_95%_HPD={0.02179267,0.05594076},height=13.489854116441741]:1.4237459999999977,((15[&length_range={3.371255,12.366837},height_95%_HPD={9.999999974752427E-7,2.200000000129876E-5},length_95%_HPD={4.642819,9.932501},IgrBrlens{all}_range={0.1838811,0.421495},length=7.34746843390806,IgrBrlens{all}_median=0.26051,length_median=7.292025,IgrBrlens{all}=0.2622657576711642,IgrBrlens{all}_95%_HPD={0.2111305,0.3194697},height_median=1.1999999998124622E-5,height_range={0.0,3.9999999984274837E-5},height=1.1979760120465585E-5]:7.292028999999999,14[&length_range={3.371255,12.366837},height_95%_HPD={9.999999974752427E-7,2.200000000129876E-5},length_95%_HPD={4.642819,9.932501},IgrBrlens{all}_range={0.1516804,0.37196},length=7.34746843390806,IgrBrlens{all}_median=0.2399958,length_median=7.292025,IgrBrlens{all}=0.2419986463518233,IgrBrlens{all}_95%_HPD={0.1934363,0.298546},height_median=1.1999999998124622E-5,height_range={0.0,3.9999999984274837E-5},height=1.1979760120465585E-5]:7.292028999999999)[&length_range={1.834565,11.945464},length_95%_HPD={3.650659,8.566183},length=5.962886954897532,posterior=1.0,IgrBrlens{all}_median=0.21324484999999999,height_median=7.2920409999999976,height_range={3.3712660000000056,12.36685},height_95%_HPD={4.6428240000000045,9.932504999999992},IgrBrlens{all}_range={0.1472089,0.3724419},length_median=5.8955955,IgrBrlens{all}=0.2148306285357318,IgrBrlens{all}_95%_HPD={0.1689005,0.2656536},height=7.347480413668162]:6.057161000000011,(18[&length_range={7.206765,17.731052},height_95%_HPD={1.9999999807396307E-6,2.2000000015509613E-5},length_95%_HPD={8.597697,14.466326},IgrBrlens{all}_range={0.287906,0.6424541},length=11.601655285607213,IgrBrlens{all}_median=0.41274485,length_median=11.6030295,IgrBrlens{all}=0.4164430040854566,IgrBrlens{all}_95%_HPD={0.3346961,0.5014988},height_median=1.1999999998124622E-5,height_range={0.0,4.100000001017179E-5},height=1.1974012993988275E-5]:11.603026499999991,(((35[&length_range={3.116046,11.303695},height_95%_HPD={9.999999974752427E-7,2.200000000129876E-5},length_95%_HPD={4.489869,8.683913},IgrBrlens{all}_range={0.09274441,0.3224954},length=6.61825233058471,IgrBrlens{all}_median=0.17688959999999998,length_median=6.605418,IgrBrlens{all}=0.17899511710769678,IgrBrlens{all}_95%_HPD={0.1232088,0.2366878},height_median=1.1999999998124622E-5,height_range={0.0,4.0000000012696546E-5},height=1.1958395802558602E-5]:6.573200999999997,(31[&length_range={2.888221,9.349122},height_95%_HPD={9.999999974752427E-7,2.200000000129876E-5},length_95%_HPD={3.802913,7.515671},IgrBrlens{all}_range={0.1121735,0.3065208},length=5.606416956396801,IgrBrlens{all}_median=0.1879674,length_median=5.589617499999999,IgrBrlens{all}=0.19045384331584203,IgrBrlens{all}_95%_HPD={0.1391186,0.2459942},height_median=1.1999999998124622E-5,height_range={0.0,3.799999998932435E-5},height=1.1948150925048106E-5]:5.551404499999997,(34[&length_range={1.302294,5.685517},height_95%_HPD={9.999999974752427E-7,2.200000000129876E-5},length_95%_HPD={1.956042,4.100201},IgrBrlens{all}_range={0.03251922,0.1771656},length=2.975847768115935,IgrBrlens{all}_median=0.08597752,length_median=2.9455535,IgrBrlens{all}=0.08735035361319374,IgrBrlens{all}_95%_HPD={0.0536532,0.1235176},height_median=1.1999999998124622E-5,height_range={0.0,4.0000000012696546E-5},height=1.1964642679181782E-5]:2.9455554999999976,32[&length_range={1.302294,5.685517},height_95%_HPD={9.999999974752427E-7,2.200000000129876E-5},length_95%_HPD={1.956042,4.100201},IgrBrlens{all}_range={0.0224847,0.1351342},length=2.975847768115935,IgrBrlens{all}_median=0.056775690000000004,length_median=2.9455535,IgrBrlens{all}=0.0580143326249374,IgrBrlens{all}_95%_HPD={0.03265087,0.08487793},height_median=1.1999999998124622E-5,height_range={0.0,4.0000000012696546E-5},height=1.1964642679181782E-5]:2.9455554999999976)[&length_range={0.169961,5.853388},length_95%_HPD={1.032197,4.228335},length=2.5899273638181017,posterior=1.0,IgrBrlens{all}_median=0.1078888,height_median=2.9455674999999957,height_range={1.3023000000000025,5.685527999999977},height_95%_HPD={1.9560490000000001,4.100211999999985},IgrBrlens{all}_range={0.05605696,0.2080147},length_median=2.563933,IgrBrlens{all}=0.10907447072588705,IgrBrlens{all}_95%_HPD={0.07312003,0.14713},height=2.975859732758621]:2.605848999999999)[&length_range={0.036147,4.365542},length_95%_HPD={0.08634,2.121839},length=1.0421415033845398,posterior=0.9597701149425287,IgrBrlens{all}_median=0.028287005,height_median=5.551416499999995,height_range={2.88823399999999,9.202252999999985},height_95%_HPD={3.808729999999983,7.424133000000012},IgrBrlens{all}_range={0.004098797,0.07891926},length_median=0.9522025000000001,IgrBrlens{all}=0.029412250189013354,IgrBrlens{all}_95%_HPD={0.0114739,0.05057107},height=5.5637333263473066]:1.0217965000000007)[&length_range={0.001878,4.150047},length_95%_HPD={0.011599,1.687541},length=0.766110455059436,posterior=0.8618190904547726,IgrBrlens{all}_median=0.012930035,height_median=6.5732129999999955,height_range={3.390209999999989,11.039517000000018},height_95%_HPD={4.545147,8.515464000000009},IgrBrlens{all}_range={3.354371E-8,0.06392923},length_median=0.6733215,IgrBrlens{all}=0.01421668037621549,IgrBrlens{all}_95%_HPD={3.354371E-8,0.02903252},height=6.576915082197759]:0.7608535000000032,33[&length_range={4.064361,11.745315},height_95%_HPD={9.999999974752427E-7,2.200000000129876E-5},length_95%_HPD={5.141115,9.4978},IgrBrlens{all}_range={0.1495952,0.3881211},length=7.253233521364313,IgrBrlens{all}_median=0.24001794999999998,length_median=7.246262,IgrBrlens{all}=0.24219167654922508,IgrBrlens{all}_95%_HPD={0.1817331,0.3062115},height_median=1.1999999998124622E-5,height_range={0.0,4.100000001017179E-5},height=1.1974887556685178E-5]:7.334054500000001)[&length_range={0.249203,6.04976},length_95%_HPD={0.685989,4.020592},length=2.36199999975012,posterior=1.0,IgrBrlens{all}_median=0.07646871499999999,height_median=7.334066499999999,height_range={4.064372999999989,11.745327999999986},height_95%_HPD={5.146482000000013,9.467798000000002},IgrBrlens{all}_range={0.03279364,0.1591567},length_median=2.280485,IgrBrlens{all}=0.07761751858195884,IgrBrlens{all}_95%_HPD={0.04855696,0.1092592},height=7.338055993128472]:2.353409500000012,12[&length_range={5.774784,15.032165},height_95%_HPD={1.0000000116860974E-6,2.200000000129876E-5},length_95%_HPD={6.949977,12.283368},IgrBrlens{all}_range={0.3000457,0.6134012},length=9.70004402873565,IgrBrlens{all}_median=0.4039658,length_median=9.687467000000002,IgrBrlens{all}=0.4076320601324354,IgrBrlens{all}_95%_HPD={0.3306266,0.490307},height_median=1.1999999998124622E-5,height_range={0.0,4.0000000012696546E-5},height=1.196414292899774E-5]:9.687464000000013)[&length_range={0.153042,5.966994},length_95%_HPD={0.311924,3.402611},length=1.9016112667416298,posterior=1.0,IgrBrlens{all}_median=0.071659,height_median=9.68747600000001,height_range={5.774790999999986,15.032188000000005},height_95%_HPD={6.949987999999998,12.283373999999995},IgrBrlens{all}_range={0.02720523,0.1440023},length_median=1.8305699999999998,IgrBrlens{all}=0.07242822361569204,IgrBrlens{all}_95%_HPD={0.04709113,0.09962078},height=9.700055992878518]:1.9155624999999787)[&length_range={0.04063,5.806829},length_95%_HPD={0.287843,3.222247},length=1.7087001089455311,posterior=1.0,IgrBrlens{all}_median=0.06834657999999999,height_median=11.60303849999999,height_range={7.206776999999988,17.731074000000007},height_95%_HPD={8.597714000000003,14.466340000000002},IgrBrlens{all}_range={0.03495351,0.1289667},length_median=1.6269580000000001,IgrBrlens{all}=0.06907624502748626,IgrBrlens{all}_95%_HPD={0.04646125,0.09462196},height=11.601667259620173]:1.7461635000000193)[&length_range={0.065167,5.315116},length_95%_HPD={0.233309,3.02684},length=1.6090735389805144,posterior=1.0,IgrBrlens{all}_median=0.07127434499999999,height_median=13.349202000000009,height_range={8.454023,19.32437300000001},height_95%_HPD={10.113752999999996,16.459606},IgrBrlens{all}_range={0.03346138,0.1312538},length_median=1.5368555000000002,IgrBrlens{all}=0.07209268774112919,IgrBrlens{all}_95%_HPD={0.04931707,0.09636561},height=13.31036736856569]:1.6154904999999964)[&length_range={0.02936,3.777256},length_95%_HPD={0.063718,1.664027},length=0.8022831166916555,posterior=1.0,IgrBrlens{all}_median=0.0260471,height_median=14.964692500000005,height_range={9.204581000000005,21.447592000000007},height_95%_HPD={11.235641000000001,18.093484999999994},IgrBrlens{all}_range={0.006825508,0.06181281},length_median=0.7323275,IgrBrlens{all}=0.026590553572338856,IgrBrlens{all}_95%_HPD={0.01380241,0.04062782},height=14.919440907546191]:0.8172324999999923)[&length_range={2.551338,14.039265},length_95%_HPD={4.220783,10.147491},length=7.11532770564718,posterior=1.0,IgrBrlens{all}_median=0.29503735,height_median=15.781924999999998,height_range={9.874434999999998,22.570759000000002},height_95%_HPD={12.111016000000006,19.289898000000008},IgrBrlens{all}_range={0.2062395,0.4707977},length_median=7.063135000000001,IgrBrlens{all}=0.29765212558720694,IgrBrlens{all}_95%_HPD={0.2331657,0.364985},height=15.721724024237892]:7.161111000000005)[&length_range={1.226578,11.64152},length_95%_HPD={2.57542,8.503527},length=5.626938578835578,posterior=1.0,IgrBrlens{all}_median=0.24854474999999998,height_median=22.943036000000003,height_range={14.423266999999996,33.034032},height_95%_HPD={17.567505999999995,27.711667},IgrBrlens{all}_range={0.1549399,0.392608},length_median=5.5656285,IgrBrlens{all}=0.2506960792978508,IgrBrlens{all}_95%_HPD={0.193634,0.3173689},height=22.837051729885083]:5.7014365)[&length_range={2.602745,19.066615},length_95%_HPD={5.430096,13.787457},length=9.504959535857028,posterior=1.0,IgrBrlens{all}_median=0.4273188,height_median=28.644472500000003,height_range={18.582554000000002,38.961096},height_95%_HPD={22.167141,34.490183},IgrBrlens{all}_range={0.2710378,0.7095647},length_median=9.498076999999999,IgrBrlens{all}=0.4311219258620696,IgrBrlens{all}_95%_HPD={0.3278451,0.5360901},height=28.463990308720586]:9.5763925,8[&length_range={23.599606,51.684747},height_95%_HPD={0.0,2.800000000036107E-5},length_95%_HPD={29.580709,45.613829},IgrBrlens{all}_range={0.9515447,1.941982},length=37.95346894502763,IgrBrlens{all}_median=1.3057439999999998,length_median=38.2075815,IgrBrlens{all}=1.3191980963643184,IgrBrlens{all}_95%_HPD={1.050235,1.575273},height_median=1.100000000064938E-5,height_range={0.0,4.500000000007276E-5},height=1.1832208896107646E-5]:38.220854)[&length_range={0.357213,11.855978},length_95%_HPD={1.237849,7.948852},length=4.612817611732182,posterior=0.9946276861569215,IgrBrlens{all}_median=0.2045562,height_median=38.220865,height_range={23.599615999999997,51.68477200000001},height_95%_HPD={29.580709999999996,45.613839000000006},IgrBrlens{all}_range={0.03097099,0.4451377},length_median=4.482849,IgrBrlens{all}=0.20733229201356668,IgrBrlens{all}_95%_HPD={0.09841987,0.3171235},height=37.96237174274591]:4.672429999999991,7[&length_range={26.036033,59.347588},height_95%_HPD={0.0,2.8000000014571924E-5},length_95%_HPD={33.40302,51.192224},IgrBrlens{all}_range={0.9750654,2.198442},length=42.541504888430765,IgrBrlens{all}_median=1.4012105,length_median=42.886455,IgrBrlens{all}=1.4146836579710107,IgrBrlens{all}_95%_HPD={1.125725,1.73247},height_median=1.100000000064938E-5,height_range={0.0,5.600000000072214E-5},height=1.199662668712899E-5]:42.893283999999994)[&length_range={10.095095,33.174802},length_95%_HPD={14.496573,27.577042},length=21.012019432408877,posterior=1.0,IgrBrlens{all}_median=1.0128944999999998,height_median=42.893294999999995,height_range={27.684558000000003,59.347588},height_95%_HPD={33.436257,51.166720999999995},IgrBrlens{all}_range={0.6774983,1.719656},length_median=20.981651499999998,IgrBrlens{all}=1.0242101989755126,IgrBrlens{all}_95%_HPD={0.7934906,1.271596},height=42.5569859523987]:21.49307500000002,(6[&length_range={5.270567,24.0404},height_95%_HPD={0.0,2.8000000014571924E-5},length_95%_HPD={8.195191,16.589989},IgrBrlens{all}_range={0.2896474,0.7082273},length=12.625624543603204,IgrBrlens{all}_median=0.4462293,length_median=12.580205,IgrBrlens{all}=0.45092200327336385,IgrBrlens{all}_95%_HPD={0.340303,0.5635971},height_median=1.100000000064938E-5,height_range={0.0,4.5999999997548E-5},height=1.1973138431336647E-5]:12.580205000000007,5[&length_range={5.270567,24.0404},height_95%_HPD={0.0,2.8000000014571924E-5},length_95%_HPD={8.195191,16.589989},IgrBrlens{all}_range={0.2677398,0.6333213},length=12.625624543603204,IgrBrlens{all}_median=0.4012418,length_median=12.580205,IgrBrlens{all}=0.4049723692403799,IgrBrlens{all}_95%_HPD={0.3098462,0.5092026},height_median=1.100000000064938E-5,height_range={0.0,4.5999999997548E-5},height=1.1973138431336647E-5]:12.580205000000007)[&length_range={33.269828,68.2861},length_95%_HPD={39.141521,60.732735},length=50.940994487631194,posterior=1.0,IgrBrlens{all}_median=1.6415915,height_median=12.580216000000007,height_range={5.270575000000001,24.040412000000003},height_95%_HPD={8.195191000000001,16.589991999999995},IgrBrlens{all}_range={1.131803,2.466743},length_median=51.405263500000004,IgrBrlens{all}=1.654909976386807,IgrBrlens{all}_95%_HPD={1.333192,1.980386},height=12.62563651674165]:51.80615400000001)[&length_range={0.409703,14.349677},length_95%_HPD={1.387556,7.243762},length=4.181960116110484,posterior=0.9996251874062968,IgrBrlens{all}_median=0.1722514,height_median=64.38637000000001,height_range={43.171291000000004,79.16783500000001},height_95%_HPD={49.903001,74.431769},IgrBrlens{all}_range={0.05973694,0.3801813},length_median=4.052509,IgrBrlens{all}=0.1750941875065615,IgrBrlens{all}_95%_HPD={0.1014267,0.25418},height=63.566361404574394]:4.255979999999994)[&length_range={0.017412,19.613432},length_95%_HPD={0.627029,12.401917},length=6.772584468515769,posterior=1.0,IgrBrlens{all}_median=0.2202538,height_median=68.64235000000001,height_range={44.6274,82.90981400000001},height_95%_HPD={54.10040600000001,79.56373300000001},IgrBrlens{all}_range={3.803249E-5,0.7057052},length_median=6.681297000000001,IgrBrlens{all}=0.22523246109461706,IgrBrlens{all}_95%_HPD={1.970715E-4,0.422763},height=67.74939804960042]:7.260269499999993,(1[&length_range={23.456844,52.116235},height_95%_HPD={0.0,3.100000000699765E-5},length_95%_HPD={27.666686,44.113787},IgrBrlens{all}_range={0.6601214,1.643335},length=36.30580451086944,IgrBrlens{all}_median=0.95620645,length_median=36.443638,IgrBrlens{all}=0.9640833452773597,IgrBrlens{all}_95%_HPD={0.7537159,1.174738},height_median=1.100000000064938E-5,height_range={0.0,5.000000000165983E-5},height=1.2116191904649861E-5]:36.443638500000006,(3[&length_range={16.004005,41.265518},height_95%_HPD={0.0,2.700000001709668E-5},length_95%_HPD={20.922073,34.76612},IgrBrlens{all}_range={0.6477634,1.485142},length=28.19584838768108,IgrBrlens{all}_median=0.9334690999999999,length_median=28.232449000000003,IgrBrlens{all}=0.940089282746127,IgrBrlens{all}_95%_HPD={0.7458757,1.148996},height_median=1.1999999998124622E-5,height_range={0.0,4.300000001933313E-5},height=1.2072088955974479E-5]:28.232447000000004,2[&length_range={16.004005,41.265518},height_95%_HPD={0.0,2.700000001709668E-5},length_95%_HPD={20.922073,34.76612},IgrBrlens{all}_range={0.8613223,1.859122},length=28.19584838768108,IgrBrlens{all}_median=1.200649,length_median=28.232449000000003,IgrBrlens{all}=1.212581294490249,IgrBrlens{all}_95%_HPD={0.9643658,1.466607},height_median=1.1999999998124622E-5,height_range={0.0,4.300000001933313E-5},height=1.2072088955974479E-5]:28.232447000000004)[&length_range={1.632886,18.685625},length_95%_HPD={3.902024,12.749163},length=8.109956167291358,posterior=1.0,IgrBrlens{all}_median=0.32753319999999997,height_median=28.232459000000002,height_range={16.004021,41.26553800000001},height_95%_HPD={20.922081999999996,34.76612},IgrBrlens{all}_range={0.1629384,0.6974691},length_median=7.9685135,IgrBrlens{all}=0.3316228022363838,IgrBrlens{all}_95%_HPD={0.2254961,0.4486167},height=28.19586045977015]:8.211190500000004)[&length_range={22.543802,52.357035},length_95%_HPD={28.765977,46.135685},length=38.21616589105464,posterior=1.0,IgrBrlens{all}_median=1.294811,height_median=36.44364950000001,height_range={23.456858000000004,52.11625600000001},height_95%_HPD={27.666686,44.113808},IgrBrlens{all}_range={0.7682563,2.211874},length_median=38.5180795,IgrBrlens{all}=1.303474461444275,IgrBrlens{all}_95%_HPD={0.983642,1.630274},height=36.305816627061596]:39.458969999999994)[&height_95%_HPD={61.202113000000004,83.598469},length=0.0,posterior=1.0,height_median=75.9026195,height_range={54.553272,83.598469},height=74.52198251811596];

End;

**TipCalib1 (Maximum Clade Credibility tree)**

**
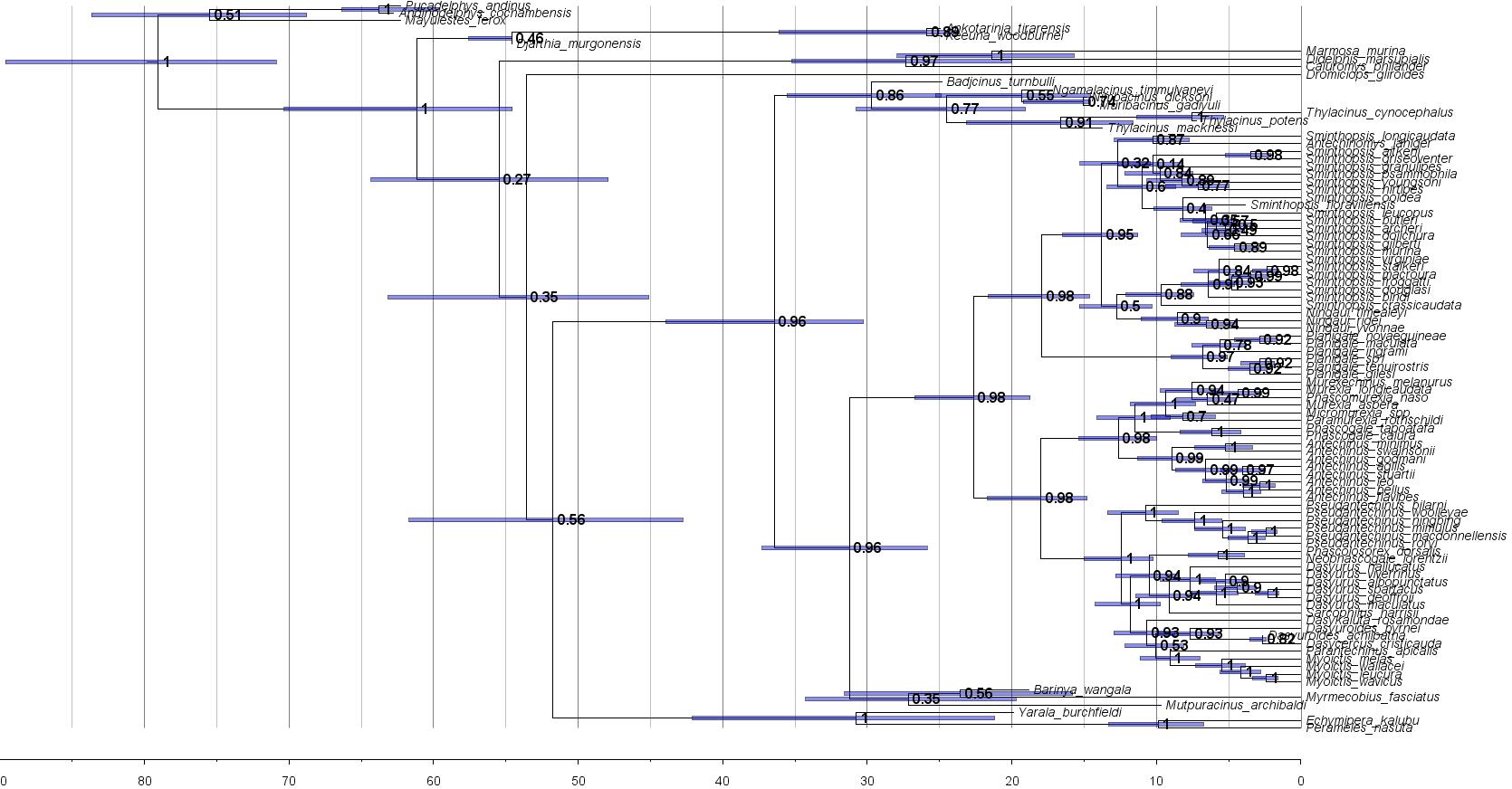
**

#NEXUS

Begin taxa;

Dimensions ntax=95;

Taxlabels

Pucadelphys_andinus

Andinodelphys_cochambensis

Mayulestes_ferox

Caluromys_philander

Didelphis_marsupialis

Marmosa_murina

Dromiciops_gliroides

Perameles_nasuta

Echymipera_kalubu

Thylacinus_cynocephalus

Myrmecobius_fasciatus

Dasykaluta_rosamondae

Dasycercus_cristicauda

Dasyuroides_byrnei

Dasyurus_hallucatus

Myoictis_wavicus

Neophascogale_lorentzii

Phascolosorex_dorsalis

Parantechinus_apicalis

Pseudantechinus_macdonnellensis

Sarcophilus_harrisii

Antechinus_flavipes

'Micromurexia_spp.'

Murexia_aspera

Murexia_longicaudata

Murexechinus_melanurus

Phascomurexia_naso

Paramurexia_rothschildi

Phascogale_tapoatafa

Antechinomys_laniger

Ningaui_ridei

Sminthopsis_crassicaudata

Planigale_gilesi

Dasyurus_albopunctatus

Dasyurus_geoffroii

Dasyurus_maculatus

Dasyurus_spartacus

Dasyurus_viverrinus

Myoictis_leucura

Myoictis_melas

Myoictis_wallacei

Pseudantechinus_bilarni

Pseudantechinus_mimulus

Pseudantechinus_ningbing

Pseudantechinus_roryi

Pseudantechinus_woolleyae

Antechinus_agilis

Antechinus_bellus

Antechinus_godmani

Antechinus_leo

Antechinus_minimus

Antechinus_stuartii

Antechinus_swainsonii

Phascogale_calura

Ningaui_timealeyi

Ningaui_yvonnae

Sminthopsis_aitkeni

Sminthopsis_archeri

Sminthopsis_bindi

Sminthopsis_butleri

Sminthopsis_dolichura

Sminthopsis_douglasi

Sminthopsis_gilberti

Sminthopsis_granulipes

Sminthopsis_griseoventer

Sminthopsis_hirtipes

Sminthopsis_leucopus

Sminthopsis_longicaudata

Sminthopsis_murina

Sminthopsis_macroura

Sminthopsis_psammophila

Sminthopsis_ooldea

Sminthopsis_virginiae

Sminthopsis_youngsoni

Sminthopsis_froggatti

Sminthopsis_stalkeri

Planigale_ingrami

Planigale_maculata

Planigale_novaeguineae

Planigale_tenuirostris

Planigale_sp1

Djarthia_murgonensis

Ankotarinja_tirarensis

Keeuna_woodburnei

Yarala_burchfieldi

Mutpuracinus_archibaldi

Barinya_wangala

Sminthopsis_floravillensis

Dasyuroides_achilpatna

Muribacinus_gadiyuli

Badjcinus_turnbulli

Nimbacinus_dicksoni

Thylacinus_macknessi

Thylacinus_potens

Ngamalacinus_timmulvaneyi

;

End;

Begin trees;

Translate

1 Pucadelphys_andinus,

2 Andinodelphys_cochambensis,

3 Mayulestes_ferox,

4 Caluromys_philander,

5 Didelphis_marsupialis,

6 Marmosa_murina,

7 Dromiciops_gliroides,

8 Perameles_nasuta,

9 Echymipera_kalubu,

10 Thylacinus_cynocephalus,

11 Myrmecobius_fasciatus,

12 Dasykaluta_rosamondae,

13 Dasycercus_cristicauda,

14 Dasyuroides_byrnei,

15 Dasyurus_hallucatus,

16 Myoictis_wavicus,

17 Neophascogale_lorentzii,

18 Phascolosorex_dorsalis,

19 Parantechinus_apicalis,

20 Pseudantechinus_macdonnellensis,

21 Sarcophilus_harrisii,

22 Antechinus_flavipes,

23 'Micromurexia_spp.',

24 Murexia_aspera,

25 Murexia_longicaudata,

26 Murexechinus_melanurus,

27 Phascomurexia_naso,

28 Paramurexia_rothschildi,

29 Phascogale_tapoatafa,

30 Antechinomys_laniger,

31 Ningaui_ridei,

32 Sminthopsis_crassicaudata,

33 Planigale_gilesi,

34 Dasyurus_albopunctatus,

35 Dasyurus_geoffroii,

36 Dasyurus_maculatus,

37 Dasyurus_spartacus,

38 Dasyurus_viverrinus,

39 Myoictis_leucura,

40 Myoictis_melas,

41 Myoictis_wallacei,

42 Pseudantechinus_bilarni,

43 Pseudantechinus_mimulus,

44 Pseudantechinus_ningbing,

45 Pseudantechinus_roryi,

46 Pseudantechinus_woolleyae,

47 Antechinus_agilis,

48 Antechinus_bellus,

49 Antechinus_godmani,

50 Antechinus_leo,

51 Antechinus_minimus,

52 Antechinus_stuartii,

53 Antechinus_swainsonii,

54 Phascogale_calura,

55 Ningaui_timealeyi,

56 Ningaui_yvonnae,

57 Sminthopsis_aitkeni,

58 Sminthopsis_archeri,

59 Sminthopsis_bindi,

60 Sminthopsis_butleri,

61 Sminthopsis_dolichura,

62 Sminthopsis_douglasi,

63 Sminthopsis_gilberti,

64 Sminthopsis_granulipes,

65 Sminthopsis_griseoventer,

66 Sminthopsis_hirtipes,

67 Sminthopsis_leucopus,

68 Sminthopsis_longicaudata,

69 Sminthopsis_murina,

70 Sminthopsis_macroura,

71 Sminthopsis_psammophila,

72 Sminthopsis_ooldea,

73 Sminthopsis_virginiae,

74 Sminthopsis_youngsoni,

75 Sminthopsis_froggatti,

76 Sminthopsis_stalkeri,

77 Planigale_ingrami,

78 Planigale_maculata,

79 Planigale_novaeguineae,

80 Planigale_tenuirostris,

81 Planigale_sp1,

82 Djarthia_murgonensis,

83 Ankotarinja_tirarensis,

84 Keeuna_woodburnei,

85 Yarala_burchfieldi,

86 Mutpuracinus_archibaldi,

87 Barinya_wangala,

88 Sminthopsis_floravillensis,

89 Dasyuroides_achilpatna,

90 Muribacinus_gadiyuli,

91 Badjcinus_turnbulli,

92 Nimbacinus_dicksoni,

93 Thylacinus_macknessi,

94 Thylacinus_potens,

95 Ngamalacinus_timmulvaneyi

;
[truncated: 362,580 more chars]
