# Supplementary material for: Total evidence phylogeny and evolutionary timescale for Australian faunivorous marsupials (Dasyuromorphia)
Source: BMC Evol Biol. 2017 Dec 4;17:240. doi: 10.1186/s12862-017-1090-0 (PMC5715987; doi:10.1186/s12862-017-1090-0)
Supplement: Supplementary file 7 — Summary of mean speciation and extinction rates estimated for modern dasyuromorphians using BAMM. (DOCX 13 kb) [file 12862_2017_1090_MOESM7_ESM.docx]

**Text S7. Summary of mean speciation and extinction rates estimated for modern dasyuromorphians using BAMM.**

BAMM analyses were run for 10 million generations, sampling every 2000 generations, with the first 10% discarded as burn-in, correcting for the incomplete sampling of dasyurid species. Values in brackets represent 95% confidence intervals.

| **Analysis** | **Clade** | **Speciation rate** | **Extinction rate** |
| --- | --- | --- | --- |
| NodeCalib1 | Dasyuromorphia | 0.13 (0.11-0.16) | 0.02 (0.004-0.05) |
|  | non-dasyurids | 0.10 (0.01-0.24) | 0.10 (0.004-0.28) |
|  | Dasyuridae | 0.14 (0.11-0.17) | 0.01 (0.001-0.03) |
|  | Dasyurini | 0.13 (0.11-0.16) | 0.01 (0.0004-0.03) |
|  | Phascogalini | 0.13 (0.11-0.16) | 0.01 (0.0003-0.03) |
|  | Planigalini | 0.14 (0.11-0.17) | 0.01 (0.0004-0.03) |
|  | Sminthopsini | 0.13 (0.11-0.16) | 0.01 (0.0005-0.03) |
| NodeCalib2 | Dasyuromorphia | 0.11 (0.09-0.13) | 0.02 (0.003-0.04) |
|  | non-dasyurids | 0.08 (0.009-0.20) | 0.09 (0.003-0.23) |
|  | Dasyuridae | 0.11 (0.09-0.14) | 0.009 (0.0009-0.03) |
|  | Dasyurini | 0.10 (0.07-0.12) | 0.008 (0.0004-0.02) |
|  | Phascogalini | 0.11 (0.09-0.13) | 0.008 (0.0005-0.03) |
|  | Planigalini | 0.12 (0.09-0.14) | 0.008 (0.0004-0.03) |
|  | Sminthopsini | 0.11 (0.09-0.13) | 0.008 (0.0005-0.02) |
| TipCalib1 | Dasyuromorphia | 0.14 (0.11-0.17) | 0.02 (0.004-0.05) |
|  | non-dasyurids | 0.10 (0.01-0.24) | 0.10 (0.004-0.29) |
|  | Dasyuridae | 0.14 (0.12-0.17) | 0.01 (0.001-0.03) |
|  | Dasyurini | 0.14 (0.11-0.17) | 0.01 (0.0006-0.03) |
|  | Phascogalini | 0.14 (0.11-0.17) | 0.01 (0.0006-0.03) |
|  | Planigalini | 0.15 (0.12-0.18) | 0.01 (0.0005-0.03) |
|  | Sminthopsini | 0.14 (0.11-0.17) | 0.01 (0.0006-0.03) |
| TipCalib2 | Dasyuromorphia | 0.14 (0.11-0.17) | 0.02 (0.005-0.05) |
|  | non-dasyurids | 0.09 (0.01-0.24) | 0.11 (0.006-0.29) |
|  | Dasyuridae | 0.15 (0.12-0.18) | 0.01 (0.001-0.03) |
|  | Dasyurini | 0.14 (0.11-0.17) | 0.01 (0.0005-0.03) |
|  | Phascogalini | 0.14 (0.11-0.17) | 0.01 (0.0005-0.03) |
|  | Planigalini | 0.15 (0.12-0.18) | 0.01 (0.0005-0.03) |
|  | Sminthopsini | 0.14 (0.11-0.17) | 0.01 (0.0006-0.03) |
| TipNodeCalib1 | Dasyuromorphia | 0.15 (0.12-0.19) | 0.02 (0.004-0.06) |
|  | non-dasyurids | 0.11 (0.01-0.30) | 0.13 (0.005-0.36) |
|  | Dasyuridae | 0.16 (0.13-0.19) | 0.01 (0.001-0.04) |
|  | Dasyurini | 0.15 (0.12-0.18) | 0.01 (0.0006-0.03) |
|  | Phascogalini | 0.15 (0.12-0.18) | 0.01 (0.0006-0.03) |
|  | Planigalini | 0.16 (0.13-0.19) | 0.01 (0.0005-0.03) |
|  | Sminthopsini | 0.15 (0.12-0.18) | 0.01 (0.0006-0.03) |
| TipNodeCalib2 | Dasyuromorphia | 0.15 (0.12-0.18) | 0.02 (0.004-0.05) |
|  | non-dasyurids | 0.11 (0.01-0.28) | 0.12 (0.004-0.33) |
|  | Dasyuridae | 0.15 (0.12-0.19) | 0.01 (0.001-0.03) |
|  | Dasyurini | 0.15 (0.12-0.19) | 0.01 (0.0006-0.03) |
|  | Phascogalini | 0.16 (0.13-0.19) | 0.01 (0.0006-0.03) |
|  | Planigalini | 0.16 (0.13-0.19) | 0.01 (0.0006-0.03) |
|  | Sminthopsini | 0.15 (0.12-0.18) | 0.01 (0.0006-0.03) |
