# Supplementary material for: Total evidence phylogeny and evolutionary timescale for Australian faunivorous marsupials (Dasyuromorphia)
Source: BMC Evol Biol. 2017 Dec 4;17:240. doi: 10.1186/s12862-017-1090-0 (PMC5715987; doi:10.1186/s12862-017-1090-0)
Supplement: Supplementary file 8 — Potentially problematic MT-CYB sequences used by May-Collado et al... [21]. (DOCX 13 kb) [file 12862_2017_1090_MOESM8_ESM.docx]

**Text S8. Potentially problematic pre-2000 *MT-CYB* sequences that show low (≤95%) similarity to more recent sequences from the same species**

| Species | Older sequence (and year of publication) | Newer sequence (and year of publication) | Sequence similarity |
| --- | --- | --- | --- |
| *Dasyuroides byrnei* | U07579 (1992) | KJ868109 (2014) | 94% |
| *Dasyurus hallucatus* | M99460 (1992) | AY795973 (2006) | 88% |
| *Dasyurus maculatus* | M99461 (1992) | KJ780047 (2015); KJ780048 (2015); KJ780049 (2015) | 91-93% |
| *Micromurexia habbema* | U07576 (1993) | KJ868125 (2014) | 88% |
| *Myrmecobius fasciatus* | U82329 (1993) | FJ515782 (2009) | 90% |
| *Neophascogale lorentzii* | U07585 (1993) | KJ868130 (2014) | 90% |
| *Ningaui ridei* | U07586 (1993) | KJ868131 (2014) | 94% |
| *Paramurexia rothschildi* | U07583 (1993) | KJ868134 (2014) | 95% |
| *Parantechinus apicalis* | M99457 (1992) | KJ868135 (2014) | 92% |
| *Phascomurexia naso* | U23461 (1996) | KJ868145 (2014) | 93% |
| *Planigale gilesi* | U07589 (1993) | KJ868147 (2014) | 93% |
| *Pseudantechinus bilarni* | U07588 (1993) | KJ868149 (2014) | 91% |
| *Pseudantechinus macdonnellensis* | M99458 (1992) | KJ868150 (2014) | 92% |
| *Pseudantechinus woolleyae* | U07593 (1993) | EU086675 (2007) | 94% |
| *Sarcophilus harrisii* | M99465 (1992) | KJ868154 (2014) | 93% |
| *Sminthopsis crassicaudata* | M99463 (1992) | AY795974 (2006) | 95% |
